# The ribosomal RNA transcription landscapes of *Plasmodium falciparum* and related apicomplexan parasites

**DOI:** 10.1093/nar/gkaf641

**Published:** 2025-07-08

**Authors:** Gunnar Mair, Julia L Daiß, Christoph Engel, Konstantin I Panov

**Affiliations:** School of Biological Sciences, Queen’s University Belfast, Belfast, BT9 5D, United Kingdom; Department of Biomedical Sciences, College of Veterinary Medicine, Iowa State University, Ames, IA 50011, United States; Regensburg Center for Biochemistry, University of Regensburg, 93053 Regensburg, Germany; Regensburg Center for Biochemistry, University of Regensburg, 93053 Regensburg, Germany; School of Biological Sciences, Queen’s University Belfast, Belfast, BT9 5D, United Kingdom; Patrick G Johnston Centre for Cancer Research, Queen’s University Belfast, Belfast, BT9 7AE, United Kingdom

## Abstract

Ribosome biogenesis is essential for the rapid proliferation and life cycle transitions of *Plasmodium falciparum*, the causative agent of malaria. In eukaryotes, ribosomal RNA synthesis is carried out by RNA polymerase I (Pol I), highly specialized transcriptional machinery. This review provides a comparative analysis of Pol I transcription apparatus in yeast and humans, serving as a reference framework to examine its evolutionary divergence in *P. falciparum* and related apicomplexans and alveolates. Bioinformatic analyses revealed that some of these organisms lack any identifiable homologues or orthologs of several canonical eukaryotic transcription factors essential for Pol I-mediated transcription, including initiation factor RRN3, activator UBF, and all specific subunits of the promoter recognition complexes. Interestingly, the parasite retains core Pol I subunits, incorporating unique parasite-specific structural domains characterized through AI-based protein complex modeling of *P. falciparum* Pol I. These adaptations may compensate for the absence of traditional regulatory factors, enabling the parasite to employ distinct mechanisms for promoter recognition and transcription initiation. The substantial differences between parasite and host Pol I transcription machinery create potential targets for therapeutic intervention with parasite-specific elements representing potential drug targets. By integrating evolutionary, structural, and functional perspectives, this review advances our understanding of Pol I transcription in alveolates and its implications for the development of novel antimalarial strategies.

## Graphical Abstract

**Graphical Abstract:**
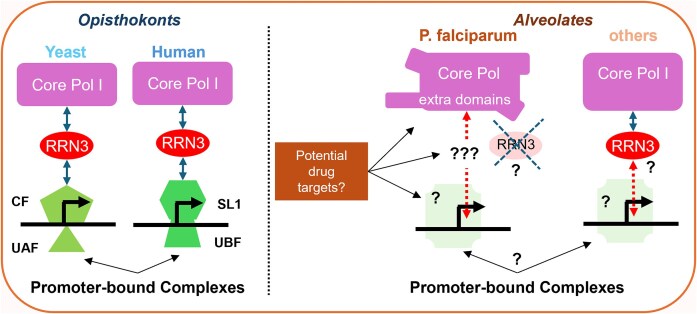

## Introduction

**Figure 1.**
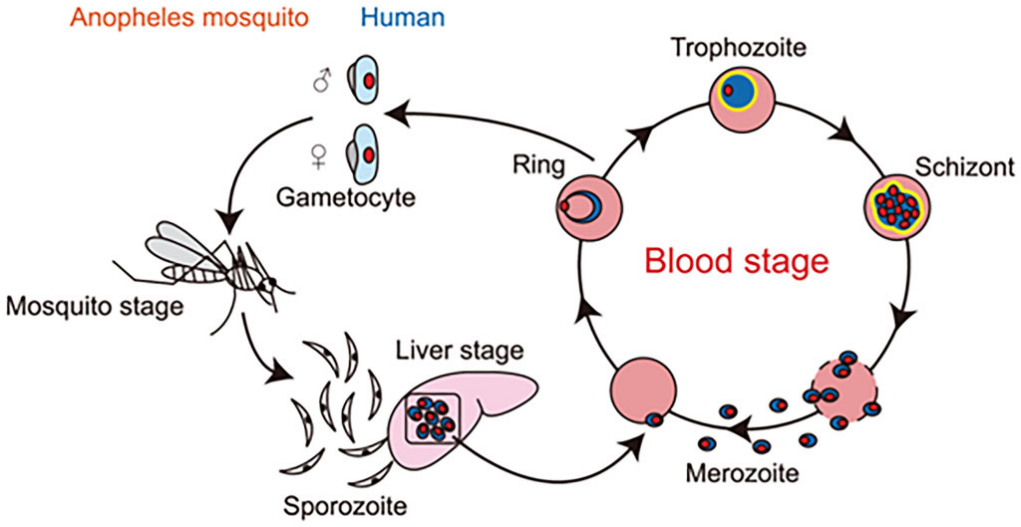
The malaria parasite life cycle. A human malaria infection is initiated with the injection of sporozoites into the skin or circulatory system during the blood meal of a female *Anopheles* mosquito. The next port of call is the liver; here a single sporozoite produces thousands of daughter cells in an infected hepatocyte; these forms are called merozoites; infectious to the red blood cell, they are the cause of the clinical manifestations of malaria caused by the repeated infection of the erythrocytes. Transmission to the mosquito relies on the formation of male and female gametocytes within the red blood cell; following a blood meal by a female *Anopheles* gametocytes differentiate into mature male and female gametes and fuse to produce a zygote; the zygote develops into the motile ookinete, establishes a sessile oocyst, and in turn the sporozoites; they populate the salivary glands, which completes the life cycle [1, 2].

The *Plasmodium* life cycle is complex and follows a rigid developmental program in the human and mosquito host. The successful transmission of this unicellular parasite relies on the timely generation of morphologically distinct and uniquely adapted parasite forms that are produced in the different host cell environments [1, 2]; a suite of ∼5000 protein-coding genes, which are contained across 14 chromosomes in the haploid genome, provides the blueprint (Fig. 1). During its life cycle the parasite develops three motile and infectious life cycle stages within the mosquito and human hosts: first the merozoite which sustains the chronic infection cycle of the red blood cell; then the ookinete, which develops in the mosquito midgut from ingested gametocytes and transverses the midgut epithelium to establish the oocyst; and finally the sporozoites, which grow in the aforementioned oocyst and move to the mosquito’s salivary glands from where they are injected during a mosquito blood meal into the human host’s skin or circulatory system; the sporozoite’s final destination is the liver, where first-generation red-blood-cell-infectious merozoites are produced in an infected hepatocyte [3]. Each of these invasion and transmission events is followed by an expansion of the surviving parasite population: the newly invaded merozoite in the red blood cell, the ookinete-turned-oocyst in the midgut epithelium, and the liver-resident sporozoite each yield tens to tens-of-thousands of clonal offspring through multiple fission (schizogony). These developmental programs require significant cell growth and cell division rates, which in turn must rely on a sufficient supply of ribosomal RNA (rRNA) for ribosome biogenesis (RiBi). How this is accomplished is largely unexplored.

Protein synthesis is a fundamental process and essential for life. Inhibition of translation by the cytoplasmic ribosome is therefore an attractive and widely used intervention strategy against many pathogens [4, 5]. Key differences between bacterial and human ribosomes play a crucial role in the success of antibiotics. Notably, aminoglycosides, macrolides, and tetracyclines are potent inhibitors of bacterial protein translation [4, 6]. Translation inhibitors have already been trialed *in vitro* against the malaria parasite *P. falciparum*, where potential antimalarials target essential components of the translation process such as the aminoacyl-transfer RNA (tRNA) synthetase or translation elongation factor 2 [7–11].

The other way to affect translational capacity is to target the initial stages of RiBi such as rRNA transcription, processing, and ribosome assembly. Small molecule inhibitors that selectively target rRNA transcription were first developed in 2009 as a potential anticancer therapeutic approach [12, 13]. These compounds aimed to target a key stage of RiBi, rRNA synthesis, by inhibiting the RNA polymerase I (Pol I). Critically, inhibition of Pol I by these drugs in cancer cells activates the nucleolar surveillance response pathway, which in turn leads to cell cycle arrest and/or activation of apoptosis [14–18].

Three compounds (quarfloxin, CX-5461, and BMH-21) inhibit mammalian Pol I and have recently been shown to act as strong inhibitors of Pol I in the unicellular parasite *Trypanosoma brucei*, the causative agent of sleeping sickness (a protozoan unrelated to *P. falciparum*). These drugs cause rapid cell death of bloodstream forms with an almost complete inhibition of rRNA transcription. In addition, transcription of the *vsg* (variant surface glycoprotein) gene, a rare example of an RNA Pol I-transcribed protein-coding gene that yields the major protective surface coat of this blood-borne pathogen, is negatively affected [19].

Although quarfloxin and CX-5461 also reduce *P. falciparum* viability in cell culture and act *in vivo* in the *P. berghei* mouse model, the work published by Merrick and colleagues claims that Pol I-mediated transcription of rRNA in *Plasmodium* is apparently unaffected by quarfloxin treatment [20, 21]. The mode of action or molecular targets of these drugs on the malaria parasite are therefore still unknown. However, quantitative polymerase chain reaction-based detection of rRNA transcripts is known to be error-prone because it relies on fast degradation of the 5′-ETS [external transcribed spacer, a noncoding part of the pre-ribosomal RNA (pre-rRNA) transcript] of the pre-rRNA, which is not always the case. Therefore, the effect of Pol I inhibitors on rRNA transcription in *Plasmodium* may need to be further verified using metabolic labeling techniques instead.

## The protein and RNA components of the *Plasmodium* ribosome

The ribosome is an essential, megadalton-large RNA and protein complex that executes protein translation in all cells. It uses the genetic information contained in the protein-coding genes within a cell’s DNA, copied into messenger RNA (mRNA), to produce the cell’s proteins. tRNA supply the required amino acid to the growing polypeptide chain. In eukaryotes, the 80S ribosome is composed of the small 40S and large 60S ribosomal subunits; the Svedberg sedimentation coefficient S characterizes each particle’s size which is defined by its specific protein and rRNA components. The 40S subunit scans the mRNA and is composed of the 18S rRNA and 30+ proteins, while the larger 60S subunit, which executes peptide bond formation, is composed of almost 50 proteins and three rRNAs: the 5S, 28S, and 5.8S rRNA [22].

The cellular RNAs engaged in eukaryotic protein translation are transcribed by three different but related DNA-dependent RNA polymerases: Pol I, Pol II, and Pol III. These enzymes have likely evolved from the simpler bacterial five-subunit (α_2_ββ′ω) and a more complex 11–13 subunit RNA polymerase found in the Archaea [23–25].

mRNA transcribed from protein-coding genes and those that function in pre-mRNA splicing (U1, U2, U4, U5), but also miRNAs and long noncoding lncRNAs are examples of Pol II-transcribed genes. The noncoding rRNAs are products of Pol I and Pol III. In most eukaryotes, Pol I transcribes a single gene: the pre-rRNA gene that unites the coding sequences for the 18S, 5.8S, and 28S rRNAs. A notable exception is the variant surface glycoprotein *vsg* genes in trypanosomes, which encode the dominant surface coat of this parasite [26]. Pol III produces the 5S rRNA and other small RNAs such as transfer RNAs (tRNAS) and small nucleolar RNAs (snoRNAs), U6 small nuclear RNA (snRNA), and the signal recognition particle RNA.

**Figure 2.**
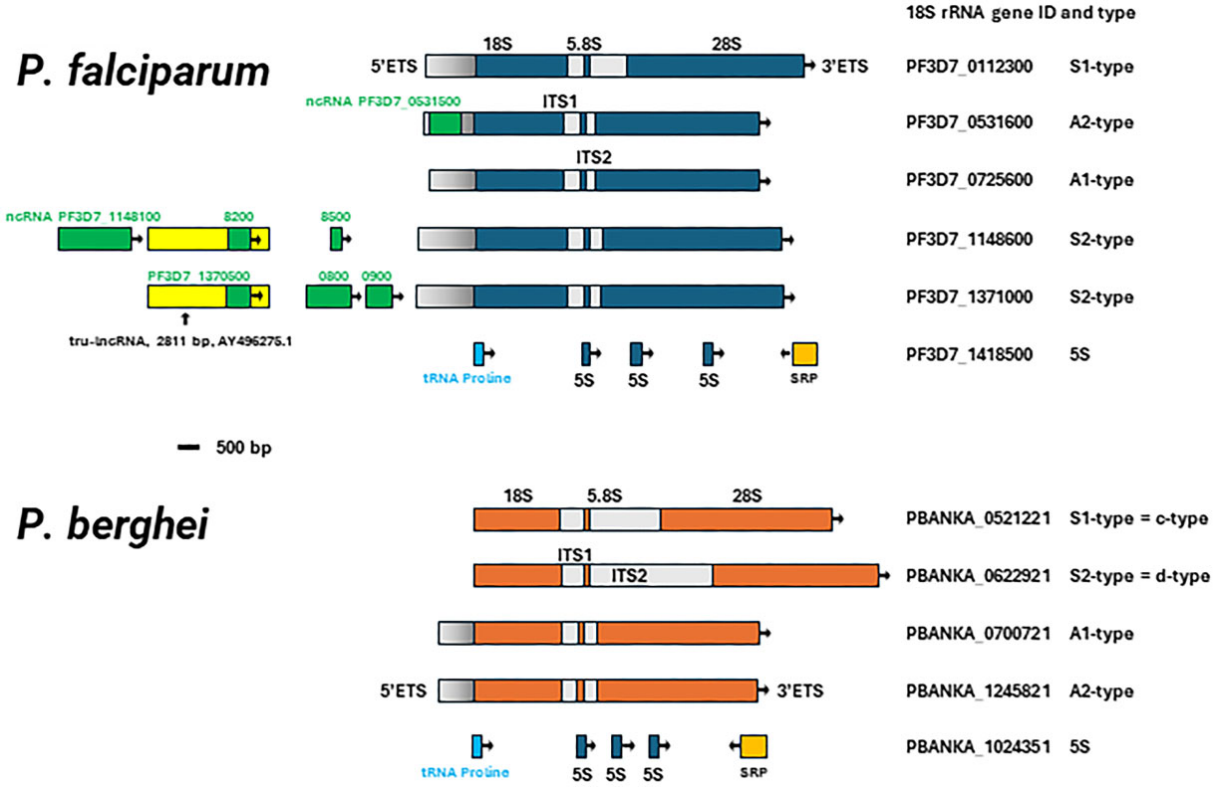
Ribosomal DNA *loci* in *P. falciparum* and *P. berghei*. The stage-specific expression patterns (A- and S-type) are indicated on the right with the corresponding gene identifiers. The five *P. falciparum* 5′ETS are from Fang *et al.* [37]; the two *P. berghei* 5′ETS (all shown as gray rectangles) are from van Spaendonk *et al.* [36]. The 2811 bp long noncoding RNA tru-lncRNA is indicated as a yellow rectangle. Seven noncoding RNAs are indicated as green rectangles. Arrows indicate direction of transcription. SRP, signal recognition particle; ncRNA, noncoding RNA; ITS, internal transcribed spacer; ETS, external transcribed spacer; and tRNA, transfer RNA. Drawn to scale from data available at plasmodb.org. The tru-lncRNA GenBank accession number: AY496275.

The protein and RNA components of the *Plasmodium* cytoplasmic ribosome are evolutionarily conserved; orthologs to almost all of the 33 small and all 47 large ribosomal proteins known in humans [22] are also present in *P. falciparum*, the most prevalent cause of malaria in humans [27, 28]. On the other hand, there is a conspicuous dearth of rRNA genes within the *Plasmodium* genus. While yeasts like *Saccharomyces cerevisiae* contain some 150–200 rRNA genes and humans up to 600 copies [29], *P. falciparum* makes do with a mere five ribosomal DNA *loci*(rDNA); the widely used rodent models *P. berghei* and *P. yoelii* utilize four to meet the cell’s demand for protein translation having proliferation rates comparable with yeast [30, 31]. In *S. cerevisiae*, all rRNA genes can be found on chromosome XII, and in humans they are clustered on the acrocentric human chromosomes 13, 14, 15, 21, and 22, while a 5S unit is localized on chromosome 1. Typically, in eukaryotes, rRNA genes are encoded as part of rDNA repeats that are separated by an intergenic spacer (IGS), and multiple copies of rDNA repeats are arranged in a head-to-tail orientation (Supplementary Fig. S1) [32–34]. In contrast, all *Plasmodium* rRNA genes exist as single copies. They are located on chromosomes 5, 6, 7, and 12 in *P. berghei* and *P. yoelii*, and on chromosomes 1, 5, 7, 11, and 13 of *P. falciparum* (Fig. 2). Two additional, yet incomplete units that lack the 28S rRNA are present on chromosome 8 of *P. falciparum*.

The single-copy rDNA *loci* of *P. falciparum* and *P. berghei* lack the IGS that separates neighbouring rRNA genes in yeast and humans. Instead, the coding regions of the pre-rRNA transcript are preceded by often large, several kilobase-long regions upstream of transcription start site (TSS); in two instances they are predicted, and in one instance have experimentally been shown, to be the origin of noncoding RNA species [35]. The TSSs of many *P. berghei* and *P. falciparum* rRNA genes have been mapped; in *P. berghei* they include an 837 base pair long 5′ETS in the two almost identical so-called A1- and A2-type ribosomal genes [36], while the 5′ETS of five *P. falciparum* pre-rRNAs range from 1042 to 1318 base pairs [37] (Fig. 2). The extent of the individual 3′ETS sequences is still unknown.

The rRNA genes in *Plasmodium* (four *P. berghei* and five *P. falciparum*) are under strict transcriptional control to ensure efficient and accurate ribosome production. They are characterized by discrete transcriptional histories across the parasite’s life cycle in the human (or mouse) and mosquito host [38] and are broadly classified as asexual (blood stage) A-type or sporozoite (mosquito stage) S-type (Fig. 2). This includes two nearly identical A1- and A2-types transcribed in the human host during asexual replication in the red blood cell; secondly, the S1-type in the sexual stage gametocyte (the form that is transmitted from the mammalian host to the mosquito vector); and finally, S2 in the mosquito sporozoite. The *falciparum* genome harbors two S2 loci, one on chromosome 11 and a second one on chromosome 13; they are almost identical [31, 38–43].

The so-called A-types of the parasite first emerge during the initial infection in the liver stage. They continue to dominate throughout the intraerythrocytic (asexual) cycle in the red blood cell, especially in the ring stage [30, 37, 44]. S1-type transcription then peaks in the gametocyte and likely sustains the ensuing ookinete development together with translationally repressed (stored) mRNA [45], while S1-type defines sporozoite formation within the infected mosquito. S1 expression is highest in the salivary gland sporozoite and subsequently declines during development in the host hepatocyte. The transcription of A- and S-type rDNA are not mutually exclusive: during blood stage development within the red blood cell, A1 and A2 clearly predominate, but S1 and S2 expression reaches almost 10% of A-type transcription levels [37, 46]. However, this could be due to the detection of S-type transcripts from sexual precursor cells or gametocytes, which are also present within red blood cells of the circulatory system.

A single *P. berghei* S-type locus (S1- or S2-type; also classified as c or d-type) provides sufficient RiBi for sporozoite development in the mosquito [42]. This is not the case in the related *P. yoelii* parasite; disruption of the d-type 18S rRNA causes a reduction in oocyst numbers and a complete failure to produce salivary gland sporozoites [47]. Both A1 and A2 types are likely essential for parasite development within the red blood cell, as experimental gene deletion has failed [42]; surprising as the progeny produced in one infected red blood cell from a single parasite during the 24-h asexual blood stage development does not exceed thirty-two; in the mosquito oocyst and infected hepatocyte, hundreds to thousands of daughter cells are produced.

**Figure 3.**
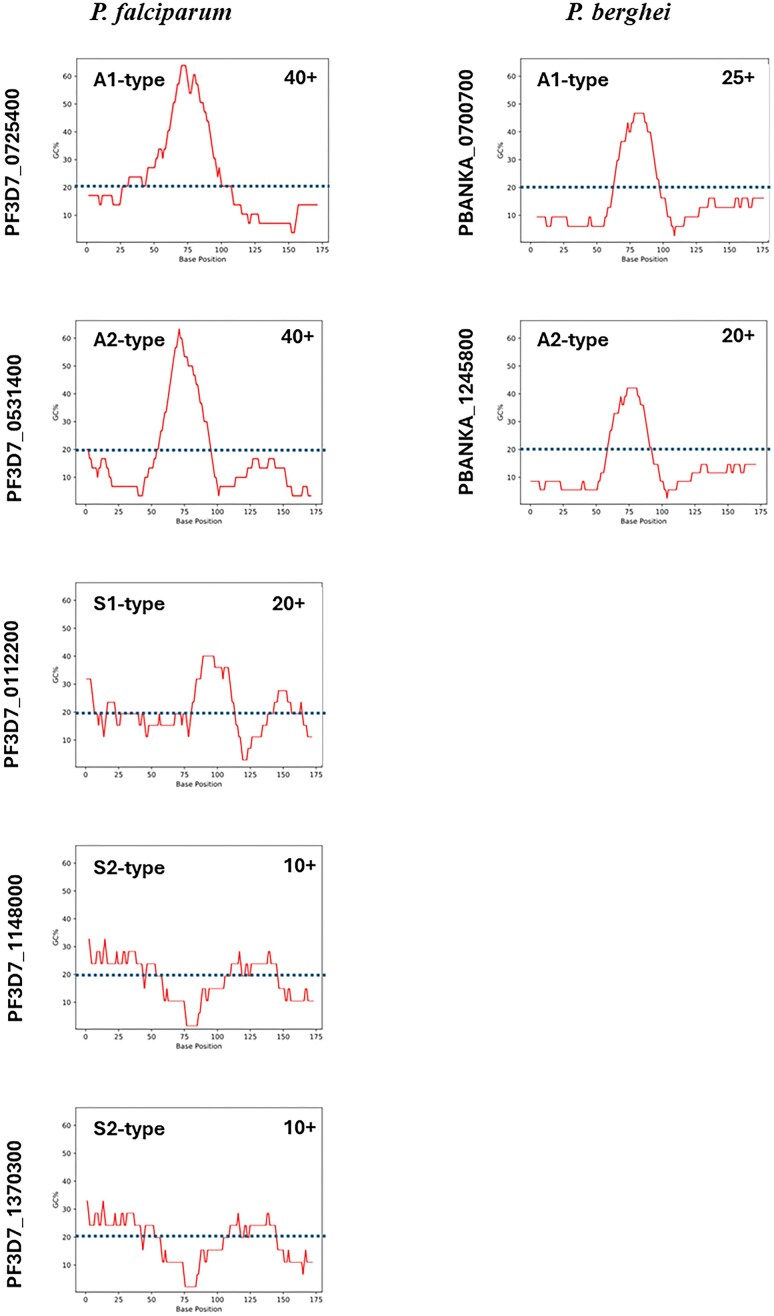
GC-content surrounding experimentally determined and proposed TSSs. TSSs are positioned at base position 101 in all sequences within the 200 base pair window. Plot generated at https://en.vectorbuildercom/tool/gc-content-calculator.html. The average GC-content is indicated by a line (https://www.ncbi.nlm.nih.gov/pmc/articles/PMC5857377/): *P. berghei*= 22.04%; *P. falciparum*= 19.34%.

These tightly regulated transcription patterns indicate a form of functional ribosome specialization that may be demanded by the unique environmental and nutritional conditions that exist in the mammalian and mosquito hosts, as well as the different host cell types [40, 48–51]. To produce such significant changes in rRNA content in the cell across the life cycle, the parasite likely possesses intricate mechanisms of transcriptional control or post-transcriptional processing mechanisms. Alas, the activation or silencing mechanisms of different rDNA loci across the *Plasmodium* life cycle are largely uncharted. The TSS of both *P. berghei* and *P. falciparum* A-type rRNAs are clearly marked by a GC-rich environment [36, 37] (Fig. 3). This is consistent with the core promoter environments of yeast and human rRNA promoters, which are defined by a GC-rich region found in the −45 to +20 region relative to the TSS. Intriguingly, GC-rich regions appear to be missing in the S-type genes, suggesting that preinitiation complex (PIC) formation and Pol I recruitment to the rDNA locus rely on a different, stage-specific set of transcription factors and/or specific chromatin modifiers. Indeed, histone acetylation and a noncoding RNA are the only factors specifically implied to impact rRNA levels in *P. falciparum*.

Sirtuin Sir2a (a histone deacetylase HDAC) negatively affects the transcript levels from all rDNA loci in *P. falciparum* [46]. In the absence of Sir2a—but not its relative Sir2b—H3K9 acetylation across all rRNA genes increases two-fold with a simultaneous upregulation of both A- and S-type rRNA in cultured blood-stage parasites. Accordingly, an additional, experimentally introduced copy of Sir2a reduces rRNA levels. This is akin to the inhibitory role of SIRT1, rather than the SIRT7-mediated activation of human rRNA transcription [52, 53]. The Sir2a gene is, however, neither required for asexual life cycle progression in *P. falciparum* [54] nor the rodent model [55].

Instead, *P. berghei* Δ*sir2a* parasites fail to produce oocysts and in turn sporozoites in the mosquito (RMgm-3609; RMgm-4435 unpublished; pberghei.eu; Religa, Janse, Waters).

In *P. falciparum*, stage-specific S-type rRNA processing involves a 2811-nucleotides-long noncoding RNA called tru-lncRNA (GenBank AY496275.1) [35] (Fig. 2). This transcript originates upstream of the two almost identical S1 rDNA *loci* on chromosomes 11 and 13 and is cleaved into two smaller 1.3 and 1.6 kb species. During *in vitro* culture, the transcription of tru-lncRNA significantly increases alongside S-type transcription when the parasite experiences an experimental temperature shift that mimics the parasite’s transmission from the 37°C environment of the human host to the ambient temperature of the mosquito [35]. The transcript appears to be involved in S-type rRNA maturation; mutants with disrupted tru-lncRNAs *loci* fail to produce S-type 18S rRNA. The molecular mechanism underlying this regulation is unknown. In mouse cells, transcripts originating from the IGS affect the epigenetic environment of the genome between rRNA genes and so inhibit rRNA transcription [56]. Thus far there is no evidence for the presence of tru-lncRNA-related RNAs in other malaria parasites, although they undergo comparable transcriptional shifts in rRNA transcription across the life cycle. The genomic region upstream of the 18S rRNA is certainly not conserved between these species.

The 5S rRNA (119 nucleotides long in *P. falciparum*), which is a component of the large ribosomal subunit, is transcribed by Pol III. Both *P. berghei* chromosome 10 and *P. falciparum* chromosome 14 encode a single 5S rRNA locus with three 5S rDNA genes [57] arranged in a head-to-tail orientation; they are flanked by two additional Pol III transcripts: a proline tRNA and the signal recognition particle RNA (7SL, 6S, or 4.5S RNA) (Fig. 2).

## Identification of components of Pol I transcription machinery in *P. falciparum* and related apicomplexans

Our current understanding of the eukaryotic Pol I transcription machinery is primarily based on studies conducted in unicellular fungi (such as yeast) and mammals (including *Homo sapiens*). While the Pol I enzymatic complex remains highly conserved in these two groups, the promoter-bound transcription factors show significant differences. By studying the presence or absence of Pol I machinery components in evolutionarily distant unicellular organisms [58], we can gain insights into the evolution of the crucial step in RiBi—rRNA transcription.

Here, we analyzed the phylogenetic distribution of Pol I factors in publicly available protein databases (specifically plasmodb.org release 67 and orthomcl.org release 6.20, both from 21 February 2024) with an aim to identify orthologs of experimentally and structurally well-characterized components of human and yeast Pol I in the human malaria parasite *P. falciparum* and selected related organisms. Our data identify the shared, divergent, and missing constituents in the human malaria parasite and define the evolutionary conservation of key protein elements, which have been recognized to be required for efficient ribosomal DNA transcription in yeast and the malaria parasite host *H. sapiens*. Owing to the recent progress in defining the phylogenetic relationships of eukaryotic protists within the *Alveolata* and the presence of many parasites of medical and veterinary importance in that group [59–61], we identify the distribution of these proteins in additional members of this evolutionary branch. Alveolates are a group of protists that include both free-living and parasitic unicellular organisms that share a common characteristic: flattened, sac-like vesicles, or so-called alveoli, positioned beneath their plasma membrane [62]. From this group, we included the following thirteen species: the free-living ciliate *Tetrahymena thermophila*; the two photosynthetic chrompodellids, *Chromera velia* and *Vitrella brassicaformis*; and ten diverse, apicomplexan parasites of both medical and veterinary importance: *Gregarina niphandrodes*, *Cryptosporidium hominis*, *Eimeria tenella*, *Toxoplasma gondii*, *Sarcocystis neurona*, *Theileria equi*, *Babesia microti*, *Haemoproteus tartakovskyi*, the rodent malaria model *P. berghei*, and the human malaria parasite *P. falciparum*. These organisms provide a diverse representation of the major phyletic groups within this extensive phylum.

**Figure 4.**
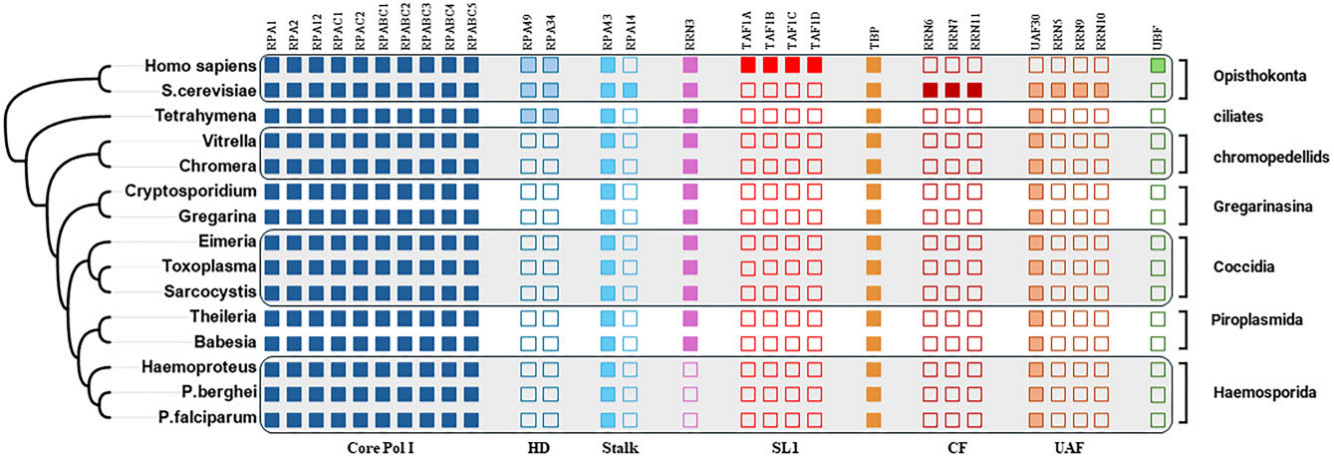
The phylogenetic distribution of RNA Pol I transcription factors in selected eukaryotes. Core Pol I—core Pol I subunits; HD—subunits of Pol I-specific heterodimer; Stalk—subunits forming stalk domain; RRN3—Pol I associated transcription factor; SL1—subunits of human Selectivity factor 1; TBP—TATA-binding protein; CF—subunits of yeast Core Factor; UAF—subunits of yeast Upstream Activating Factor; and UBF—human Upstream Binding Factor. Phylogenetic tree of alveolates highlighting the relationships of selected apicomplexan parasites, chrompodellids, and the ciliate *T. thermophila* in comparison to Opisthokonta (*H. sapiens* and *S. cerevisiae*; adapted and generalized from Janouškovec at al. [62]). The headers refer to the components required for ribosomal DNA transcription identified in *H. sapiens* and yeast. Filled and empty squares indicate the presence and absence of proteins. The species refer to *Babesia microti* strain RI, *Chromera velia* CCMP2878, *Cryptosporidium hominis* isolate TU502_2012, *Eimeria tenella* Houghton 2021, *Gregarina niphandrodes* unknown strain, *Haemoproteus tartakovskyi* strain SISKIN1, *Homo sapiens* REF, *P. falciparum* 3D7 and *P. berghei* ANKA, *Saccharomyces cerevisiae* S288C, *Sarcocystis neurona* SN3, *Tetrahymena thermophila* (strain SB210), *Theileria equi* strain WA, *Toxoplasma gondii* ME49, and *Vitrella brassicaformis* CCMP3155, as indicated on https://orthomcl.org/orthomcl/app/ (release 6.21 of 7 May 2024).

We selected a number of human and yeast proteins known to be involved in rRNA transcription (Supplementary Table S1), encompassing components from the Pol I enzyme core, promoter-bound transcription factors, and proteins experimentally shown to impact transcriptional efficiency. We also indicate whether the selected protein is essential for certain species. We queried OrthoMCL and used BlastP to identify orthologs of these sequences in the protein-coding genomes of *P. falciparum* and related alveolates. Through this bioinformatics analysis, we identified a total of 23 orthologs in the human malaria parasite, as well as orthologs in other alveolates (Fig. 4 and Supplementary Table S1).

The majority of the identified factors are highly conserved and are assigned to the same ortholog groups as the corresponding proteins from *H. sapiens* or yeast, fifteen to different ones (Supplementary Table S1). Some proteins were missing; out of those, we identified four in a closely related strain (*Sarcocystis neurona* and *Cryptosporidium hominis*); seven proteins were theoretically identified in transcript shotgun assembly or genome sequences; one missing protein component from *T. thermophila* could be identified in the related species *T. borealis* (Supplementary Table S1).

Throughout this manuscript, we provide multiple sequence alignments to highlight the degree of conservation between those members identified in our candidate organisms and *H. sapiens*. Supplementary Fig. S2.1 through to S2.17 highlights pairwise *Homo sapiens* (or *S. cerevisiae* if there is no human protein) and *P. falciparum* alignments, while Supplementary Figs. S3.1 through to S3.18 provide a global alignment view of all proteins identified in the search across all queried organisms. In Supplementary Table S2, we listed molecular weights and lengths of components of Pol I transcription machinery identified in this work and elsewhere, including human and yeast proteins.

## RNA Pol I

**Figure 5.**
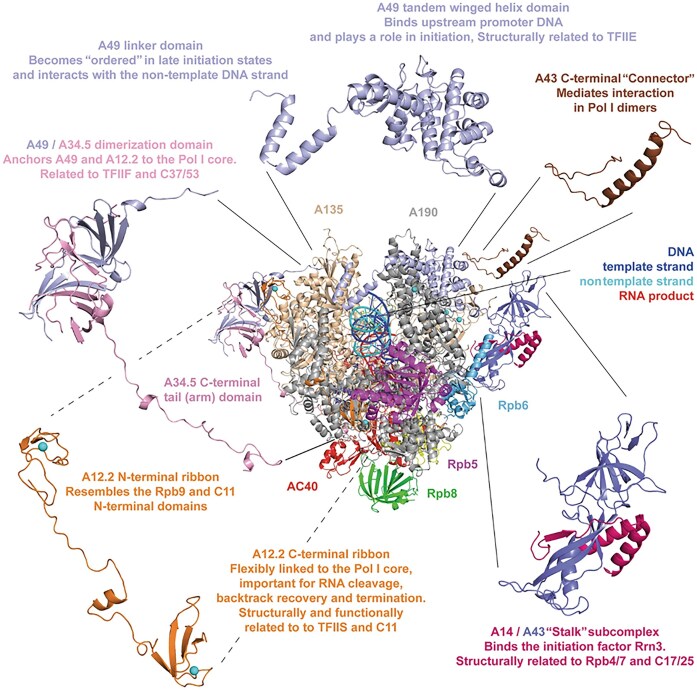
Ribbon model of RNA Pol I highlighting specific subunits and domains. The “front view” looks along the incoming (“downstream”) DNA. Subunits not visible in the front view but present in the model are AC19, Rpb10, and Rpb12. Cyan spheres depict coordinated zinc atoms. Taken from Pilsl and Engel [63].

The Pol I enzyme is an evolutionarily conserved multiprotein complex composed of 10 “core” subunits that form a horseshoe or claw-shaped structure to accommodate the rDNA template (Fig. 5, taken from [63]) [64, 65] and (see Supplementary Table S1 for yeast and human components). These “core” subunits are also highly conserved across all queried apicomplexan species and are readily identifiable in these organisms based on sequence similarity (Fig. 4 and Supplementary Table S1) [57, 58]. Three of these subunits are Pol I-specific, with functional homologs in Pol II and Pol III, while others are shared between two polymerases [e.g. RPAC1 (AC40) and RPAC2 (AC19) shared with Pol III] or among all three [e.g. RPABC1 through RPABC5 (ABC27-ABC10), which are common to Pol I, II, and III].

## “Core” subunits

In yeast and mammals, the two Pol I-specific largest subunits, RPA1 (A190) and RPA2 (A135), form the catalytic center of the polymerase (Fig. 5), and the identified *P. falciparum* orthologs are expected to fulfill the same role. Interestingly, the two main catalytic subunits, along with RPA12, are substantially larger than their human and yeast orthologs, while all other subunits are of similar size across the three species (Supplementary Table S2).

**Figure 6.**
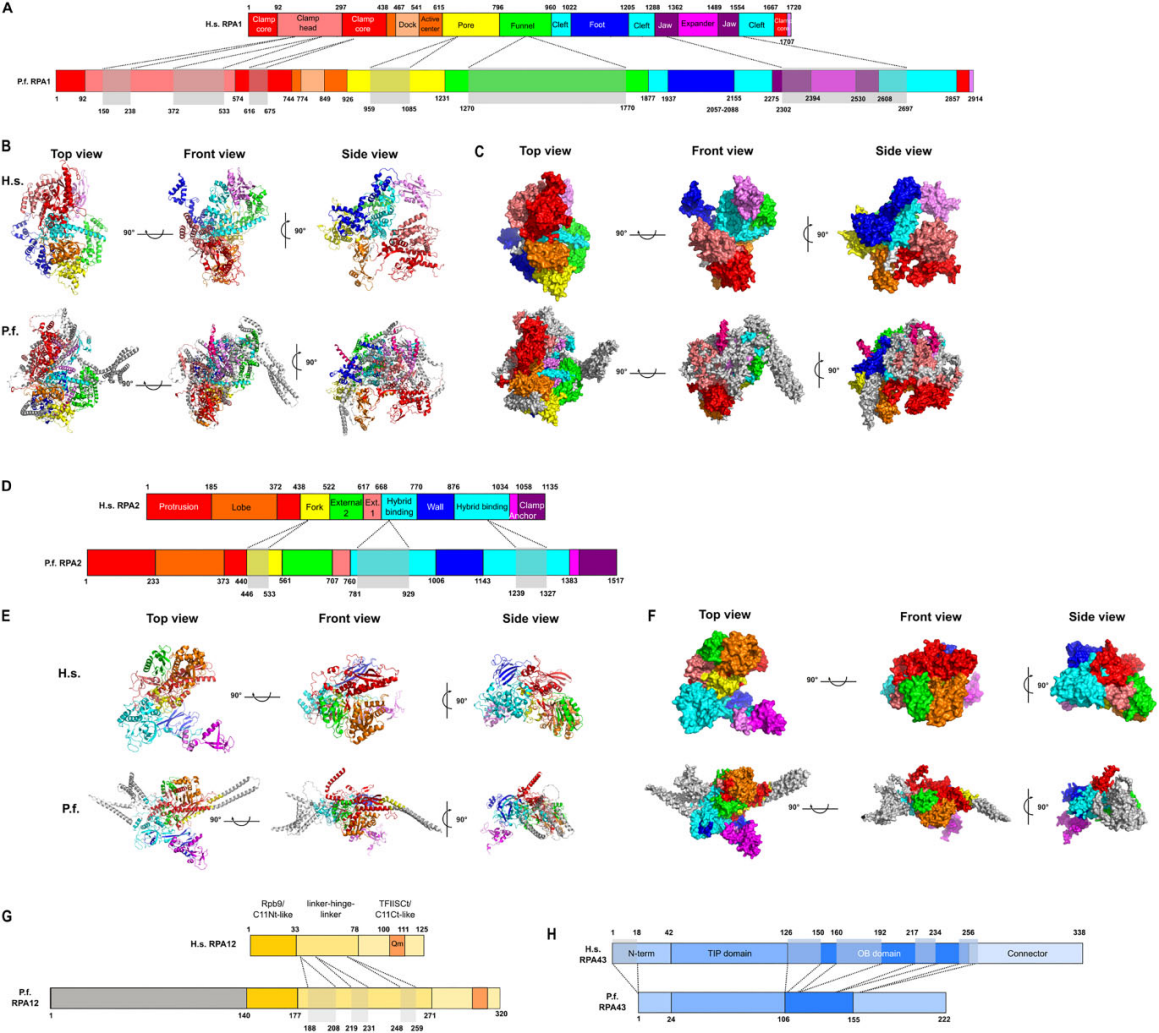
Comparison of the human (H.s.) and *P. falciparum* (P.f.) Pol I subunits with largest differences in size (RPA1, RPA2, RPA12, and RPA43). A, D, G, and H. Schematic outline of domain architecture of RPA1 (**A**), RPA2 (**D**), RPA12 (**G**), and RPA43 (**H**). The amino acid residue numbers at the domain boundaries are indicated. The largest amino acid insertions are marked by gray boxes. Ribbon diagram of RPA1 (**B**) and RPA2 (**E**). Domains are colored and color-coded as in panel (A) (RPA1) or as in panel (D) (RPA2). Inserts in P.f. subunits are in gray. A surface representation of RPA1 (**C**) and RPA2 (**F**). Domains are colored and color-coded as in panel (A) (RPA1) or as in panel (D) (RPA2). Inserts in P.f. subunits are in gray. Human subunits structure is from 7OB9; P.f. subunits are modeled in this work.

The largest Pol I subunit RPA1 of *P. falciparum* was already cloned in 1983 [66]; it substantially larger than its human ortholog (2914 versus 1720 amino acids), but the proteins share 29% identity. The most sizable insertions in *P. falciparum* RPA1 are present within the clamp head, pore, funnel and cleft domains (Fig. 6A and Supplementary Figs S2.1/S3.1 for alignment; see also reference [67] for a detailed domain structure).

RPA2 is also noticeably larger than its human counterpart (1517 versus 1173 amino acids) with a large extension within the Fork and the Hybrid binding domains (Fig. 6D and Supplementary Figs S2.2/S3.2 for alignment). The sequence is, however, 35% identical to human RPA2.

Another Pol I-specific “core” subunit, RPA12 (A12.2) (Fig. 5) plays a crucial role in rRNA cleavage during proofreading [68, 69] and is required to resolve deep backtracks [70]. Human RPA12 shares relatively low similarity to the *P. falciparum* protein (22%). The *P. falciparum* RPA12 ortholog is 194 amino acids longer than RPA12 from *H. sapiens* (Supplementary Table S2) due to a large N-terminal extension (Fig. 6G and Supplementary Figs S2.3/3.3 for alignment). *P. falciparum* RPA12 is 370 amino acid long and exhibits a conserved C-terminal TFSII-type zinc finger domain, similar to human and yeast RPA12. Although a second C4-type zinc finger falls below statistical significance, the protein remains distinctly recognizable as an RPA12-like protein through this conserved C-terminal region. Notably, within the conserved QXRSADEXXTXF motif, the two D and E residues play a critical role during proofreading. The high sequence and size conservation of the C-terminus is likely necessitated by the requirement of RPA12 to access the native RNA transcript and allow RNA cleavage during this control mechanism [71]. The N-terminal extensions vary widely in the queried species: from the 136 amino acids long *Tetrahymena* ortholog to the largest from *Vitrella brassicaformis* which is predicted to be 1432 amino acids long.

RPAC1 (AC40) and RPAC2 (AC19) are shared between Pol I and Pol III, while RPABC1 through to RPABC5 (ABC27-ABC10) (Fig. 5) presented in all three eukaryotic RNA polymerases. These shared polymerase subunits remain largely conserved with identities above 30% and reaching 55% for RPABC5 between human and *P. falciparum* (Supplementary Figs S2.4/S3.4 to S2.10/S3.10).

## Pol I unique subunits

The “core” Pol I is associated with additional Pol I-specific subunits that have no homologues in Pol II and Pol III. In yeast, the two subunits A49 and A34.5 form a heterodimer, assembling a Pol I-specific subcomplex [72], while two others (A43 and A14) form a so-called stalk (Fig. 5). The stalk in humans (and all other organisms except *Saccharomycotina*) is composed of a single protein, RPA43.

Subunit RPA49 is 419 amino acids long in humans (515 in yeast), and RPA34 is 510 amino acids long in humans (233 in yeast) (Supplementary Fig. S3.12 and S3.13). The domain architecture is maintained in yeast and human orthologs [73, 74], although the identities are no >22% and 24%, respectively, and human RPA34 contains a C-terminal extension. The RPA49 and RPA34 heterodimer is associated with the RPA2 lobe along with the N-terminus of RPA12 [69, 75–78] (Fig. 5). Although this heterodimer is not essential in yeast, it is required for efficient transcript elongation *in vivo* [79–81] and plays a critical role in transcription and cell proliferation in metazoans [74, 82–86]. This subcomplex is specific to Pol I but homologous to the Pol III C37/53 subcomplex [87, 88], and the Pol II initiation factors TFIIF and TFIIE [72] contribute to both transcription initiation and elongation [89, 90]. It facilitates the recruitment of the initiation factor RRN3 [78, 90] and supports its release during promoter escape [91–93].

In yeast, the N-terminus of RPA12 (amino acids 1–85) is needed to keep RPA49 associated with Pol I [71] and regulates Pol I loading onto rDNA [74]. The N-terminus of human RPA12 also interacts with the RPA49-RPA34 heterodimer at the lobe of Pol I, anchoring the protein and stabilizing its structure [68, 77, 94].

Surprisingly, the RPA49-RPA34 heterodimer was not identified in our sequence search in all the queried apicomplexan species except the ciliate *Tetrahymena thermophila*, where the sequence conservation with its human orthologs is low: 19% for RPA49 and 13% for RPA34 (Supplementary Figs. S3.13 and S3.13). Therefore, we speculate that the RPA49-RPA34 heterodimer has either diverged significantly in *P. falciparum* and other apicomplexans, making it unrecognizable, or it is absent and consequently not required for rRNA transcription in these organisms. The existence of divergent RPA49-RPA34 in *Tetrahymena* suggests that this heterodimer was present in the last common alveolate ancestor but may have been subsequently lost in the apicomplexan lineage.

The human and yeast RPA43 stalk subunits (338 and 326 amino acids in length, Supplementary Table S2) are characterized by a 123 and 125 amino acid long N-terminal SHS1_Rpb7-N domain (SHS1 domain found in the N terminus of Rpb7p/Rpc25p/MJ0397; PF03876); an RPA43 OB (oligonucleotide/oligosaccharide binding) domain in Pol I (PF187875) is found at the C-terminus. RPA43 but not RPA14 orthologs are encoded in the genomes of *P. falciparum* (Supplementary Fig. S2.11) and *P. berghei*, as well as *Toxoplasma gondii*, and they are assigned to the same ortholog group as those from yeast and humans (OG6_103 646) (Supplementary Fig. S3.11). Additional members from this study are divided between three additional ortholog groups. There is a substantial size variation in this set of proteins. The sizes of human and yeast RPA43 are 326 and 338 amino acids, while the *P. falciparum* protein is particularly short with 222 amino acids; the N-terminal SHS1_Rpb7-N domain is also small with 67 amino acids in length (Fig. 6H). Those from *Babesia microti* and *Theileria*, two related parasites from the sister group the *Piroplasmida*, are 229 and 237 amino acids in length, while the *T. gondii* member is 477 amino acids long (Supplementary Fig. S3.11).

## Essential Pol I-specific initiation factor RRN3

Pol I is unable to independently recognize and bind to the rDNA promoter and must be recruited to the TSS by accessory factors. RPA43, together with the specific helix α12a of subunit A190, provides the crucial attachment point for the essential initiation factor RRN3 [95], which bridges Pol I and the promoter-bound transcription factors. Importantly, only the Pol I-RNN3 complex can be recruited to the promoter-bound transcription factors and hence is vital for the PIC formation [78, 96–98]. In fact, a single point mutation in yeast RRN3 interface abolished interaction with Pol I and results in severe growth defects [99]. Despite the low sequence conservation between human and yeast RRN3 (21% identity) and different sets of promoter-bound transcription factors, the human RRN3 protein can functionally complement a yeast strain lacking the RRN3 gene [97].

RRN3 was identified in all queried organisms except the bird malaria parasite *H. tartakovskyi*, *P. falciparum*, and *P. berghei* (Fig. 4 and Supplementary Fig. S3.14 for alignments). These three organisms are members of the order *Haemosporidia*. However, it is found in the immediate sister group, the *Piroplasmida*, which includes organisms from the genus *Babesia* and *Theileria*. The absence of an RRN3-like transcription factor in other *Haemosporida* was confirmed through additional BlastP searches using human and yeast, as well as piroplasmid RRN3 sequences, along with all identified RRN3 proteins from this study at plasmodb. The lack of RRN3 in this lineage is a distinctive feature of this phylogenetic branch within the *Apicomplexa*. The absence can be attributed to either the loss of RRN3, with rRNA transcription not necessitating this bridging element, or the rapid evolution of the protein rendering it unrecognizable.

The OG6_152 531 RRN3 ortholog group encompasses 42 representatives of RRN3 family proteins from *Alveolata*. Part of this group is *Toxoplasma gondii* protein RRN3; it is annotated as an RRN3 protein in OrthoMCL and ToxoDB. With 3150 amino acids, the protein is substantially expanded compared to yeast and human RRN3. The 672 amino acids long Pfam RRN3 domain (6.4e-63) is located between positions 771 and 1442.

## Yeast promoter recognition transcription factors

In yeast, promoter-associated components of the PIC include the three-subunit core factor CF (comprising RRN11, RRN7, and RRN6), which forms a bi-lobal structure, engages and bends a proximal promoter element, and is both mechanistically distinct from other transcription systems and essential for Pol I initiation [100]. The seven-subunit upstream activating factor (UAF), together with TBP, is unique to unicellular fungi and includes RRN5, RRN9, RRN10, UAF30, and histones H3 and H4 and is, although not essential, required for promoter targeting and high-level transcription [101]. None of the RRN-annotated genes are present in the queried organisms, except H3 and H4, but a single protein related to UAF30 containing SWIB/MDM2 domains persists across all lineages (Supplementary Table S1).

## Human promoter recognition transcription factors

In mammals, rRNA transcription is directed by two promoters: a distant Spacer promoter and the 47S promoter positioned near TSS [102]. The 47S promoter (also known as a core promoter) is composed of the core element (covering the TSS), linker, and upstream control element (UCE). During transcription initiation, both the TSS-proximal core element and UCE are bound by Selectivity factor 1 (SL1) and upstream binding factor UBF. SL1 is a six-subunit complex composed of TATA-binding protein (TBP) and four TBP-associated factors: TAF1A (distantly related to yeast RRN11), TAF1B (yeast RRN7), TAF1C (yeast RRN6), and TAF1D [76, 96, 103, 104]. SL1 exhibits species-specific characteristics; this means that although its general architecture appears to be preserved across different species, e.g. mice and humans, they cannot substitute for each other and are incapable of initiating transcription in a species other than their own (hence the name) [105]. No components of this complex were found in the queried parasite organisms except TBP, either because they are absent or because they cannot be recognized (Supplementary Fig. S3.16).

The Six-HMG (high mobility group)-box upstream binding factor (UBF) is characterized by several DNA binding HMG-box domains, a C-terminal acidic region that interacts with SL1, and an N-terminal dimerization domain. The protein also connects with the RPA49-RPA34 heterodimer [74, 106]. The UBF homodimer brings together the Core and UCE promoter by wrapping DNA around itself, thereby altering the promoter's 3D-structure. This action stabilizes the PIC, and facilitates promoter escape, and thus activates Pol I transcription [106, 107]. UBF also binds within the transcribed region creating an open, nucleosome-depleted chromatin configuration along the entire RRNA gene [108–110]. Importantly, UBF is a key player in the formation of Nucleolar Organized Regiones (NORs), structures that are predecessor of the nucleolus [76, 102, 108, 110].

UBF-like proteins with multiple HMG-box domains are restricted to metazoans, and accordingly we failed to identify orthologs within the queried alveolates. Shorter proteins with one or two HMG-box domains are, however, encoded in the genomes of many species, including *S. cerevisiae* (Hmo1) and *Plasmodium*. In yeast, the role of UBF may be fulfilled by a combination of UAF and Hmo1, a significantly smaller protein with a single HMG-box [108, 109, 111–113]. Specifically, yeast Hmo1 is an important regulator of open, nucleosome-depleted regions in Pol I transcription [76, 111–116]. Both the *P. falciparum* and rodent malaria parasite genomes encode four proteins with HMG-box domains; they range in size from 98 to over 2000 amino acids in length. The roles of the two shorter proteins, HMGB1 and HMGB2, have been explored experimentally in some detail. High Mobility Group Box 1 and HMGB2 localize mostly to the nucleus, which has been established independently by IFA assay and protein tagging [117–119]; the subcellular localizations of HMGB3 and HMGB4 are not known yet. HMGB1 is likely essential for *P. berghei* development in the infected mouse model [120]. Rodent Δ*hmgb2* parasites are attenuated during asexual blood stage development [120–122], and the *P. berghei* NK65 strain knockout mutant grows poorly in the mouse host and is ultimately cleared by the immune system after a 2-week-long infection [120, 121].

The *P. falciparum* Δ*hmgb2* parasite, on the other hand, grows normally during the asexual blood stage in cell culture but experiences a defect during mosquito infection instead [123], a defect also found in *P. yoelii* with a reduction in ookinete, oocyst, and sporozoite numbers [122].

**Figure 7.**
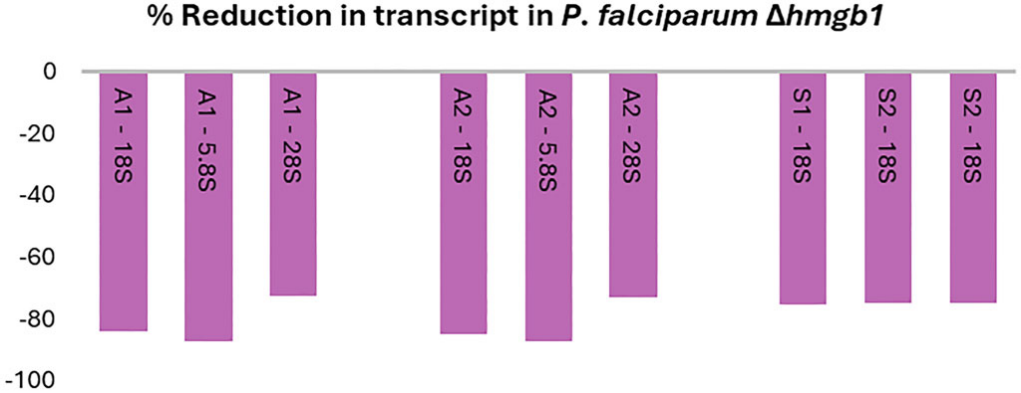
rRNA response in *hmgb1* deletion mutants. FPMK values normalized for wild-type rRNA values. Data from Lu *et al.* [118].

HMGB1 is most highly expressed in *P. falciparum* ring stage parasites, the life cycle stage form that develops from the RBC-infective merozoite and undergoes further cell growth followed by DNA replication and mitosis to produce 16–32 daughter cells. While HMGB1 was reported to occupy centromeres, its absence also negatively impacts the transcription levels of a small subset of genes [118]. Among the 87 downregulated transcripts, the 18S, 5.8S, and 28S rRNAs of both A-type loci show a >70% reduction in expression levels (Fig. 7). Notably, all three 18S rRNA variants from the singular S1 and the dual S1 loci were equally reduced; the effect on the 5.8S and 28S rRNAs was not listed. The expression levels specified by FPKM values (fragments per kilobase of transcript per million mapped reads) for the two S types 18S transcripts were, however, ∼40 times lower than those reported for the A-type genes. This transcriptome analysis strongly suggests that HMGB1 plays a crucial role in the efficient transcription of A-type rRNA genes during the asexual blood stage cycle. However, direct evidence linking HMGB1 to the rDNA *loci* was not obtained in the associated chromatin immunoprecipitation experiment [118]. *In vitro* studies have demonstrated that *P. falciparum* HMGB1 and HMGB2 interact with cruciform (4H) DNA structures [119]; both proteins localize to a subregion within the nucleus but there is no evidence whether they directly bind rDNA *loci* in the parasite or not.

### Structure and potential roles of parasite-specific Pol I subunit segments

To get insight into the potential roles of parasite-specific regions in Pol I subunits, we ran structure predictions using AlphaFold 3 (AF3) [124] of parts of *P. falciparum*, *Tetrahymena thermophila*, and *Toxoplasma gondii* Pol I based on the homologous subunits we identified. Due to maximal residue number limitations of the current software, it is not possible to predict the entire enzyme complexes. Instead, we overlaid three partial models using subunits (RPA1 and RPA2), which we placed in all partial predictions for each organism to generate a comprehensive, complete Pol I model. The resulting top 5 ranking structure predictions were manually compared to determine the consistency of predicted models. To assess whether predicted structures of parasite-specific regions are known from other proteins or transcription systems, these segments were subjected to structure-based homology searches using PDBeFold and DALI servers. Independently, we performed sequence-dependent domain predictions for the main insertions using “HH-Pred” for independent comparison. Finally, the results were compared with each other and among organisms (see Supplementary methods for more details).

Figure 6B, C, E, and F compares the human and *P. falciparum* RPA1 and RPA2 subunits, with parasite-specific segments highlighted in gray and domains depicted in various colors. Ribbon diagrams and surface representations of the models for both parasite subunits clearly demonstrate that the three-dimensional domain structure of these subunits is largely conserved. Parasite-specific segments are primarily located around the core, potentially forming new interfaces for interactions with other molecules.

**Figure 8.**
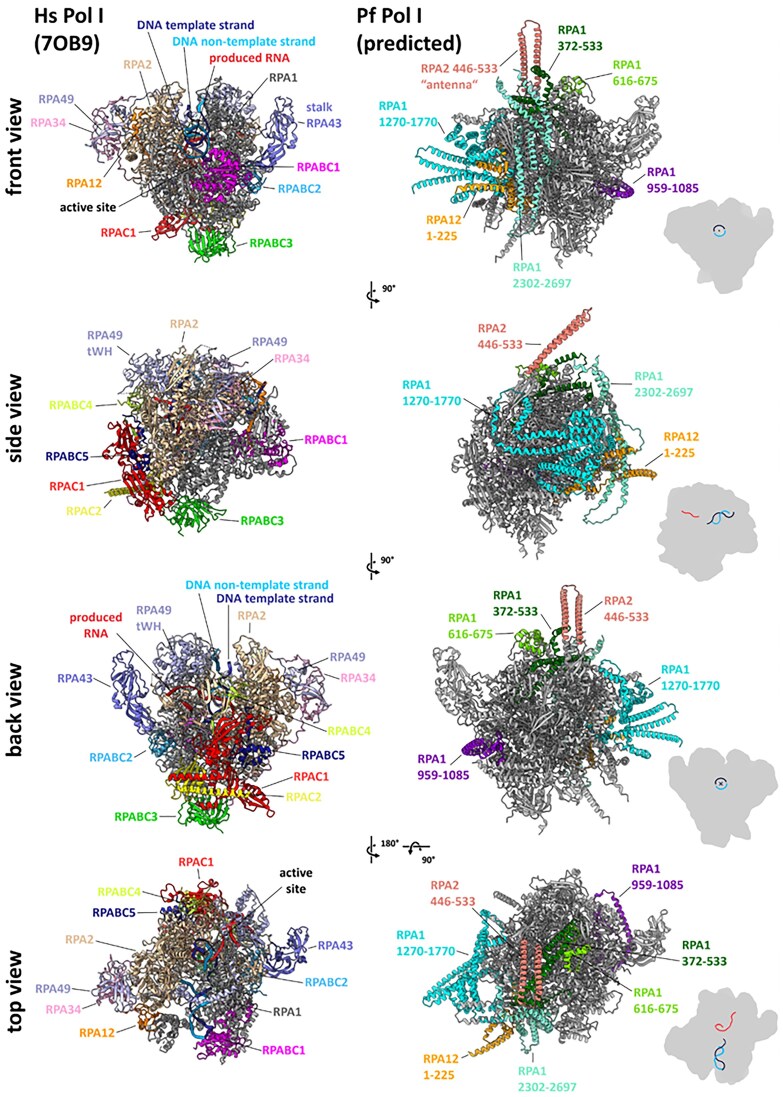
Comparison of the predicted *P. falciparum* Pol I structure with its human counterpart. Left: Structure of elongating human Pol I (PDB 7OB9) shown in front, side (core module), back, and top views. Subunits are colored as follows: RPA1 (gray), RPA2 (wheat), RPAC1 (red), RPAC19 (yellow), RPABC1 (magenta), RPABC2 (hafnium), RPABC3 (green), RPABC4 (lemon), RPABC5 (density), RPA43 (slate), RPA12 (orange), RPA49 (light blue), and RPA34 (pink). Right: Structure predictions of *P. falciparum* Pol I presented in the same orientations. The composite model was constructed from predictions using different subunit combinations (see Methods). Conserved regions of *P. falciparum* Pol I are shown in gray, while large insertions relative to the human enzyme are highlighted in different colors: RPA1 (372–533: dark green, 616–675: green, 959–1085: dark violet, 1270–1770: cyan, 2302–2697: aquamarine blue), RPA2 (446–533: salmon), and RPA12 (1–225: orange). Bottom right of each view: Schematic overviews outline the orientations relative to the template. The Pol I core is shown in gray, the non-template DNA strand in medium blue, the template DNA strand in dark blue, and the synthesized RNA in red.

A composite model of *P. falciparum* Pol I that has the highest confidence score is displayed in Fig. 8. Inspection of the structural models clearly showed that parasite Pol I adapts the characteristic shape of a multi-subunit polymerase [125] with a high-domain homology to its yeast and human counterparts.

Most noticeably, subunit RPA2 contains an insertion in the “protrusion” domain (*P. falciparum* YFE^446^-ELY^533^). This insertion is predicted to form an elongated, coiled-coil-like motif with high confidence across all AF3 outputs and has therefore been termed the “antenna” due to its distinctive shape. To our knowledge, the antenna domain is unique to Pol I from the genus *Plasmodium* and is absent in Pol I of other apicomplexan parasites. A distant homology to a similar feature in the tuberculosis RNAP beta-prime subunit was detected in 3D-fold searches (DALI); however, the function of this structural element remains unclear. It can be speculated, though, that the “antenna” may facilitate parasite-specific transcriptional regulation, potentially interacting with other parasite-specific factors to adapt rRNA synthesis to the parasite’s unique life cycle and environmental conditions.

A very prominent insertion of the funnel domain within subunit RPA1 (1270–1770) is inconsistently predicted by AF3. All highest-scoring output models are of low confidence in our view. Its location is predicted directly between the two “funnel” helices that are conserved in structure among eukaryotic Pols. This funnel insertion is found in those parasites that lack homologues of RPA49/34 subunits (Fig. 4 and Supplementary Table S1). The location of the insertion is in proximity to the position that is occupied by the “dimerization” domain of RPA49/RPA34 (Fig. 5), which anchors this sub-complex to the polymerase core. This insertion may partially substitute the RPA49/RPA34 complex.

Similar to the RPA1 funnel insertion, an insertion at the N-terminus of RPA12 (Figs 4, 6 and Supplementary Table S1) is detected but its structure is inconsistently predicted. The highest-scoring models diverge among themselves and are thus considered low confidence. Nevertheless, similar to the RPA1 funnel insertion, this RPA12 N-terminal insertion is not found in parasites that lack RPA49/RPA34, again indicating a possible replacement of the heterodimer functions. The location of the RPA12 N-terminus is predicted to be in close proximity to the position that is occupied by the ‘dimerization’ domain of RPA49/34, supporting a speculation about functional compensation. In fact, in yeast the A12 N-terminus physically interacts with the dimerization domain [64, 65]. Furthermore, a 3D-fold search of the highest-scoring structure prediction of the RPA12 N-terminal insertion (PDBeFOLD) indicates some structural correlation to the yeast RPA49 tandem-winged helix domain, and a domain homology search using HH-pred indicates similarity of the *P. falciparum* insertion with the Pol II general initiation factor TFIIE. Note that TFIIE is structurally and functionally similar to the RPA49 [72], although the TFIIE subunit showing similarity to RPA49 is TFIIE-beta, and HH-pred generated domain similarities are found with TFIIE-subunit alpha. Also, note that the positions of the “super Pol” mutations leading to high level of processivity [126] are close to the positions of both insertions but not identically positioned on the RPA2 lobe.

Hence, there might be compensation for the roles that RPA49/RPA34 usually carry out by a combination of the insertions in the RPA1 funnel and the RPA12-N-terminus, but evidence remains circumstantial. Also note that the C-terminal region of subunit RPA12, which is structurally and functionally similar to domain III of the Pol II elongation/cleavage factor TFIIS, is again conserved among parasite predictions and yeast/human structures.

Regarding the parasite Pol I “stalk” sub-complex, it becomes clear that the predicted fold of an insertion in *P. falciparum* subunit RPA43 resembles the structure of the second yeast stalk-subunit RPA14, which is absent in parasites (and metazoans) [76, 127]. This indicates possible compensation for the loss of this subunit by elements inserted into the stalk-subunit RPA43 in parasites. Such compensation might be required in the absence of RPA49/34 homologues or have a role independent of the RPA49/34 sub-complex in parasites, for example affecting Pol I processivity and therefore compensating for the reduced number of rRNA genes.

The *P. falciparum* RPA1 coiled-coil part of the clamp core domain carries a noticeable insertion (P.f. 618–676) (Fig. 4, 6 and Supplementary Table S1). This part is distinct, meaning structurally extended, in Pol I compared to other Pols [64]. This coiled-coil is important in initiation and elongation and contracts during DNA melting and remains contracted throughout elongation [128]. The domain also interacts with the RPA49 linker and tWH domains in Pol I [129] and with the elongation factor Spt5 in Pol II [130]. *P. falciparum* modeling results of this clamp-core insertion are consistent among top-ranking models. A functional correlation with the RPA2 antenna domain in the absence of the RPA49 linker/tWH domain is possible.

Interestingly, the predicted Pol I-specific ‘dock’ domain of subunit RPA1 is structurally conserved. This domain plays a crucial role in interactions with key initiation factors, such as RRN3 [95, 131] and the N-terminal zinc ribbon domain of Rrn7 or TAF1B (subunits of Core Factor or SL1, respectively) [100]. This implies that parasite Pol I retains the capacity to interact with homologues of these factors. However, this is confusing considering the apparent absence of these factors in many parasite species.

Several other insertions, for example in the RPA1 clamp head, are inconsistently predicted with low confidence. This indicates that they have no function conserved among parasite species or that they adapt unknown folds, which makes structure prediction hard to impossible even with the tools available to date.

## Conclusions

Here we provide compelling evidence for the conserved nature of the Pol I enzyme in *P. falciparum* and many related alveolate organisms. These protists retain an ancestral enzymatic Pol I core, whose components have, for the most part, diverged little and remain highly conserved with those from *H. sapiens* or yeast. Predictably, all identified *P. falciparum* Pol I enzymatic factors resist genetic engineering by piggyBac DNA transposon insertion, and these observations are consistent with data obtained from a *P. berghei* gene deletion screen and a genome-wide CRISPR study in the Toxoplasma GT1 strain [54, 132, 133].

Our work reveals the shared, divergent, and absent components essential for efficient ribosomal DNA transcription in alveolates through detailed sequence similarity analysis. Leveraging the latest advances in multi-protein-complex structure modeling, we explored the structural features of *P. falciparum* Pol I compared to its human counterpart, uncovering several striking structural differences that may be key to the parasite’s unique transcriptional machinery. Notably, we identified several additional domains (Fig. 8) within the two main catalytic subunits, RPA1 and RPA2, which appear unique to *P. falciparum* and closely related parasites within the genus *Plasmodium*, *P. berghei* for example. These domains may substitute for loss of certain specific Pol I subunits (Fig. 4) and could impact the processivity of *P. falciparum* Pol I. Even more intriguing, our analysis suggests that *P. falciparum* Pol I can still interact with promoter recognition factors present in humans and yeast despite the apparent absence of recognizable homologs in *P. falciparum* itself (Fig. 4).

Another intriguing finding is the absence of any identifiable homologue of the essential transcription factor RRN3 in the *Haemosporida* lineage, which includes *P. falciparum*, *P. berghei*, and *H. tartakovskyi*. Critically, RRN3 is present in the genomes of closely related species within the immediate sister group, the *Piroplasmida*, as well as in all other queried alveolates, where it displays a wide range of protein sizes. This suggests that *Haemosporida* lineage may develop a mechanism of transcription initiation that does not require RRN3.

Notably, the predicted molecular weight of the ten-subunit core of *P. falciparum* is considerably larger than its human counterpart: 678.34 kDa versus 472.86 kDa (computed from the accession numbers in Supplementary Table S1), despite lacking RPA49/RPA34 and RRN3, which suggests a functional replacement of these proteins by additional Pol I domains.

One of the most fascinating and unexpected outcomes of this study was the complete absence of recognizable homologues to essential transcription factors from human and yeast systems in the queried alveolate lineages. The missing components include all specific SL1 subunits, UBF, and all components related to the yeast core factor and UAF. This raises fundamental questions about transcription initiation in these parasites, especially given that eukaryotic enzymes cannot bind directly to the rRNA promoter. It should be noted that to date, only two ways of organizing Pol I transcription machinery are known: the multicellular organism model (e.g. mammals) and the unicellular organism model (e.g. yeast). The apparent absence of canonical factors allying to any of two models points to fascinating possibilities: these organisms may have evolved novel, yet-to-be-discovered mechanisms for recruiting Pol I to the rDNA promoters across the complex life cycle or exhibit evolutionary divergence from a common ancestral gene that has rendered these factors unrecognizable in these organisms. Most excitingly, this fundamental difference between *Plasmodium* and human transcription initiation machinery opens up compelling new opportunities for therapeutic intervention.

In the absence of UBF, we propose that *P. falciparum* HMG-box proteins play a crucial role in creating a nucleosome-depleted region that supports efficient rRNA transcription and perhaps rRNA promoter recognition. The HMGB1 knockout in *P. falciparum* experiences a significant reduction in rRNA transcript levels, including 18S, 5.8S, and 28S, particularly in asexual stages [118] (Fig. 7). This protein is predominantly expressed in the ring stage, whereas its paralog HMGB2 is mainly detected in sexual stage parasites (gametocytes). In the related rodent model *P. yoelii*, mutants lacking *hmgb2* produce 50% fewer ookinetes and 90% fewer oocysts, leading to a notable reduction in sporozoite formation in the mosquito host [122]. Particularly the female gametocyte is known to store substantial amounts of translationally repressed mRNA, which is used to instruct ookinete formation in the mosquito. FISH experiments have shown that the female is also provided with an rRNA [134]. Possibly HMGB2 plays a role in the transcription of S-type rRNA genes in the female gametocyte, which are required for the formation of its the ookinete and later in the oocyst and sporozoite.

Our analysis raises several questions which must be addressed experimentally:

- How is Pol I transcription initiated in the absence of canonical transcription factors?
- How are A- and S-type rRNA transcription levels controlled across different parasite life cycle stages?
- What are the functional roles of the extensions and insertions in *P. falciparum* Pol I subunits?
- Can differences between *Plasmodium* and human Pol I machinery be exploited for therapeutic purposes?

An experimental starting point may be the use of proximity labeling techniques that have allowed the identification of divergent protein components, the nuclear pore complex for example.

## Supplementary Material

gkaf641_Supplemental_Files

## Data Availability

Data available on request.
