# Supplementary material for: The ribosomal RNA transcription landscapes of Plasmodium falciparum and related apicomplexan parasites
Source: Nucleic Acids Res. 2025 Jul 8;53(13):gkaf641. doi: 10.1093/nar/gkaf641 (PMC12235521; doi:10.1093/nar/gkaf641)
Supplement: gkaf641_Supplemental_Files [file gkaf641_supplemental_files.zip › Supp_data.pdf]

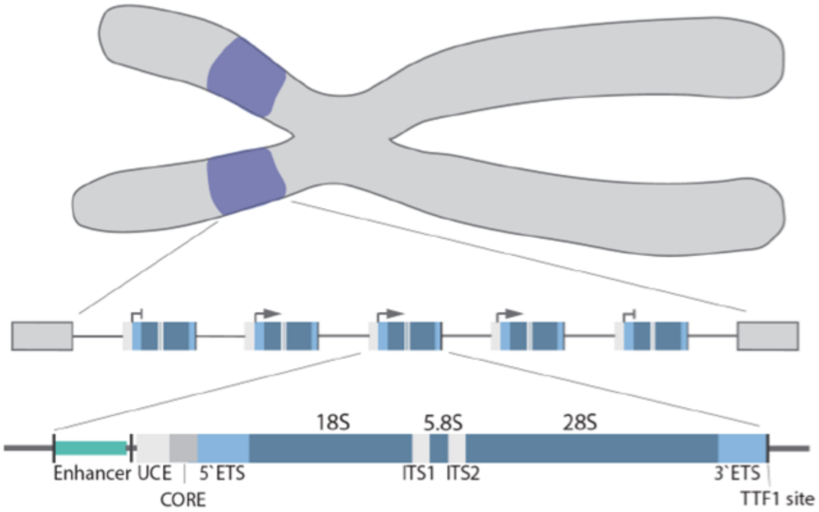

Figure S1. rDNA gene arrays (purple) are located on the short arms of the human acrocentric chromosomes. Organization of a single rDNA gene: enhancer, upstream control element (UCE), core promoter (CORE), 50/30 external transcribed spacer (ETS), 18S, 5.8S, 28S, internal transcribed spacer (ITS1/2), and transcription terminator factor 1 (TTF-1) site. Taken from Panov at al. [34].

Figure S2. Pairwise protein alignments; *Homo sapiens* and *P. falciparum*

https://www.ebi.ac.uk/jdispatcher/msa/clustalo
https://www.bioinformatics.org/sms2/color_align_cons.html

Species abbreviations used in the following alignment pages:

hsap	Homo sapiens
pfal	Plasmodium falciparum 3D7

Figure S2.1. RPA1

```
hsap|ENSG00000068654 MLISKNMPWRRLQGISFGMYSAEELKKLSVKSITNPRYLDSLGNPS---ANGLYDLALGPADSKEVCSTCVQDFSNCSGH 77
pfal|PF3D7_0509400   MYDINKSICAEIKSAELDVLNSGELRRISMGKYENEKYEYEKTHKSGVVTKMYYDPKYGTIDHNQICSVCFERHENCTGH 80

hsap|ENSG00000068654 LGHIELPLTVYNPLLFDKLYLLLRGSCLNCHMLTCPRAVIHLLLCQLRVLEVGALQAVYELERILNRFLE---------- 147
pfal|PF3D7_0509400   IGHIEFVLPLFNPLFYKELQELLNLICYHCYNFCCSEDVIFLLKHIFQLKSLNQIESTNKIKYKKNCDYEYIIDQVEKLY 160

hsap|ENSG00000068654 -------------------------------------------------------------------------------- 147
pfal|PF3D7_0509400   KKICKDSNISLDDIVEAIYDDYENVHEDKDGNFDESVDLLDSLEKIKDEPDEKNKNNNNKNNNNNNNNNNNYNNDVDID 240

hsap|ENSG00000068654 ---------------------------------------ENPDPSASEIREELEQYTTEIVQNNLLGSQGAHVKNVCESK 188
pfal|PF3D7_0509400   VDNNNNNVDINVDNNNNYFNSDLVDLAPNEEIYEEETDAGRSDDNNKENKPDYNKLKERIKKYKFDANKLAFQSNYNYEE 320

hsap|ENSG00000068654 SKLIALFWKAHMN-AKRCPHCKTGRSVVRKEHNSKLTITFPAMVHR---------------------------------- 233
pfal|PF3D7_0509400   YLILSKTIKLHMKKSSKCCFCKFQRNISAKVSQRRDTINFIGVHTNNPFFKKYMPKKKQKKNEKDDQEDVDDLENMNNID 400

hsap|ENSG00000068654 ------------TAGQKDSEPLGIEEAQIGKRGYLTPTSAREHLSALWKNEGFFLNYLFSGMDDDGMES----------- 290
pfal|PF3D7_0509400   DINYNDDVDNMQHMNDDESQLNSIQSRNENNKLMIKRESKKIEYNNNVNDESIKIEEDNNEDDDKQMDRESSYPHNNNNN 480

hsap|ENSG00000068654 -------------------------------------------------------------------------------- 290
pfal|PF3D7_0509400   NNDNNENYNKLSGGYENSFNEISTDNIRNDKNKNSTFADMVNHYNRTQAPKEKCTIRLFSFQVIDILKKIFENNNKDIIN 560

hsap|ENSG00000068654 -------RFNPSVFFLDFLVVPPSRYRPVSRLGDQMFTNGQTVN---------------------LQAVMKDVVLIRKLL 342
pfal|PF3D7_0509400   LLYPFTKRDGYKKFFLYDMGVSGNRFRSQSKGIHVRTKITNTLCRFNNFIKLCINAKKKVDFDYLLEHNKKDLKLYKDMF 640

hsap|ENSG00000068654 ALMAQEQKLPEEVATPTTDEEKDSLIAIDRSFLSTLPGQSLIDKLYNIWIRLQSHVNIVFDSEMDKLMMDKYPGIRQILE 422
pfal|PF3D7_0509400   SVFSLKMKYKFNNNIMDDDTDITKMDFLKYYTLVYNNKSAYINILFSTLQLGVNTFYDSTLTEKVNNKHLKNISIRQILD 720

hsap|ENSG00000068654 KKEGLFRKHMMGKRVDYAARSVICPDMYINTNEIGIPMVFATKLTYPQPVTPWNVQELRQAVINGPNVHPGASMVINEDG 502
pfal|PF3D7_0509400   KKEGILRKNIMGKRVNNCARTVISPDTFIETNQIGMPIEFAKVLTIDEYITQNNFTYIKKLIENGPNIYPGALSYRDSNG 800

hsap|ENSG00000068654 SRTALSAVDMTQREAVAKQLLTPATGAPKPQGTKIVCRHVKNGDILLLNRQPTLHRPSIQAHRARILPEEKVLRLHYANC 582
pfal|PF3D7_0509400   RVFKLSHKYEDRLKVIEKIEKLNFQTEN----SYILHRHARDGDVVIMNRQPTLHKFSIMAHYLKIFEKEKVFRLNYVNC 876

hsap|ENSG00000068654 KAYNADFDGDEMNAHFPQSELGRAEAYVLACTDQQYLVPKDGQPLAGLIQDHMVSGASMTTRGCFFTREHYMELVYRGLT 662
pfal|PF3D7_0509400   SSYNADFDGDEMNLHLLQTPLARAEATHLMNCDFLFTSFKDGSPLRGLAQDFILGGLHLTSLETFLNYDEYCNLLQCSLN 956

hsap|ENSG00000068654 DKVG---------------------------------------------------------------------------- 666
pfal|PF3D7_0509400   SLISQKNSFFIKKKNNNNNNKKNTNRTNSIMKNTRNSQYNIIQNDLNNNDSKEDYLKKMSILKNQQSLDDNAKISHPVS 1036

hsap|ENSG00000068654 -----------------------------------------------------------RVKLLSPSILKPFPLWTGKQV 687
pfal|PF3D7_0509400   NDEARNNSIYEPTNNLLKPQDIDILKEINHGEQFNGNVLKITNYNYLDPYAIILNRTSFTIITEEPAILYPKKLWTGKQL 1116

hsap|ENSG00000068654 VSTLLINIIPEDHIPLN-----LSGKAKITGKAWVKETPRSVPGFNPDSMCESQVIIREGELLCGVLDKAHYGSSAYGLV 762
pfal|PF3D7_0509400   ITSILKTVIDKVAIETYNKYNDNEFMNTYKGINYVAKAKTSPDLWSFDPLKENEVIIKNSELLQGVLDKLHFGASSNSLV 1196

hsap|ENSG00000068654 HCCYEIYGGETSGKVLTCLARLFTAYLQLYRGFTLGVEDIILVKPKADVK------------------------------ 811
pfal|PF3D7_0509400   HLCHELFGPKTSAVMLDCFGRLFINFLQLRGTSLSLYDFILNKNAKKEKSLIKKRISFTGFYLQNLFAYSIANSVNADIT 1276

hsap|ENSG00000068654 -------------------------------------------------------------------------------- 811
pfal|PF3D7_0509400   EMNSYTKKKMNTGNQRKKLILNKMYDLYEKQNELINNNFNEKENYLNKMEKRNIKKDSHVMKDEHILSISENSYLLALLN 1356

hsap|ENSG00000068654 -------------------------------------------------------------------------------- 811
pfal|PF3D7_0509400   KELQNVIYHILNEVQFCDDDASLLNKEKNEKENEKENKIANTCINYKHNNSHVDDEQNCNSSLYSCVQYVLNKDLFNLYE 1436

hsap|ENSG00000068654 -------------------------------------------------------------------------------- 811
pfal|PF3D7_0509400   KYLNGKNEEANKHILYNNCQIDERNNHFLNSVTNKLYKALKEPTFFNSTSFTIKKITKVIEKVLTFLKAARCNKRLKIYI 1516

hsap|ENSG00000068654 -------------------------------------------------------------------------------- 811
pfal|PF3D7_0509400   IFELFQNKNIMKYFPSLVNYINSFHFDVSNEEIIKNVINNIYFKEEDESPISSINNNNNNKVEDDCNQDIKIKGDQETNE 1596

hsap|ENSG00000068654 ---------------RQRIIEESTHCGPQAVRAALNLPEAASYDEVRGKWQDAHLGKDQRD------------------- 857
pfal|PF3D7_0509400   FIDDEKIKTKRKCSQEKTIQEIKKYKLKKFLHFYAELHKKCNYEEGRGYDIDEQNDYNIDEKDFMNDYNDNNNNNNDNLF 1676

hsap|ENSG00000068654 -------------------------------------------------------------------------------- 857
pfal|PF3D7_0509400   RRSFVKNEMMSVNHTSDDVYDEKIIRNCLMSSLPSFTLNDEITNLSYNGKYQYYEDYLCSKKKDEEDLNFKYYNMDDVFY 1756

hsap|ENSG00000068654 -----------------FNMIDLKFKEEVNHYSNEINKACMPFGLHRQFPENSLQMMVQSGAKGSTVNTMQISCLLGQIE 920
pfal|PF3D7_0509400   KMEYLIHNYFYLKRSDFDNLIDSLFQPYLCRVSSGTNNLINMNNLMNKFLNNGFSNMIFTGAKGSKVNYSMICGMLDQQY 1836

hsap|ENSG00000068654 LEGRRPPLMASGKSLPCFEPYEFTPRAGGFVTGRFLTGIKPPEFFFHCMAGREGLVDTAVKTSRSGYLQRCIIKHLEGLV 1000
pfal|PF3D7_0509400   LEGKRVPRMRSGKTLPSFHRYDYGARSCGLITDCFLEGLRPQEYFFHCMSGREGLIDTAVKTAKSGYIQRCLIKCMESVI 1916

hsap|ENSG00000068654 VQYDLTVRDSDGSVVQFLYGEDGLDIPKTQFLQPKQFPFLASNYEVIMKSQHLHEVLSRADPKKALHHFRAIKKWQSKHP 1080
pfal|PF3D7_0509400   LHYDGTVRNEDNSIIQFLYGEDGIDPSKTAYLDLS--KDLLHNYHLSFSKYLNNNMHSKILNNQELFLKHEKDNDTKDNE 1994

hsap|ENSG00000068654 NTLLRRGAFLSYSQKIQEAVKALKLESENRNGRSPGTQEMLRMWYELDEESRRKYQKKAAACP----------------- 1143
pfal|PF3D7_0509400   EPLISKYNPYTYIGSVSDKFNNKLNNTLLWNDLNFPEYENVPMNFDTLSSSLLKSKYYNSLCNPGESIGILVAQSFGEPA 2074

hsap|ENSG00000068654 --------------DPSLSVWRPDIYFASVSETFETKVDDYSQEWAAQTEKSYEKSE-----LSLDRLRTLLQLKWQRSL 1204
pfal|PF3D7_0509400   TQMTLNTFHLAGTENVTMGIPRLKEIFLTSKLTSKPMIYVPIKLQVDNLKNDANKMKNYITSIADKILSSYKSIFLTDVI 2154

hsap|ENSG00000068654 CEPGEAVGLLAAQSIGEPSTQMTLNTFHFAGRGEMNVTLGIPRLR----------------------------------- 1249
pfal|PF3D7_0509400   YGIGVDRKIILKENQNEQKHTINLSDIINMNDEELSGDTQLAKINNVLKNCNINFDMKKEWNYEIIIQFENLYHFCNINK 2234

hsap|ENSG00000068654 ------EILMVASANIKTPMMSVPVLNTKKALKRVKSLKKQLTRVCLGEVLQKIDVQESFCMEEKQNKFQVYQLRFQFLP 1323
pfal|PF3D7_0509400   HLTISFILYKVVNALLNSVLNKVHETFVFHNLINMNSLNHEYYDELYDFISKKMSEEFNFNMDKYEYKDDEHQINAKLKF 2314

hsap|ENSG00000068654 HAYYQQ-----------------------------------------EKCLRPEDILRFMETRFFKLLMESIKKKNNKAS 1362
pfal|PF3D7_0509400   MEKENERYDSKRGTNNNISGEYQNASHTIASHMEDDSPMKSYNDISNKENMSPYDDGKGKDGDRDQDDMSDIELKDNSEK 2394

hsap|ENSG00000068654 AFRNVNTRRATQRDLDNAGELGRSRGEQEGDEEEEGHIVDAEAEE------------GDADASDAKRKEKQEEEVDYESE 1430
pfal|PF3D7_0509400   NSNSDQASKNADSDTDEEEEDDELKSEKEKEEDEKEYDEEGESDTNSKIDSDHKSDSDNKSDSDNKSDSDHKSDSDHKSD 2474

hsap|ENSG00000068654 EEEEREGEENDDEDMQEERNPHREGARKTQEQDEEVGLGTEEDPSLPALLTQPRKPTHSQEPQGPEAMERRVQAVREIHP 1510
pfal|PF3D7_0509400   SDHKSDSDHKSDSDNKNDSDNKNDSDIDSDSESEMVSSNKKEIININNKDNYYTNRNSSNESKLSECSNTKGSTVNYFSS 2554

hsap|ENSG00000068654 FIDDYQYDTEESLWCQVTVKLPLMKIN-----FDMSSLVVSLAHGAVIYATKGITRCLLNETTNN--------------- 1570
pfal|PF3D7_0509400   ETSATHHILVDEEESGIFPNLKNKKLNNMNDNKNMSSTTVSSSLSSSSLSTPLNKKNIEDDEHINNIHFIEKNKYNEEMD 2634

hsap|ENSG00000068654 -------------------------------------------------------------------------------- 1570
pfal|PF3D7_0509400   NKEEFITKSFTRLSYDYKNNINLISSDDDQENIEENEEGQKMKKSEQRYVKNIIDKIKSSLLCFIKNIHFSPVTWILKLN 2714

hsap|ENSG00000068654 --------------------KNEKELVLNTEGINLPELFK--------------------------YAEVLDLRRLYSND 1604
pfal|PF3D7_0509400   IGWDINFFPYPIDFLNHIKSEINKEVLFKVKDLNNPKILKGKDITHSGAEYELQIEGKNIYKLYNIKDKYIDKTKLYCND 2794

hsap|ENSG00000068654 IHAIANTYGIEAALRVIEKEIKDVFAVYGIAVDPRHLSLVADYMCFEGVYKPLNRFGIRSNSSPLQQMTFETSFQFLKQA 1684
pfal|PF3D7_0509400   IYAIVKTYGIEAGRLCITKELKKVFDAYGIQIDFRHLSFISDFMTHTGDLKAFNRHGLGYFRNVFHKMSFECATNFLIQG 2874

hsap|ENSG00000068654 TMLGSHDELRSPSACLVVGKVVRGGTGLFELKQPLR---- 1720
pfal|PF3D7_0509400   CIHNSIDYLSTASSSLFFGKHIKVGTNLADIVTCIDKQNL 2914
```

Figure S2.2. RPA2

```
hsap|ENSG00000125630 MRAKEAAETGLRPLLPACTERLASGVYRETGVRCAGGHMDPGSRWRNLPSGPSLKHLTDPSYGIPREQQKAALQELTRAH 80
pfal|PF3D7_1134700   -MAEEVNRNIKRTSELINNNKVDKSFLKNINLQITDGEIKLHLKIIEALYP----NMKLKVDKLINGSYNIFTKFLVQSH 75

hsap|ENSG00000125630 VESFNYAVHEGLGLAVQAIPPFEFAFKD-------------ERISFTILDAVISPPTVPKGTICKEANVYPAECRGRRST 147
pfal|PF3D7_1134700   IDDFNSFINVYIKNIAENIPIMEFSPQQNNYSLLNMNKNNSESVKFFVSDIQIKNPMIKN-DKGEYRADYPYLCKLSART 154

hsap|ENSG00000125630 YRGKLTADINWAVNGISKGIIKQFLGYVPIMVKSKLCNLRNLPPQALIEHHEEAEEMGGYFIINGIEKVIRMLIMPRRNF 227
pfal|PF3D7_1134700   YEGELLIKINRQYK-DEIASTTICAGHIPIMIMSNLCNLSNLNKKELAQKGEDQSLLGGFFVVSGRLKVIRYVIHPKYNT 233

hsap|ENSG00000125630 PIAMIRPKWKTRGPGYTQYGVSMHCVREEHSAVNMNLHYLENGTVMLNFIYRKELFFLPLGFALKALVSFS-DYQIFQEL 306
pfal|PF3D7_1134700   LLLNADNKIHIN------------CLLNDNTVVINFLTLTRNNSYVYGFRFQNAVCSLPFHLLLMILSPIKKKSYIFNKI 301

hsap|ENSG00000125630 IKGKEDDSFLR----NSVSQMLRIVMEEGCSTQKQVLNYLGECFRVKLNVPDWYP--NEQAAEFLFNQCICIHLKSNTEK 380
pfal|PF3D7_1134700   KLGVENENAIKYIELFINSIFLKDTFNEKELFEKYNLSYLGRIAYLRRGIFKYNLNCYEKKAKDILKYCILPHIKNNSEK 381

hsap|ENSG00000125630 FYMLCLMTRKLFALAKGECMEDNPDSLVNQEVLTPGQLFLMFLKEKLEG----------------------WLVSIKIA 437
pfal|PF3D7_1134700   FETMCFMFKKLIYSNFKLITPINKDSLENHAVTTCSNLLANLLKDQVMNSLCRLYIRYTRSFFKYFETKYNTFKLSVKQI 461

hsap|ENSG00000125630 FDKKAQKTSVSMNTDNLMRIFTMG------------------------------------------------------- 461
pfal|PF3D7_1134700   YLRYKMFVLEEMQEDRLNQISLSKSSEDINDSSYQDNNDYKNESKKKLKSIFINEKEKLFRSVDNYFKELYNDNYMFLEN 541

hsap|ENSG00000125630 ----IDLTKPFEYLFATGNLRSKTGLGLLQDSGLCVVADKLNFIRYLSHFRCVHRGADFAKMRTTTVRRLLPESWGFLCP 537
pfal|PF3D7_1134700   CKSFSNTSSAILFFFKTGNISSEN-LHYQQKSGWVIAADEINNLRLITNFRAIHRGTFFQDVKVLTPRKLLGESWGFICP 620

hsap|ENSG00000125630 VHTPDGEPCGLMNHLTAVCEVVTQFVYTASIP-------ALLCNLGVTPIDGAPHRSYSECYPVLLDGVMVGWVDKDLAP 610
pfal|PF3D7_1134700   VHTPDGTPCGLLNHLAQYCHIHNLSSNEAHKLNIKLYLKKIGINVNLDDTSGIHTIYDDENIPIIVDSIPITYIGEKDFN 700

hsap|ENSG00000125630 GIADSLRHFKVLREKRIPPWMEVVLIPMTGKPSLYPGLFLFTTPCRLVRPVQNLALGKEELIGTMEQIFMNVAIFEDEVF 690
pfal|PF3D7_1134700   RTVYKLKYAKNNNLFNLKSYFEINAYLN--EPLIMNSIIINTFPGRLIRPLLNLKTKDIEFISPSYQPYISVAINNEDVK 778

hsap|ENSG00000125630 AG------------------------------------------------------------------------------ 692
pfal|PF3D7_1134700   KNNLARKLLKKKEQVVNSMKIDRTPGSIKQKFLYHQNRENLLWKLKKESKDGSYKYEKHKIDRKLLKILDQKKNNDSDTN 858

hsap|ENSG00000125630 ----------------------------------------------------------------------VTTHQELFPH 702
pfal|PF3D7_1134700   TDNYVSSSEYDTDSDYDYGYDSNSDGDSKSNSNFDSKSDNISGSDNNMSTTTYIDSDENINIDMYEQIPQKFEYMELKET 938

hsap|ENSG00000125630 SLLSVIANFIPFSDHNQSPRNMYQCQMGKQTMGFPLLTYQDRSDNKLYRLQTPQSPLVRPSMYDYYDMDNYPIGTNAIVA 782
pfal|PF3D7_1134700   SFLSFLASLTPFSDHNQSPRNIYQCQMLKQTMGIQSLNNVYTFTNKIYRMITPQFPLVVTRDYEFYGVDNFPSGTNAVVA 1018

hsap|ENSG00000125630 VISYTGYDMEDAMIVNKASWERGFAHGSVYKSEFIDLSEKIKQGDSSLVFGIKPGD-----------PRVLQKLDDDGLP 851
pfal|PF3D7_1134700   ILAYTGYDMEDALIINKASADRGIFRTHIYKTEFIDLQKVGESSNFVFGNNIRYLNNNKNNNKNLAYENKGKFLNKDGLP 1098

hsap|ENSG00000125630 FIGAKLQYGDPYYSYLNLNTGESFVMYYKSKENCVVDNIKVCSNDTGSGKFKCVCITMRVPRNPTIGDKFASRHGQKGIL 931
pfal|PF3D7_1134700   CVQQKITEGSPLYSYINKGNELTTYESFRNHGEYFVDFVSIGKNNSN----QVAVVKLRSTRQPQVGDKFASRHGQKGVV 1174

hsap|ENSG00000125630 SRLWPAEDMPFTESGMVPDILFNPHGFPSRMTIGMLIESMAGKSAALHGLCHDATPFIFSEE------------------ 993
pfal|PF3D7_1134700   SRLFPQEDMPFAESGIVPDIIFNPHGIPSRMTIGMLIESICGKAASLYGKRIDATPFRKYTQQKSFNNPWIDNCGIKGFL 1254

hsap|ENSG00000125630 ----------------------------------------------------------------------NSALEYFGEM 1003
pfal|PF3D7_1134700   EKNDTIKSDLDTCQKEKDKNKNINNNNNNKIKENKYNNSNDSNASIDSDDNTNNCNDKRDEEEKKANITYDEKIDYFAKL 1334

hsap|ENSG00000125630 LKAAGYNFYGTERLYSGISGLELEADIFIGVVYYQRLRHMVSDKFQVRTTGARDRVTNQPIGGRNVQGGIRFGEMERDAL 1083
pfal|PF3D7_1134700   LLNKGYDYYGTELLYSGIYGVPLQAHIFIGVIYYQRLRHMAYDKAQVRRTGPVCNLTHQPLKGKRKHGGIRVGEMERDGI 1414

hsap|ENSG00000125630 LAHGTSFLLHDRLFNCSDRSVAHVCVKCGSLLSPLLEKPPP----SWSAMRNRKYNCTLCSRSDTIDTVSVPYVFRYFVA 1159
pfal|PF3D7_1134700   ISHGCSFVINERFLMSSDGHECFVCPKCGLILSPIMQFNTTGKIMKGRSIGGKSKMAVCKSCDVSCKIIFIPYVIRYLLN 1494

hsap|ENSG00000125630 ELAAMNIKVKLDVV--------- 1173
pfal|PF3D7_1134700   ELICLNVTIRLNMKSVENVFDMK 1517
```

Figure S2.3. RPA12

```
pfal|PF3D7_0407300   MFFTSKFLFNKNVNNDETFDSRSKHSGDEEDEGYDKSDIKEENNNIKKKHNSKKKEKRKDGHKNKDTNIDLNADTDKNKN 80
hsap|ENSG00000066379 -------------------------------------------------------------------------------- 0

pfal|PF3D7_0407300   KNTKKLSQELENINKEKKKVHFEEDEITSEELLNYFIKSYKNKINIENDELCQILTGKKYYDIERLAKNATEITQYDLKN 160
hsap|ENSG00000066379 -----------------------------------------------------------MSVMDLAN-----------T 9

pfal|PF3D7_0407300   KNIQKNKQSYCKTCGYIYNYDDYMYLFMKYFQLLNIKNHNEFSYDIIKCIYCGSVLGDIEILFNQFNNNENYIELYSYRE 240
hsap|ENSG00000066379 CSSFQSDLDFCSDCGSVLPLPG--------------------AQDTVTCIRCG------------FNINVRDFEGKVVKT 57

pfal|PF3D7_0407300   KYIFDKNKKDYWKNKITSFNKNTSLFKEEEKTAYNITYEKCLDCGNDFLHFINIQTRSADEGSTIIYFCPNCKKQTTVNN 320
hsap|ENSG00000066379 SVVFHQLG-----------TAMPMSVEEGPECQGPVVDRRCPRCGHEGMAYHTRQMRSADEGQTVFYTCTNCKFQEKEDS 126
```

Figure S2.4. RPAC1

```
hsap|ENSG00000171453 MAASQAVEEMRSRVVLGEFGVRNVHTTDFPGNYSG--YDDAWDQDRFEKNFRVDVVHMDENSLEFDMVGIDAAIANAFRR 78
pfal|PF3D7_1143300   ---MENVKYRDNFVQLGEEGPRNATTTNFYGSYYFSDKENYFDIRKFEENLEMNVIKNEENILILEIKNMDVSIANALRR 77

hsap|ENSG00000171453 ILLAEVPTMAVEKVLVYNNTSIVQDEILAHRLGLIPIHADPRLFEYRNQGDEEGTEIDTLQFRLQVRCTRNPHAAKDSSD 158
pfal|PF3D7_1143300   IMLSEVPTIAIEKVNMYQNTGIIADEILCHRLGLIPFKFDADLINYK-EDNEKYNHLNCFCFKLHVKFS-----SKRTGN 151

hsap|ENSG00000171453 PNELYVNHKVYTRHMTWIPLG-NQADLFPEGTIRPVHDDILIAQLRPGQEIDLLMHCVKGIGKDHAKFSPVATASYRLLP 237
pfal|PF3D7_1143300   DN----YQSIYSKDLKWCPINEQQRIKFEKNPPKVVDENILITKISSGQEIELICFLQKGIGKTHAKWSPVCTAVYKMYP 227

hsap|ENSG00000171453 DITLLEPVEGEAAEELSRCFSPGVIEVQEVQGKKVARVANPRLDTFSREIFRNEKLKKVVRLARVRDHYIFSVESTGVLP 317
pfal|PF3D7_1143300   HFSFNT-NEQLSYEEKKDLVNICPQKVFDIEDSQQIFVKNP--LSCSTCRVCIEKYPKKISFQKEKNHFIFTIESTGCFS 304

hsap|ENSG00000171453 PDVLVSEAIKVLMGKCRRFLDELDAVQMD 346
pfal|PF3D7_1143300   AADIFKKALFILRQKVINVKEALDEQLCP 333
```

Figure S2.5. RPAC2

```
hsap|ENSG00000186184 MEEDQELERKISGLKTSMAEGERKTALEMVQAAGTDRHCVTFVLHEEDHTLGNSLRYMIMKNPEVEFCGYTTTHPSESKI 80
pfal|PF3D7_1415200   MEEKKYYENLKN-----------------------LTHATFCFENEDHTLGNCLRCILLQKEGVEFAGYTVPHPTQPEI 56

hsap|ENSG00000186184 NLRIQTRGTLPAVEPFQRGLNELMNVCQHVLDKFEASIKDYKDQKASRNESTF 133
pfal|PF3D7_1415200   NVRIQTTG-KPAIDILKESLDDLGNMCDIMLNKFNEALKQS------------ 96
```

Figure S2.6. RPABC1

```
hsap|ENSG00000099817 MDDEEETYRLWKIRKTIMQLCHDRGYLVTQDELDQTLEEFKAQSGDKPSEGRPRRTDLTVLVAHNDDPTDQMFVFFPEEP 80
pfal|PF3D7_1364800   --MEDPVTRFYKCRKTCCEMLEDRGYIMTPREKLENFSTFKEMFEDNDRQ----RSKMGISTTHKNDSNNRIIVYFADEV 74

hsap|ENSG00000099817 K-VGIKTIKVYCQRMQEENITRALIVVQQGMTPSAKQSLVDMAPKYILEQFLQQELLINITEHELVPEHVVMTKEEVTEL 159
pfal|PF3D7_1364800   KKTGVKPLRELTERMEEKSIQRAILVTQNILTPFARDAIKEAAPRHIIENFLDTELLVNITKHELVPRHIPLTSDEKKNL 154
```

```
hsap|ENSG00000099817 LARYKLRENQLPRIQAGDPVARYFGIKRGQVVKIIRPSETAGRYITYRLVQ 210
pfal|PF3D7_1364800   LQRYKIMENKLPRIQDVDPVCRYFGLSKGQVVKIIRPSETAGRYVTYRLVV 205
```

Figure S2.7. RPABC2

```
hsap|ENSG00000100142 MSDNEDNFDGDDFDDVEEDEGLDDLENAEEEGQENVEILPSGERPQANQK------------RITTPYMTKYERARVLGT 68
pfal|PF3D7_0303300   MDGFNDNYLNEDEDFMGDEFGQGIDSDDGENYENDIDIITDHQIKKDNKSDYENSEANEDNIRITSPYLTKYEKARIIGT 80

hsap|ENSG00000100142 RALQIAMCAPVMVELEGETDPLLIAMKELNARSASEPPGEEPPWMDRGTRDEHLPSCPGCPAVASNTRCCPPAACLPGIS 148
pfal|PF3D7_0303300   RALQISMNAPLTIPIETSNDMINSKNEYDNYLNNDPLVIAEKELYNK--------------SIPFILRRYLPNGSYEDWR 146

hsap|ENSG00000100142 LSRRRRLREE 158
pfal|PF3D7_0303300   LDELIID--- 153
```

Figure S2.8. RPABC3

```
hsap|ENSG00000163882 -MAGILFEDIFDVKDIDPEGKKFDRVSRLHCESESFKMDLILDVNIQIYPVDLGDKFRLVIASTLYEDGTLDDGEYNPTD 79
pfal|PF3D7_1213700   MASNILFEDRFVISSVD--NSKFEKVSRIKAKSTGYDAELILDVHSELFKVEEKKAIYLALQDKLLNRNDEKGWEQNEN- 77

hsap|ENSG00000163882 DRPSRADQFEYVMYGKVYRIEGDETSTEAATRLLRLRAAEWQCSRITGWGLLFQLCVRVLWGPAHEAAGGCQQPAWIRGG 159
pfal|PF3D7_1213700   ---VPLNNIEYIMSGRIFKFEELSS--------------ERRTVYASFGGLMMALTTDKQFIGDLEID--------MKIY 132

hsap|ENSG00000163882 LQSLSPDEEASLLNLA 175
pfal|PF3D7_1213700   LLTKNIDIERTRE--- 145
```

Figure S2.9. RPABC4

```
hsap|ENSG00000147669 MDTQKDVQPPKQQPMIYICGECHTENEIKSRDPIRCRECGYRIMYKKRTKRCILLTMLSKYELGGNE 67
pfal|PF3D7_1342700   MYIREQDEDISTEPVVYICGECGIDTVIPPNASLRCKNCGSKIFFKKRSRRVMQYEAR--------- 58
```

Figure S2.10. RPABC5

```
hsap|ENSG00000177700 MIIPVRCFTCGKIVGNKWEAYLGLLQAEYTEGDALDALGLKRYCCRRMLLAHVDLIEKLLNYAPLEK-- 67
pfal|PF3D7_0708100   MIIPVRCFTCGKLIGNLWSVYEKKLEEGLSKCDALNELNLYRYCCRRMILTHADMMDKLLCYNIYERKL 69
```

Figure S2.11. RPA43

```
hsap|ENSG00000105849 MAAGCSEAPRPAAASDGSLVGQAGVLPCLELPTYAAACALVNSRYSCLVAGPHQRHIALSPRYLNRKRTGIREQLDAELL 80
pfal|PF3D7_0208700   ------------------MAQNPWFVKKSKTLRTSQLEKFINKFNEEYEHLMHMTRFKYIKRTLESIKENSDLIINKKTF 62

hsap|ENSG00000105849 RYSESLLGVPIAYDNIKVVGELGDIYDDQGHIHLNIEADFVIFCPEPGQKLMGIVNKVSSSHIGCLVHGCFNASIPKPEQ 160
pfal|PF3D7_0208700   SILRISCVAQLQPKYLNKIDDGISVYLSN--FMLKANHDVEGFC------------------------LCFNKIKLKEK- 115

hsap|ENSG00000105849 LSAEQWQTMEINMGDELEFEVFRLDSDAAGVFCIRGKLNITSLQFKRSEVSEEVTENGTEEAAKKPKKKKKKKDPETYEV 240
pfal|PF3D7_0208700   --------------------------------ESRVMNNDPSIMFVKISFKLLILVL-----------------KENYEI 146

hsap|ENSG00000105849 DSGTTKLADDADDTPMEESALQNTNNANGIWEEEPKKKKKKKKHQEVQDQDPVFQGSDSSGYQSDHKKKKKKRKHSEEAE 320
pfal|PF3D7_0208700   KAKINKIEP-----------LKIHLDIFGIVEAIFSEDMFKDFHYDSRNNRFRREGKFFSLYDIVLFTIKKITYGDNGAN 215

hsap|ENSG00000105849 FTPPLKCSPKRKGKSNF 337
pfal|PF3D7_0208700   VKVIGYF---------- 222
```

Figure S2.12. RPC6

```
pfal|PF3D7_1421400   MNN--------------INKQLVKD--IYQIGLEHKEAISIDSLEEIYEKKKKGKLKRNEIVYALNILENARACSIKNEN 64
hsap|ENSG00000132664 MAEVKVKVQPPDADPVEIENRIIELCHQFPHGITD--QVIQNEMPH---------IEAQQRAVAINRLLSMGQLDLLRSN 69

pfal|PF3D7_1421400   NTIITRMRNEQVTKKLKELSDIDFLIFTKVENSQNNGIWTADLRKQTKLLIHQVQKGVKLLCENKLIKQVNNIHVKNRKM 144
hsap|ENSG00000132664 TGLLYRIKDSQNAGKMKGSDNQEKLVYQIIEDAGNKGIWSRDIRYKSNLPLTEINKILKNLESKKLIKAVKSVAASKKKV 149

pfal|PF3D7_1421400   YILYDLEASEKVIGGSFYTDGEFNKKVVDYIRENICFYLYNNNNSS----------------VPSVINYIKKLNNSVDY 207
hsap|ENSG00000132664 YMLYNLQPDRSVTGGAWYSDQDFESEFVEVLNQQCFKFLQSKAETARESKQNPMIQRNSSFASSHEVWKYICELGISKVE 229

pfal|PF3D7_1421400   FSENDIYRVIKTLSYEERINIYKSNNDEEL--------IYYN---NEKKNFLYNFPCFSCNLFNKCNSDINTTINPRSC 276
hsap|ENSG00000132664 LSMEDIETILNTLIYDGKVEMTIIAAKEGTVGSVDGHMKLYRAVNPIIPPTGLVRAPCGLCPVFDDCHEG--GEISPSNC 307

pfal|PF3D7_1421400   MYLNFYLNLENE 288
hsap|ENSG00000132664 IYMTEWLEF--- 316
```

Figure S2.13. RPC4

```
hsap|ENSG00000168495 MSEGNAAGEPSTPGGPRPLLTGARGLIGRRPAPPLTPGRLPSIRSRDLTLGGVKKKTFTPNIISRKIKEEPKEEVTVKKE 80
pfal|PF3D7_1463400   MSNRYNHDEYS----------------LRRSINNTLRSKSSFLNNRDENNQNITLKKFVPNIDNLKNE------NNVRK- 57

hsap|ENSG00000168495 KRERDRDRQREGHGRGRGRP--EVIQSHSI----FEQGPAEMMKKKGNWDKT-VDVSDMGPSH------IINIK-----K 142
pfal|PF3D7_1463400   ---NDNDNKFEEDEEGLNNKHLEQLIKKTINIDLENEGISTKTTSKNKYNNKEYPSHAIDPATLNNNVNLLNIEALNLCK 134

hsap|ENSG00000168495 E-KRETDEETKQIL-----RMLEKDDFLDDPGLRNDTRNMPVQLPLAHSGWLFKEENDEPDVKPWLAGPKEEDMEVDIPA 216
pfal|PF3D7_1463400   EESLEKKNKVKTSIDIYELNNISKNIFYNKKCASGDVHFLPLTLPFF--------------------------------- 181

hsap|ENSG00000168495 VKVKEEPRDEEEEAKMKAPPKAARKTPGLPKDVSVAELLRELSLTKEEELLFLQLPDTLPGQPPTQDIKPIKTEVQG--- 293
pfal|PF3D7_1463400   -----------------------------NKNEKQKKIRKLLLDKEPFFFSIQLPNMLPALLSKEEEEQNGEQTKNDTK 231

hsap|ENSG00000168495 --------EDGQVVLIKQEKDREAKLAENACTLADLTEGQVGKLLIRKSGRVQLLLGKVTLDVTMGTACSFLQELVSVGL 365
pfal|PF3D7_1463400   QNDQTEKNEKKKKSIINKSST---NDSYQLSNIHTIPNGKFAKLIIYKNKKIKMKINDILFDINEGSACTFSQEIGCF-- 306

hsap|ENSG00000168495 GDSRTGEMTVLGHVKHKLVCSPDFESLLDHKHR 398
pfal|PF3D7_1463400   -IKENSEFIFLGNCDYKLVATPNIERIIHKK-- 336
```

Figure S2.14. General transcription factor IIE subunit 1

```
hsap|ENSG00000153767 MA-DPDVLT-EVPAALKRLAKYVIRGFYGIEHALALDILIRNSCVK-EEDMLELLKFDRKQLRSVLNNLKGDKFIKCRMR 77
pfal|PF3D7_0717300   MENSKDIFYDKEKKFFQYLMIYISRFFMSDEEIVIFDLFINNECLYLEKDIVNNINMNEQKIRSILSKLLKDKFIEIQK 80

hsap|ENSG00000153767 VETAADGKTTRHNYYFINYRTLVNVVKYKLDHMRRRIETDERDSTNRASFKCPVCSSTFTDLEANQLFDP-MTGTFRCTF 156
pfal|PF3D7_0717300   YKNNEKGTNFQTFYCLNNY--IVYVLDFRIKQMENELQK---KKNESDIYICNFCNATYSQLDAQILPLDSYDAHFLC-Y 154

hsap|ENSG00000153767 CHTEVEEDESAMPKKDARTLLARF-------NEQI-------EPIYALLRETEDVNLAYEILEPEPTEIPALKQSKDHAA 222
pfal|PF3D7_0717300   CNNKIELIENDDH--NDEKIYNKYTKYLNILKEHIEKLKNYFIPLYTEKFSKKNVNSNSFFFER----------SS---- 218

hsap|ENSG00000153767 TTAGAASLAGGHHREAWATKGPSYEDLYTQNVVINMDDQEDLHRASLEGKSAKERPIWLRESTVQG-AYGSEDMKEGGID 301
pfal|PF3D7_0717300   -------------DESFTN----------NSSEVSMTNNSSLI----------------SQSQLNKVVDGGKRKKDDGLT 259

hsap|ENSG00000153767 MDAFQEREEGHAGPDDNEEVMRALLIHEKKTSSAM--------AGSVGAAAPVTAAN-GSDSE-----SETSESDDDSPP 367
pfal|PF3D7_0717300   DT-----------------CSSSILKKDKKIKICMNVKSKKSVSKHVEHEDPTKNVRKNSNAKVEDFISKEKERKNKQNE 322

hsap|ENSG00000153767 RPAAVAVHKREEDEEEDDEFEEVADDPIVMVAGRPFSYSEVS-------QRPELVAQMTPEEKEAYIAMGQRMFEDLFE 439
pfal|PF3D7_0717300   KMAQQNVSKSNENA---K----QLEEEQMLEPELPFFYVKKYNKRFSLLDAQKLQQDMTQEEFENFMELQETYLDQL-- 392
```

Figure S2.15. General transcription factor IIE subunit 2

```
hsap|ENSG00000197265 MDPSLLRERELFKKRALSTPVVEKRSASSESSSSSSKKKKTKVEHGGSSGSKQNSDHSNGSFNLKALSG---SSGYKFGV 77
pfal|PF3D7_0110800   MS-SISPNKKFALTSASDKPITLIRTNVSNENNNVRRKMLRNFR--NSRNTTCPDCKEKGIIICDTSEGTQICNGCGMVV 77

hsap|ENSG00000197265 LAKIVNYMKTRHQRGDTHPLTLDEILDETQHLDIGLKQKQWLMTEALV-------------------NNP---------K 129
pfal|PF3D7_0110800   ESKILSEEQ------EWRNFQNDGQSKNTDRNRVGEVSDIWLENNLTSTTFIKSSKKLQHLNMMTQINKNDQTLISAFNI 151

hsap|ENSG00000197265 IEVIDGKYAFKPKYNVRD--------------------------------------KKALLRLLDQHDQRGLGGILLEDI 171
pfal|PF3D7_0110800   LKLICDTFFLRSNVIERAKEITKELQDMEQLKNRINNLNMLAVVYLACREAGHIKSIKELITFDRSYKEKDLGK-TINKL 230

hsap|ENSG00000197265 EEALPNSQKAVKALGDQILFVNRPDKKKILFFNDKSCQFSVDEEFQKLWRSVTVDSMDEEKIEEYLKRQGISSMQE---- 247
pfal|PF3D7_0110800   KKVLPSR-----------AFVYNENISHLIYSLSNRLQLSID----------------LIEAIEYVVKKASTLITTSHRL 283

hsap|ENSG00000197265 ----SGP-KKVAPIQRR-----KKPA------------SQKKRRFKTH----------------NEHLAGVLKDYSDITS 289
pfal|PF3D7_0110800   NSLCGGSIHLIVELNTNEEKNMKLPNLSQIATVCGVTTNTLKTTFKELLNAAEYIIPKYYLSEDNPKLSMLKQKYLSEDK 363

hsap|ENSG00000197265 SK-- 291
pfal|PF3D7_0110800   RKKN 367
```

Figure S2.16. General transcription factor IIF subunit 1

There appears to be no ortholog for this protein in Plasmodium

Figure S2.17. General transcription factor IIF subunit 2

```
hsap|ENSG00000188342 MAERGELD--LTGAKQNTGVWLVKVPKYLSQQWAKASGRGEVGKLRIAKTQGRTEV---SFTLNEDLANIHDIGGKPA-- 73
pfal|PF3D7_1144600   MNNSKENDSVSKLSFKNKNIKLIKVPKFVSNKWLQYNNKDIVGLLGCNNNNNNDSEITELYVQKDDSDNVKKLRCNKNNT 80

hsap|ENSG00000188342 ------------------SVS------APREHPFVLQSVGGQTLTVFTESSSDKLSLEGIVVQR---AEC------RPAA 120
pfal|PF3D7_1144600   VNTYILKQNVIKLKSTNKSVSNSAGTLNNKSNDYTNTNKGKNTMNNLSKDNSDKHKEFDYVVCADLIKTCDYTYSFLPTL 160

hsap|ENSG00000188342 SENYMRLK------------RLQIEESSKPVRLSQQLDKVVTTNYKPV-ANHQYNIEYERKKKEDGKR------------ 175
pfal|PF3D7_1144600   DQDYSSILKERHYKTNVKKERFTIIETRNE----ENMDATH-TLFKYYTSEDKLNNESKDKNMKNNKRLFTDNDNLNTVS 235

hsap|ENSG00000188342 --------ARADKQHVLDM------LFSAFEKHQYYNL--KDLVDITKQPVVYLKEILKEIGVQNVKGIHKNTWELKPEY 239
pfal|PF3D7_1144600   HSTSKQKAKQSKKMHVFDLDKAKISIFKIFEREGQNGVPFSFFTKSFNIPANHIKGILDEIAIKGKRDTDKKTVYFL--- 312

hsap|ENSG00000188342 RHYQGEEKSD 249
pfal|PF3D7_1144600   KNCIG----- 317
```

Figure S2.18. UAF

```
yeast_UAF30     MAELNDYSTMIDILLSDMDLETVTTKKVRMALKEVYAIDVESQGKAINKLIRKHLDLVKERPRFERSLEDLLKENATLAI 80
PBANKA_1233000  ---------------MNTIRAKLFNSKNKIVGYESFSPLNKFMTKYNYSTIDKNNEIVKEKKPNGLQIECEIKS------ 59

yeast_UAF30     ELTKEITVSKRSSGEEKNDSETKGTHVEKKKGTVSKSPISTRKVTLSKSLASLLGEHELTRTEVVRRLWAYIKAHNLQNP 160
PBANKA_1233000  ------------------------------------------------PLKEFLNTNTASRVFVLKYAWKYIKDNNLQNP 91

yeast_UAF30     NNKKEILCDEKLELILGKS-TNMFEMHKILASHMTEPKKISDCPPLIQEVRRKEKPIVSDSEQSDTKGI 228
PBANKA_1233000  DMKRKIIPDKKLKQVLEKDEVDMLEIPKLLFKHVISYRKE----------------------------- 131
```

Figure S3. Multiple Protein Sequence Alignments

https://www.ebi.ac.uk/jdispatcher/msa/clustalo
https://www.bioinformatics.org/sms2/color_align_cons.html

Species abbreviations used in the following alignment pages:

bmic	Babesia microti strain RI
chot	Cryptosporidium hominis isolate TU502_2012
cvel	Chromera velia CCMP2878
etht	Eimeria tenella Houghton 2021
gnip	Gregarina niphandrodes Unknown strain
hsap	Homo sapiens
htar	Haemoproteus tartakovskyi strain SISKIN1
pber	Plasmodium berghei ANKA
pfal	Plasmodium falciparum 3D7
scer	Saccharomyces cerevisiae S288C
sneu	Sarcocystis neurona SN3
tequ	Theileria equi strain WA
tetr	Tetrahymena thermophila (strain SB210)
tgon	Toxoplasma gondii ME49
vbra	Vitrella brassicaformis CCMP3155

Figure S3.1. RPA1

```
htar|Htart_000206800 -----------------------------------------------------MYDVNAVQCVNVESVQLDVLSSEELRK 27
pber|PBANKA_1109000  -----------------------------------------------------MYDINSAKCAEIKSAELGVLSSQELKR 27
pfal|PF3D7_0509400   -----------------------------------------------------MYDINKSICAEIKSAELDVLNSGELRR 27
etht|ETH2_0728100    ------------------------------------------------------MDPNSLVSSNVKSARLEFLGDDEIRK 26
sneu|SN3_00201460    -----------------------------------------------------MFDANSTVCTSVHSASLDMYAPQEVEI 27
tgon|TGME49_244880   -----------------------------------------------------MYDANATICSSVHSASLTLFSQDEVRA 27
gnip|GNI_013220      ----------------------------------------------------MSFDANGVICTDINKASFQLLSEEDVKK 28
chot|ChTU502y2012_38 -------------------------------------------------------MNSAPICSDIHSASFDLLRASEVKK 25
bmic|BMR1_03g03880   -----------------------------------------------------MLDFSTAVTSEISGAYFTLLTNEEVAA 27
tequ|BEWA_039100     -----------------------------------------------------MLSYDTAVSTEVEGISLSFLSNEEIHT 27
cvel|Cvel_8831       -----------------------------------------------------MLDPRKPAAAQVASAQFICLSSDDVRR 27
vbra|Vbra_21242      -----------------------------------------------------MIDATGAVSTEVRRLTFAMQTKEEIRA 27
tetr|Q23DD6          MNAHCVQNQFRVQKIESKKEAIKERNKQDKLKDKKQKQKELLLYTYRYQMSNSLGQFQEVISRRLAGVGFSYYTADEIRK 80
hsap|ENSG00000068654 -----------------------------------------------------MLISKNMPWRRLQGISFGMYSAEELKK 27
scer|YOR341W         -----------------------------------------------------MDISKP-VGSEITSVDFGILTAKEIRN 26

htar|Htart_000206800 ISVGEYGSYNRVEWEDHKSKK--GMVWDVKQLMNQLKMEQQSNFLGDESGMNVEANGTDVKNVSNANATEASKKQVKESK 105
pber|PBANKA_1109000  ISLGKYENQLQ-EFEKI-SKD----------------------------------------------------------- 46
pfal|PF3D7_0509400   ISMGKYENEKY-EYEKT-HKS----------------------------------------------------------- 46
etht|ETH2_0728100    LSCCRVVTAAAFSAPAGGAAGSSSRKWGADA-----------------------------AAA--------------AAA 63
sneu|SN3_00201460    FSACKITETHVFHSIDEGTFAVPAATVAAAA-----------------------------AAA--------------GAA 64
tgon|TGME49_244880   LSSCKISDTNVFLDPHASSSSL----------------------------------------S--------------ATF 53
gnip|GNI_013220      TAVCRIFSTVIYDGEQ---------------------------------------------------------------- 44
chot|ChTU502y2012_38 LSVCHVTSTTTFESTG---------------------------------------------------------------- 41
bmic|BMR1_03g03880   LKRVNVNSAVPFDANG---------------------------------------------------------------- 43
tequ|BEWA_039100     LSVSEIKNIGRSGYES---------------------------------------------------------------- 43
cvel|Cvel_8831       RSACEVTSLDVWEKEEG--------------------------------------------------------------- 44
vbra|Vbra_21242      QSVTRIDRVGVWTP-LG--------------------------------------------------------------- 43
tetr|Q23DD6          LSVKEITNPIAFDS-LN--------------------------------------------------------------- 96
hsap|ENSG00000068654 LSVKSITNPRYLDS-LG--------------------------------------------------------------- 43
scer|YOR341W         LSAKQITNPTVLDN-LG--------------------------------------------------------------- 42

htar|Htart_000206800 AARIAVMPVNVKKKKTRTLDPRYG---TEMR-----GQICEVCFEEHSFCTGHVGHLEVALPLFNPLFFEDIYSILKMVC 177
pber|PBANKA_1109000  -----------GTSNRITYDPRFG---TIDY-----SQICSVCFEKHDGCLGHIGHIEFVLPVFNPLFYKDLQELLNLVC 107
pfal|PF3D7_0509400   -----------GVVTKMYYDPKYG---TIDH-----NQICSVCFERHENCTGHIGHIEFVLPLFNPLFYKELQELLNLIC 107
etht|ETH2_0728100    AAAAAGLA---AAVPGGLHDSRMG---VFDG-----RETCGTCGAS-GSCPGHLGHIELELPALQPILMPSLVKLLKGVC 131
sneu|SN3_00201460    SASSGGLP---TPVVGGLYDRRLG---SLDG-----REPCATCGCR-SDCPGHPGHVQLALPIFQPALLPSLVKILKFTC 132
tgon|TGME49_244880   TAARGGGP---VAVSGGLHDSRLG---SLDG-----REICETCGCR-SDCPGHLGHIDLALPVFQPIFLPSLVKVLKLSC 121
gnip|GNI_013220      -----------A-KLGGIHDPRLG---PMDSEPRRGDPSCVTCGGR-DDCPGHLGYIQLYQPLYHPLFMPTLVRLLKGTC 108
chot|ChTU502y2012_38 -----------ESKVGGLHDLRMG---PLSN-----RDFCGTCGSR-NECPGHIGHIELELPVFHPLFLGNLVRILKCCC 101
bmic|BMR1_03g03880   -----------A-TINGVHDPAMG---TNDG-----RDKCSTCNGT-SDCNGHLGIIEFPLPLYHPLFKSSLVKLLKLMC 102
tequ|BEWA_039100     -----------A-GKSCIYDPALG---SSDI-----YKHCSSCNEM-TNCDGHLGHIDFAVPLFHPLMLPSLVKLLKTVC 102
cvel|Cvel_8831       -----------NPKEGGLHDLRMGALRGVQQ-----STRCRSCKLTEKHCPGHPGHIELREPCYNPLFFSALTQIMKLVC 108
vbra|Vbra_21242      -----------LADARGVHSPLLG---PLDK-----TMPCPTCGLT-NECPGHNGHIELSTPMFHPSMMQPLATILRAVC 103
tetr|Q23DD6          -----------RPIKGGLYDPCMGVS-PYDK-----FTRCETCGCEGLECPGHMGHVELLLPVYNPFLIDKLLKLLRAQC 159
hsap|ENSG00000068654 -----------NPSANGLYDLALG---PADS-----KEVCSTCVQDFSNCSGHLGHIELPLTVYNPLLFDKLYLLLRGSC 104
scer|YOR341W         -----------HPVSGGLYDLALG---AF-L-----RNLCSTCGLDEKFCPGHQGHIELPVPCYNPLFFNQLYIYLRASC 102

htar|Htart_000206800 FHCFSISFDGKKLLHMKHEYNLKALSDIAKGSELRVLDKGVSQLNYTI-------------EKTEQI--YEEVLRAGVTE 242
pber|PBANKA_1109000  YNCYGLCYSEDVIFLLKHIYQLKSLNEIESSNKFII--KKNNENEYVV-------------SEIERL--YNKICKESNIT 170
pfal|PF3D7_0509400   YHCYNFCCSEDVIFLLKHIFQLKSLNQIESTNKIKY--KKNCDYEYII-------------DQVEKL--YKKICKDSNIS 170
etht|ETH2_0728100    FECRRLRLSRQQKSLEVKRFQLLQLLLLQELRLMDTVVTRNSSSSS-SSS----------KAAGNE---EALKSRKEILQ 197
sneu|SN3_00201460    LYCGRLRISAAVSSLMIKAFELLQLGRFEELQKVIEIAVSSLRTTPAARS----------ALEGECL-PFSAGQRAALSR 201
tgon|TGME49_244880   LHCERLRVSTPAASLFVKAFELLQLGCFDEVEEASRCASSALRAPTRG------------GSAG--L-ALPQSQRVAVLR 186
gnip|GNI_013220      LLCRRLKIDPGLATMYLKKFQMVQMGALQMLEEFEQLSSMSSGGRYEQ---------------------SSL-------- 159
chot|ChTU502y2012_38 WHCCCIRLSKKVCNGYVKRFESIGKNSNV---------------DSKD---------------------LE--------- 136
bmic|BMR1_03g03880   FQCCRLKIPNKRVKEFLIKFRLLRSGLISRFAKYSLLTNLSK-------------------------------------- 144
tequ|BEWA_039100     FYCLKLKLRKKQVLKFQRLFDLAKAGMLSDLIVFDQLFGNDKDDDEKD---------------------AEASNRTKLHQ 161
cvel|Cvel_8831       INCRRLRLPPGARQIYATRFKLLSTETLSDSAYHEALTNPSLLHGEK----GDLPIQVYAGDDTETARSAAESLRKLRRR 184
vbra|Vbra_21242      FRCNKLKMPTDKQESFVKRFKLLGLGGLGEMRTFTRARMPMSEKFAEELLKSLLEENPDT--ALNETEAIADEFDRARDR 181
tetr|Q23DD6          LKCNKFRMTNQKLKMFANVFTLIKLGYIFEAKEYKQTCESL----------SILQIQNNK--ETTQIQSYADKRRKSSDY 227
hsap|ENSG00000068654 LNCHMLTCPRAVIHLLLCQLRVLEVGALQAVYELERILN------------RFLEENPDP--SA---------------- 154
scer|YOR341W         LFCHHFRLKSVEVHRYACKLRLLQYGLIDESYKLDEITL------------GSLNSSMYT--DD---------------- 152

htar|Htart_000206800 MENILESSSENEES---DGEGEKKESSRYEDQMESDCTGKFFKEKGENIHDLND----FSMFTYGENYKKKEKQRM--NK 313
pber|PBANKA_1109000  IEDIVESICQDYENSFGNSGNGNNENGSHEDNQTYELWKKINDD----------------------MYSDD-------QK 221
pfal|PF3D7_0509400   LDDIVEAIYDDYENVHEDKDGNFDESVD-----LLDSLEKIKDEPDEKNKNNNNKNNNNNNNNNNNNYNNDVDIDVDNNN 245
etht|ETH2_0728100    IFAAVEAELNARMQ---QQQQ-Q----------EEQ-----------------------------------------QDK 222
sneu|SN3_00201460    AFRILEEAENAASS---ADEG-G----------DAECEATVRE-----HH------HYYRLAEGKDLYVKEETGE-RASS 255
tgon|TGME49_244880   IFRKIEEELQRRAG---ADAP-L----------AASREAPA----------------------------SPAKGA-QASV 223
gnip|GNI_013220      ----------------------------------A--------------------------------------------- 160
chot|ChTU502y2012_38 -------------------------------------------------------------------------------- 136
bmic|BMR1_03g03880   ----------------------------------K--------------------------------------------- 145
tequ|BEWA_039100     LS-------------------------------KE--------------------------------------------- 165
cvel|Cvel_8831       ELRKLKERLNAREEQIRQSIGGQ--------------------------------------------------------- 207
vbra|Vbra_21242      ANASV-NDFIGRESRR-RGVG----------------------------------------------------------- 200
tetr|Q23DD6          KSSDT-EQIMPKKSQI-EQEGAK--------------------------------------------------------- 248
hsap|ENSG00000068654 ------SEIR---------------------------------------------------------------------- 158
scer|YOR341W         ------EAIEDNEDEM-DGEGSK--------------------------------------------------------- 168

htar|Htart_000206800 QSVDVD----VADFCKL-ELESENEYGLYKEKNKY----QNEKETWEN------ET---EDACRKCKY--QFDINKLAHM 373
pber|PBANKA_1109000  N---------QGDFSKK-FAK--KKNSNYDLKNQKSNQEIENKETKEK------IK---FLKEKIKKY--KFDISKLAFQ 278
pfal|PF3D7_0509400   NNVDINVDNNNNYFNSD-LVDLAPNEEIYEEETDAGRSDDNNKENKPD------YN---KLKERIKKY--KFDANKLAFQ 313
etht|ETH2_0728100    EQQDVN----------KPAAAAAAA---AD------------KEDTGV-----------AAAVKETFM--LQHIAEDFSS 264
sneu|SN3_00201460    RSAAFEEGADLSASTRKRGATAAPAPGEAE--------GQPEQDRRHKARLFLQKL--DIGE-AEDAR--LTHFNEDVSQ 322
tgon|TGME49_244880   EFEDLEERASPGETRRKTRETQAAV---TE--------KDEAKEQQRVARLLSEST--DFEKEKTRLL--LHSFGSDISA 288
gnip|GNI_013220      ------------------------------------------ERRVEMRRLLADMKVL-EEKQRKEGK--LLNLEEDISV 195
chot|ChTU502y2012_38 ---------------------------------------------------IYN---------VNDA-----QSTDGIAS 151
bmic|BMR1_03g03880   ------------------------------------------SRMDKFERLFKKE---RRLCLEGLRK--RTAYHKIVKN 178
tequ|BEWA_039100     ------------------------------------------SKKDHIKKILFKLKKVAQVRSGGDAQ--ATNLTRLKNS 201
cvel|Cvel_8831       RSGALL--------------------TAYRDEQ--IHKPLSEREDFPQ-----D-----YGDSLS----MGVLPMTQVRF 251
vbra|Vbra_21242      -----------------------------------------------------E-----YFGERE----QDMFRDSTAKF 218
tetr|Q23DD6          ----IY--------------------QNN----------INNLEDKKK-----Q-----LLSLINVKQNNTEQFSKGVSY 284
hsap|ENSG00000068654 ----------------------------------------EEL----E-----Q-----YTTEIV----QNNLLGSQGAH 180
scer|YOR341W         QSKDIS--------------------STL----------LNELKSKRS-----E-----YVDMAIAKALSDGRTTERGSF 208

htar|Htart_000206800 TSYNYYEYLSLRDTLIRLAKRGAMNRCIFCMQPQVIKVKKADSCTGFTFVVNSNNYAH---LKDFSKYISESVPKEQTKK 450
pber|PBANKA_1109000  SNYNYEEYIILNKAIKLHMKKG--ANCSNCKFRRSITVKTSQKKDTINFIGTYTN------SSFFKKYISKNNKNGSFEE 350
pfal|PF3D7_0509400   SNYNYEEYLILSKTIKLHMKKS--SKCCFCKFQRNISAKVSQRRDTINFIGVHTN------NPFFKKYMPKKKQKKNEKD 385
etht|ETH2_0728100    SALQVAAWRSCRSRV--LQQAAAVNRCSHCGVANAVSVHLAPKNSGVELKWTWQSHLPYTSSSYF-GTLFDGGPQGGPPG 341
sneu|SN3_00201460    SAWQVSAWRHLRRLL--WTRVLSTTVCPNCGRSCSATFHVAQRSVAVEMRWKWTGKPPFDRKSVFFSSLHTRNSRQATED 400
tgon|TGME49_244880   TAWQVATWRKLRDLL--WTRAAASCVCANCGRSSAVTFHVAQQATGVEMRWKWTGQPPFDKNSALFSFLRNATPRREEEG 366
gnip|GNI_013220      SSSSIENWRRITRDL--YDRC-AQSHCTHCGRKFSSVIHKKDLDSNVEIQWNFDADCPVDRNILLKMLDAQEEIERV--G 270
chot|ChTU502y2012_38 NSYSLEIWEGIKREF--LDKASKHSSCSSCGNSFKGRVKESQLGIGITLSWPEGSKPPVSLGSTWSSSSNQEDTRSE--Y 227
bmic|BMR1_03g03880   SSTILHLYEETREIF--FGLK-NLQKCHHCKSACPSSIKAAGDGTTVDILWGNSIDYIS--------------------- 234
tequ|BEWA_039100     SNFDVNIWNEIRNRF--FAIGAKTKRCNHCGDRNSIAVKQSADSTHIEISWAPSSLEPKVFKDFQK-------------Y 266
cvel|Cvel_8831       DSF-----VSLRKEL--MDRCTKPSRCPNCGASQKYSVKPSPDQSY--FVVEWPSGAPDPLESAWKGVEPVVARPGQSLA 322
vbra|Vbra_21242      EIYM--ALRRGFI-V--YANKCANRRCPHCTHKCKMGVRPAAKWGVGLMQLSWGKK----VDSPWSQEQEARFMSDQRET 289
tetr|Q23DD6          TNAMNSAFEQALKEF--WNS-INIGKCPHCKEPQQA----IRKEGFSKFFIKK--------------------SSSEAKK 337
hsap|ENSG00000068654 VKNVCESKSKLIALF--WKAHMNAKRCPHCKTGRSV----VRKEHNSKLTITF--------------------------- 227
scer|YOR341W         TATVNDERKKLVHEF--HKKLLSRGKCDNCGMFSPK----FRKDGFTKIFETA--------------------LNEKQIT 262

htar|Htart_000206800 ERNE-----VLDQ-----IGDKEQGQEQTKDKKKGNGHVELSSFYDIGKEVKTEEDKTWNDAVTVLESPVEYTFSNDMD- 519
pber|PBANKA_1109000  YGDNDEDDDNLD------DNKINYDNA------------EVGK------------------------------------- 375
pfal|PF3D7_0509400   DQEDVDDLENMNN-----IDDINYNDDVDNMQHMNDDESQLNSIQ----------SRNENNKLMIKRESKKIEYNNNVND 450
etht|ETH2_0728100    G--P-----P----------GSSQGGPPKGGPQEGTE------------------------------------------- 361
sneu|SN3_00201460    G--S-----A----------GAS----------AAVD------------------------------------------- 410
tgon|TGME49_244880   E--------------------GR----------KKSD------------------------------------------- 373
gnip|GNI_013220      D--D-----S-------------IGTVRDARNVA---------------------------------------------- 284
```

```
chot|ChTU502y2012_38 S--D-----S----------GSESGKEPDSYNSGDSDNQEEGSIS----------------------------------- 255
bmic|BMR1_03g03880   ----------------------------KHYN------------------------------------------------ 238
tequ|BEWA_039100     ---------------------------QGKYGEKDKE--TERVI------------------------------------ 281
cvel|Cvel_8831       DADDEDALRRADEEEEEDEDEGEEGEEEEG-QEGGKS----------------------GEKPALPKSSTK--------- 370
vbra|Vbra_21242      PRWM---------------------------------------------------------ELYLPRAVIK--------- 303
tetr|Q23DD6          SSS----------------------------------------------------------------------------- 340
hsap|ENSG00000068654 ------------------------------------------------------------------PAMVH--------- 232
scer|YOR341W         NNRV---------------------------------------------------------KGFIRQDMIK--------- 276

htar|Htart_000206800 --TFITDGV-RDGNNDIIEE------------DENEDEN------EMLFD-IK-ENDGVDENITSSFSDVQ-----ASSI 571
pber|PBANKA_1109000  -------GNKR--GKEIKSE-------------KRKNLNNLENGVNQFLDGVY--NKKGGDEEDKLFKDAVASYKR-KAS 430
pfal|PF3D7_0509400   ESIKIEEDNNEDDDKQMDRESSYPHNNNNNNNDNNENYNKLSGGYENSFNEISTDNIRNDKNKNSTFADMVNHYNRTQAP 530
etht|ETH2_0728100    ----------------------------------------GA------------------PGARVQPDEGCLKYSR--TG 381
sneu|SN3_00201460    ----------------------------------------LE------------------DDAKDLFNERCLTYGK--SG 430
tgon|TGME49_244880   ----------------------------------------AK------------------DDAAALFDERCLTYGK--SG 393
gnip|GNI_013220      ------------------------------------------------------------NIDDEEIAKDHQTLGR--RG 302
chot|ChTU502y2012_38 ------SSQQS----------------NESKKRKKHAETKNKSS-AKLFKRSY------YDDFSMNIPQEAKSHTK--QG 304
bmic|BMR1_03g03880   -------------------------------------------------------------------------------R 239
tequ|BEWA_039100     ----------------------------------------------NMLN----------EEFESP---MLKGLNA--TG 300
cvel|Cvel_8831       ----AVPRVFF-----------------------------GAV--S---GDVA------RETEEEYRVDSAPNRRPGRGY 406
vbra|Vbra_21242      ----ALDDPN------------------------------VDV--SDL-MMRY------KE-AMEYMVKDIAKENRR--- 336
tetr|Q23DD6          ----------------------------------------QKK--H---QDDY------TSDDDQSESGHSKAEKAN--- 366
hsap|ENSG00000068654 ----R-----------------------------------TAG--Q---KDSE------PLGIEEAQIG----------- 251
scer|YOR341W         ----KQKQAKK-----------------------------LDG--S---NEAS------ANDEESFDVGRNPTTRPK--- 309

htar|Htart_000206800 LEKKELHMHSFQVINIMRKIFSK-KNEEFLKLVYPL----------------TKGRKPEAFFLYDVGICASRFRCEKTK- 633
pber|PBANKA_1109000  NEKCTVRLFSFQIIDLLKKIFVQ-DNKEIINLLYPF----------------TKKDGYQVFFLYYMGISSNRFRTQSRG- 492
pfal|PF3D7_0509400   KEKCTIRLFSFQVIDILKKIFEN-NNKDIINLLYPF----------------TKRDGYKKFFLYDMGVSGNRFRSQSKG- 592
etht|ETH2_0728100    KAIGSVELQGFQLYPLLQSLFSHDEDRLLLAYLFPM----------------TVRLGPSVFFQQVVAVAANRFRPPPAAA 445
sneu|SN3_00201460    QAIGKMQLHAFQLRPLMERLFSHRLDRLLLQCLLPV----------------STRLASSCFFISTLPVPASNFRPPAA-G 493
tgon|TGME49_244880   RAIGKLHLHAFQLMPMLQNVFKNSVDRRLLHCLFPM----------------SIQLSSSCFFISSLPVSANKFRPPLAGG 457
gnip|GNI_013220      RSMGAVNLQAYQIYPIMRDLYNS-PDRPLLHFLIPA----------------TAKHGARFFFMNVIPVSSNKFRPKAKSL 365
chot|ChTU502y2012_38 RGMITQHFQAFQIVPIIKNIWEN--NRKIFEFIFPI----------------CKSLGWEAFFMFTVPVSANKFRPLGMAV 366
bmic|BMR1_03g03880   SQEDKSHVTAFELIPFIQKLFEA--QSKILKHLFPF----------------TIMNGHGSFFMYNMCVTPNKFRAYLPIE 301
tequ|BEWA_039100     KSINMMHLQAFHLVPALNKLFTE--NPGFLGNIFPQ----------------SRFHGWRIFIMDCMGVSPNRFRPPLVTS 362
cvel|Cvel_8831       RVVTKEAVLPSDLLKILEIAWEE--DSELIERLFPM----------------TRTAGIGAFFMSAISVPSNLARPLKPSL 468
vbra|Vbra_21242      --YGGQQLAAMDLYPILQKVWNN--GAEVLEFVFPV----------------TRRLKHEAFFMDVMAVSGNKYRPLVRLS 396
tetr|Q23DD6          --ANQKYLDPLEVKELMRRFWDE--ERKLLNIIYGTIEVTPQTLKNSSKGFMIRRNDYNMFFIEVIPVTPNRFRPENKLG 442
hsap|ENSG00000068654 ---KRGYLTPTSAREHLSALWKN--EGFFLNYLFSGMDDD----------GMESRFNPSVFFLDFLVVPPSRYRPVSRLG 316
scer|YOR341W         --TGSTYILSTEVKNILDTVFRK--EQCVLQYVFHSRPNL----------S-RKLVKADSFFMDVLVVPPTRFRLPSKLG 374

htar|Htart_000206800 ----------------------TFVKTLAINRLSGTNKALKLCMQIEPGMDVAQILKGGTEQLMNYQQLFKNCNLGSTM- 690
pber|PBANKA_1109000  ----------------------IHKRTKITNYICKLNSFVKLCINSKKNIDFDYLIEYNKNELNLYNEMFLLFSLRIRP- 549
pfal|PF3D7_0509400   ----------------------IHVRTKITNTLCRFNNFIKLCINAKKKVDFDYLLEHNKKDLKLYKDMFSVFSLKMKY- 649
etht|ETH2_0728100    AAKGF------GGREAAAGRQLLHPRTQQLQDILKLNEKLKFALNVLRHPDPLGALKDPS-QWA---ALFMGAPEQQQQ- 514
sneu|SN3_00201460    AAFQR---------GGGRSMPVLHPRTATLLDLLKINERVLLAVNVMKHPDPIGALEDPS-RLM---ELFAGQPVGSVA- 559
tgon|TGME49_244880   AAFGR---------EGGGGMPLLHPRTATLLELLKVNEKVQFALAIMRHPDPTGALENPE-SLA---ELFGCAVGGKAE- 523
gnip|GNI_013220      -GFGS---SADAGAKADEEGGILHPRSAALLEILQENAKIGFALGALRYFDPEAHFAKDEGSLD---AAFSM-------- 430
chot|ChTU502y2012_38 -G---------------RNISLLHPRTSGLLEILTANDRLKFYVDLQR------KENTNESSIE---GEESS-------- 413
bmic|BMR1_03g03880   -G---------------KNKVSLHSRTASLITIVKTVNDLNNLLKSEN------LGT---GV------------------ 338
tequ|BEWA_039100     -D---------------K-QIVLHARSSALLEIVIANEFLKILFRVKN------HPNIQEAFKD---EEYIS-------- 408
cvel|Cvel_8831       TSSG---------------RVILHQRTLALLQVLAAAKSVDALLVGKGGDSKVAELRGLS-VS----EMLKSKSSRKARS 528
vbra|Vbra_21242      NKSGGGGDEFEREGHGGRVRHMLHPRSVALQRILELSLKIEDHWDWAFE-LIERHLRYLV-QV-------------KHFG 461
tetr|Q23DD6          EQTFL------------------HGHTVVLTKILQFNVELRRMIVLQ------------R-Q---------------SLS 476
hsap|ENSG00000068654 DQMFT------------------NGQTVNLQAVMKDVVLIRKLLALM------------A-QE-------------QKLP 352
scer|YOR341W         EEVHE------------------NSQNQLLSKVLTTSLLIRDLNDDL------------S-KL-------------Q--- 407

htar|Htart_000206800 -----T---------------------------------EREIEYRTFDEEEQLKLFSKHIDCI--CRNKGVFINMLINV 730
pber|PBANKA_1109000  -----R---------------------------------AVS-SSIDFNIEIAKSDFLRCYSLI--YNNKIAYLNILFSN 588
pfal|PF3D7_0509400   -----K---------------------------------FNN-NIMDDDTDITKMDFLKYYTLV--YNNKSAYINILFST 688
etht|ETH2_0728100    -----QQQ------------------------------QQQQQQLRLAPEQ---QQLLQEFV-EQHAVGGAKWLSTYALQ 555
sneu|SN3_00201460    -----G------------------------------TGSKQSLSAVLTVQE---QQRVKEFV-MVHAAGNATWLPTYSAQ 600
tgon|TGME49_244880   -----TEPEQEPARKKKLAAEPGSDDAPAA----KVSAAKSAVASILSPEE---QEKLKEFV-LVHAAGNAAWLTSYAVQ 590
gnip|GNI_013220      ---------------------------------------KDKDGVKKAIED---------H------KGNYSLLHQYCRQ 456
chot|ChTU502y2012_38 ---------------------------------------KTGKGAKTIKE---------EI------EVNSNNLISYVSS 439
bmic|BMR1_03g03880   ---------------------------------------AT-ISSNVNKLQ-----A----------AL------NALVD 357
tequ|BEWA_039100     ---------------------------------------KL-LGYNVNETQ---LRQFEEYVCGSGCDFNS-SIYNHTLN 444
cvel|Cvel_8831       VLGGRVQKEKEKEKEGGKETGEGAEEI-------EET----G--------TTSRIVLEGIRSLD-PGF--LQNGKVAMRQ 586
vbra|Vbra_21242      LEDGDEQ-PKKTAKKGKKAAAAGAAAAAAERPTVDVSKDEEREVAKLAAEERRRQPLNVTYQLM-EGAVI--TLDEDIKQ 537
tetr|Q23DD6          EE---------K-----------------KKKLIDQLDSRFQNQLQLKQNQNFQ-------QTI-NNIVKLNDVITKWLD 522
hsap|ENSG00000068654 EE---------V-----------------ATPTTDE-----EKDSLIAIDRSFL-------STL-PGQSLIDKLYNIWIR 393
scer|YOR341W         --------------------------------------------------KDKV-------SLE-DRRVIFSRLMNAFVT 429

htar|Htart_000206800 MQRIMNSFYKTSSYMLLMGMSFKNISLKDVLDKKEGVIRKHIMGKRVNHCARTVISPDTFIDTNQIGIPKSFALKLTVPE 810
pber|PBANKA_1109000  LQLTINTFLDSTLIDKRLQKNMDNISIKDILDKKEGILRKNIMGKRVNNCARTVISPDTFIETNQIGVPIEFAKTLTIDE 668
pfal|PF3D7_0509400   LQLGVNTFYDSTLTEKVNNKHLKNISIRQILDKKEGILRKNIMGKRVNNCARTVISPDTFIETNQIGMPIEFAKVLTIDE 768
etht|ETH2_0728100    LQLKLNEMTDSTKA-E--KPQAAPPGVRQWMEKKAGAIRQRMQGKRVNYAARTVLAPDGLLAPNEVSVPLDFAMKLSVPE 632
sneu|SN3_00201460    LQRKVNEIVDQTKA-E--NPATASPGIRQWLEKKEGAIRQKMMGKRVNFAARTVLAPDALIATNEIGVPLEFAMKLYIPE 677
tgon|TGME49_244880   LQQKVNEIVDKTKA-E--KPLTAPAGIRQWMERKAGTIRQKLMGKRVNYAARTVLAPDALIATNEVGVPLDFAMKLSIPE 667
gnip|GNI_013220      TQEKINAYIDSTKT----NTRNPPPAIRQWMEKKSGAIRQKMMGKRVNFAARTTIAPDAQINTNEIGIPIPFAMKLSIAE 532
chot|ChTU502y2012_38 LQEKVNMYLDQSKS-F--KPSSAPPGIRQWLEKKAGVIRQKMMGKRVNYSARTVIGPDPYLDTNEIGVPLMFALNLTIPE 516
bmic|BMR1_03g03880   STSTATNSAGSVKKDS--FLATNRPGIKQLIEKKSGLLRSNMMGKRVNYSARSVIAPDCFLDTNEIGVPLRIASSLDFPE 435
tequ|BEWA_039100     LQRRLSMYMDSGKQGT--STLAQRPGIKQSMEHKSGTVRQNMLGKRVNYSARTVIAPDCFMDTNQMGMPLKFAMELTIPE 522
cvel|Cvel_8831       LQQAINGYLDKTKAA---DPSSTENGVRQLIEKKQGIFRMKMMGKRVNFSARSVISPDLNIDPNEIGVPTVIAKRLHFEE 663
vbra|Vbra_21242      MQMQVIAYLDKNRMP-ATKRDDYPPGVRQGLEAKAGLMRMKMMGKRVNFAARSVIGPDPYIEPNEIGVPEAFAMVLTLPE 616
tetr|Q23DD6          IQEAVNCFLDSSKATK--TQDREGTGIRQLLEKKEGLFRMKMMGKRVNYAARSVISPDPNLNTNEVGVPLFVAKKLTFPE 600
hsap|ENSG00000068654 LQSHVNIVFDSEMDK---LMMDKYPGIRQILEKKEGLFRKHMMGKRVDYAARSVICPDMYINTNEIGIPMVFATKLTYPQ 470
scer|YOR341W         IQNDVNAFIDSTKAQGRTSGKVPIPGVKQALEKKEGLFRKHMMGKRVNYAARSVISPDPNIETNEIGVPPVFAVKLTYPE 509

htar|Htart_000206800 YVTKYNLNYIRRLLLNGPNIYPGALYVTNEKNKDIKISPDMNVRKQLIQKLHHL-------------------K-HDSDN 870
pber|PBANKA_1109000  HITENNFEYIKKLIENGPDIYPGALSYKDSQGRVFKLSHSYENRMKLINDLTNL-------------------LRREKKE 729
pfal|PF3D7_0509400   YITQNNFTYIKKLIENGPNIYPGALSYRDSNGRVFKLSHKYEDRLKVIEKIEKL-------------------N-FQTEN 828
etht|ETH2_0728100    AATSFNAFALSQMVINGPFKHPGALALEDDQGNFYNLEFMSA--AARAGKAAKLLSAAAAG--------------AAEGR 696
sneu|SN3_00201460    PVTPRNAHLLAAMVINGPFRHPGAVSVRDAAGNVFVLEYLSP--AARQAKARQILAAIQPSAARGLSASSRTGGGAGAES 755
tgon|TGME49_244880   KVTPRNVHVLSEMVINGPHKHPGALAIMDDAGNLFNLEFLSP--ATRRAKAHQLLLSASASASA-LS--GSSADMPTTNG 742
gnip|GNI_013220      HAVPHNVDLLCKLIMNGSKTYPGCNMIIEPSGRRLDLTRRELTESQKRMQCKLLRNHVQHQMHS-------AQNSHDLTK 605
chot|ChTU502y2012_38 RVGTHNVSLMANLVENGPNLYPGANKLLLG-GILYDLSRLDQ--NQRKAKARLLLSSV----LS-------ASNPFLGNH 582
bmic|BMR1_03g03880   RVTRHNIKFLMRLVENGPYRYPGANYIRQGKALF-FLGGLSD--MARKAKAK--LLSI----NT-------------PDR 493
tequ|BEWA_039100     HVTKYNVNFLRKLVLNGSKIYPGANFLRDSNGRLYSLSTLSY--SERVAKAKLLLTGI----TE-------------GNS 583
cvel|Cvel_8831       KVTSENFKWLRSLVIKGPRFWPGAIYVIEQEGRKKSLEFLSS--AERKKIARQVLPAVPRPEADMEGGVAVMRRRDKGLR 741
vbra|Vbra_21242      MVTPFNYRWLHKLVMNGPQKYPGANMVQDSRGQAVTLVRMSY--SARRGIAGKLLLKGSSDR--------------AAEA 680
tetr|Q23DD6          YVNEYNAKHLRKLIINGPFQHPGANFIV-ENGNKISLEALNE--QQRIGLAKTLLNNS---------------------K 656
hsap|ENSG00000068654 PVTPWNVQELRQAVINGPNVHPGASMVINEDGSRTALSAVDM--TQREAVAKQLLTPATGAP--------------KPQG 534
scer|YOR341W         PVTAYNIAELRQAVINGPDKWPGATQIQNEDGSLVSLIGMSV--EQRKALANQLLTPSSNVS--------------THTL 573

htar|Htart_000206800 PLIVHRHLRDGDVIILNRQPTLHKFSLMGHFVKVF--------------------------------------------- 905
pber|PBANKA_1109000  TLILHRHARDGDIVIMNRQPTLHKFSIMAHFLKIF--------------------------------------------- 764
pfal|PF3D7_0509400   SYILHRHARDGDVVIMNRQPTLHKFSIMAHYLKIF--------------------------------------------- 863
etht|ETH2_0728100    SFRVYRHLRDGDLVLMNRQPSLHRLSLMGHFVKINKPFNSEFNFNSFLEKAGLKRKDGSYRQVTRA---LLDKRQQQ--Q 771
sneu|SN3_00201460    SFTVYRHVLDGDVVLMNRQPSLHRLSLMGHFVRISRGACSWYDVNDYLLRNGVQHKLVEKQSGSRLAGPRVAKKENREAG 835
tgon|TGME49_244880   SFTVYRHVLDGDAVLMNRQPSLHRVSLMGHFVKITRGVSSVYHINDYLVRNNLLHLLVERRAARTSRKTVKSDSGSEADE 822
gnip|GNI_013220      PYTVLRHLRDGDVVIMNRQPSLHRASMMAHFVRVL--------------------------------------------- 640
chot|ChTU502y2012_38 HPIVYRHLIDGDIVLMNRQPTLHRPSIMAHYIRIQ--------------------------------------------- 617
bmic|BMR1_03g03880   QTIVYRHLLQGDVVLMNRQPTLHRPGIMAHFVRVC--------------------------------------------- 528
tequ|BEWA_039100     PRIVYRHVLDGDVALMNRQPTLHKPGIMGHFIKVL--------------------------------------------- 618
cvel|Cvel_8831       YTVVMRHLIDGDVVLMNRQPSLHRPSIMAHMVKVM--------------------------------------------- 776
vbra|Vbra_21242      PTIVYRHLRDGDVVLMNRQPTLHKPGIMAHVVRVI--------------------------------------------- 715
tetr|Q23DD6          NKVVYRHLQSGDVLLFNRQPTLHKASLMSHIARVL--------------------------------------------- 691
hsap|ENSG00000068654 TKIVCRHVKNGDILLLNRQPTLHRPSIQAHRARIL--------------------------------------------- 569
scer|YOR341W         NKKVYRHIKNRDVVLMNRQPTLHKASMMGHKVRVL--------------------------------------------- 608

htar|Htart_000206800 -------------------------------------------------DKEETLRLNYVNCSSYNADFDGDEMNVHILQ 936
pber|PBANKA_1109000  -------------------------------------------------EKEKVFRLNYVNCSSYNADFDGDEMNLHLLQ 795
```

```
pfal|PF3D7_0509400   -------------------------------------------------EKEKVFRLNYVNCSSYNADFDGDEMNLHLLQ 894
etht|ETH2_0728100    QQQQQQ---------------------------QQGEPAAAAAAAKSDQAPERVFRINFVNCSSYNADFDGDEMNLHVPQ 824
sneu|SN3_00201460    KNGSHQDSGGRVSLIHYWRRFRKGAEGAESSPAQKSAATRKDADSADGFTKEDVFRLNFVNCSSYNADFDGDEMNMHLPQ 915
tgon|TGME49_244880   ASAQRRKPGKKKSLVHFSGLFKKKAKVAKLAEE---EHRTTHHDEEPGLTKENVFRLNFVNCSSYNADFDGDEMNMHLPQ 899
gnip|GNI_013220      -------------------------------------------------TGQKTFRLHYANCNSYNADFDGDEMNLHCAQ 671
chot|ChTU502y2012_38 -------------------------------------------------PKDNIMRLNYVNCGTYNADFDGDEMNLHLPQ 648
bmic|BMR1_03g03880   -------------------------------------------------LNQKTFRLNYVNCSTYNADFDGDEMNMHVPQ 559
tequ|BEWA_039100     -------------------------------------------------TNQKIFRLNYVNCSTYNADFDGDEMNLHLPQ 649
cvel|Cvel_8831       -------------------------------------------------PGMKTFRLHYTNCNTYNADFDGDEINMHFLQ 807
vbra|Vbra_21242      -------------------------------------------------KGEKVLRLHYVNCNTYNADFDGDEMNLHLPQ 746
tetr|Q23DD6          -------------------------------------------------PREQTIRMHYANCKSYNADFDGDEMNLHFLQ 722
hsap|ENSG00000068654 -------------------------------------------------PEEKVLRLHYANCKAYNADFDGDEMNAHFPQ 600
scer|YOR341W         -------------------------------------------------PNEKTLRLHYANTGAYNADFDGDEMNMHFPQ 639

htar|Htart_000206800 TQEARSEVSHLMNSDSLFTSFKDGSPLRGLTQDFILGAVHLTSLETFLTFDEYTNLLQTALNGLMAKTNCFHFGSSS--- 1013
pber|PBANKA_1109000  TPLARAEATHLMNSDFLFTSFKDGSPLRGLAQDFILGGLHLTSLETFLNYDEYCNLLQCSLNGLLRQKNTFFFSKAK--- 872
pfal|PF3D7_0509400   TPLARAEATHLMNCDFLFTSFKDGSPLRGLAQDFILGGLHLTSLETFLNYDEYCNLLQCSLNSLISQKNSFFIKKKN--- 971
etht|ETH2_0728100    DPVSRSEGRLILNADSQFTVPKNGDLVRGLIQDYCLAGVLLTSRDSFFDLQQFSTLVYAGLERYLGCHDTCV-------- 896
sneu|SN3_00201460    DLMARTEAAYLMNADCHFLVPKDGGPMRGLIQDFCLAGCMLTARDTFFTADMFHTLVYSGLSCYIDSRENTV-------- 987
tgon|TGME49_244880   DHLARAEAAYILNADCHFLVPKSGEPLRGLIQDLCLAGCLLTARDTFLDIHSFQTLVYSGLSCYLDSGPGRV-------- 971
gnip|GNI_013220      DPTARAEAALICNADCNYTVAKSGEPLRGLIQDHVLGGNFLTIRGTFLTRPQYFALVYVGVASFTDKGEKL--------- 742
chot|ChTU502y2012_38 SLFSRSEAKYIMDASRQYSVPKSGEPLRGLIQDSCIGGAFLTSKSTFLNREEYFHLLYTSLSFIFDKKSSALS------- 721
bmic|BMR1_03g03880   DHLARAEAEAILDADAQNSGPKDGNPIRGLIQDHCQSGAMLTHKDTFLTRTQFNDLAFEALQTAFRNNMYT--------- 630
tequ|BEWA_039100     DPLSQAEAQLIANADCQFVVPKNGQPIRGLIQDHCQGGALLTSKNTFLRKDEYFNLIYVSLDAFIS-CSDN--------- 719
cvel|Cvel_8831       SDQARAEGHILMDADEMYYTAKDGSPLRGLIQDHCLGGAFLTSRDTFLEKDEYEQLVYEGIYRFFHQGTMNTVQMWQPVY 887
vbra|Vbra_21242      DPISRAEAQLLVNADNQYTVPKSGDPIRGLIQDHIIGGTLMTSRGTMLSAHEYTNLVYAALQTFVNAGGNQTPLSQR--- 823
tetr|Q23DD6          SHLARAEGYHISLNDRQFINTTNGNPLRELIQDTIVSAVFLTHKEKFLQKEEFQELVYAATWNMFEKNNNST-------- 794
hsap|ENSG00000068654 SELGRAEAYVLACTDQQYLVPKDGQPLAGLIQDHMVSGASMTTRGCFFTREHYMELVYRGLTDK-----VG--------- 666
scer|YOR341W         NENARAEALNLANTDSQYLTPTSGSPVRGLIQDHISAGVWLTSKDSFFTREQYQQYIYGCIRPE-----DGH-------- 706

htar|Htart_000206800 ----SPKKNPSDNINEDKDKN---------------NNNSSKDKTV------------DENN----------R------- 1045
pber|PBANKA_1109000  ----SRNISGSSNTKDGN-----------------MNSGD---------------------------------------- 891
pfal|PF3D7_0509400   ----NNNNNNKKNTNRTNSIMKNTRNSQYNIIQNDLNNNDSKEDYLKKMSILKNQQSLDDNAKISHPVSNDEARNNSIYE 1047
etht|ETH2_0728100    ------------RITLDRSKE---LE----------EAFSVDLDLN-----CNLNSNLN--------------------- 925
sneu|SN3_00201460    ------------VLDRGRGQG---RL---------PLGP-------------RN-------------------------- 1004
tgon|TGME49_244880   ------------ILDRGRKAS---SR---------LHASEVSLPKS-----GKTDGNAEDEAG----------------- 1005
gnip|GNI_013220      ------------DLDNGKVVS-------------------------------------------------------SEF- 754
chot|ChTU502y2012_38 -------TSPMIYSSQGQVIF--GSNNRFSEVNSPLGKVKIDECIL------RNQSNLNSNVQ------------NSNFE 774
bmic|BMR1_03g03880   -----------IYPNDDG-EY--NDNCSFNTVYKDKNNISVDWDLW------RNMPKLY--------------------- 669
tequ|BEWA_039100     -----------IYLKDEE-DT-----------YRICENIKIDNELY------QITKRLK--------------------- 749
cvel|Cvel_8831       GQDQRQSGEGLLDLKEGKKNLARMRR--KNKG-SQGGEGNDDEDLL------GIDKG----------------------E 936
vbra|Vbra_21242      -------------------------------------------------------------------------------- 823
tetr|Q23DD6          -------------------------------------------------------------------------------- 794
hsap|ENSG00000068654 -------------------------------------------------------------------------------- 666
scer|YOR341W         -------------------------------------------------------------------------------- 706

htar|Htart_000206800 ------RDNNKGIKDNKDDESTLNEEPLKMTNASYLDPYAY-----------HINEMEFSIVLEKPAILYPVKRWTGKQV 1108
pber|PBANKA_1109000  --------------NDKSISNSFRGINAKISNYSYIDPYAS-----------NLNRTNFTIKTEEPTILFPKKLWTGKQL 946
pfal|PF3D7_0509400   PTNNLLKPQDIDILKEINHGEQFNGNVLKITNYNYLDPYAI-----------ILNRTSFTIITEEPAILYPKKLWTGKQL 1116
etht|ETH2_0728100    ---------------------------KKSKIISNFN---------PKNGKNGPSSTPEGIALLTPAVLWPRRLWTGKQV 969
sneu|SN3_00201460    --------------------------SRGESAVAGGDPYRRRLLAGNTSGERAKTGEGSRLVIVPPAIVWPQKLWTGKQV 1058
tgon|TGME49_244880   ------------------------GEEGRERFKAPGDPYRRQLIEQTC-------RSNCRLHVEPPAILWPKPMWTGKQV 1054
gnip|GNI_013220      ---------------------------------SAGDPYAR-----------ALGARNLRIVVEEPTIWKPEPLWTGKQV 790
chot|ChTU502y2012_38 ------------IE---FSLDSLDTCIAPKNIPKNDSSCKTDFKIHS---NIHNSHIFNNLILEPPTIIKPIHLWTGKQV 836
bmic|BMR1_03g03880   ---------------------------------K-RSEISS-----------LYDKLDISVIMDQPAIIYPVKLWTGKQL 704
tequ|BEWA_039100     ---------------------------------VMSDPFSR-----------KFPNHGREIYIDPPAIVYPVPLWTGKQV 785
cvel|Cvel_8831       DSDDILETEELNIFGKKL--GAVDQVGSVRHCIRRGDPMDEI----HRTTPNKEMPEKLRVRTEPPAIVWPRPLYTGKQV 1010
vbra|Vbra_21242      ----------------------VDKKPTARHSVV---------------ERRMFIHRNKRILVEPPAILKPKRVWTGKQV 866
tetr|Q23DD6          -------------------------------------------------------TKPYKIFLVPPAIQKPKPLWTGKQL 819
hsap|ENSG00000068654 -----------------------------------------------------------RVKLLSPSIIKPFPLWTGKQV 687
scer|YOR341W         -------------------------------------------------------TTRSKIVTLPPTIFKPYPLWTGKQI 731

htar|Htart_000206800 ITSILKTVLDKVWQFTIGENDEQEEVEMTTQMNSETNNGMNNQMNNRMNNQMKNQRNDNIKDFGLNYIGKAFTPPTLWAF 1188
pber|PBANKA_1109000  ISSILKTIINELAIETYNKHGDNS------------------F---------------MENFKGINYVAKAKTDPELWSF 993
pfal|PF3D7_0509400   ITSILKTVIDKVAIETYNKYNDNE------------------F---------------MNTYKGINYVAKAKTSPDLWSF 1163
etht|ETH2_0728100    ITTILKTLVDGFARTRLRHVTAAAAAPPRTSSSS---------------SSSKRLEEALKGYKGINLEAKAKTPGDAWGG 1034
sneu|SN3_00201460    ITCVLKTLVDGIAAKRLAQVEIKRGDGNN--DAV---------------DERGRRQVSAAAYRGINLTSKAKTPGDAWGG 1121
tgon|TGME49_244880   ITCILKTLVDGMAASRFSQCRRSRQGAQ----GL---------------GSKPTDEDALNRYEGVNLTGKAKTPGDAWGG 1115
gnip|GNI_013220      INSILKTLIDSVAVLEYGRKAD-----------------------------GTANQAFLKRYRGINLVSKAKTPGDAWMG 841
chot|ChTU502y2012_38 FTSLLKTLLYNLVS----TE--------------------------------------PSVYAGINLISKSRTPGDSWNG 874
bmic|BMR1_03g03880   ITCIFKSLIDSIARYRLGD----------------------------------------IGYRGINFTGNSKIPD----- 739
tequ|BEWA_039100     ITSILKTLVDGMSTDIRGGV--------------------------------------SKGPRGINLVSKSKTPGDAWGG 827
cvel|Cvel_8831       FTSVLKNVVHRCIEARLPEGE---------------------------------------KFGGIDYDGKSKTPGTAWGG 1051
vbra|Vbra_21242      ITTILKTLVDQFATHSLGHTKG------------------------------------FRNYRGLNFNGRSKLPGDVWNG 910
tetr|Q23DD6          ITNIIKIVVQQNQKKD--------------------------------------------YEQGVYMEMKAKLT------ 849
hsap|ENSG00000068654 VSTLLINIIPEDHI-------------------------------------------------PLNLSGKAKITGKAWVK 718
scer|YOR341W         ITTVLLNVTPPDMP-------------------------------------------------GINLISKNKIKNEYWGK 762

htar|Htart_000206800 D-----------QIKESEVIIKHSELLQGIFDKSQFGVK-TYSVIHLVQEIYGSKVAAVLLDAFARVFINFLQI-RGSSI 1255
pber|PBANKA_1109000  D-----------PIKECEIIIKNSELLQGVLDKLHFGAS-SNSFVHLCYELFGPKSAAVMLDCFGRLFISFLQL-RGTSL 1060
pfal|PF3D7_0509400   D-----------PLKENEVIIKNSELLQGVLDKLHFGAS-SNSLVHLCHELFGPKTSAVMLDCFGRLFINFLQL-RGTSL 1230
etht|ETH2_0728100    V--------EDGDTEESTVVIKESELLQGVFDKNQFGSS-SGGLLHCVYLLLGSTAASALLQSLGALFSCFLKL-HGATT 1104
sneu|SN3_00201460    V--------EDGDTEEERVIIQESELLQGVFDKAQFGSN-AGGLLHCVFILLGSAAAGLLLQSLAALFCAFLKL-RGATA 1191
tgon|TGME49_244880   A--------EDGDKEEEVVIIRNSELLQGVFDKAQFGPT-AGGLVHCVFLLFGGAAAGLLLQSLAALFCAFLKL-RSATT 1185
gnip|GNI_013220      R--------LGTDKEEQIVIIRNSELVQGVFDKAQIGAA-SYGLVHLCYELFGPRVAGMLLSVFARVFSSYLQI-RGFSC 911
chot|ChTU502y2012_38 K--------EDGENEEAVVIIRNSELLQGVIDKNQIGSN-SYCLVHLCTELVGPLIGGKLLSAIFNLSQVYLQM-RGFTC 944
bmic|BMR1_03g03880   ------------NGDDTSIIMQNSELLMGVLDKSQFGAT-PFSLCHVINQLLGHRACGALLSSFARLFTTFLRS-YGTTC 805
tequ|BEWA_039100     S--------NDGNKEESTIIIQNSELLQGVLDKSQFGAS-SYGLTHLIFELLGPRICGMLLNSFSYLFTSFLQM-HGVTC 897
cvel|Cvel_8831       K--------DEGPTDEGEVVIRESELLGGVIDKSQIGAS-SYGVAHQIFELGGPRTAGLFLAAVGRCFTAYLAN-RGFSC 1121
vbra|Vbra_21242      V--------LDGDDEEADCVIRESEMLQGLMDKSQFGGA-KHGFIHLLFDLFGPKMAGLVLSAFGRCFTCFLQH-RGFSC 980
tetr|Q23DD6          --ADELTGIY---KDDTVVIIRDNELLQGVLDKNQFGAGATYGLMHAFYELYGASLTGKLFTALAKLLSAHLQM-HGFTC 923
hsap|ENSG00000068654 ETPRSVPGFNPDSMCESQVIIREGELLCGVLDKAHYGSS-AYGLVHCCYEIYGGETSGKVLTCLARLFTAYLQLYRGFTL 797
scer|YOR341W         GS------------LENEVLFKDGALLCGILDKSQYGAS-KYGIVHSLHEVYGPEVAAKVLSVLGRLFTNYITA-TAFTC 828

htar|Htart_000206800 SMFDFILKENVKEIKRNIKKQISFTGFYMQNYVLEKMAENVSINWADHM--LNEMKDCNAEHT-SLTK----------LK 1322
pber|PBANKA_1109000  SLYDFILKKKAKDEKKKIKKRISFTGFYLQNLFVYTIGKALNADMN-----KMDEYSNK--------------------K 1115
pfal|PF3D7_0509400   SLYDFILNKNAKKEKSLIKKRISFTGFYLQNLFAYSIANSVNADIT-----EMNSYTKK--------------------K 1285
etht|ETH2_0728100    SPADFVLRTDGLQQREELVQQIVHAGTFLQEHFVFRVCGQLG--AAAAA--ADEAAHQLQRRLAAYKQQQQQTPAARAAV 1180
sneu|SN3_00201460    CPGDFVMRRKGEFERKRLAERIVQAGTYLQEHFTARLNNLMG--WTEKE--QGSGSLGGTEECKVAVKS-----AACSPG 1262
tgon|TGME49_244880   SPADFVMRREGELQRQALVRQVIQAGTYLQEHFTARMNALMG--WGDDG--ADNPLLPTLSESGS--------------H 1247
gnip|GNI_013220      SSGDFQMTLEGEKARQRMIARCTAAGTLLQEQFIDSVYQHMH--WRENI--ENASGVQSKYVH----------------- 970
chot|ChTU502y2012_38 SISDMLLTPQAESERIELIKKYNHASVFIQEAFQYYIFKEIG--GKNDL--NNQTYFSEKFDI----------------- 1003
bmic|BMR1_03g03880   SIEDFMLNPKPELERVKLINSLTLNGFYLQSLFATALGHGTD--KEPKQ--SDRSFS-DDFIY----------------- 863
tequ|BEWA_039100     SPKDFILTSDAERLRIRILRRIKYTGLHIQQLFISAMREKFE--KKNNA--LSSGDAPHAVIL----------------- 956
cvel|Cvel_8831       GLGDLYMNQEGECKREALLRKVYRLNVSTLQGYVGGTLL----KEPIEG---------TS-------------------- 1168
vbra|Vbra_21242      NTGDLFLSPEGELERSLVMRDVHKASRFGLWLMVRQRLP----MWPLHVKLQQEVMEATLFKH----------------- 1039
tetr|Q23DD6          GMDDLLVSKESDEQRLQEIKAAHVDGVLACAEFVG-------------------MKD----------------------- 961
hsap|ENSG00000068654 GVEDILVKPKADVKRQRIIEESTHCGPQAVRAALN-------------------LPEAAS-------------------- 838
scer|YOR341W         GMDDLRLTAEGNKWRTDILKTSVDTGREAAAEVTN-------------------LDKDTP-------------------- 869

htar|Htart_000206800 SDKKGEKEDI-GEDEDEYDHAERENYHFPISNWNQQVYTF-LDMYKKREQIVRLMGREKIKKTGKQR-----S------- 1388
pber|PBANKA_1109000  ISNYEECQ-------------------------NIFSNKL-YDIYLKKSQIINKYERKLGKEINIGD-----KHMNTNLC 1164
pfal|PF3D7_0509400   MNTGNQRK-------------------------KLILNKM-YDLYEKQNELINNNFNEKENYLNK--------------- 1324
etht|ETH2_0728100    AAAGGGAADAAAAATAGAAAAARDPDELKAAA----AAAA-AEA-----------FQAGEGEAQQQRRKHALLQMAAAAA 1244
sneu|SN3_00201460    ISVGGEVQSHRRADDVGVV----------------------LRP-----------CRKDLGGENAQSKCAALLHMISNAG 1309
tgon|TGME49_244880   AEAGGETGSGVAQDEQ----------------------------------------------AKVASKDAALLHMAAASV 1281
gnip|GNI_013220      --VGKA------------------------------------------------------------------LSSALAAS 982
chot|ChTU502y2012_38 ---YKG------------------------------------------------------------------LNSNNEFE 1014
bmic|BMR1_03g03880   ---DNG------------------------------------------------------------------INGMDIM- 873
tequ|BEWA_039100     ---SAL------------------------------------------------------------------EHLIDEVG 967
cvel|Cvel_8831       -----------------------------------------------------VLV----------SPQA------AAVC 1179
vbra|Vbra_21242      ------RRRM--------IRALEREREEDGMNWMPWTKYLQWHRMGRPPVTPDLIYKGDP----EGKPTAA--AAAAGVG 1099
tetr|Q23DD6          -------------------------YEVP---------QG-WNLYNRPNFVL----NS------KGR------------- 983
```

```
hsap|ENSG00000068654 -------------------------YDEV---------RGKWQD------------------------------------ 848
scer|YOR341W         -------------------------ADDP---------ELLKRL------------------------------------ 879

htar|Htart_000206800 -------HSSNMF-----IESSDEDSYRVVMSEKEK--YLNNIATER-------ERIKR---EENGEKKDAVDSYNKLLR 1444
pber|PBANKA_1109000  LTKKNISKKFNLFND-E-EFNLKKDLYFITFLNKEIEDIFI---------------------------------QNRILN 1209
pfal|PF3D7_0509400   MEKRNIKKDSHVMKDEH-ILSISENSYLLALLNKELQNVIYHILNEVQFCDDDASLLNKEKNEKENEKENKI--ANTCIN 1401
etht|ETH2_0728100    AAAAGP-AEE-----GEAAAELGAAAAATVLAEKL---------SLARLADAAAELLDQQQQ------------------ 1291
sneu|SN3_00201460    DTHVEQ-FNKEMTKS---SSSLKGVPALEVHLERL---------RLARLADAVCTLLKIGGQTEEE--KGT----N---- 1366
tgon|TGME49_244880   APKDAT-RESEMNGDAVGQDELSAALEGALKLEKL---------NLARLSDAAAYALRVLGTSTTS--GDRTEPQS---- 1345
gnip|GNI_013220      KH-------Q-----------------AGTLATSY---------VKER---------YQQFVK----------------- 1003
chot|ChTU502y2012_38 KTKKAI-LEL-----------------RDLKKEKY---------SSKI---------SKEGQKN---------------- 1042
bmic|BMR1_03g03880   NMDKRP-RRE-----------------IDLLGTSE---------SVTK---------RRGGQGT---------------- 901
tequ|BEWA_039100     KVVNLP-F-T-----------------RDILLECK---------STDR---------AREIIGM---------------- 994
cvel|Cvel_8831       EAGKL---------------------------ESKK--------KTKNAA-------GKGGEGD----EDK--------- 1204
vbra|Vbra_21242      GGGKPT----PMD---------------TTAAAKRK--------KKRRTA-------DGGGEGK----GDGGEP------ 1135
tetr|Q23DD6          ----------------------------------------------------------------------FSSN------ 987
hsap|ENSG00000068654 -------------------------------------------------------------------------------- 848
scer|YOR341W         -------------------------------------------------------------------------------- 879

htar|Htart_000206800 HLRRGEFIKDVPADEYCKTVVQKVMKNSPKKKKRMIGQWGETYEVGRSQRIRHIPCNENHLYIDKFPSSDKTSIS-MYEH 1523
pber|PBANKA_1109000  GNVKNE---QSEETEKCEYSNLTHLNYIIK--KE-NYELFEKYVVGKTKLNS--------------------L------N 1257
pfal|PF3D7_0509400   YKHNNS---HVDDEQNCNSSLYSCVQYVLN--KD-LFNLYEKYLNGKNEEAN--------------------KHI-LYNN 1454
etht|ETH2_0728100    -----------------------------------------------------------------------------QHQ 1294
sneu|SN3_00201460    ---AVAFAKQL-TSK-----------------------------HGSAGSTQL--------QYKEIQKDAK------QFL 1399
tgon|TGME49_244880   ---PSAFSADTDEAA-----------------------------KGRKGRVKH--------EEDALEDSERSTTLQRKDA 1385
gnip|GNI_013220      -------------------------------------------------------------------------------- 1003
chot|ChTU502y2012_38 -------------------------------------------------------------------------------- 1042
bmic|BMR1_03g03880   -------------------------------------------------------------------------------- 901
tequ|BEWA_039100     -------------------------------------------------------------------------------- 994
cvel|Cvel_8831       -------------------------------------------------------------------------------- 1204
vbra|Vbra_21242      ------------------------------------------MAV------------------D---------------- 1139
tetr|Q23DD6          -------------------------------------------VL------------------K---------------- 990
hsap|ENSG00000068654 -------------------------------------------------------------------------------- 848
scer|YOR341W         -------------------------------------------------------------------------------- 879

htar|Htart_000206800 --------TDVENTIMDEMID-LMSGRLKERFLRGHINFTSNQIKELSNSIVQILKESQTNKRAKLYILYKLAQSETIKR 1594
pber|PBANKA_1109000  EQD-----ENVYSEFVNNIVN-KLYNSIKNPSFYKTTTFTSTQIVKVIEKIVNFLKEAKCNKKLKVFILFELFQNENITK 1331
pfal|PF3D7_0509400   CQI-----DERNNHFLNSVTN-KLYKALKEPTFFNSTSFTIKKITKVIEKVLTFLKAARCNKRLKIYIIFELFQNKNIMK 1528
etht|ETH2_0728100    QQRKQQQQQQVNPWLLLRRQQ--LPLAAAA--VRPTAAAAAQQLRLLLQNLKRMQ--NMPQQRRQLLLL--LSSSQLLQQ 1366
sneu|SN3_00201460    CKKSQAGAMCLSSWILTRLQR--LPLVAA--GKLITATSAAVQLQKFVLMLKTLSEREDETGRAWLARL--QETSPKFTQ 1473
tgon|TGME49_244880   RSSESAYPLSLNPWVLSRLLK--TPELLAASGALPTRKRMCEDLRRLIETLSQVQKTGSPTARKELLYF--LACSPSLQQ 1461
gnip|GNI_013220      ------------TEQINQFV------------------------------------------------------------ 1011
chot|ChTU502y2012_38 -----DNYLEINPSELVDFSD--------------NKNLTSTVLDVLIDTIKNFSENSNNKLEKK-LLL--LELVKSLKQ 1100
bmic|BMR1_03g03880   -------PYSMEERIIPEFSM--------------HSDY----------------------------------------- 919
tequ|BEWA_039100     -------LFHH----IAQVSQ--------------KDDL----LNLILKSLEKLSS------------------VPRIKQ 1027
cvel|Cvel_8831       -------------------------------------------------------------------------------- 1204
vbra|Vbra_21242      ------EPPEAEPPLLPHPSPPKTPKQTKKSKHPPPPAAAAKEEEE---K--EEEEEEPPKTKKKQKKV---T----GSK 1201
tetr|Q23DD6          ------RKPEV--------------------------------------------------------------------- 995
hsap|ENSG00000068654 -------------------------------------------------------------------------------- 848
scer|YOR341W         -------------------------------------------------------------------------------- 879

htar|Htart_000206800 YFPLLHKYISSIRNRVNDTDILRSASSNVKELLMEREDISET------------------------------IDDS---- 1640
pber|PBANKA_1109000  YFPSLIELANDFNTDISSEEIVENVINNIL---LKKDLKLETEQVSSHNV-----------------EKNKFND------ 1385
pfal|PF3D7_0509400   YFPSLVNYINSFHFDVSNEEIIKNVINNIY---FKEEDESPISSINNNNNNKVEDDCNQDIKIKGDQETNEFIDDEKIKT 1605
etht|ETH2_0728100    QLPRVAQLLQTQRL---------------LPLYMQPKPCSSSS-----SSSKVADP----------------SGGSFS-- 1408
sneu|SN3_00201460    QLLRLAQDRQGDSS---------------VRHQAEAHAAAPAP-----N-VEVSPA----------------VGD----- 1511
tgon|TGME49_244880   QLPHLAKVLGTERL---------------LPAHQRL--RTTLA-----K-ESQTET----------------LDG----- 1497
gnip|GNI_013220      ---TVDGLLNKDPQN-------VRLKNTTVPPHWPADPSKS--------------------------------------- 1042
chot|ChTU502y2012_38 QRLSIDGLFKVEENP-------DRFTHDASGILFPKDVCTNNS-------SKDSDP----------------SKD----- 1145
bmic|BMR1_03g03880   FTDSYSI-NKSNNLP-------Q--------------------------------------------------------- 934
tequ|BEWA_039100     LVLSYILMLKNGDSY-------K--------------------------------------------------------- 1043
cvel|Cvel_8831       --------------------------------------------------------------------VRLYLH------ 1210
vbra|Vbra_21242      TAPAAAQRQQDDDQP------------------MEP--QEEE------------DH-------QDEEDKEVWVG------ 1236
tetr|Q23DD6          -------------------------------------------------------------------------------- 995
hsap|ENSG00000068654 -------------------------------------------------------------------------------- 848
scer|YOR341W         -------------------------------------------------------------------------------- 879

htar|Htart_000206800 -----NEQSFGP-LPIILRKLIEMDKCVN-------------------NT--DVYGKGYNSNE----------TDFLHIH 1683
pber|PBANKA_1109000  -------SKWEKTNDVILKRYLHLYSRLI-------------------KVEKEKKGKINDST----SKFKHDEDE--DKI 1433
pfal|PF3D7_0509400   KRKCSQEKTIQEIKKYKLKKFLHFYAELH-------------------KKCNYEEGRGYDIDEQ--NDYNIDEKDFMNDY 1664
etht|ETH2_0728100    QLKYPPPNVV---VALPGGGAIEGSARLL-------------------KARLYHPSWLWQQQQQQQQS-------AAAAF 1459
sneu|SN3_00201460    VQLLPVPSTL---VCLPNGGFIQGDPSLL-------------------QTRLYHPSWLWNAGRRR----------AADVA 1559
tgon|TGME49_244880   SFHCPPPNEM---VSLPNGSAIQGDAALL-------------------QKRMYHPSWLWGGEEAGNPSAEGDSSDAAYAM 1555
gnip|GNI_013220      ------------------------------------------------AESQQNSPWLTEAQRSEFRDQGTA-------- 1066
chot|ChTU502y2012_38 --------------------F----YSNN-------------------DAILYDGSWLSIGKMEESI------------- 1169
bmic|BMR1_03g03880   ----------------------------V-------------------MVSKYYPSWLYQSPNRGINSSESTVTR----- 962
tequ|BEWA_039100     ----------------------------P-------------------QLLSSFPSWLHHDPTDVHTTREKEN------- 1069
cvel|Cvel_8831       -----------------GREFEHR-PAMLD--ARLIAAIRAEF-------------------GTQPLK----------ER 1241
vbra|Vbra_21242      -----------------GKEILELMPVYVSKTEGLFTAKERNRLRDALLRGYHMPVWKRKPPGSGLLD----------KH 1289
tetr|Q23DD6          --------------------------DYLSNENPILEQIQRKLVRD--------------PQLAEAQD----------NV 1025
hsap|ENSG00000068654 ----------------------------------------AHLGKD--------------QRDFNMID----------LK 864
scer|YOR341W         ----------------------------------------QEILRD--------------NNKSGILD----------AV 895

htar|Htart_000206800 G---FEGNDNV--------------SDDDNYENRDAVKNCFKHVMSQ-FELS---EGFVQNNSTGMDIDIFEKYKNFKN- 1741
pber|PBANKA_1109000  NETNLENSQNI--------------NENTIIGNPDIIKNSLISSLPS-FALN---EDITNLSY-NNRYQYYEKYLNIK-- 1492
pfal|PF3D7_0509400   NDNNNNNNDNLFRRSFVKNEMMSVNHTSDDVYDEKIIRNCLMSSLPS-FTLN---DEITNLSY-NGKYQYYEDYLCSKKK 1739
etht|ETH2_0728100    AAGSFESPARY----VLQ-------------TAA-----------------AA---AAAT----ST-------------- 1484
sneu|SN3_00201460    AAQVFEKIDRY----ILQ-------------AAE-----------------RA---SVTSG---NV-------------- 1585
tgon|TGME49_244880   AANIFEGTERY----VLK-------------GAR-----------------NA---GVGGGEG-GM-------------- 1583
gnip|GNI_013220      ------GFGDQ----LVAEEDL-I------QSKAGSSKTGLRKATEEHSAVMKAVDCDTSSSW-NV-------------- 1114
chot|ChTU502y2012_38 ------------------------------EDKF----------------------DDEENEF-NN-------------- 1182
bmic|BMR1_03g03880   CNNCFEEFNQN----SMTPKDVKL------GYSL----------------------SPRSA---EK-------------- 993
tequ|BEWA_039100     ------------------------------VYKL----------------------GGAQS---TC-------------- 1080
cvel|Cvel_8831       E-----T---------------------------------LKTL------------------------------------ 1247
vbra|Vbra_21242      E-----TP-----------------EEKAKEGKPGIVVPGLERLIT---------------------------------- 1313
tetr|Q23DD6          Y-----K------------------------------------------------------------------------- 1027
hsap|ENSG00000068654 F-----K------------------------------------------------------------------------- 866
scer|YOR341W         T-----S------------------------------------------------------------------------- 897

htar|Htart_000206800 --EVSYLPVCD-PLQVYKKFDFIITNFYSSNTNFFDKLMDSLLQPHLCKASSAVDGLI-KMESMLNKFKHNGFSAMIFA- 1816
pber|PBANKA_1109000  DEKYSRFKFYR-IDDVFHKLDFLINNFFSLKRLNFDGLLDSLFQPYLCRVSSGTNNLI-TMENLMNKFLDNGFSNMIFT- 1569
pfal|PF3D7_0509400   DEEDLNFKYYN-MDDVFYKMEYLIHNYFYLKRSDFDNLIDSLFQPYLCRVSSGTNNLI-NMNNLMNKFLNNGFSNMIFT- 1816
etht|ETH2_0728100    --KQKALLSPSILTQER--MRALLSLRFGGGLKELDEMLDSFFMGFMGKLASRSTDIM-NGKCFLYRFPRNGFASMIKT- 1558
sneu|SN3_00201460    --GIRTFSIPSKLTQQR--IGSLYRNHFLGRKEMFNEMLDAFFQSAIGKVASASTDIM-DGSSFLYRFPQNGFAGMIKS- 1659
tgon|TGME49_244880   --QLRTFLMPSLLTARR--IGALYTGHFRGREDTFNEMLDAFFQSAMGKVASKSTDIM-KGSYFLYRFPRNGFASMIKS- 1657
gnip|GNI_013220      --SRSLHLIPKQPDWRLG---------------D----VKKMYQWMLGQL-SKLKAMG-ASDKDTRQFLLQGFTTVPELA 1171
chot|ChTU502y2012_38 --HGDFFAYPNELTRRK--LESLIGNKLNTKKDSLLKIIDAISKIGLGDITSKLGNLL-KGSSSKFPFPYNGFSSMVLT- 1256
bmic|BMR1_03g03880   --NGFKFFYPGKLTHKNSQFEEIL------FNSKLKVLLDKFFNKCQGKLSSRIQDLM--DEASRILFPNNGFLRMIKT- 1062
tequ|BEWA_039100     --SSFHNLICDRFRGNE---------------QEFYRMFDRFFQNNIVGVSSDINSVV---DSTLLKFPENGFFGMVAT- 1139
cvel|Cvel_8831       ----------------------------------------EKFGKMTMGATSGENGQICGGPTLQVKFPHNGFHSMVLT- 1286
vbra|Vbra_21242      -------LGCELM-------EEHGFTRFRRN----KQRIDCMVQRMTQKKASQSQEIVSGGKYLHRKFPRNGFAGMVMT- 1374
tetr|Q23DD6          -------------------------------------------STMSQKGSKIL-N--TALGGLIKKFPFNNFTTMTNS- 1060
hsap|ENSG00000068654 -------------------------------------------EEVNHYSNEINKA--CMPFGLHRQFPENSLQMMVQS- 900
scer|YOR341W         -------------------------------------------SKVNAITSQVVSK--CVPDGTMKKFPCNSMQAMALS- 931

htar|Htart_000206800 -------GAK-------GSKVNYGMICGLLGQQYLEGKRVPRMKSGKTLPSFHKYDYGSRSCGLISDCFLEGLRPQEYFF 1882
pber|PBANKA_1109000  -------GAK-------GSKVNYSMICGMLDQQYLEGKRVPRMRSGKTLPSFHKYDYGARSCGLITDCFLEGLRPQEYFF 1635
pfal|PF3D7_0509400   -------GAK-------GSKVNYSMICGMLDQQYLEGKRVPRMRSGKTLPSFHRYDYGARSCGLITDCFLEGLRPQEYFF 1882
etht|ETH2_0728100    -------GAK-------GSNVNYAMICVFLGQQSLEGRRVQPMSSGKSLPAFAAADFGSLARGFVLSSFLTGLREEEFYF 1624
sneu|SN3_00201460    -------GSK-------GSNINFAMISVFLGQQSLEGRRVKNMPSDRSLPSFCPYDFGSRARGFITSSFLSGLKEEEYFF 1725
tgon|TGME49_244880   -------GSK-------GSNVNFAMISVFLGQQSLEGQRVKFMTSDRSLPAFLPFDFGSRARGFITSSFLSGLKEEEYFF 1723
gnip|GNI_013220      IYFPELAKVVGTRKMWAGERLEDRLV--VAAAARLTGTVPPPAPLGRL-------------------------------- 1217
chot|ChTU502y2012_38 -------GAK-------GSRVNYNMICVMLGQQELEGRRVPIMPSMKTLPSFATYDLGSRAGGLITDRYLDGLHAQEFFF 1322
```

```
bmic|BMR1_03g03880   -------GAK-------GSMVNFIMICGMLNQQSLEGNRVPIMVSGRTLPSFGVFDFGTRAGGFVTDRFITGLRPQEYFF 1128
tequ|BEWA_039100     -------GAK-------GSKVNFAMICSALSQQTLEGRRVPVMPSIRTLPSFAFGDFGSRAGGFISDRFLTGLRPQEYFF 1205
cvel|Cvel_8831       -------GAK-------GSSVNYGMITGFLGQQEIEGRRPTVMASMKSLPSFAEYDVGPRATGFIGDRYLTGLRPQEYFF 1352
vbra|Vbra_21242      -------GAK-------GSKVNYSMIGAMLSQQDLEGRRVPMMFTSKALPCFAKYDLGARNGGLIMDRFLTGLRPPEYFF 1440
tetr|Q23DD6          -------GAK-------GSKVNHTQVSALLGQQELEGRRVPVMATGRTLPCFLPYDPNPRAGGFISDRFLSGLRPAEFFF 1126
hsap|ENSG00000068654 -------GAK-------GSTVNTMQISCLLGQIELEGRRPPLMASGKSLPCFEPYEFTPRAGGFVTGRFLTGIKPPEFFF 966
scer|YOR341W         -------GAK-------GSNVNVSQIMCLLGQQALEGRRVPVMVSGKTLPSFKPYETDAMAGGYVKGRFYSGIKPQEYYF 997

htar|Htart_000206800 HCMSGREGLMDTSIKTATSGYLQRCLIKCMESVMCHYDGTVRDEDGSIIQFLYGEDGIDPSKTAYMN--NAKELLNNYNL 1960
pber|PBANKA_1109000  HCMSGREGLIDTAVKTAKSGYIQRCLIKAMESIVLHYDGTVRNEDNSIIQFLYGEDGIDPSKTAYLN--IPSDLINNYNI 1713
pfal|PF3D7_0509400   HCMSGREGLIDTAVKTAKSGYIQRCLIKCMESVILHYDGTVRNEDNSIIQFLYGEDGIDPSKTAYLD--LSKDLLHNYHL 1960
etht|ETH2_0728100    HCMAGREGLIDTAVKTAKSGYLQRSLVKSLEGLRLAYDGSVRDSDNSILQFVYADDGLDPAAAAALQ--HMQIYTENPFL 1702
sneu|SN3_00201460    HCMAGREGLVDTAVKTAKSGYLQRSVIKSMEALTVRYDGSVRDADDSVIQFVYGDDGRDPMHRSTFE--KITCLLENPDL 1803
tgon|TGME49_244880   HCMAGREGLVDTAVKTAKSGYLQRSVTKGLEALTVRYDGSVRDADDSIVQFVYGDDGRDPAHRLTFE--KIDMLLENPAI 1801
gnip|GNI_013220      -------------------------------------------------------------------------------- 1217
chot|ChTU502y2012_38 HCMSGREGLVDTAVKTARSGYLQRCILKGLESMIVNYDGTVRSEDDTIVQFIYGDDGIDVSKSSYLS--KLEDLVRNKTL 1400
bmic|BMR1_03g03880   HCMSGREGLIDTAVKTSKSGYLQRCIIKAMEAVIVHYDATVRDADGTIVQFKYGGDGIDLAKSPFLH--KPQHILHNINL 1206
tequ|BEWA_039100     HCMSGREGLVDTCVKTAKSGYLQRCVLKAMEDVIVCYDATVRGSDGSIIQFAYGEDGIDVSKSAYLD--RPRDIVANSHL 1283
cvel|Cvel_8831       HCMSGREGLVDTAVKTARSGYLQRCLVKGMEGCLVEYDGSVRDSDGSIVQFAYGEDGIDTTRTSYLE--KFDVALQHEPI 1430
vbra|Vbra_21242      HCMSGREGLVDTAVKTAKSGYLQRALMKTMEGITSLYDSTVRDADGLVLQFKYGEDGVDVMKSPYVD--KFEYALDNLAI 1518
tetr|Q23DD6          HCMAGREGLIDTAVKTSRSGYLQRCLMKHLEALKVAYDYTVRDADGSVVQFYYGEDSIDPNKVKYLE--KFRFLAENFTS 1204
hsap|ENSG00000068654 HCMAGREGLVDTAVKTSRSGYLQRCIIKHLEGLVVQYDLTVRDSDGSVVQFLYGEDGLDIPKTQFLQPKQFPFLASNYEV 1046
scer|YOR341W         HCMAGREGLIDTAVKTSRSGYLQRCLTKQLEGVHVSYDNSIRDADGTLVQFMYGGDAIDITKESHMT--QFEFCLDNYYA 1075

htar|Htart_000206800 TFPKYLKG--DMYNRIAKSVELFSEFKKRK---------------------------------------REEAEQAMREK 1999
pber|PBANKA_1109000  SFSKYLNY--GSDKLILNNQKLFIKNEN---------------------------------------------------- 1739
pfal|PF3D7_0509400   SFSKYLNN--NMHSKILNNQELFLKHEKD--------------------------------------------------- 1987
etht|ETH2_0728100    LRWLNRPPAGWVEEKEIKENKKENKKENKKEIKKE------IKKEIKKE-------IKKELKEKKQKETNTEQEEDSEEE 1769
sneu|SN3_00201460    LRCVSPVSPDIAAGNAAERKELF-QH------------------------------------------------------ 1828
tgon|TGME49_244880   LQRVHAPPAELSNGKQSANTKPG-AS------------------------------------------------------ 1826
gnip|GNI_013220      -------------------------------------------------------------------------------- 1217
chot|ChTU502y2012_38 LERDVK------FEESMGNKFLTNSF----------------KNVFKYE-------NRQNLFK-----TCLNQDILSDEK 1446
bmic|BMR1_03g03880   LPDKTL------DGRAVT----------------------------------------------------------IDHE 1222
tequ|BEWA_039100     IPQDEV------AKVNVE----------------------------------------------------------ASEK 1299
cvel|Cvel_8831       ERARFKLDKAEKNARVRTAVKKRLKYEVLGEVH---------------------------------QT------------ 1465
vbra|Vbra_21242      LQSRYSTDKAMSTPNVARSIRRAD--------------------DYSKQVARELRSIRQAVKERNQQRKAVEERQFDLGP 1578
tetr|Q23DD6          FTQKYDPYHLKDSHDFESVNRFLKK------------------------------------------------------- 1229
hsap|ENSG00000068654 IMKSQHLHEVLSRADPKKALHHFRAIKKWQSKHPNTLLRRGAFLSYSQKIQEAVKALKLESENRN-----------GRSP 1115
scer|YOR341W         LLKKYNPSALIEHLDVESAL------------------------KYSKKT------LK---------------------- 1103

htar|Htart_000206800 LIERIKMEMQRENIDDVNMDINWNTVMNWNADINFNMHMENIWGISEFSHLNKMHSQERDSNYDAGFFCEFNPYRYVGIV 2079
pber|PBANKA_1109000  -----------------------------------------------------------NKKNEDPLICQYNPYTYIGSI 1760
pfal|PF3D7_0509400   ---------------------------------------------------------NDTKDNEEPLISKYNPYTYIGSV 2010
etht|ETH2_0728100    AEMGLKEKENKRENKNKNKSED-----------------ENNYLPDK---------TLETFLKKMEAQPFNSPLSKLGAA 1823
sneu|SN3_00201460    --EGVKED------------------------------------------------------ETEDMEGPVSPLSHVGIT 1852
tgon|TGME49_244880   --EDLVADA----------TDD----------------------------------------AECLIQGCGSPLTCLGVT 1854
gnip|GNI_013220      -------------------------------------------------------------------------------- 1217
chot|ChTU502y2012_38 SFKKVKKSVEKDCFDNLNISDNN---------------KTNAIDVKN---------ISSTSLNEMTVNSLLPTILNGGSV 1502
bmic|BMR1_03g03880   TLV------------------------------------------------------------KM--LDLIPSLNSSGKL 1240
tequ|BEWA_039100     FME------------------------------------------------------------KM--RLFTS-------- 1309
cvel|Cvel_8831       --------------------------------------------------ATLSAKGHGKGTLMDPTTAAYPPHTEPTVV 1495
vbra|Vbra_21242      AIERIETT------------------------------FKDPWKEVDD-----PAPSNPEMSPPDPSAEKYHPMANVGST 1623
tetr|Q23DD6          --------------------------------------------------RKRQESSETEKKTCDTIMNNFLPGKHIGSV 1259
hsap|ENSG00000068654 GT----QE------------------------------MLRMWYELDE--ESRRKYQKKAAACPDPSLSVWRPDIYFASV 1159
scer|YOR341W         -------------------------------------------YRKKH----SKEPHYKQSVKYDPVLAKYNPAKYLGSV 1136

htar|Htart_000206800 SDKYEKDIKKQANGVFNRNSNFTYFNF------------------------------------------NKIPQNFVILT 2117
pber|PBANKA_1109000  SDNYNIKLNNIL-----KKKKFNLTNY---------------------------------------------NENFDSLS 1790
pfal|PF3D7_0509400   SDKFNNKLNNTL-----LWNDLNFPEY------------------------------------------ENVPMNFDTLS 2043
etht|ETH2_0728100    SYLFEQTLEEVVSAACARYERTRQWYSKQQQQQQQQ--------------------------------DVYRCGIGAAEL 1872
sneu|SN3_00201460    CPKFERDSVELISKVLKERRRVRSFHSNSSLDRTLRKTKVNAAKQKHNRLPSISAGSCSKHG----LEVDAEQESTRAEL 1928
tgon|TGME49_244880   AFQFENDMQLVVDKAVASYESLRAAYSRTRETRHKKK------RR-KESGSEVSGASAAR--------DARTTGLEKEEL 1919
gnip|GNI_013220      -------------------------------------------------------------------------------- 1217
chot|ChTU502y2012_38 SELFLNSLSETNIQKLTRISSDSKYYSDNNYSFKGKRKS-------RN---SLVNNK-----------YSAEEYESMLNM 1561
bmic|BMR1_03g03880   -----------------------------------------------------TAN--------------------LSNE 1247
tequ|BEWA_039100     -------------------------------------------------------N---------------------YQD 1313
cvel|Cvel_8831       SEKYHRAIREFAKKVGRQEKEKEGPTGEDADPGKKRG---------RKSDSSGNGGKKARGGGGDGDGKETTRLTDPESI 1566
vbra|Vbra_21242      SEKYREKIFEWIRGNPFLLKDAEAYIAQMGEEGLEEE------------DSKKKK-------ETKIKHVAQGKLMTPQEF 1684
tetr|Q23DD6          SERVYKNVKEYIKKEQQSADPYS-----------------------------------------NIFN-IKQDQLSPQKF 1297
hsap|ENSG00000068654 SETFETKVDDYSQEWAAQTE----------------------------------------------KS-YEKSELSLDRL 1192
scer|YOR341W         SENFQDKLESFLDKNSKLFK--------------------------------------------------SSDGVNEKKF 1166

htar|Htart_000206800 EFILRSKY-FNSLCAAGEPLGVLVAQSIGEPATQMTLNTFHLAG--T-ENVTMGIPRLKEILYNRV-SISKPIMYVPVKI 2192
pber|PBANKA_1109000  SSLLRSKY-FHSLCSPGESIGILVAQSFGEPATQMTLNTFHLAG--T-ENVTMGIPRLKEIFLTSK-LTSKPMIYVPIKL 1865
pfal|PF3D7_0509400   SSLLKSKY-YNSLCNPGESIGILVAQSFGEPATQMTLNTFHLAG--T-ENVTMGIPRLKEIFLTSK-LTSKPMIYVPIKL 2118
etht|ETH2_0728100    QQLLRVVY-HKSCMQPGEAVGVVAAQSVGEPATQMTLNTFHLAGHGA-ANVTMGIPRLRELLQLGA-KTKTPQIIIPIKG 1949
sneu|SN3_00201460    QTLLKLLY-HKCLAPPGEAVGCVAAQSIGEPATQMTLNTFHLAGHGA-ANVTMGIPRLRELLQQGG-ATKTPVIYVPLRS 2005
tgon|TGME49_244880   EKILRVIY-HKCCMPPGEAVGCVAAQSIGEPATQMTLNTFHLAGHGA-ANVTMGIPRLRELLQQGA-VTKTPILYIPLRT 1996
gnip|GNI_013220      -------------------------------------------------------------------------------- 1217
chot|ChTU502y2012_38 EKLLRLKY-MKTQISPGEAVGCLAAQSIGEPATQMTLNTFHLAGHGA-ANVTLGIPRLKELLQTGG-DSKTPYIFIPLLK 1638
bmic|BMR1_03g03880   ERRRLGHYLTNFSVASGEPVGCLAGQSIGEPATQMTLNTFHLAGHSA-ANVTLGIPRLIEILIHSG-STCKPVMAIPILG 1325
tequ|BEWA_039100     QRVLLVHY-KHSQCDAGDAVGCVAGQSIGEPATQMTLNTFHLAGHGA-ANVTLGIPRLIELLRTTG-DSSTPYFSATLLG 1390
cvel|Cvel_8831       EEFLRLRY-SRHLVAPGEAVGALAAQSIGEPSTQMTLNTFHLAGSGG-ANVTLGIPRLRELLQTTA-PASTPVMHLAIRK 1643
vbra|Vbra_21242      RVMMMLKY-HDGLLQPGENVGCVAAQSIGEPSTQMTLNTFHLAGSGGATNVTLGIPRLVEILQRASRKQKTPLAYVALRR 1763
tetr|Q23DD6          HMLFNVKY-LNSLIHPGDSVGCLAAQAIGEPSTQMTLNTFHLAGHGG-VNLTLGIPRLREILMTATDKLKTPVMLLNL-- 1373
hsap|ENSG00000068654 RTLLQLKW-QRSLCEPGEAVGLLAAQSIGEPSTQMTLNTFHFAGRGE-MNVTLGIPRLREILMVASANIKTPMMSVPV-- 1268
scer|YOR341W         RALMQLKY-MRSLINPGEAVGIIASQSVGEPSTQMTLNTFHFAGHGA-ANVTLGIPRLREIVMTASAAIKTPQMTLPI-- 1242

htar|Htart_000206800 ETRTSATKVIGTASNSTINTVAFTSSTYGKGQTMGAKGVLMDTNVEEQISKLSEKFLMVCKDIKVKDVIVGITIEEKVLV 2272
pber|PBANKA_1109000  EEKY-INNP---------------------------------NKIKNYITNIADKILSSYQSILLSEVIYGIGMDRKLIL 1911
pfal|PF3D7_0509400   QVDNLKNDA---------------------------------NKMKNYITSIADKILSSYKSIFLTDVIYGIGVDRKIIL 2165
etht|ETH2_0728100    LSSSGSSSSSSSSSSE--GRNGQ-----------------AAAAAAAAAAANAATALRGFKTIRMKDFVHAVGAEASVCY 2011
sneu|SN3_00201460    RTNLVDGD-----------DRVLT----------------SEISESAILAQNAATALRGFTTINLAACVHSVGVETKVCY 2058
tgon|TGME49_244880   AANRLQDSPNA--------DLEAR----------------EELDEGRVLVRNAGMALRSFTSFGLADCVHSVGVEANVCY 2052
gnip|GNI_013220      -------------------------------------------------------------------------------- 1217
chot|ChTU502y2012_38 DINLKKDKSSK-----------FA----------------DEENDNKDFINYAEKVLDCFKSIPLSDIIENVGVESSIVY 1691
bmic|BMR1_03g03880   NSAQEI-------------------------------------------SDNAKVAIRSLLTIKLSELINSIGIDSDL-I 1361
tequ|BEWA_039100     QDEAKI-------------------------------------------AKNAQLALNALRTVPLSDIVHSVGIETDV-Y 1426
cvel|Cvel_8831       RHGEKG--------------------------------------VQETV-RMAVLMCQMLTRLPLRNISRFIGARQVTIQ 1684
vbra|Vbra_21242      IPDWPD--------------------------------------FQRVFGERAGYRCRKVTYAHFM---KAVSFNRQLVT 1802
tetr|Q23DD6          ---SNP--------------------------------------EVAQNKEQVQNHARKLQKINLLELVNNIEVKEQKII 1412
hsap|ENSG00000068654 ---LNT--------------------------------------KKAL--KRVKSLKKQLTRVCLGEVLQKIDVQESFCM 1305
scer|YOR341W         ---WND--------------------------------------VSDE---QADTFCKSISKVLLSEVIDKVIVTETTGT 1278

htar|Htart_000206800 VDTSVQQHELLNAANTIMGIETNPREVHMDMNVGAFIDMDKTSVRSNMIFEGESRNEMQVMQYKQMNDNMYNKNENHNGY 2352
pber|PBANKA_1109000  RDKIENKEIHTI-----------------------------------------------------VLDNLGSKNNEENGF 1938
pfal|PF3D7_0509400   KENQNEQ-KHTI-----------------------------------------------------NLSDIIN-------- 2183
etht|ETH2_0728100    QG------------------------------------------------------------------------------ 2013
sneu|SN3_00201460    QSPYCLE------------------------------------------------------------------------- 2065
tgon|TGME49_244880   QPPNCPK------------------------------------------------------------------------- 2059
gnip|GNI_013220      -------------------------------------------------------------------------------- 1217
chot|ChTU502y2012_38 DEDG---------------------------------------------------------------------------- 1695
bmic|BMR1_03g03880   LLDG---------------------------------------------------------------------------- 1365
tequ|BEWA_039100     A------------------------------------------------------------------------------- 1427
cvel|Cvel_8831       E------------------------------------------------------------------------------- 1685
vbra|Vbra_21242      MM------------------------------------------------------------------------------ 1804
tetr|Q23DD6          VV------------------------------------------------------------------------------ 1414
hsap|ENSG00000068654 E------------------------------------------------------------------------------- 1306
scer|YOR341W         SN------------------------------------------------------------------------------ 1280

htar|Htart_000206800 SYEYGNKYGHESNTYPEYFNTKKLSNGKEETQEDGIQTSDELYKRCWNILDNITIEEEEEEEEEGGDKEEGGDKEEGGDK 2432
pber|PBANKA_1109000  DWNKVDICK------------------------------TTDYSKILQ-------------------------------- 1956
pfal|PF3D7_0509400   -MNDEELSG------------------------------DTQLAKINN-------------------------------- 2200
```

```
etht|ETH2_0728100    ---------CAAD------------------------------------------------------------------- 2017
sneu|SN3_00201460    --------GCPAFHLPPYTKS----------------------------------------------------------- 2078
tgon|TGME49_244880   --------GCPAFHLPPYVSR----------------------------------------------------------- 2072
gnip|GNI_013220      -------------------------------------------------------------------------------- 1217
chot|ChTU502y2012_38 -------------------------------------------------------------------------------- 1695
bmic|BMR1_03g03880   -------------------------------------------------------------------------------- 1365
tequ|BEWA_039100     -------------------------------------------------------------------------------- 1427
cvel|Cvel_8831       -------------------------------------------------------------------------------- 1685
vbra|Vbra_21242      -------------------------------------------------------------------------------- 1804
tetr|Q23DD6          -------------------------------------------------------------------------------- 1414
hsap|ENSG00000068654 -------------------------------------------------------------------------------- 1306
scer|YOR341W         -------------------------------------------------------------------------------- 1280

htar|Htart_000206800 EEYLFGTKASRKYKSMLCTGTHLKVQSKQEMRNIFCIQYENLFTCCKKNKHITVYSILMKSVNCILMAIFKKIKENLQKT 2512
pber|PBANKA_1109000  ----------------VIENTKLNFDLKKEWVHEIVIQFDNLNHFCKVNKHLSIPFILYKMVNIVLVSILNKIQSSISHH 2020
pfal|PF3D7_0509400   ----------------VLKNCNINFDMKKEWNYEIIIQFENLYHFCNINKHLTISFILYKVVNALLNSVLNKVHETFVFH 2264
etht|ETH2_0728100    ----------------GSAAAAAAAASGLQWQYEVSIQLEPLQHYCSVAQQFSPDDLLLLVLNKVIRPLLKKAKKLAVAA 2081
sneu|SN3_00201460    ----------------T-AASWTAPFSRLYWQYDVCIQMENLSHFVQVVPTYNQAEVCFAVLKKVIRTFLKKVSRLMVAS 2141
tgon|TGME49_244880   ----------------NNSAQSIAPSSRMYWQYEVCLQLENLAHFSQVVKSFTPAQICTLVLRKVIRPFLKKAYRLMVAA 2136
gnip|GNI_013220      -------------------------------------------------------------------------------- 1217
chot|ChTU502y2012_38 ------------------KFKSSHKKSAYCWNYEISIQFSDLEVFCQSLPHYNMSSLMNLTLSKCIKPFLRNVNNMYTIS 1757
bmic|BMR1_03g03880   ------------------YLGNADARNRIIWEYTATLQFENLKNFGKVNLGVSLDEILSSAKNALIA-FTLRVQYLISVT 1426
tequ|BEWA_039100     -----------------------RPDGSKVWEYTATVQFENLSFFKKVVGGFKTQNILKLTSNCLLNRFLRRVMGLMVVT 1484
cvel|Cvel_8831       ----------------------AKGRRRETVSTEIKIKLISFEILRQGIPGCTMAALGAFLHRHWTSELVKQVWIFVVES 1743
vbra|Vbra_21242      -------------------GEATHGLPQRWWYFRMRFHLHDIREIVSAMPFITYEQIEQMCYQKYLPRLLKIFKKMF--- 1862
tetr|Q23DD6          -------------------DNNILPFNERFRHYEIKINLEDLEAIHFA---------FQLNIDTINRRIEKVFLPNL--- 1463
hsap|ENSG00000068654 ------------------------EKQNKFQVYQLRFQFLPHAYYQQE---------KCLRPEDILRFMETRFFKLL--- 1350
scer|YOR341W         -------------------T----AGGNAARSYVIHMRFFDNNEYSEE---------YDVSKEELQNVISNQFIHLL--- 1325

htar|Htart_000206800 LPSMKDAHEGMFYDELEEAVTEIV----KI--SANGIDKSIEVMYSEKSKKKNEISL-TKDIKESFE-SSSTNGG--NDK 2582
pber|PBANKA_1109000  NIKNIHFINHEHYDELFDFISGKMSEEFSF--NHEKY------EYKND---EEEINAKLKYMSKNFE-GNKSGDE--ND- 2085
pfal|PF3D7_0509400   NLINMNSLNHEYYDELYDFISKKMSEEFNF--NMDKY------EYKDD---EHQINAKLKFMEKENE-RYDSKRGTNNNI 2332
etht|ETH2_0728100    VSQYELSDAAELQNDYQQMYRSFILKERFK--SQW-D-------RR----------GFVKDILRSGR-IDCETSGAPEGP 2140
sneu|SN3_00201460    VAHYGRAGSSAARNDMQSFFQQRVCQDNRL--NSW-H-------YR----------QKVKEILRTAR-VATGTAEAGEAP 2200
tgon|TGME49_244880   VAHHGRTDFAEMRSDLQAFFQNAICQDNKL--TQW-D-------HR----------SKVKEILRTAR-VGDSTTGEA--T 2193
gnip|GNI_013220      -------------------------------------------------------------------------------- 1217
chot|ChTU502y2012_38 SIRSSKFSNQD-NDETDEFVSKYVLGHNSD--RNIGI-------FK----------GALNSMIQKGN-FSFGGGGGGEGE 1816
bmic|BMR1_03g03880   VDMHNKYQTSPQMDFIQEFVNRYYFKETFG--KI----------------------DKLAERYKRKV-F----------- 1470
tequ|BEWA_039100     MDVNIPYELPDNTDYLEECWNTLVLQKQLI--KK----------------------DKQSLRIRKMV-LSSGSSG----K 1535
cvel|Cvel_8831       VADRVRKLA-DSGNLTQKFASDEVFEALRL-DRGGRECRHWISLIKKVVKHEK------KLTSRPSHMTARGVFGGNAHM 1815
vbra|Vbra_21242      ------------------------MVYFDITEEAWEGSKDWGLKGGKSKAEQ----------ARE---AEDAAGGGGDDI 1905
tetr|Q23DD6          ------------------------FQYINK--ELKRDQKSE----------L------------------------QTRV 1483
hsap|ENSG00000068654 ------------------------MESIKK--KNNKASAFRNVNTRRA---T------------------------QRDL 1377
scer|YOR341W         ------------------------EAAIVK--EIKKQKRTTGPDIGVAVPRL------------------------QTDV 1355

htar|Htart_000206800 YIQEEGREHGDETSDGADTLLLNSLGMGENTEEMDPEID---IDMDPEMEGVDDMEN---DFVN--------------YE 2642
pber|PBANKA_1109000  YERYNGS-LKVP----------KKYNEEN-DDNF-------------------SIDNKNEDTENEKNNKSDGNNDSDQND 2134
pfal|PF3D7_0509400   SGEYQNASHTIAS-HMEDDSPMKSYNDISNKENMSPYDDGKGKDGDRDQDDMSDIELKDNSEKNSNSDQASKNADSDTDE 2411
etht|ETH2_0728100    LG---PPSSSSSS----------SSA---ARGEMDEEREALKDAA----------------------------AAAAAAD 2176
sneu|SN3_00201460    TA---DSFVGGSV----------SRSVIERKDGADDEQEGQ--HRQ----------SRDAD--------AEGEKKNSADE 2247
tgon|TGME49_244880   DE----LLSSGAT----------VR-----AGNEDDRENGGGNKRA----------EADADEK-----EADGSDPASSAD 2239
gnip|GNI_013220      -------------------------------------------------------------------------------- 1217
chot|ChTU502y2012_38 LGQYEDDFDGGSR----------SDRKKEPK---SEDDSGNNTKK--------------------------------EEI 1851
bmic|BMR1_03g03880   -------------------------------------------------------------------------------- 1470
tequ|BEWA_039100     VA----------R----------SKDREEPV---DDEP------D--------------------------------VEI 1554
cvel|Cvel_8831       R---------------------RDEEDQEDEGEEEEDRE-----ER-------------------------------EEE 1838
vbra|Vbra_21242      DD-A------------------AADEMREPEGEEEGDSD-----LERDDEAVDPL-IGDDDEG--QDEGAPRDRDEDLYE 1958
tetr|Q23DD6          L---------------------KKK------ENESSGME-----T-------EGL-VSDFDKE-------------AA-S 1509
hsap|ENSG00000068654 D---------------------NAGELGRSRGEQEGDEE-----E-------EGH-IVDAEAE-------------EGDA 1410
scer|YOR341W         A---------------------NSS--SNSKRLEEDNDE-----E-------QSH------------------------- 1375

htar|Htart_000206800 DEEHKGIGTERSNKRAAFKN-----------------KNYNKSAI---SNTN--NNR-HIGDDGSEDDGDEDEEAEDGGG 2699
pber|PBANKA_1109000  GRESDEY---KSEQ----ED-----------------KEYDESED---AENDNDTDS-ILSQNGPDSDDDIKHES----- 2181
pfal|PF3D7_0509400   EEEDDEL---KSEKEKEEDE-----------------KEYDEEGE---SD----TNS-KIDSDH-KSDSDNKSDS----- 2457
etht|ETH2_0728100    DEDKEEK-------RNAKKG-----------------RDSDGEEQDEDVSEDSAES---EEEEGEEESGEEDEEQ----- 2224
sneu|SN3_00201460    DDEEEE------------RS---------------------EAEEEEDGSEDGKTRS-PDEADGVEGLWTSSQEK----- 2288
tgon|TGME49_244880   EEEKEE------------KG-----------------DDQDEGDDDEQQSEGGRASE-SEQSDGEQES-SKRQRS----- 2283
gnip|GNI_013220      -------------------------------------------------------------------------------- 1217
chot|ChTU502y2012_38 DDADNDM-------NNEMDG-----------------DDKDKNDDDDNSSVESSSST-SEEENGKDDSDEETE------- 1899
bmic|BMR1_03g03880   ---------------------------------------------------RC--------------------------- 1472
tequ|BEWA_039100     EEQ----------------E-----------------PEEDVQEEEDESETEC----------GSD-------------- 1577
cvel|Cvel_8831       DEEEFAR------------------VLEGVEEEMDDE-EMEEEDEEEGS--FQKS--KKKKKRKVRGKTEEDEDS----- 1890
vbra|Vbra_21242      DEEDIRKRKRRRKR-REEEGEEGEEGEEGSDLDIDEELMMDEGDEDEGEGEGDESDESGKDKDSEAEEQEEDEEA----- 2032
tetr|Q23DD6          DGEELKKAADALV----SEV-----------------EYGIFTETE---------------------------------- 1534
hsap|ENSG00000068654 DASDA-KRKEKQE----EEV-----------------DYESEEEEERE-----------------------GEEN----- 1440
scer|YOR341W         ---------KKTK----QAV-----------------SYDEPDEDEIE-------------------TMREAEKS----- 1401

htar|Htart_000206800 GGTTKGKNGDEREYEHDDNATSSEDG-T--------------------------------DEEKGE----VEEEKEEKEE 2742
pber|PBANKA_1109000  ------MNSSESDYNK------------------------------------------------------YDEKQKNKEK 2201
pfal|PF3D7_0509400   ------DNKSDSDHKSDSDHKSDSDHKSD-------------------------------SDHKSDSDNKNDSDNKNDSD 2500
etht|ETH2_0728100    ------QQQQQQQQ----------------------------------QQQQESESDSSSEEEEANVKQEKGEAAKVKRE 2264
sneu|SN3_00201460    ------KSGRRKERKLRDSHNSAEGGVVAAE------EAEI-------ESHYTDSESES----RLSAE-SSGESGPEEER 2344
tgon|TGME49_244880   ------KSGQRAD--------SARGDATH---------IKV-------EQTSEDESSASCEDEEATSS-DEDDDDPEDDG 2332
gnip|GNI_013220      -------------------------------------------------------------------------------- 1217
chot|ChTU502y2012_38 ----------------------------------------M-------NN-------GKFTE-EAN---ENDESGSEEDG 1921
bmic|BMR1_03g03880   ------------------------------------------------------------TM------------------ 1474
tequ|BEWA_039100     ----------------------------------------------------------SCSY-SSS---ESDEDAEQEEE 1595
cvel|Cvel_8831       ------------DEEMDDDHDGQRRKLK-AR------------------------AKQVLD----------DEDAGSDID 1923
vbra|Vbra_21242      ------------AEEEEDRPAKKPKKPTPPPAAAAAAAAAAPSPSPAPESDEGEEPEEVADEDMADAD--QDQDQDRDQD 2098
tetr|Q23DD6          ---------------DELKSKSKP-------------------------------------------------------- 1543
hsap|ENSG00000068654 ------------DD-EDMQEERNPHRE--------------------------------------GAR--KTQEQDEEVG 1467
scer|YOR341W         ------------SDEEGIDSDKE--------------------------------------------S--DSDSEDEDVD 1423

htar|Htart_000206800 KEEDSEEEME----TEMIDADEE-HEHGNR------------NRNGNSEEYTRSSNKEGTESDKEYIIIDEKK------- 2798
pber|PBANKA_1109000  KKKKSEKKKIRKKKSKLLNETDS-------------------------ESDSSS-----DQSITDSLIIDEESKGSNKSK 2251
pfal|PF3D7_0509400   IDSDSESEMVSSNKKEIININNKDNYYTNRNSSNESKLSECSNTKGSTVNYFSS-----ETSATHHILVDEEESGIFPNL 2575
etht|ETH2_0728100    IKE---EEFEANKQPI---------------------------------------------------------KKIKK-- 2282
sneu|SN3_00201460    MAEDAVDDKVA-LQTA---------------------------------------------------------AGSPR-- 2364
tgon|TGME49_244880   HTGKAEESTVSSEKPA---------------------------------------------------------KGEVK-- 2353
gnip|GNI_013220      -------------------------------------------------------------------------------- 1217
chot|ChTU502y2012_38 -------DLVR--RSL---------------------------------------------------------N------ 1929
bmic|BMR1_03g03880   -------------------------------------------------------------------------------- 1474
tequ|BEWA_039100     -------D-----------------------------------------------------------------T------ 1597
cvel|Cvel_8831       AGDESEEDR----------EDED-GE----------------EGVGRRG------------------------RGWRGRR 1952
vbra|Vbra_21242      MAAAEQEDEDAGA-PEEKEEEKP-PE----------------KPKGYRQ------------------------VDWKKPL 2136
tetr|Q23DD6          ---------------------------------------------QSA-----------------------------EPV 1549
hsap|ENSG00000068654 LG--TEEDP--SL-PALL---TQ-PR----------------KPTHSQ-----------------------------EPQ 1493
scer|YOR341W         MN--EQINK--SI-V-------------------------------EA-----------------------------NNN 1438

htar|Htart_000206800 ----IETLLEN----------------------------LKLR---HKNGLYFLQANDG------TNNLEEF-------- 2829
pber|PBANKA_1109000  KEDKLNTSGKKKKLIDGND-------YIKNEQ-------HRKE---NKNKLKEV-NSDYSMSSESEAELKKEMNKKFSEY 2313
pfal|PF3D7_0509400   KNKKLNNMNDNKNMSSTTVSSSLSSSSLSTPL--NKKNIEDDE---HINNIHFIEKNKYN---EEMDNKEEFITKSFTRL 2647
etht|ETH2_0728100    ---EQQ--QQQQQQQQ----------QPQQQLLDLADVLRGKRFDRTLGLFVDA--KPSKANPETLNPKP-------LSS 2339
sneu|SN3_00201460    -S-NRRTFASNNINIS----------KRSSPLLLSFAELLRGKEFNLRTHLFEAQ------------------------- 2407
tgon|TGME49_244880   -R-DRRTL---DAAFE----------SMFSPLLLSFARLVKGETFDMNTHLFFPQ--DKKQCSRA------------SKT 2404
gnip|GNI_013220      -------------------------------------------------------------------------------- 1217
chot|ChTU502y2012_38 ---GRNNFTG--------------------------------------ENLTEND------------------------- 1943
bmic|BMR1_03g03880   --------------------------------------------------TR---------------------------- 1476
tequ|BEWA_039100     ---GYKGFES--------------------------------------EIKF---------------------------- 1608
cvel|Cvel_8831       REGRE--------------------------------TEDEGEGAGVSAGGRVIG------------------------G 1976
vbra|Vbra_21242      RPERLSPFRPWSDNIR----------QRVLPR-LDLRSEQEHRQYGYWDGNAYVD------------------------- 2180
tetr|Q23DD6          KR-----------------------------------VPTRTSKAELFEASPY--------------------------- 1567
hsap|ENSG00000068654 GP-----------------------------------EAMERRVQAVREIHPFID------------------------- 1513
```

```
scer|YOR341W          MN-----------------------------------KVQRDRQSAIISHHRFIT------------------------- 1458

htar|Htart_000206800  -----------QRH--------MKDFE-----------------RLELPVFEK-MKNIFRFLKSLKFCPVSWILKIEIGW 2872
pber|PBANKA_1109000   SYDYKNRINKKNLD--------VSDYE-DPSIGISGSKNKLNDQKYIKNLTDKIKSSLLCFIKKIDFSPITWVLKFELSW 2384
pfal|PF3D7_0509400    SYDYKNNINLISSD--------D-DQE-NIEENEEGQKMKKSEQRYVKNIIDKIKSSLLCFIKNIHFSPVTWILKLNIGW 2717
etht|ETH2_0728100     EYP----------P--------PAPFE-ADAVWSSGAS---------FKKLAQLDMESATILKDIKVCRKTWRIVVKFGW 2391
sneu|SN3_00201460     ------------------------------------------------------LRTVASILQGISVCPRTWRVVVKFGW 2433
tgon|TGME49_244880    PLA----------V--------PAPVE-SDGLWADGRK---------YKGFVLITREVASILHEVFVCPRTWRIILKFGW 2456
gnip|GNI_013220       -------------------------------------------------------------------------------- 1217
chot|ChTU502y2012_38  ----------------------------------SKIP---------CINLKILTKDVLKHVIDFNIGRKTNILSIKLGW 1980
bmic|BMR1_03g03880    ------------------------------------GK---------VVGSKALKSKYATFYHSLKFSKHLWLMEYKLRI 1511
tequ|BEWA_039100      ------------------------------------GK----------QDIRSIDRKVFRFAHSLKFCKDTSRLILKFGW 1642
cvel|Cvel_8831        AYGKE--MSKMQKDSRGRRLVRETRWDPDEMEYTVI--LE-------------HPIVPEFIPAPLDFLRPA--------- 2030
vbra|Vbra_21242       RRGYI--VGT---------VGVFKAYDKEEMTSEEYKPLE---VRGITFKTEVLDSRKSLIRGPIMFDRKGSCMTLEFKY 2246
tetr|Q23DD6           -YNG----------------------HTIEGERIVC-NIK---LPLDSKKLLMVNLVEKILKKTLVYE------------ 1608
hsap|ENSG00000068654  DYQY----------------------DTEESLWCQV-TVK---LPLMKINFDMSSLVVSLAHGAVIYA------------ 1555
scer|YOR341W          KYNF----------------------DDESGKWCEF-KLE---LAADTEKLLMVNIVEEICRKSIRQ------------- 1500

htar|Htart_000206800  VMDLFPYRIDFSDVFENEIPTETIYSIKDFKNPKIVRG-SDIT--QTKSEFELQIEGKNAFKLFNLKDKYIDKSKFYCND 2949
pber|PBANKA_1109000   DINFFPYPIDFLSCLQNEISKEVLFKVKDLNNPKILKG-KDIT--HSGEEYELQIEGRNVYKLFNIKDKYIDKTKLYCND 2461
pfal|PF3D7_0509400    DINFFPYPIDFLNHIKSEINKEVLFKVKDLNNPKILKG-KDIT--HSGAEYELQIEGKNIYKLYNIKDKYIDKTKLYCND 2794
etht|ETH2_0728100     PFDKCPYRLELLPIVKGLVEKVTLQEPQGVSNPRIVQG-SGCS---TGSPMELHCSGSNLQWLHRLKETAVDHSKLETND 2467
sneu|SN3_00201460     PMEKCPYRLEVLPVLRNLLQKSYIQRPEGVAAPRIVQG-EGCA---LGQESELQCEGSNIQWIHQMKEEAIDHAAIRTND 2509
tgon|TGME49_244880    PFERCPYRLELLPLLMGLLKKSKMQEPEGVAAPRIVQG-EGCV---TKQKVEIQCEGSNFGWIHRMKDEAIDHNGILTND 2532
gnip|GNI_013220       -------------------------------------------------------------------------------- 1217
chot|ChTU502y2012_38  PVIRCPHYIDFLPVLKNCILRTNIQSISCLKNARITRN-QKETSDKSLSQFEVTVEGTNVGHVFKISPRYINHDKIRFND 2059
bmic|BMR1_03g03880    PFRKCPYKLCLMQILKNCVDAQVVKSVKGVKNPFIVHK-NTMT----GAEYELQCSGSNLNLLISLINDGLDCNRVTSND 1586
tequ|BEWA_039100      PVSKCPYYLDLLPLLKQEISQETLRKSPGIRQSRVVLN-NSHD---GKDDYTLHCDGTNLKRVYLLKEGIIDFNKIKIND 1718
cvel|Cvel_8831        ------------LKAASRLHVNKIEGVHKA--ALVRHAPEEIAHSATGHEYEIVTEGVNLFAAAQLSDEWIDHTKSRAND 2096
vbra|Vbra_21242       NADLYPKALVPKIQWHRPFRKKLICGSQKISCSKVLKDKDGGRNTVTGAEYEIEFNGIDFGMFGKLKDEFIDHTKTRMND 2326
tetr|Q23DD6           --------------------------VKNISNATVLKK-D----NKGATSYQIQTEGVNFKQIWK-MDHF-DSNTIDSND 1655
hsap|ENSG00000068654  --------------------------TKGITRCLLNET-TN---NKNEKELVLNTEGINLPELFK-YAEVLDLRRLYSND 1604
scer|YOR341W          --------------------------IPHIDRCVHPEP-EN---G----KRVLVTEGVNFQAMWD-QEAFIDVDGITSND 1545

htar|Htart_000206800  IQTMSNLYGIEAARVCIIKELKKVFDAYGIVIDFRHLSFLGDYMTHTGVIRGFQRSEVG-KYRNVLHQMSFESATTFFKT 3028
pber|PBANKA_1109000   IYTIVKTYGIEAGRLCITKELKKVFEAYGIKIDFRHLSFISDFMTHTGDLKSFNRYGIN-AYRNVFHKMSFECATSFMIQ 2540
pfal|PF3D7_0509400    IYAIVKTYGIEAGRLCITKELKKVFDAYGIQIDFRHLSFISDFMTHTGDLKAFNRHGLG-YFRNVFHKMSFECATNFLIQ 2873
etht|ETH2_0728100     VLAMLQHYGIEAARALYLKEFSRVFAAYGIKVHSSHLGLIADFVTRDGAIIPFNRLGIQ-HCDSPFLQMAFETSFRFLTE 2546
sneu|SN3_00201460     VRAMLKHYGVEAARVCLVKELEKVFSAYGIKVDYRHLALIADFMTHDGGIRPFNRSGIA-ASNAPFLQMSYETSFKFLTE 2588
tgon|TGME49_244880    VRAVLKYYGIEAARVCLARELERVFSAYGIKVDYRHLALIADFMTHGGTLRPFNRLGMA-ASNAPFLQMSYETSFKFLTE 2611
gnip|GNI_013220       -------------------------------------------------------------------------------- 1217
chot|ChTU502y2012_38  IQTVYKYYGVEAARHCIINELRNVFSVYGINVDYRHLTLISDSITASGKLRVFNRMGTISYNISPFLQMSFETSMKFLTE 2139
bmic|BMR1_03g03880    IIAIHHTYGIEAAYAKIIQELKMVFASYGISVDHRHLSLVADYMTNDGDIKGFSRYGIA-RHTSPLQQISFESSMKFLTK 1665
tequ|BEWA_039100      ISTVLHYYGVEAARACIVSELNKVFSVYGIDVNYRHLTLIADFMTQKGDIRAFSRYGMA-KHNSPLLQMSFESTMKFLMS 1797
cvel|Cvel_8831        IVPVFLTLGVEAARVVYIQQLALVFDAYGIKVDKRHLSLISDFLFRSGKLRAFNRTAMQ-SETSPLLQMSFETSTGFLSE 2175
vbra|Vbra_21242       PYMTLVHLGVEASQRIIIQELEAVFSVYGINVSFRHLSLIADVMCRNGDYRSFSRHGLK-YHHSSLLRMSFETALGNLNE 2405
tetr|Q23DD6           IAALNKCYGIEAARNAIVKEIVTVFGVYGVVVDYRHLYLIADYLTFSGEMKAMNRVWMD-ESISPFLKMSFETCVKFLTN 1734
hsap|ENSG00000068654  IHAIANTYGIEAALRVIEKEIKDVFAVYGIAVDPRHLSLVADYMCFEGVYKPLNRFGIR-SNSSPLQQMTFETSFQFLKQ 1683
scer|YOR341W          VAAVLKTYGVEAARNTIVNEINNVFSRYAISVSFRHLDLIADMMTRQGTYLAFNRQGME-TSTSSFMKMSYETTCQFLTK 1624

htar|Htart_000206800  GCITKSIDNLVTPSSNLYLGKTLKLGTNIMDVIPDICNQPIN-------------------------------------- 3070
pber|PBANKA_1109000   GCIQNSIDYLSTPSSSLFFGKHIKVGTNVSNIVTCIKGGK---------------------------------------- 2580
pfal|PF3D7_0509400    GCIHNSIDYLSTASSSLFFGKHIKVGTNLADIVTCIDKQNL--------------------------------------- 2914
etht|ETH2_0728100     ACERSAVDNLKTPSAALIVGSPAVIGSHQSQLLAVLPDFSDS-------------------------------------- 2588
sneu|SN3_00201460     ACERAATDSLSTPSGGLIVGTPATVGSAQCEIYTQLGSVAGCSS------------------------------------ 2632
tgon|TGME49_244880    ACERAAVDSLSTPSGSLVVGAPSVIGSQQHQVFTQLHPALHRQRDLFQLSRKSCVASQHGGTGDTKGEPEALVSQIKKKL 2691
gnip|GNI_013220       -------------------------------------------------------------------------------- 1217
chot|ChTU502y2012_38  ACLRGALDNLKSPASSISVGKMVSVGTGISRTYTNFYQNSFGGKRKD------------------K--DGSIQKKIKD-- 2197
bmic|BMR1_03g03880    ATERMAQDNLKSPSGALVTGARVFVGSSLCDLLYQLPAKKA---KYR------------------R--PKPSDFKFLD-- 1720
tequ|BEWA_039100      ASERGAYDNLKTPAGAIIAGKPVHVGSNLCKIMQVINIKER---ELP------------------N--EQNAQIMF---- 1850
cvel|Cvel_8831        ALVKQSTDNLKTPAGALVTGKQTRGGTGMAEVLVRPQLKGMPSL-WSRWPGADRK------------QPGRKKRKLTK-- 2240
vbra|Vbra_21242       AIETDAADHLTTPAGAVVAGKLARVGTGTFDILPVLPAGQGPVKKEFSFRAIKME------------EDGE--------- 2464
tetr|Q23DD6           AATNNEIDTLSTPSSSIVLGRVPQVGTGVFDILHKV-------------------------------------------- 1770
hsap|ENSG00000068654  ATMLGSHDELRSPSACLVVGKVVRGGTGLFELKQPLR------------------------------------------- 1720
scer|YOR341W          AVLDNEREQLDSPSARIVVGKLNNVGTGSFDVLAKVPNAA---------------------------------------- 1664

htar|Htart_000206800  ----------------------------------------------------------------------------- 3070
pber|PBANKA_1109000   ----------------------------------------------------------------------------- 2580
pfal|PF3D7_0509400    ----------------------------------------------------------------------------- 2914
etht|ETH2_0728100     ----------------------------------------------------------------------------- 2588
sneu|SN3_00201460     ----------------------------------------------------------------------------- 2632
tgon|TGME49_244880    GASEHQADNATDRKETDRAETAGEQEVTGVKKEKCSKRHKERRDSAMSNEAFQPGENGEDDDAKQKKKRKRKSFDFI 2768
gnip|GNI_013220       ----------------------------------------------------------------------------- 1217
chot|ChTU502y2012_38  --KKEKNDDETKKGKKTR----------------------------------------------------------- 2213
bmic|BMR1_03g03880    --QQENESNESEISDDVMSN--------------------------------------------------------- 1738
tequ|BEWA_039100      ----------------------------------------------------------------------------- 1850
cvel|Cvel_8831        ---------TTDKGRVSSGEN---KKMAK---------------IIPQEEDEKPGNSGMQQKEKSKKRKEGR----- 2285
vbra|Vbra_21242       ----------------------------------------------------------------------------- 2464
tetr|Q23DD6           ----------------------------------------------------------------------------- 1770
hsap|ENSG00000068654  ----------------------------------------------------------------------------- 1720
scer|YOR341W          ----------------------------------------------------------------------------- 1664
```

Figure S3.2. RPA2 (trimmed)

```
htar|Htart_000272800 KKLHINKLVKGTYNIFTRYLVQSHIEDYNSFLNVYIKNMPQHI----PP---IEFSPKLNH----YGLADVAKKKNQSVK 79
pber|PBANKA_0913800  IKLKIDKVINGTYNIFTKFLVQSHIDDFNSFINVYIKNMPETL----PV---IEYSPNLNK----YSCLNYERKPKERIK 137
pfal|PF3D7_1134700   MKLKVDKLINGSYNIFTKFLVQSHIDDFNSFINVYIKNIAENI----PI---MEFSPQQNN----YSLLNMNKNNSESVK 120
tetr|Q22C62          AKQS-----NLDFSKKLKQAAFAHIDSFNFIYDEGLKKITQYL---Q---------PVEIIKEQRSEEYKQSYLPFKSMK 76
hsap|ENSG00000125630 LTDPSYGIPREQQKAALQELTRAHVESFNYAVHEGL----GLAVQAIP--------PF-------EFAFKD-----ERIS 112
scer|YPR010C         ------------AFPLLQEAVQPHIGSFNALTEGPDGGLINLGVKDIGEKVIFDGKPLNSEDEISNSGYLG-----NKLS 96
cvel|Cvel_15870      -MPR--GIPEIEPNDRWTSLASAHIDSFNTFLNEYLPAIVEGL----PR---IHVHPLYYKTMDPPPSVDYDRHSDKVLR 70
sneu|SN3_04700070    TMPAS-ARCRCAFNAALQQAAFPHVGSFDTFARVYIHRIVRSL----PV---LYVDPVLDTDR-D--AINPYYVPTEHVK 145
gnip|GNI_119110      YLPHKPTYETPQFNEKFLSATHPHIASFNLFLNHYIKEIPKHL----EP---VYVDVTEDPRI-T--GLDPHHFPSEYLT 90
etht|ETH2_1434600    LNPLS-PLPSPHFNAALQQCVYPHILSYNTFADCYVEEMVKEL----PF---IYVDPNYDPDR-H--EANPYYKPSDYLK 107
tgon|TGME49_297530   TMPAS-PHPRAAFNATLQQAVFPHVRSFDSFLEVYLQEMVKDL----PV---VYVDPSYDPDK-H--EANPFYSPTDYIK 109
vbra|Vbra_22055      SHPVTTNQPASQPTPLVKKAVFSHIQSFNWFLQSALPRIVAEL----PA---FEVDPHEDFDWRS--SFPRPTPPKISLK 96
chot|ChTU502y2012_41 --------MEYQTRDRLLSAVWPHIESFNTFLSVGLKNIVKNL----PK---IYVDSGFIQDD-C--ELNAAYSPAEYLR 62
bmic|BMR1_01G02877   --MKSMREMDSPYRLELSDIAASHIGSFDAFLDTYSKRIVECL----PP---VYVAPNYNQML-H--ELNSGTAAPDYVK 68
tequ|BEWA_006420     ---------MVEFNVDRMSISHSHVDSFNAFIDHYSVNMVKRI----PP---VYISCDNEY-L-S--RFLNPNEKPYYVK 60

htar|Htart_000272800 FFVSDIKIKKPVILGENL-------------------------------------------------------------- 97
pber|PBANKA_0913800  LYVSDIIIKSPIIKSDNG-------------------------------------------------------------- 155
pfal|PF3D7_1134700   FFVSDIQIKNPMIKNDKG-------------------------------------------------------------- 138
tetr|Q22C62          VWIENLEIATPIRADS-G-------------------------------------------------------------- 93
hsap|ENSG00000125630 FTILDAVISPPTVPKG-T-------------------------------------------------------------- 129
scer|YPR010C         VSVEQVSIAKPMSNDGVS-------------------------------------------------------------- 114
cvel|Cvel_15870      FGITRLEIGKPVHADPEE-------------------------------------------------------------- 88
sneu|SN3_04700070    LGIESVSLGTPTRMHLPAASEESASSTSSSVSSSSSSSPSRRSKGAATAAAAVAATASSSPHQKKKQQQQGNGLQRQQEQ 225
gnip|GNI_119110      VSISRVELGMPRRVDNES-------------------------------------------------------------- 108
etht|ETH2_1434600    LGIAEAVLGFPQKLGVGGLQAK-------------------------------------------------------S-- 130
tgon|TGME49_297530   LAISSVSIGAPVRFDAAGARLQ-------------------------------------------------------PGV 134
vbra|Vbra_22055      FWLRSVEVGRPTRFEDTM-------------------------------------------------------------- 114
chot|ChTU502y2012_41 IGVTSAKIGKPIFGNSES-------------------------------------------------------------- 80
bmic|BMR1_01G02877   FFIKDVRIGYPRRNDCT--------------------------------------------------------------- 85
tequ|BEWA_006420     ITVLSLKIGYPTRPGSEC-------------------------------------------------------------- 78

htar|Htart_000272800 --ETAIKKDYPYLCKIAGRNYEGEIVVTLNRVCNG------EVFSTSLSCGYMPIMVLSDKCNLCGLTKEELVQ-KGEDE 168
pber|PBANKA_0913800  ----NTRPNYPYLCKLACSTYEGEVLMVINKQYKN------EIVRTTVSTGFIPIMVLSDLCNLKNLNKKELVQ-KGEDE 224
pfal|PF3D7_1134700   ----EYRADYPYLCKLSARTYEGELLIKINRQYKD------EIASTTICAGHIPIMIMSNLCNLSNLNKKELAQ-KGEDQ 207
tetr|Q22C62          --VAEDNKLMPEECRIGRKTYGAPLFAKVARSIDGGKP----ESIN-IRLGDIPIIVRSDHCHLAKMTPKELVK-AHEDC 165
hsap|ENSG00000125630 --ICKEANVYPAECRGRRSTYRGKLTADINWAVNGISK----GII-KQFLGYVPIMVKSKLCNLRNLPPQALIE-HHEEA 201
scer|YPR010C         --SAVERKVYPSESRQRLTSYRGKLLLKLKWSVNNGEE----NLFEVRDCGGLPVMLQSNRCHLNKMSPYELVQ-HKEES 187
cvel|Cvel_15870      ----KNPILLPTHCRVGHFTYAAPLKIYYTREHGG----LHAPEEFGFEAGYFPIMLKSDKCHLKGMGPPGLLK-SREDP 159
sneu|SN3_04700070    QNGQQQQLLLPRHCRETHTTYAAPLYISFFRSSST--NGRSSVIRKTVQLGGFPVMVRSCVCSTRGMSPEELQQ-AGEDT 302
gnip|GNI_119110      -HSGSDTRQFPVHARLSHVTYGAPMYISYVWNNSA--G--KKVKERTVLSGVCPVLVRSDACHLSKLSAFEMARYAGEDI 183
etht|ETH2_1434600    ---DAGSRLLPRHCRDSHISYGAPLHVSFVCYRTG--DESNAAIKKTMVVGQVPVMVKSNRCTLSGMSPTEMVN-AGEDL 204
tgon|TGME49_297530   GGSGDDQLLLPKHCREAHITYGAPLKVSFVRSD----RRDGAALKKEVVVGMAPLMVRSKLCSTRGMSPAELQR-AGEDV 209
vbra|Vbra_22055      ----MDQTLFPNHCREARVHYAAPLTFKWAYQWSDEDEP----TEVVDPAGQLPIMVQSIKCNLHGLTSEQMVA-HKEDE 185
chot|ChTU502y2012_41 --SG--TKLTPQHARNGHISYSAPLWLEFEGYNSS----LDTVSKHSIYAGTIPIMVKSSNCHLFGLGTKELVE-KGEDP 151
bmic|BMR1_01G02877   --DDADERLYPLQARISHTTYSAPIYVTFALQSTSGGVECSQIVHKELLAGQIPVMVKSKRCNLCGLTGDELVA-RGEDA 162
tequ|BEWA_006420     --IGIKQHMYPRHAKTSHTTYEAPLVVTFGIKFSD----TESIVTKELIVGHIPVMVKSKRCNLSGLTPEEMVQ-QGEDI 151

htar|Htart_000272800 SVLGGYFVVSGRLRIIRFITSRKFNTCMVMNKGK----------RYAYMLCLLK-DNTVVLNNLLQKENDTYVHISRYYG 237
pber|PBANKA_0913800  SLLGGFFIISGHLKIIRYVICPKYNSILLNEEN------------KVHINCLLS-DNSVFINSLAYLKNYEYEFLSRFQG 291
pfal|PF3D7_1134700   SLLGGFFVVSGRLKVIRYVIHPKYNTLLLNADN------------KIHINCLLN-DNTVVINFLTLTRNNSYVYGFRFQN 274
tetr|Q22C62          NEFGGYFIVNGNEKIVRMLILQKRNYPIAFIRAGYVNRGPNYTPFAVQMKCVRE-DLYSKTITLHYVSDGSVFLRILHRK 244
hsap|ENSG00000125630 EEMGGYFIINGIEKVIRMLIMPRRNFPIAMIRPKWKTRGPGYTQYGVSMHCVRE-EHSAVNMNLHYLENGTVMLNFIYRK 280
scer|YPR010C         DEIGGYFIVNGIEKLIRMLIVQRRNHPMAIIRPSFANRGASYSHYGIQIRSVRP-DQTSQTNVLHYLNDGQVTFRFSWRK 266
cvel|Cvel_15870      NEPGGIFVVNGNEKLIRLVISTRCNFPMPMRRGTFRNRGWRFSDKAIICRCQRP-DGSQTSNILHYCTDGTMYLRILINR 238
sneu|SN3_04700070    DDGGGYFIVNGVEKVLRFVLQQRTNFPLALKRESFQEREEFCTSHAVVFRCQRL-DGTCISNFLLDTEDGRCCFRILLNR 381
gnip|GNI_119110      EEVGGYFIVNGNERGVRFVIQQRCNYPIAIKKKSLRDRDVFYTEYAVAFRAARS-DGSSTNNIVFYTEDNQCVFRILLNR 262
etht|ETH2_1434600    DELGGYFIINGNERVLRFVIQQKTNYPIALKRESFRNREIFATDLAVLLRSQRI-DGTCTSNILLDTEDGRCCYRILLNR 283
tgon|TGME49_297530   DDGGGYFIINGNERVIRFVVQQRTNFPIALKRPAFANRDAFCTPYAVLLRSQRY-DGTSTSNILLDTEDGRCTYRILLNR 288
vbra|Vbra_22055      NDPGGYFIINGNERLIRFVIKQRSNYPMLLRKHAP-FKRLGKLKCMITMACLRQ-DGSQLVNHIYSFLDGTAIFQTLIKR 263
chot|ChTU502y2012_41 NEVGGYFIINGNERVIRYVIQQRCNYPIGLKRPRFATFDSFGSEYAVLMRSLRD-DGTSTANYLYGTSDHQCIYRVLLNR 230
bmic|BMR1_01G02877   QEPGGYFIVRGNERLIRHMISSRSNYPIGVKRKQFRAKDAFNTEYAIIMRSQRR-DGTVVGNVLCYTEDERCVLRVAINK 241
tequ|BEWA_006420     DEPGGYFIIRGVEKIIRQVIITRSNYPIAIKSENNSLRNVLFTDYSIVMRCQSHDDGCYVTNVLYLTINKRCMFRVLIKN 231

htar|Htart_000272800 NVILMDFHILAMCLSPIKSK-----TYIINKLKAGITNENVM-KYIE-----------------QYV------NTSFINA 288
pber|PBANKA_0913800  SFIHIPFNVLILCLSPIKKK-----SYIYNKIKLGIKNENAI-KYIE-----------------NYI------NSIFLKN 342
pfal|PF3D7_1134700   AVCSLPFHLLLMILSPIKKK-----SYIFNKIKLGVENENAI-KYIE-----------------LFI------NSIFLKD 325
tetr|Q22C62          QEFLIPAIVILKALADISDL------QIYNKLVKGQSSKSAISDRVEVLIRGS--------------------------K 292
hsap|ENSG00000125630 ELFFLPLGFALKALVSFSDY------QIFQELIKGKEDDSFLRNSVSQMLRIV--------------------------M 328
scer|YPR010C         NEYLVPVVMILKALCHTSDR------EIFDGIIGNDVKDSFLTDRLELLLRGF--------------------------K 314
cvel|Cvel_15870      AEHYVPFFAVFNCLCSNAGPLKANRAVAYRKLADFGGGKDARELQEIAFMRIRQMHIEGPRDNDTFADAFKQADVDRDAS 318
sneu|SN3_04700070    QEHFFPFLLLLRCLAGELSL----QQLK-AKLLQHCWEDPHEAATV---------------NFFLEE------EAFEDGA 435
gnip|GNI_119110      GEWTCPFWLLLKALFPTCQL----KMVK-EKLLSL-GEDLELESEI---------------SN-LWH------DFIEGDV 314
etht|ETH2_1434600    QEHFCPFPLLLRCIGGGLSL----QQFK-LKFMEGTWGDLEESTQL---------------HA-LFE------EVWGNEK 336
tgon|TGME49_297530   QEYFCGFFLLLRCLAGNLSL----AQLK-AKLLEGCWDDAADATAR---------------SQ-LVE------ELWGQEA 341
vbra|Vbra_22055      RSVYLPFYLLLLATNPLPAG----DQCIWERLIDGDVTNMQLLFLV-----QTLMDDAKTRFSTLWP------------- 321
chot|ChTU502y2012_41 QEWLIPFWPLLMAVGSIYDS----NVLR-SKILQYCNPKDENLFNTINMLTLEWGLDNSVGVDAEFN------SVSFGNK 299
bmic|BMR1_01G02877   VIKTIPFGTLLKAAKPHMST----WDLK-KRLLEDFLHNEEMTV----------------HFYNIFQ------NMIFLEP 294
tequ|BEWA_006420     SVVLIPLTLLLRALVPYMSD----DELK-QKLLQNTIGDEEMTT----------------YYHQFFL------DMIRCDP 284

htar|Htart_000272800 DMNDRELIEDCYLGYLGKYVRFRTGVLRFLRGTVQQKAKQYLKFCVFPHL--DNNAEKFEALCFMFKMLILNKF----KL 362
pber|PBANKA_0913800  NINEKEIFEKYNLKYIGKYANVSRTIFRYYEGNNHKKGKSVINYCVLPHL--QTNSEKFETICLMFKQLVLSKF----KL 416
pfal|PF3D7_1134700   TFNEKELFEKYNLSYLGRIAYLRRGIFKYNLNCYEKKAKDILKYCILPHI--KNNSEKFETMCFMFKKLIYSNF----KL 399
tetr|Q22C62          AM--SLNTKEECLAHLGKNFR-VVLNITNPEITDKQVGEIFMNENICVHL--NNNIDKLNTICVMISKLYALVS----DE 363
hsap|ENSG00000125630 EE--GCSTQKQVLNYLGECFR-VKLNVP-DWYPNEQAAEFLFNQCICIHL--KSNTEKFYMLCLMTRKLFALAK----GE 398
scer|YPR010C         KRYPHLQNRTQVLQYLGDKFR-VVFQAS-PDQSDLEVGQEVLDRIVLVHLGKDGSQDKFRMLLFMIRKLYSLVA----GE 388
cvel|Cvel_15870      TFSASDDWCQRWKDQFLDLAGLRLREGLPAHTTDDDKIRVLCDEYVLPHL--KDWGKKAELLALMWRMMLMVIR----NK 392
sneu|SN3_04700070    AFADSDIYEHRALARLGKLFYSAVEWLLPPAATLEDAGRIVIEQAVLPHV--DDWSEKFEVALLMFRKLRLMKA----GK 509
gnip|GNI_119110      LHADQDLHEDRHLALLGYQFWDGVYQRLPPGATYATAGRYIIDRYVLVHT--ESWIGKFETLILMWQKLWCLKR----GL 388
etht|ETH2_1434600    DFVDTDLREHRPLHQLGQVFWRGVSWLLPPGSTFEDAGRFIIDRYVLVHV--KEWSSKFETLLLMFRKLRQLRR----GK 410
tgon|TGME49_297530   TFADSDVLENRPLHQLGLLFWKAVHWLLRPGSSYEAAGTYVIRHFVLPHL--SSWSEKFDMALLMYKKLRLLKQ----GK 415
vbra|Vbra_22055      -----SLSRDKARQLLGDLIWDHIRELVSPWCSYRECSTLFLRKYVLPHV--DSDAAKFDMLCFMWKKMISRAHLKGKHK 394
chot|ChTU502y2012_41 GPGDTDILENRYLHQIGRLVWEGVAFHLPPGSLLQEAGRFLIDNYVLVHL--EEWNAKLECLILMFIKLIKLQL----NE 373
bmic|BMR1_01G02877   LVADVDFVENRYLHQLGKACWHRVHTFMRPGSSLAECGLFVMKNYICVQS--ESLDDKFECLLHMFKKLILLAR----GY 368
tequ|BEWA_006420     ILSDIDFTENRYLYRLGKVCWSRINQYLHPGATFEECAVYFLKYFVIIHA--HTNVEKFETLLFMFNKLIKLSR----GE 358

htar|Htart_000272800 IETNEMDSLEFQGVATPSHFISGVLKDQIMNALNIFAYRYQDMYIKTLNKKFEQFKTQLIDISTKYKNYIEDELKEL-KQ 441
pber|PBANKA_0913800  ITTVNKDSLEYHSVTTCSSLLSNILKERILSCFYRMQMLYSRSLHKFLEKKYEVFKLNVKQIYLRYKEMILHELSED-HF 495
pfal|PF3D7_1134700   ITPINKDSLENHAVTTCSNLLANLLKDQVMNSLCRLYIRYTRSFFKYFETKYNTFKLSVKQIYLRYKMFVLEEMQED-RL 478
tetr|Q22C62          ICPDNLDSLQNQEVLLPGHLYMMILREKLEEMLLGIRAKVFKDGSRPE-------------------E------------ 412
hsap|ENSG00000125630 CMEDNPDSLVNQEVLTPGQLFLMFLKEKLEGWLVSIKIAFDKKAQKT--------------------------------- 445
scer|YPR010C         CSPDNPDATQHQEVLLGGFLYGMILKEKIDEYLQNIIAQVRMDINRGM-------------------------------- 436
cvel|Cvel_15870      VKCCNQDAFAFQEVLLPGQLIAQVVKSALYDQLMSMRTFLELGCFRMK-------------------G------------ 441
sneu|SN3_04700070    IAPENLDSFAYQELVLPGQMMAAVLKDALLSCLAKVRLNYLQELRMAK-------------------T------------ 558
gnip|GNI_119110      IPAENVDSFAFQEVLGPGQLFAAVLKDALWNSLQAVRLAYNNEVREAK-------------------L------------ 437
etht|ETH2_1434600    IPPESMDAFSYQEVILPGQILAAVLKDALFNSLIKLRTQYMVELRQVK-------------------T------------ 459
tgon|TGME49_297530   ISAESLDSFAYQEVVLPGQILASVLKDALFSCLAKIRLHYLQEIRMLK-------------------N------------ 464
vbra|Vbra_22055      TTYENVDAFAFQEVITPGQIFGAVFRDVCEQNLMRMRGSFYGEAKQAR-------------------N------------ 443
chot|ChTU502y2012_41 IREESIDSFAYQELLSPGNLYSALFKDAIFSFLQKIRSTYLIEIRSKS-------------------G------------ 422
bmic|BMR1_01G02877   IIPDQIDSLAFQELTLPGQIYASVLKESLYSALTKLKSLYSIEVANFK-------------------KYLATVEGV---- 425
tequ|BEWA_006420     IQSESYDSFAFQEILLPGQTYSCILKESLYLALTRIKKIYSTEISYFG-------------------RYLQGDVSARSKR 419

htar|Htart_000272800 QEKKTEREKERN-ENTEVNSESKKETQVSDNGREIPSSITASSVETLKKQHLQQMKEQYLSEKEKLFDSINTCVTSMFEE 520
pber|PBANKA_0913800  NQILLNNNADKLENNEELTSE--------------------NDHMNSKMDSTKNLRLMFMNEKEKLFASADGYLREMFSD 555
pfal|PF3D7_1134700   NQISLSKSSEDINDSS----Y--------------------QDNNDYKNESKKKLKSIFINEKEKLFRSVDNYFKELYND 534
tetr|Q22C62          -------------------------------------------------------AI--RIPHIAYLKK----------- 424
hsap|ENSG00000125630 -------------------------------------------------------SV--SMNTDNLMR------------ 456
scer|YPR010C         -------------------------------------------------------AI--NFKDKRYMSRV---------- 449
cvel|Cvel_15870      ------RESENLEA----------------------------------------AAE--WLLSEKTFRTC---------- 463
```

```
sneu|SN3_04700070    ----------SGED----------------------------------------LVE--VAHSDALFDVI---------- 576
gnip|GNI_119110      ----------AGDD----------------------------------------VYG--IIRSESLFEQI---------- 455
etht|ETH2_1434600    ----------AGHD----------------------------------------PTL--VIASNKFFDAA---------- 477
tgon|TGME49_297530   ----------SGND----------------------------------------PTA--AIYSNKFFDLA---------- 482
vbra|Vbra_22055      ----------NNAD----------------------------------------PVA--HLKSAKVFERR---------- 461
chot|ChTU502y2012_41 ------EKNKESKN----------------------------------------GAN--LLRDTAYFKSV---------- 444
bmic|BMR1_01G02877   --------DDMEKV----------------------------------------MLE--FCSNISLFNYL---------- 445
tequ|BEWA_006420     KFMDFGENQDSSTL----------------------------------------IKQ--LLSSVALFNYA---------- 447

htar|Htart_000272800 DASMANGIYAFVAIGVAIQFFFKTGTITS-KSAGCVQSTGCVINADEINNLRMLTNFRALHRGSVFVNMKTLGPRKLKGE 599
pber|PBANKA_0913800  ARVFIENSRTFGAASFSISYLFKTGNIAS-DYIHYQQKSGWVILADEINNLRLLTNFRSIHRGSFFQDVKVLSPRKLLGE 634
pfal|PF3D7_1134700   NYMFLENCKSFSNTSSAILFFFKTGNISS-ENLHYQQKSGWVIAADEINNLRLITNFRAIHRGTFFQDVKVLTPRKLLGE 613
tetr|Q22C62          ------AIEFQGSIGNKIEYFLSTGNLKSQTGLDLMQAKGFTIIADKLNNMRFLSHFRSVHRGAYFTEMKTTTVRKLLPE 498
hsap|ENSG00000125630 ------IFTMGIDLTKPFEYLFATGNLRSKTGLGLLQDSGLCVVADKLNFIRYLSHFRCVHRGADFAKMRTTTVRRLLPE 530
scer|YPR010C         ------LMRVNENIGSKMQYFLSTGNLVSQSGLDLQQVSGYTVVAEKINFYRFISHFRMVHRGSFFAQLKTTTVRKLLPE 523
cvel|Cvel_15870      ------CERHNKTVARNVKYFLGTGNVRT-RTLDILQVSGFVIGAERINIYRYIAHFRAVHRGAYFAQMKTTGVRRILGE 536
sneu|SN3_04700070    ------TERCAPEVVQKMTYFLATGNIRT-TQLDLQQLSGWTLTADRLNIHRFLSHFRGVHRGQFFTTMKTTSVRKLLGE 649
gnip|GNI_119110      ------VMRTCQGPAKALSFFCATGNIRT-YQLDLQQVAGWTITADKLNMNRFIAHFRAIHRGQFFAKMRTTEVRKLVGE 528
etht|ETH2_1434600    ------TDRCASEIGHKISYFMATGNIRT-TQLDLQQLSGWTVTADRLNLNRFLSHFRAVHRGQFFAQMKTTAVRKLLGE 550
tgon|TGME49_297530   ------TDRCCPEIAQKLSYFMATGNIRT-TQLDLQQLSGWTVTADRLNVHRFLSHFRAVHRGQFFTTMKTTSVRKLLGE 555
vbra|Vbra_22055      ------AHGASLEISSRMINFVATGNIKT-EHLDLLQTAGWSISAERINWYRFLAHFRVVQRGSRFMGSRNTMPRKLRPE 534
chot|ChTU502y2012_41 ------LSKNISMIPKRLQYFMATGNVKT-TQLDLQQLSGWTVVADRLNFNRFLSHFRAVHRGQFFTTMKTTDVRKLTGE 517
bmic|BMR1_01G02877   ------TEKVAPEVPSKVNYFLSTGNINS-SYFEFQQNLGWTIIADRMNYFRFMSHFRSVHRGNAFTQMLSTSMRKLLGE 518
tequ|BEWA_006420     ------TDKIAPLISKRLLYFLSSGNALN-SHFELNQSAGFVVVADRINYHRFASHFRSIHRGNIFTKMRSTEVRRLLGE 520

htar|Htart_000272800 SWGFICPVQTPDGAPCGLLNHLTQLTHVHTEASNEC-HKLNIKIFLRHLGIQVNVDDSSGI------------------- 659
pber|PBANKA_0913800  SWGFICPVHTPDGSPCGLLNHLSQYCCIYNVASNES-HKFNIKMYLKKIGININLDETSGI------------------- 694
pfal|PF3D7_1134700   SWGFICPVHTPDGTPCGLLNHLAQYCHIHNLSSNEA-HKLNIKLYLKKIGINVNLDDTSGI------------------- 673
tetr|Q22C62          NWGFICPVHTPDGGPCGLLNHISQSCKPLTRPGYDGNIHFNLVTLCTEIGMNPIVND----------------------- 555
hsap|ENSG00000125630 SWGFLCPVHTPDGEPCGLMNHLTAVCEVVTQFVYTASIPALL----CNLGVTPIDGA----------------------- 583
scer|YPR010C         SWGFLCPVHTPDGSPCGLLNHFAHKCRISTQQSDVSRIPSIL----YSLGVAPASHT----------------------- 576
cvel|Cvel_15870      SWGFLCPVHSPDGAPCGLLLHLTCDARAIGTGRLPAGARHNLLRALI--GLGMEGV--------------GLNFLHQ--- 597
sneu|SN3_04700070    TWGFLCPVHTPDGAPCGLLLHLTQSAEPVTTPPTKR-AVASVKMFLSKLGVAVDAEGETGLPAADLASAARMRFLHTLFA 728
gnip|GNI_119110      TWGFVCPVHTPDGPCCGLLLHMSQDAVVVADPPDGR-FKAIVIDFLQSQGYQT----------SGVGRV----------- 586
etht|ETH2_1434600    SWGFLCPVHTPDGAPCGLLLHLSQMAQPVAVPPDRG-AIAAVRLFMKARGACVDLDGVSGLPPSDLGAACGGQAASH--- 626
tgon|TGME49_297530   TWGFLCPVHTPDGAPCGLLLHLTQSAAPVALPPDRR-ARASIRLFLHKHGVCVDLEGTSGLPPSDVGASARQAARPAV-- 632
vbra|Vbra_22055      TWGFLCPVHTPDGSPCGLLLHLTQDAYVTSAPVGAS-TFDRVAKVLTRLGCLVTSA--NEAPPWGLPGS----------- 600
chot|ChTU502y2012_41 TWGFLCPVHTPDGEPCGLLLHLSQDCIPVTQWDSQE-TLRRLRNYLISHGISCDDGLGSLSSTWGIESSSRDDL------ 590
bmic|BMR1_01G02877   SWGFLCPVHTPDGTPCGLLTHLSKHAWPLTSAPDSG-TMGAVRKFLKDLGYPLDLRAPG---------GITLDY------ 582
tequ|BEWA_006420     TWGFICPVHTPDGAPCGLLLHISQYAVLVTDPKCDD-SISRVKQCLKHLGYCVDLCGNT---------LITNYF------ 584

htar|Htart_000272800 -------------------------------------------YNVQEGQTIPIIVDSVPITYISPEHFKRTYYKLLYAK 696
pber|PBANKA_0913800  -------------------------------------------HTIYENQTIPIIVDSVPITYISETEFKRTVYKLKYAK 731
pfal|PF3D7_1134700   -------------------------------------------HTIYDDENIPIIVDSIPITYIGEKDFNRTVYKLKYAK 710
tetr|Q22C62          ------------------------------------------LSIVFPPTSVTIMLDGRVIGYIESKNAQTFCQSLKYLK 593
hsap|ENSG00000125630 ------------------------------------------PHR-SYSECYPVLLDGVMVGWVDKDLAPGIADSLRHFK 620
scer|YPR010C         ------------------------------------------FAA-GPSLC-CVQIDGKIIGWVSHEQGKIIADTLRYWK 612
cvel|Cvel_15870      ----NGP---------------------------------TDTESDERFETLPVLLDGEVVGRLPLGNAAETVERLRLWR 640
sneu|SN3_04700070    PNDGRGPLPLPPAHLSSSAPVPSSGTAAATAAAAARQSTSAGMQQQRLQGTLPLLVDGRVVAHLDRRDFVWWKELLSRFK 808
gnip|GNI_119110      ---------M--------------------------------SPAPGALGSYPIVVDGEPICRLPRETFDEMALAFKRLK 625
etht|ETH2_1434600    -------------------------------------------SEGGGINCVPLVVDGVVVCKIRKAEFSFWMEQLRHAK 663
tgon|TGME49_297530   ----RGPTGV--------------------------------VAATSSASTFPLVVDGAVVCRLDRKDFAFWLETFRDLK 676
vbra|Vbra_22055      --------------------------------------------------WAHIILDGRIIGRVQMELLERVSLSLRMWK 630
chot|ChTU502y2012_41 -------------------------------------------YSPDLTKSIPVMLDGRVLFHIPREDFMIWENKLRTLK 627
bmic|BMR1_01G02877   ---------------------------------------------RYVHKPVPVVVDGIPIARIPSDDAPQLMDALISAK 617
tequ|BEWA_006420     -------------------------------------------TSKDEKHYIPILVDGIPICTIPSSDSMNVYNQLRQAK 621

htar|Htart_000272800 NKN-LYN-LKASFEIIAHFK----ESA-LMDAIYINTTPGTLLRPVFNIKMKTIEFVTPSSQPYMSIALNYEELKKSKKI 769
pber|PBANKA_0913800  NHN-LYN-LKSHFEILAYLN----EQS-MMDSIIINTCTGRIIRPILNLKMKCIEFISPGYQPYVSIAVNYDDILKNNLA 804
pfal|PF3D7_1134700   NNN-LFN-LKSYFEINAYLN----EPL-IMNSIIINTFPGRLIRPLLNLKTKDIEFISPSYQPYISVAINNEDVKKNNLA 783
tetr|Q22C62          IMQDPTRLIPADLEIGYVPKLNLEKQN-IFPGIFLHTNEARFVRPVKHLKLNALEWITPFEQVHLSIACIEEDIRND--- 669
hsap|ENSG00000125630 VLR-EKRIPP-WMEVVLIPMT--GKPS-LYPGLFLFTTPCRLVRPVQNLALGKEELIGTMEQIFMNVAIFEDEVFAGV-- 693
scer|YPR010C         VEG-KTPGLPIDLEIGYVP-P--STRG-QYPGLYLFGGHSRMLRPVRYLPLDKEDIVGPFEQVYMNIAVTPQEIQNNV-- 685
cvel|Cvel_15870      MKEGKKQGVPWHMEMAYFPP----DKL-MFPALYLFTGPGRVVRPVRHLASRCIEYIGPLQQLFLNIACDEKDIERQQRC 715
sneu|SN3_04700070    REE-KWN-FLKYWEIAGFPP----NEG-HFEGIYVFSYPGRLIRAVRHIDSNSLDYVGPLMQPWTAIASRETDVRRDRRL 881
gnip|GNI_119110      NSG-RHG-FPSQAELCAFKK----GSG-AFEGIFLYSFPGRLVRPVRDVNTRQVEWIGTLAQPWLKIACSDKEVEEANAA 698
etht|ETH2_1434600    GCG-LDG-FKTHWELVGFDE----DSN-QFEGIFMFTMPGRLVRPVKHLASGRLEFIGPLQQPWMSIACRERNVLLNERS 736
tgon|TGME49_297530   SRE-AED-FKQHWEVVGFPH----NGG-NLESIFIFTFPGRLVRPVRHIESGRIEYIGPLMQPWAAIACRPSDVKRSDRL 749
vbra|Vbra_22055      ILG-FAG-LPEHMEVVAIGQ----NCGFYYPGLYLFTTPGRVVRPVRNIRAWRPEWIGSLEQGWLNIAVFETDIFDFSVD 704
chot|ChTU502y2012_41 WQT-NPSVLPVHAEIACVPP----DSG-MFSGIYIFTAPGRLVRPIMHLETAQVDWIGPLGQPWMNIGVTEEETRKST-- 699
bmic|BMR1_01G02877   LEG-IKG-IRKDFEIFSTCE----AKG-QMEYIYVLTFPGRMVRMVKNLKFDRVELIGPIAQQWSFIAINTAELDQHR-- 688
tequ|BEWA_006420     LNG-NFG-MKQHYEIIALPD----SKT-TYSNISVMTYPGRMIRPVRCVKTGNVEWIGPITQLWSTIAVSEYELSLSH-- 692

htar|Htart_000272800 RSFFKKKKKRGELAKKFTSSERMKLTLEQQYILY------------------------------------------LKEQ 807
pber|PBANKA_0913800  RKILKKKENIVNLNMNN-TKNTKKMTLKEQYHFY------------------------------------------TNKK 841
pfal|PF3D7_1134700   RKLLKKKEQVVNSMKID-RT-P--GSIKQKFLYH------------------------------------------QNRE 817
tetr|Q22C62          -------------------------------------------------------------------------------- 669
hsap|ENSG00000125630 -------------------------------------------------------------------------------- 693
scer|YPR010C         -------------------------------------------------------------------------------- 685
cvel|Cvel_15870      TEAAEAISIVQTPFAKRSA-----------EVMVRAEELGL----------------------------ADDEIEALV-- 754
sneu|SN3_04700070    LQLHEPVLQLLQQQQQNATA----ATAAAAATAATATATAATATATAAAATAAAATATAATAATAATAAAATAATATATG 957
gnip|GNI_119110      IEVQKRILLGLLARPENE--------KEPEPL------------------------------------------------ 722
etht|ETH2_1434600    MTAHQPLIEKILRDQIAEVD----NTEDT--V------------------------------------------------ 762
tgon|TGME49_297530   LRLHGPVVSEIVSSSVSTDS----LASRFQAL------------------------------------------------ 777
vbra|Vbra_22055      NLCLDMRQMKRLKDQKGADS----LTDKELLHICRSTDPKELSDKLDIIASQQ-------DV-------KIKEIKAAIRQ 766
chot|ChTU502y2012_41 ---------KLLEIQKR--------------------------------------------------------------- 707
bmic|BMR1_01G02877   ---------ARIKLEYN--------------------------------------------------------------- 696
tequ|BEWA_006420     ---------SILESE----------------------------------------------------------------- 698

htar|Htart_000272800 KREEK--------TQQSAKTEVVDNNVRGY---AEKY-------TNGTPLQKN--MNLNR---------------EKEV- 851
pber|PBANKA_0913800  HNI---EKE----AKNYNESEIVRKENKKASKSKNKV-------LNILENKKQ--YQFNE---------------DEYIS 890
pfal|PF3D7_1134700   NLLWKLKKESKDGSYKYEKHK-----------IDRKL-------LKILDQKKNNDSDTNT---------------DNYVS 864
tetr|Q22C62          -------------------------------------------------------------------------------- 669
hsap|ENSG00000125630 -------------------------------------------------------------------------------- 693
scer|YPR010C         -------------------------------------------------------------------------------- 685
cvel|Cvel_15870      -----QQDRQNAAR-----------------------------------ENP-------G-------------------- 767
sneu|SN3_04700070    KKEECQKEEVEAGRATKRKTAKVG---RGGGHATDKAAGEQREQAGGTKDSPEGEGPTGGAGEAAVAGATAAERLEEEEA 1034
gnip|GNI_119110      -----R--------------------------YKDLVK-----------------L------------------------ 730
etht|ETH2_1434600    -----VQD--NQGR-----------------LESDYQS-----------------L------------------------ 777
tgon|TGME49_297530   -----SVDSASQER-----------------HSEE--T-----------------G------------------------ 792
vbra|Vbra_22055      AKEERQAR--REGR------------------ERQKKKKKKQRAEGGGAGDPEVDVETSDS--------------EDEKR 812
chot|ChTU502y2012_41 -----------------------------------------------LIGISDTESEE---------------------- 718
bmic|BMR1_01G02877   -----------------------------------------------LSSNPQSNPQSNPP----------------SNL 713
tequ|BEWA_006420     -----------------------------------------------NVNKPHI-------------------------- 705

htar|Htart_000272800 ---FEN--------------------------------DTDSRNS---------------------SDYESDESDELSDI 875
pber|PBANKA_0913800  SSDYE-----------------------------------------------------------TSSESDKYSDDNMSST 911
pfal|PF3D7_1134700   SSEYDTDSDYDY----------------GYD---SNSDGDSKSNS----------------NFDSKSDNISGSDNNMSTT 909
tetr|Q22C62          -------------------------------------------------------------------------------- 669
hsap|ENSG00000125630 -------------------------------------------------------------------------------- 693
scer|YPR010C         -------------------------------------------------------------------------------- 685
cvel|Cvel_15870      ASEFPL----SHEIFQRAIQRSRAR-----D-KIIQEASTSSQKRPKKS--------KKKTSFADMFPDATTNP------ 823
sneu|SN3_04700070    AEEMALATRFAAVLHQQTLQEERQQ-------LLLQEEEDE-KGKQK--------------------AEGKTEP-----V 1081
gnip|GNI_119110      -------------------------------------FTEA-SKSAE--------------------AKAKIFK-----K 747
etht|ETH2_1434600    -------------------------------------FNGQ-CDSST--------------------PNCGRMD-----V 794
tgon|TGME49_297530   -------------------------------------ETGE-TGETG--------------------ESGKRFP-----L 809
vbra|Vbra_22055      AENMRI----EAVVELFRIAEDRKHTFEGLDVTMMQRVLDA-CPAPTHEKLCVADMMDRHMKVEDILSEGVYWDMRPKEP 887
chot|ChTU502y2012_41 ----QL----KN--DLKA------------------------------L------------------------------- 727
bmic|BMR1_01G02877   PS--NL----PSNLPPYP------------------------------S------------------------------- 726
tequ|BEWA_006420     AP--SI----SETLSLHR------------------------------N------------------------------- 718

htar|Htart_000272800 SELSQTDLEEIDLYEQIPEKYEYMELKETSFLSLVAALTPFSDHNQSPRNIYQCQMLKQTMGLQCLNTLYAFTEKIYRII 955
pber|PBANKA_0913800  TYIDSDENLNIDIYEQIPQKFEYMELKQTSFLSFLASLTPFSDHNQSPRNIYQCQMLKQTMGIQSLNMFYTFTNKIYRII 991
```

```
pfal|PF3D7_1134700   TYIDSDENINIDMYEQIPQKFEYMELKETSFLSFLASLTPFSDHNQSPRNIYQCQMLKQTMGIQSLNNVYTFTNKIYRMI 989
tetr|Q22C62          --------------------TEYQELSPSYILSILPSQIPFLNYNQSPRNMYQCQMAKQTMGTSMHNFPYRFDNRIYRLL 729
hsap|ENSG00000125630 --------------------TTHQELFPHSLLSVIANFIPFSDHNQSPRNMYQCQMGKQTMGFPLLTYQDRSDNKLYRLQ 753
scer|YPR010C         --------------------HTHVEFTPTNILSILANLTPFSDFNQSPRNMYQCQMGKQTMGTPGVALCHRSDNKLYRLQ 745
cvel|Cvel_15870      --EIRSLSNFPPYIENVAVDYSHVEVRPTSILSLLASLTPFSDHNQSPRNMYQCQMLKQTMAIPTYVTKHRHDNKLYKLD 901
sneu|SN3_04700070    TETEQAAAADLLFSHHVPLHHEYVELKPTAFLSIAASLTPYSDHNQSPRNIYQCQMLKQTTGTPFHSVPFRHDNKAYRLI 1161
gnip|GNI_119110      FGLTCSEAEQVALIQTCPLDYDYVEVSPTSFLSINASFTPLSNHNQSPRNMYQCQMLKQTMGTPFHNICNRTDNKTYRIL 827
etht|ETH2_1434600    --RGFQEVRDALVTSHVPVRYDYVELSPTAFLSVTAALTPFSNHNQSPRNIYQCQMLKQTMGTPFHSIPYRHDNKAYRLI 872
tgon|TGME49_297530   IAANEASGTDALFSFSAPVKYEYVELKSSAFLSIAASLTPYSHHNQSPRNIYQCQMLKQTMGTPFHSIPFRHDNKAYRLI 889
vbra|Vbra_22055      AGTVW-LDDEFDLYENSPVKYTHVEMKPSAMLSIMASLTPFSNHNQSPRNMYQCQMLKQTLGIPFHNTVYRFDVKSYHLH 966
chot|ChTU502y2012_41 --KKL-IKDNDRVLSSVPVNYTHMELHPSTILSVTASLIPYAHHNQSPRNMYQCQMLKQTMGTPCLNYPYRTDNKMYRLL 804
bmic|BMR1_01G02877   --FES-YKRIDKLLENIPVHYTHIEIEPHGILSICANCTPFSDFNQSPRNMYQCQMLKQSMGIPAHSQPLRSDGKLYNLI 803
tequ|BEWA_006420     --KEC-NTNVDKFLENAPVSYEFVELKPSGILSVIAALTPFSNHNQSPRNMYQCQMLKQSMGVPYHCETYRSDNKSYRLI 795

htar|Htart_000272800 TPQYPLVVTRDYELYGMDNYPTGTNAVVAILSYTGFDMEDALIILNKSSVDRGIFQCSVYKTERINLIKDFEN-----PNI 1030
pber|PBANKA_0913800  TPQFPLVVTRDYEFYGVDNFPSGTNAVVAILAYTGYDMEDALIINKASSDRGMFRTHIYKTEVIDLYKKTNS-----PDF 1066
pfal|PF3D7_1134700   TPQFPLVVTRDYEFYGVDNFPSGTNAVVAILAYTGYDMEDALIINKASADRGIFRTHIYKTEFIDLQKVGES-----SNF 1064
tetr|Q22C62          NGQIPMVKGMDPESYGLSDYPSGTNAIVAVISYTGYDMEDAMILNKSSYERGFGHGCVYKNYSYDVAELNKEKNSKFKML 809
hsap|ENSG00000125630 TPQSPLVRPSMYDYYDMDNYPIGTNAIVAVISYTGYDMEDAMIVNKASWERGFAHGSVYKSEFIDLSEKIKQGDSS---L 830
scer|YPR010C         TGQTPIVKANLYDDYGMDNFPNGFNAVVAVISYTGYDMDDAMIINKSADERGFGYGTMYKTEKVDLALNRNRGDPITQHF 825
cvel|Cvel_15870      QLQRPIVRTGEFEAARLSDYPSGTNAIIAVIPFTGYDMEDACILNKASVERGMFVGETTKTKIIDAAPPKSETA-EMRYW 980
sneu|SN3_04700070    TPQRPLVRTRDYRLYDFDDYPSGINAVVAVICYTCFDMEDAMVINKASVERGAFHSCVYKTKVVDATPESSKTA-ESTTY 1240
gnip|GNI_119110      TPNRPLVRNWDYRRYDFDFYGSGTNAVVAVISYTGFDMEDAMIINKHAMERGAFDGCVYKTKVVDAAPAGCRMI-DMRNF 906
etht|ETH2_1434600    TPQKPLLRTRDYRHIDFDDYPTGVNAVVAVMCYTGFDMEDAMILNKSSVERGLFHGCVYKTKIIDAAPSGSRAQ-DAEEY 951
tgon|TGME49_297530   TPQRPLVRTEDYRRFDFDDYPSGVNAVVAVMCYTGFDMEDAMILNKGSMERGAFHGCVYKSKVIDAAPASARAG-AASQY 968
vbra|Vbra_22055      TPQRPIVRTHEYDSYNFDTYPCGTNAIVAVISYSGYDMEDAMIMNKASMERGLCHASVYKTKVIEAAPPGLDIK-YASPY 1045
chot|ChTU502y2012_41 TPQEPTIITKGYSSFGFSEYPTGTNAIVAVMSYSGYDMEDAMILNKSSYDRGMFHACVYKTKIISAAPPSATKKSDAESY 884
bmic|BMR1_01G02877   TPQSPLVSTSDYRRLHLNNYATGANAVVAIMAYTGFDMEDAMVINKSSIERGFLRATLCRTKVLDAAPPGCSAK-EGHCY 882
tequ|BEWA_006420     TPQKPLVTTEEYRKMGYDDYSTGINAIVAVIAHSGFDMEDAMILNKASLDRGAFHGCIYKTKIIDAMPPSAVNK-DSHSY 874

htar|Htart_000272800 VFGNNERYELGNIMENSETDKTNSMNTNRGAKTSIEMDGFPSIQQTVDADSFLFSYIDTKTAL----------------- 1093
pber|PBANKA_0913800  VLGNNIRYNANSLSS-KN-----NI--REHKGKFLNKDGLPCINQKILENNPLYSYINKKNEL----------------- 1121
pfal|PF3D7_1134700   VFGNNIRYLNNNKNNNKN-----LA--YENKGKFLNKDGLPCVQQKITEGSPLYSYINKGNEL----------------- 1120
tetr|Q22C62          NEVKN-ID-------------------KSQIAEGLEGDGIPAIGSVIKKGTAVFQIYDTQKNA----------------- 852
hsap|ENSG00000125630 VFGIK-PG-------------------DPRVLQKLDDDGLPFIGAKLQYGDPYYSYLNLNTGE----------------- 873
scer|YPR010C         GFGND-EW-------------------PKEWLEKLDEDGLPYIGTYVEEGDPICAYFDDTLNK----------------- 868
cvel|Cvel_15870      RFGTSTMQ-------------------RGAEWEELDEDGFPPVSSKLTDGSLLSRSEYIGPAASA--------------- 1026
sneu|SN3_04700070    SFGNADCL-------------------GRKRAATLDSDGFLPISSRVRKGDVFCRAQATERGGGGGGDNREGEGKGKGDE 1301
gnip|GNI_119110      VFANRTAS-------------------GRKFDEGLELDGLPAIGTKLVRGSSLYRVEDIRLRAMD--------------- 952
etht|ETH2_1434600    QFTNTSKL-------------------GRKCIPSLDADGFPTIGAKITQGKTLCRVQRHTHS--T--------------- 995
tgon|TGME49_297530   TFGNVNEF-------------------GRKHIATLDGDGFPAIGAKIEKGNTYCRVQTSSSS--S--------------- 1012
vbra|Vbra_22055      RFNNLDAS-------------------GVRAVSSLDKDGLPFIGSKVTEGSPLYRIEHSATRV----------------- 1089
chot|ChTU502y2012_41 FFHNIGPD-------------------GELVVKELSADGLPNIGQRLTKGSPICRVERTGSGGGP--------------- 930
bmic|BMR1_01G02877   VFRGPSN-------------------------GPLDADGLVRVGQLIANGSPLARIEYAGTGAGA--------------- 922
tequ|BEWA_006420     YFNNINDA-------------------GNLIIKHLDKDGLPSVGQKVKKGMIFCRVEARERQNGE--------------- 920

htar|Htart_000272800 -----TSYEKYGKSGTYFIDTIGVAT---------------------------------------DENNHQFSTIKLRSH 1129
pber|PBANKA_0913800  -----TTYEIFKTHGDYYVDCVSNSQ------------------------------------------NNQIAIIRLRTT 1154
pfal|PF3D7_1134700   -----TTYESFRNHGEYFVDFVSIGK---------------------------------------N-NSNQVAVVKLRST 1155
tetr|Q22C62          -----MKTFYHKDSELGRLDGIALVHDE-----------------------------------KTKE---PRVEYRIRIP 889
hsap|ENSG00000125630 -----SFVMYYKSKENCVVDNIKVCSND-----------------------------------T-GSGKFKCVCITMRVP 912
scer|YPR010C         -----TKIKTYHSSEPAYIEEVNLIGDE-----------------------------------SNKFQELQTVSIKYRIR 908
cvel|Cvel_15870      --DKGARREIYKGDEDAFVSHVTKIGPKLKIPGRNDKIN------------------------FGLEPESERAMMTLRIL 1080
sneu|SN3_04700070    YGRQQVIEHEYKEGENGFVDGVVLSVPAAAASGSTAGAAAAPATSGAAAGAAAGATATAGTAAGGSEGSGCRASVRLRCP 1381
gnip|GNI_119110      GTADDVHSHAYNDDEEAFVEKVHLTNPDNDRGSR----------------------------YAATV-LCQRALITLRCI 1003
etht|ETH2_1434600    AVQAPALQTTYKDDEPAYVDGITLTSAPAPAEER----------------------------FLKTGLKGCRASLRLRSV 1047
tgon|TGME49_297530   SFASNGIQHAYKDGETAYVDGVTFISPPDAAHER----------------------------LAKSGLKGCRASLRLRCV 1064
vbra|Vbra_22055      -----AKTSYYNDEEDAFVERINRISPPDVAAYRKQI-S-------------------QVGKKGGGAFCAERVLLKLRCN 1144
chot|ChTU502y2012_41 -SN--ATIQTYHDDEVAFVEKINRIASGISIELQQDSLSG-----------------GGGDGASSSSGYGEKVSIKLRIV 990
bmic|BMR1_01G02877   ----RPRVEMYREEEPARVSSVTLVTP-----------NP-----------------SRID-KCVESAPCSRAIIRLQSV 969
tequ|BEWA_006420     -IHITERLEKYDDEEG-VIDQVTLVGF-----------SD-----------------SSADKSSSSGIKCTRVVIKLRIV 970

htar|Htart_000272800 RKPRIGDKFASRHGQKGVVSILFPHEDMPFSESGIVPDIIFNPHGIPSRMTIGMLIESICGKAASMYAKRIDATPFRKYL 1209
pber|PBANKA_0913800  RPPLVGDKFASRHGQKGVISRLFPHEDMPFSESGIVPDVIFNPHGIPSRMTIGMLIESICGKAASIYGKRIDATPFRKYT 1234
pfal|PF3D7_1134700   RQPQVGDKFASRHGQKGVVSRLFPQEDMPFAESGIVPDIIFNPHGIPSRMTIGMLIESICGKAASLYGKRIDATPFRKYT 1235
tetr|Q22C62          RNPIIGDKFSSRHGQKGVLSVLWPHIDMPFTESGITPDVIINPHAFPSRMTIGMLIESMAGKSGALNGNILETQPFQN-- 967
hsap|ENSG00000125630 RNPTIGDKFASRHGQKGILSRLWPAEDMPFTESGMVPDILFNPHGFPSRMTIGMLIESMAGKSAALHGLCHDATPFIF-- 990
scer|YPR010C         RTPQIGDKFSSRHGQKGVCSRKWPTIDMPFSETGIQPDIIINPHAFPSRMTIGMFVESLAGKAGALHGIAQDSTPWIF-- 986
cvel|Cvel_15870      RRPVVGDKFSSRHGQKGVVSMLMPQEDMPFTEQGIVPDILFNPNGFPSRMTMGMLIEIMAGKAAALHGVPFIDGSSFRKY 1160
sneu|SN3_04700070    RRPCLGDKFASRHGQKGILSLVWSHEDMPFSESGLVPDILFNPHGFPSRMTVGMLIEAMAGKAAAVHGAYQLPTFLCM-R 1460
gnip|GNI_119110      RRPILGDKFASRAGQKGILSLLYPPEDLPFSPSGIVPDLVFNPHGFPSRMTIGMVVESMAGKVAAITGDYQSSTPFDDYP 1083
etht|ETH2_1434600    RKPIIGDKFASRHGQKGILSMLWPHEDMPFSESGIVPDILFNPHGFPSRMTIGMLIESMAGKAAAVHGAFQDATAFREFR 1127
tgon|TGME49_297530   RKPVIGDKFASRHGQKGILSMLWPHEDMPFTESGLVPDILFNPHGFPSRMTIGMLIESMAGKAAAVHGAYQDATPFREFP 1144
vbra|Vbra_22055      RNPIVGDKFSSRHGQKGILSRLWPHEDMPFTESGLVPDILFNPHGFPSRMTIGMLIESVAGKVAAIHGTPKQDGTPFRRF 1224
chot|ChTU502y2012_41 RNPMVGDKFASRHGQKGILSMLWPQENMPFSESGMVPDILFNPHGFPSRMTIGMLLESMGGKAACIHGARSLDATPFNIN 1070
bmic|BMR1_01G02877   RVPDVGDKFASRHGQKGILSMTWRHEDMPFTESGIVPDILFNPHGFPSRMTIGMLIEALAGKSAAVTGEVGADGT----- 1044
tequ|BEWA_006420     RNPVVGDKFASRHGQKGILSMTWPVEDMPFLESGIVPDIIFNPHGLPSRMTVGKLIECMAGKSAAVHGQFQDATCFRKYP 1050

htar|Htart_000272800 KQK----------------------------------------------------------------------------- 1212
pber|PBANKA_0913800  KQK----------------------------------------------------------------------------- 1237
pfal|PF3D7_1134700   QQK----------------------------------------------------------------------------- 1238
tetr|Q22C62          -------------------------------------------------------------------------------- 967
hsap|ENSG00000125630 -------------------------------------------------------------------------------- 990
scer|YPR010C         -------------------------------------------------------------------------------- 986
cvel|Cvel_15870      QKL----------------------------------------------------------------------------- 1163
sneu|SN3_04700070    RNSLPRFSSTNSNTHEQVARPVRISRSAPAPPPHAVAAAAPEVITDAAAAPVVAAAPVVAAAPVVAAAPVVAAAPVVAAA 1540
gnip|GNI_119110      RNCIASG----TGQHKQ--------------------------------------------------------------- 1096
etht|ETH2_1434600    KQE----------------------------------------------------------------------------- 1130
tgon|TGME49_297530   PQS----------------------------------------------------------------------------- 1147
vbra|Vbra_22055      PKV----------------------------------------------------------------------------- 1227
chot|ChTU502y2012_41 ISD----------------------------------------------------------------------------- 1073
bmic|BMR1_01G02877   -------------------------------------------------------------------------------- 1044
tequ|BEWA_006420     KQS----------------------------------------------------------------------------- 1053

htar|Htart_000272800 --------------------------------------------------SVNNEWVDNGGLRGYLAANEQRQKEDSKKN 1242
pber|PBANKA_0913800  --------------------------------------------------SFNNPWIDGCGVKGFLNKLDENADEKKTTI 1267
pfal|PF3D7_1134700   --------------------------------------------------SFNNPWIDNCGIKGFLEKNDTIKSDLDTCQ 1268
tetr|Q22C62          -------------------------------------------------------------------------------- 967
hsap|ENSG00000125630 -------------------------------------------------------------------------------- 990
scer|YPR010C         -------------------------------------------------------------------------------- 986
cvel|Cvel_15870      --------------------------------------------------KTGNEWIDSDRVKDRRLRCSD---ERKDGS 1190
sneu|SN3_04700070    PDVPAASDFPAASDFPAAAPPGVVAAAPVVGAASDVPAAAPDVVAAAS-GVPAAAASDVPAAAGYMKRGERY-------- 1611
gnip|GNI_119110      ------------------------------------------RWLDGRGGRRDNRWIDQCGYNGWARRGNRY-------- 1127
etht|ETH2_1434600    -------------------------------------------------KTG-ARWIDQEGHHGYVLRGGRY-------- 1152
tgon|TGME49_297530   -------------------------------------------------ADGKNKWVDQGGYHGWVKRGERY-------- 1170
vbra|Vbra_22055      --------------------------------------------------RMNKPWLDNGGMRGLLTDKAKPIMKAVEGF 1257
chot|ChTU502y2012_41 --------------------------------------------------DL--------------S---R--------- 1077
bmic|BMR1_01G02877   ----------------------------------------------------------------------T--------- 1045
tequ|BEWA_006420     --------------------------------------------------KFNNRWIDQNGIKGWNERGQK--------- 1074

htar|Htart_000272800 SDRQQE----NR------------------------SNNQFSNQHNKNMKSSKPIFDDKIDYFAKLLLNKGYDYYGTECL 1294
pber|PBANKA_0913800  ------------------------------------------KGSTGNSDTTEISYDEKIDFFAKLLLNKGYDYYGTELL 1305
pfal|PF3D7_1134700   KEKDKNKNINNNNNNKIKENKYNNSNDSNASIDSDDNTNNCNDKRDEEEKKANITYDEKIDYFAKLLLNKGYDYYGTELL 1348
tetr|Q22C62          -----------------------------------------------------YQDDDIVNYFGKQLQSHGYNYYGNEVM 994
hsap|ENSG00000125630 -----------------------------------------------------SEENSALEYFGEMLKAAGYNFYGTERL 1017
scer|YPR010C         -----------------------------------------------------NEDDTPADYFGEQLAKAGYNYHGNEPM 1013
cvel|Cvel_15870      YL------------------------------L----------SEPPRPKEDEGEEETTGDYFGKALLAAGYQYYGKECL 1230
sneu|SN3_04700070    -------------------------------------------RTAEEEKKAAEEGDAAVDYFGKALMRAGYQYYGTEQL 1648
gnip|GNI_119110      -------------------------------------------LTDTEEAS-RADVDNPVDFFGKSLLYHGYQYYGNETL 1163
etht|ETH2_1434600    -------------------------------------------RTPEEEVKAQEQGDTPVDYFGKALLASGYQYYGKEEL 1189
tgon|TGME49_297530   -------------------------------------------RTPEEERQAQDEPETPVDYFGKALMRAGYQYYGTEEL 1207
vbra|Vbra_22055      FI------------------------------RPGKMGGQKRPGVPLDEEDKGDEWDDSIDYFGKALTQAGFEYYGTEPM 1307
chot|ChTU502y2012_41 --------------------------------------------SEENCEDDKAGEKTTVDYFGKALERAGFQYYGCEPL 1113
```

```
bmic|BMR1_01G02877   --------------------------------------------------FSHCTGSDPVAYFGSVLRRAGFRYYGTEDM 1075
tequ|BEWA_006420     --------------------------------------------YASDDEGDDTNHDDVVDYFGKTLLMAGYDYYGTEAM 1110

htar|Htart_000272800 YSGVYGVPMQAHIFMGVIYYQRLRHMV-----YDKAQARRLGPVCNLTHQPVKGKKRHGGMRMGEMERDGMISHGCSFTI 1369
pber|PBANKA_0913800  YSGIYGVPLQAHIFFGVIYYQRLRHMA-----YDKAQVRRTGPVCHLTHQPLKGKKKHGGIRLGEMERDGLISHGCSFII 1380
pfal|PF3D7_1134700   YSGIYGVPLQAHIFIGVIYYQRLRHMA-----YDKAQVRRTGPVCNLTHQPLKGKRKHGGIRVGEMERDGIISHGCSFVI 1423
tetr|Q22C62          YSGTYGCMMKVDIYMGIVYYQRLRHMV-----SDKAQARSTGPVDMLTQQPIKGRKRGGGIRMGEMERDALLSHGISFCM 1069
hsap|ENSG00000125630 YSGISGLELEADIFIGVVYYQRLRHMV-----SDKFQVRTTGARDRVTNQPIGGRNVQGGIRFGEMERDALLAHGTSFLL 1092
scer|YPR010C         YSGATGEELRADIYVGVVYYQRLRHMV-----NDKFQVRSTGPVNSLTMQPVKGRKRHGGIRVGEMERDALIGHGTSFLL 1088
cvel|Cvel_15870      YSGVTGEPMDADIFMGVVYYQRLRHMV-----NDKWQVRSTGPVDSTTRQPVKGRKRGGGIRVGEMERDSLIAHGSSFAL 1305
sneu|SN3_04700070    YSGVYGVPMLVHIFTGLIYYQRLRHMVGAKEVADKSQVRSTGPVDPLTRQPVKGRKKFGGIRFGEMEKDAVLSHGAAYVL 1728
gnip|GNI_119110      YSGIYGCEMQCQIFLGIIYYQRLRHMV-----SDKWQCRSVGPIDHLTRQPLKGRKRGGGVRLGEMERDALLGHGAAFML 1238
etht|ETH2_1434600    YSGVYGMPLTCHIFTGLIYYQRLRHMV-----TDKAQVRATGPIDSLTHQPVKGRKRHGGVRFGEMERDALIAHGATALL 1264
tgon|TGME49_297530   YSGVYGVPLTAHIFTGLIYYQRLRHMV-----TDKHQVRATGPVDALTHQPVKGRKRHGGVRFGEMERDALISHGAAALL 1282
vbra|Vbra_22055      YSGLFGGLMEADIFIGIVYYQRLRHMV-----SDKAQVRATGPVDMNTRQPVKGRKRHGGIRLGEMERDSLIAHGAAFCL 1382
chot|ChTU502y2012_41 YNGMTGVELPCHIFMGVIYYQRLRHMV-----ADKAQVRATGPIDALTRQPVKGRKRHGGIRFGEMERDSLISYGVSSIL 1188
bmic|BMR1_01G02877   YSGATGTTIKTSIFTGCIYYQRLRHMV-----ADKAQVRSTGPIDSLTHQPVQGKKRHGGIRFGEMERDSLISYGAPFLL 1150
tequ|BEWA_006420     YCGTLGTEIQAHIFVGCIFYQRLRHMV-----ADKAQVRATGPIDTITKQPVKGKKNLGGIRFGEMERDALIAHGTSQLL 1185

htar|Htart_000272800 SERFLYSSDLHECFVCPKCGLILSPILQYNVNGQV-----------KKGRS----IGGTKRMAICKACN----------- 1423
pber|PBANKA_0913800  NERFLMNSDGHECFVCPKCGLILSPILQYNCSGKL-----------IKGRS----IGGHSKMAVCKSCN----------- 1434
pfal|PF3D7_1134700   NERFLMSSDGHECFVCPKCGLILSPIMQFNTTGKI-----------MKGRS----IGGKSKMAVCKSCD----------- 1477
tetr|Q22C62          HDRFLNCSDYSEGYVCNSCGSLVSTYLHTNLMSGEAID-------SFYNYN----KGVQKSKPKCRKCQ----------- 1127
hsap|ENSG00000125630 HDRLFNCSDRSVAHVCVKCGSLLSPLLEKPPPSWSA---------------------MRNRKYNCTLCS----------- 1140
scer|YPR010C         QDRLLNSSDYTQASVCRECGSILTTQQSVPRIGSI-------------------------STVCCRRCSMRFEDAKKLLT 1143
cvel|Cvel_15870      LDRLFHCSDDHRCYCCPRCGSIQTPIFANRMEVDGV-PARGVTPVEFAGLGVIGPNQRPIADPICSACG----------- 1373
sneu|SN3_04700070    QDRLLHCSDGHKTYCCPSCGNILSAAAPFVEAAAEEKPELKLK--KQLRRN----LGGTRFVPLCKVCR----------- 1791
gnip|GNI_119110      QDRLLNCSDGHLSYVCPKCGSILSPCL---------------D--TVKSRS----AGGKQKIPICRVCE----------- 1286
etht|ETH2_1434600    QDRLLHCSDAHRAFCCPCCGSILSPFQS--------------S--DMKGRS----VGGRKPVPLCRICG----------- 1313
tgon|TGME49_297530   QDRLLHCSDAHKTFCCPSCGSILTPSAV--------------P--NLRGRT----AGGKRAVPFCRVCQ----------- 1331
vbra|Vbra_22055      HDRLMHCSDEHKTYVCPSCGSFTSPVMPLRVSEME-----------RHGLK----SAALRPVPRCQACK----------- 1436
chot|ChTU502y2012_41 VDRLLICSDEHRAYVCPKCGLIISSISKRAMAQSI-----------SSSKL----SNCIPPIAVCRVCK----------- 1242
bmic|BMR1_01G02877   QDRLLNCSDLHLACICPECGSLLSASTGPNDSQDM---------------------RDTTKPPVCTVCS----------- 1198
tequ|BEWA_006420     QDRLMHCSDGHFAYVCPKCGSIISATISSVNSN-----------------------TGKIQISQCRICK----------- 1231

htar|Htart_000272800 ---------------------------VSCNLILIPYVL---------------RYLMNELICLNVVIRLNVKTVEQLL- 1460
pber|PBANKA_0913800  ---------------------------VSCKIVYVPYVL---------------RYLLNELVCLNITIRLNVQSIESIF- 1471
pfal|PF3D7_1134700   ---------------------------VSCKIIFIPYVI---------------RYLLNELICLNVTIRLNMKSVENVF- 1514
tetr|Q22C62          --------------------------NAECSKVVIPFVL---------------RYLTNELAAMNIKLAFKLE------- 1159
hsap|ENSG00000125630 -------------------------RSDTIDTVSVPYVF---------------RYFVAELAAMNIKVKLDVV------- 1173
scer|YPR010C         KSEDGEKIFIDDSQIWEDGQGNKFVGGNETTTVAIPFVL---------------KYLDSELSAMGIRLRYNVEPK----- 1203
cvel|Cvel_15870      ---------------------------VKCPIIAIPYIF---------------RYLCEELTGLNVGLKLRLETAPGVAY 1411
sneu|SN3_04700070    ---------------------------LPCRLVIIPFSLSFSSVARYAAMEDTVIYLINELAGMNVAVRLNLADYGRRV- 1843
gnip|GNI_119110      ---------------------------VPCRRANLPYVF---------------RYISNELASMNIAIALQVTECDREI- 1323
etht|ETH2_1434600    ---------------------------VPCRLVQLPYIF---------------R------------------------- 1326
tgon|TGME49_297530   ---------------------------VPCRLIMVPYVF---------------RYLANELAAMNVTIRLKLAEFGQPL- 1368
vbra|Vbra_22055      ---------------------------VPCRLVALPFVL---------------RYLCAELAAINVHVKLDLSSRGERI- 1473
chot|ChTU502y2012_41 ---------------------------SPCRIITLPYIF---------------RYLSNELAAMNIVIRLHLQQEDVPI- 1279
bmic|BMR1_01G02877   ---------------------------VPCHWVRIPYVL---------------RYLANELASINVGLKFTLRQFGHAV- 1235
tequ|BEWA_006420     ---------------------------VGCKLVMIPYVL---------------RYVANELATMNIGIKLHLSQLGTPI- 1268
```

Figure S3.3. RPA12 (trimmed)

```
chot|ChTU502y2012_40 INA-TKQRNSN------FPKFQFSFSKGDNFNSQLYGGCPSCGNSYDFLFLLDAFENEYT-------------------- 98
gnip|GNI_090920      ------------------------SAELDHFDYYARNGCPNCGAEFQFLLETD--------------------------- 100
etht|ETH2_1359700    --A-------LEGERSATSSCSITSKHGVCFDWLVALGCLNCGAPVELRRDLVAADEACAA-------------AAAAAK 143
tgon|TGME49_250060   ESAQPRRRPADARSLVGVA-CS----QGVSYDWLLALGCLNCGRSVTVADDLLPLLEEED-------------------- 163
srcn|SRCN_4945       EGAATKREDEERIAFVETSCCSDGQRNTPSYDWLYALGCLNCGKPLTLAADLLPSAEAATAGRSETSGCRPHTGTTTAGK 275
cvel|Cvel_23889      -----------------------------------RASKKQCDEVMVEHEWGRQVMYWWCHVP-----DRPM-------- 251
vbra|Vbra_96         VGGGRPVRRQRRTQRASY--PR-----------------------------------FYKEEG-----ERKK-------- 1286
bmic|BMR1_03g04085   ------DTECSRNNIRAK--PKYIINNSRSYNYLIARGCPQCSRDVDFSSP-NEVFN----------------------- 97
tequ|BEWA_040210     RGSGHADNNADEEEMDAG--ERDTNMDPVSYDYLVAVGCRFCDSSIDFKSA-YMFFDY---EG-----PNN--------- 137
htar|Htart_000304900 -----------ENAIITF--PKYS-ISEKNIQREKNDYCKNCGYIYNYKDYTYMYLMFLKKNK-----AQNS-------- 292
pber|PBANKA_1004900  -----------KNS-ADI--THQS-IMIKSIQKEKKQFCKHCGYIFNYDDYNYLFIKRFNMSK-----INYD-------- 246
pfal|PF3D7_0407300   -----------KNA-TEI--TQYD-LKNKNIQKNKQSYCKTCGYIYNYDDYMYLFMKYFQLLN-----IKN--------- 198
tetr|Q22CT1          ----------------------ME-IDIKNLN-TASEFCPQCGNMIEMPLY----------------------------- 27
hsap|ENSG00000066379 ---------------MSV--MDLA-NTCSSFQ-SDLDFCSDCGSVLPLPGA----------------------------- 32
scer|YJR063W         ----------------------------MSVV-GSLIFCLDCGDLLENPNA----------------------------- 22

chot|ChTU502y2012_40 ----SPSHNLIENHYLRCKICNYRFLKLHNHNSSD-STIGTN-------------------------------------- 135
gnip|GNI_090920      --------IPLQGLALACQTCGHRCAPMHTEGDSE-SVADRLDP------------------------------------ 135
etht|ETH2_1359700    ECSAKALATAAEKAVLRCRRCGHDIAPLHSSDESE-SLVERMESGA---------------------------------- 188
tgon|TGME49_250060   --------RLDASAALVCRGCGHHIAPLHGANESD-SLVDCMQQSRRRHQAEAADAELNGERKTDVKRENKGANRGDGGE 234
srcn|SRCN_4945       DCSSSSGQSTEERPAVVCSNCGHRVCPLHEANEPE-TLADAGLLPDGSS-------------------SSDSSSRGGGGC 335
cvel|Cvel_23889      ---RQM--GMYPSELH----EPRALTDEDLETRAVQKQVDKAEIR----------------------------------- 287
vbra|Vbra_96         ---KGTGGGR---QHVK-AQTDFLVNDEKDRGRAQ--MRDAPPEV----------------------------------- 1322
bmic|BMR1_03g04085   ------------SGKICCVDCGYIIAPAILSSTRE-D---LL-PV----------------------------------- 125
tequ|BEWA_040210     -------GFVQPNEFLRCQTCNSIIAPIFKKGDSD-SLVDSL-RA----------------------------------- 173
htar|Htart_000304900 -----V--GTNNAIKLYCIYCGSYINNIHTYED----------------------------------------------- 318
pber|PBANKA_1004900  -----DNNPVLCENTIKCIYCGYIIEDLDINENIN--------------------------------------------- 276
pfal|PF3D7_0407300   -------HNEFSYDIIKCIYCGSVLGDIEILFNQF--------------------------------------------- 226
tetr|Q22CT1          ----------S--DKVECSKCEFLCSVLEYKCAPI--------------------------------------------- 50
hsap|ENSG00000066379 ----------Q--DTVTCIRCGFNINVRDFEGKVV--------------------------------------------- 55
scer|YJR063W         ----------VLGSNVECSQCKAIYPKSQFSNLKV--------------------------------------------- 47

chot|ChTU502y2012_40 ----------SNSSEKKRIY--ITGIRKSLCFGTNNKWWAKSISVGKRI-------ERDSDNKTGKSNSVIRVL-DAMHK 195
gnip|GNI_090920      ----------EYIGGRGGIY--LRGSRKMHDFGQNNKSWWQNYYLPKKV-------RHELQD-------LL--SNNELTS 187
etht|ETH2_1359700    --------NAKPGVVSGDIY--MYGSRRCYNFGSVSKQSWQQRFLGAEF-------EQQLLQ------HHLKIMLSAGDG 245
tgon|TGME49_250060   LGETANAATASRALTEDGVY--LFGQRKQHRLGDFSKRWWQQRFLGERF-------GERLLE------QQLRLVQAFSGA 299
srcn|SRCN_4945       S-------NKKSFGTSDGVY--LFGLRQQYVTGEWGKRFWQQRFLGDSF-------QQQQLQ------QRLRLVEAVGKE 393
cvel|Cvel_23889      ----------HAKWPRKGI----DSVRHTFTLMEVYKQF-----------DITGGLDKAIFMNT-VDGEMRRFRLAAYQN 341
vbra|Vbra_96         ----------DEYWGQQSVTCAPHVIEEEWMYMPEYWRRWREWGHPACPVTLCRGLSYPILSHP--------FI-IDDKY 1383
bmic|BMR1_03g04085   ----------D-SFDDKSLY--LFGCRESYVYDQQGKDLWKDYLNETNI----S-------LLE--------MA-KSSFS 172
tequ|BEWA_040210     ----------D-EADAQSVY--LYGTRSGYVFEESGKRWWKSHMNSALL-----------------------DA-KSAKK 216
htar|Htart_000304900 ------------SKKQKYIE--VDSYRTKILYDQNEKKIWKEKLTLF-N----K-------GSA--------IV-SDTKY 363
pber|PBANKA_1004900  ------------YNTNNYIE--LHSYREKYLFDNNKKTYWQNKIATF-D----K-------NIE--------LF-KEGES 321
pfal|PF3D7_0407300   ------------NNNENYIE--LYSYREKYIFDKNKKDYWKNKITSF-N----K-------NTS--------LF-KEEEK 271
tetr|Q22CT1          --------------------------VSRIEF--NQKKQWLEQYRASQN----K--KIHGIEEE--------DL-EDKHK 87
hsap|ENSG00000066379 --------------------------KTSVV------------FHQ-----------LGTAMPM--------SV-EEGPE 77
scer|YJR063W         --------------------------VTTTAD--DA---FPSSLRA-----------KKSVVKT--------SL-KKNEL 76

chot|ChTU502y2012_40 KKAPKIQEICPECSHNEAFFTQFQARSADEGTTVMYECCKCQHRRVINN 244
gnip|GNI_090920      NKGQRTKETCDACGHDEAFFTTFQARSADEGATILYECTRCHSRKTFNN 236
etht|ETH2_1359700    GNKTTCREICERCGHNEAFFSTFQARSADEGMTVMYECTKCHHRRVFNN 294
tgon|TGME49_250060   KKGVVCKETCEACGHGEAFFSTFQARSADEGMTVMYECVKCGHRRVFNN 348
srcn|SRCN_4945       KQGVVCKDTCEKCGNKEAFYSSFQARSADEGMTVMYECTRCGHRRVLNT 442
cvel|Cvel_23889      DMRTPYHVPCEKCGHKVQWMKTMQLRSADEGMTVFLQCVKCKVITITNN 390
vbra|Vbra_96         EQYRYICEECPRCGHPRMRFATKQCRGMDEGMTVLYECPLCEYRFVQND 1432
bmic|BMR1_03g04085   KGTQTVKEICQKCGNEEQYYSTFQARSADEGMTIMYECTSCGNRTTFNV 221
tequ|BEWA_040210     SGKQTVKQICEKCGHDTHTFSTFQARSADEGMSVMYECTKCGNRLVIAT 265
htar|Htart_000304900 DTYNITYDPCTNCGHSFLYYTNIQTRSADEGSTIIYLCPQCKKKTTVNN 412
pber|PBANKA_1004900  GAYNITYEKCTDCDHDFLYFVNIQTRSADEGSTIIYFCPNCKKQTTVNN 370
pfal|PF3D7_0407300   TAYNITYEKCLDCGNDFLHFINIQTRSADEGSTIIYFCPNCKKQTTVNN 320
tetr|Q22CT1          HKKATLKQECPDCGHDTLYFWTVQTRSADEGSTVFYECQDCKFKFTANN 136
hsap|ENSG00000066379 CQGPVVDRRCPRCGHEGMAYHTRQMRSADEGQTVFYTCTNCKFQEKEDS 126
scer|YJR063W         KDGATIKEKCPQCGNEEMNYHTLQLRSADEGATVFYTCTSCGYKFRTNN 125
```

Figure S3.4. RPAC1 (trimmed)

```
cvel|Cvel_24657        ------------------------MPVKVKKEEETGLPKLEETAPSSSSA---AGAERDAPKDQVSSELDPCGILHGFQL 53
bmic|BmR1_04g08170     ------------------MIPD--------IESTTPRIKLSPNGVNP--------------------VWNRYSDSCGVVD 34
htar|Htart_000383500   DKDIDKDINKIKKKKRNKTYPRDAANKMSEVKGKDKYVQLTQEGPCNASTTYFHGTYSLL-------HGEKYFDRDEFKN 153
pber|PBANKA_0905700    ---------------------------MSNIKYKDQFVKMGQEGPRNVTTTNFYGSYFLT-------ENENFFDIKKFEE 46
pfal|PF3D7_1143300     ---------------------------MENVKYRDNFVQLGEEGPRNATTTNFYGSYYFS-------DKENYFDIRKFEE 46
chot|ChTU502y2012_40   -------------------------MVSEKKERNNRYLECTTEGPRNVGGYGYIGSNELN-SIIK-GDNYRKQTVEEIIE 53
tequ|BEWA_026680       ---------------------------------MPNFVYLDASGIKYSY-----DKFT----------DQNAPSVLEEIK 32
vbra|Vbra_22425        ------------------MDEDEERPLPAHLQQHKSRVICGVEGPRYTESYEYAGTFSSM-------HHDDAFSLEGFKD 55
gnip|GNI_141390        ------------MGEYLIFRPEKMEGLPSHLQAQVKELVCGLEGPRDVESITSASTFAAM-------GVCPDYDMKKLVK 61
etht|ETH2_1306500      -------------------MPAGKGAPRGP-HGLPNRVQCTLEGPRESP---------------VVSHETEVFDLGTFEK 45
sneu|SN3_02800130      SSRAAS--SSSASSASCSSSSSTSPSATGSSLVHSNRLVCEAEGPRYTSSGCYAGAFLSS-SFPSAACLEEVFSLEQLQK 95
tgon|TGME49_267390     -----------------MSLPGHEGRPSASIPLHSNRLVCAAEGPRHTNSGGYAGAFLSS-SY-----VDDVFSIDKFCE 57
tetr|Q235X0            --------------------------MSNKTQKMKKNIVFTNEGIQHSQEMNYS---A---------AFTSEEDFNTFKE 42
hsap|ENSG00000171453   ------------------------MAASQAVEEMRSRVVLGEFGVRNVHTTDFPGNYS---------GYDDAWDQDRFEK 47
scer|YPR110C           ---------------------------------MSNIVGIEYNRVTNTTSTDFPGFSK---------DAENEWNVEKFKK 38

cvel|Cvel_24657        KFPDNCISDN--GLTLSFEIRGVHVGVVNALRRAIIAEVPTVMVDTIHIFQNTSDFSDWKINHRVALTPLVIDPDNLEHW 131
bmic|BmR1_04g08170     NLAIKFLKRE--FDHSIIEIKGLDVSFVNAIRRILIAEVPSLAIETVMVWQNTGLIHDDVLAHRLALVPFNVDPEEINY- 111
htar|Htart_000383500   NLKINILEKR--EDFIQFEIINIDASLANAFRRIMIAEVPTIAIETVTIFQNTGIIADEILCHRLGLIPFQFDADLINY- 230
pber|PBANKA_0905700    NLEMKIIKNE--ENLLILEIKNLNVSIANALRRIMISEVPTIAIEKVNIFQNTGVIADEILCHRLGLIPFKFDADLINF- 123
pfal|PF3D7_1143300     NLEMNVIKNE--ENILILEIKNMDVSIANALRRIMLSEVPTIAIEKVNMYQNTGIIADEILCHRLGLIPFKFDADLINY- 123
chot|ChTU502y2012_40   TIDIKVNELK--SDYINFDISGIDASIANALRRILLVEIPTVAIETVQIYQNNGVIQDEVLAHRLGLIPMLIDPNTIEF- 130
tequ|BEWA_026680       NVKIRFIKNT--KFHSVVEFEGLNVSIANALRRILIAEIPTLAIETVEIRQNTGVVQDELLAHRLGLVPFNVDPEDFSY- 109
vbra|Vbra_22425        ELEVRMLTRT--DELVRFEMRGIDAALANAFRRILIAEVPTMALHTIQVYQNTGVLCDEVLVHRLGLIPIRLDPQHFEY- 132
gnip|GNI_141390        RMSIDMRQKG--EERVLFDLKGVDVAMANALRRVMIAEVPTMAIETVHLYQNTGVLQDEVLCHRLGLVPFKVEPETYKY- 138
etht|ETH2_1306500      NLKIKILECK--PQIVVLELKGCDASLANALRRILISEVPTAALETIQIWQNTGVVQDEVLAHRLGLIPFKVNPKKLKF- 122
sneu|SN3_02800130      KTKIKFLEVS--SDRVVFELTGLDAAIPNALRRILISEVPTVAIETVQIWQNTGVMQDEVLAHRLGLIPFRVDPSAIDY- 172
tgon|TGME49_267390     RTSLKFLSVS--PERVVFELKGVDAAIPNALRRILISEVPTVAIETVQIWQNTGVVQDEVLAHRLGLIPFIVDPTALNY- 134
tetr|Q235X0            NIKIEVIRYDNKNKELEFDLMGVDAPIANALRRIMIAEVPTMAIHKVYIYQNTSIIPDEVLAHRLGLIPIYADPRLFEE- 121
hsap|ENSG00000171453   NFRVDVVHMD--ENSLEFDMVGIDAAIANAFRRILLAEVPTMAVEKVLVYNNTSIVQDEILAHRLGLIPIHADPRLFEY- 124
scer|YPR110C           DFEVNISSLD--AREANFDLINIDTSIANAFRRIMISEVPSVAAEYVYFFNNTSVIQDEVLAHRIGLVPLKVDPDMLTW- 115

cvel|Cvel_24657        YYKNTEPELKVYTEKNGVNFRLHVVGTED---------------------MRKVYSRDLKWAPRTNAERQKWKR------ 184
bmic|BmR1_04g08170     -RGH-----ENLNESNSVCFELKVAHDANCI-----------------EKYQSVYTSQLKWIPLGDEQHKRLGK------ 162
htar|Htart_000383500   -REE----SEKHNEDNCFCFKLHVKCKPL-----------------GDQKTMNIYSKDLIWVPLNANQKAQFQN------ 282
pber|PBANKA_0905700    -KDT----HEKYNNLNCFCFKLHVKCNSINN------------NKIGNENYQCIYSKDLKWCPLNEEQKIKFES------ 180
pfal|PF3D7_1143300     -KED----NEKYNHLNCFCFKLHVKFSS---------------KRTGNDNYQSIYSKDLKWCPINEQQRIKFEK------ 177
chot|ChTU502y2012_40   -RNE----DEDLNEKNSVCFKLNVKCTKDDI------------NHEKGITSKAIYSKDLVWIPLSDNQRKKFGK------ 187
tequ|BEWA_026680       -KNH-----ESLGDKNSLCFKLSAKCLRSDS-------------QGR--NTLSLYAKDLVWHPFNEAQKLKFQD------ 162
vbra|Vbra_22425        -RRE----NEEHSAENSVKFKLHVKCDQG---------------------VRSVYSRDFVYEPLSDLQRQKFPPPQPSRH 186
gnip|GNI_141390        -RTE----NEELTAENSLCFKLHIVCTPENL------------DQEK--KCLPVYSRDFVWVPLSEQQKEQYP------- 192
etht|ETH2_1306500      -RNP----NQELSEENSLKFKMHVKCSPGDL------------KPHQ--KSMPVYARDLKWIPMSERQKQKFAQ------ 177
sneu|SN3_02800130      -RQP----NTEWTEENSLLVSLHVKCTEQSL------------APDQ--YSLPVYARDIVWEPLSPAQKEAFAH------ 227
tgon|TGME49_267390     -RQP----NQEVTDENALRVSLHVKCTEQSL------------GPHQ--TSLPVYAKHIKWEPMSPSQKEAFAA------ 189
tetr|Q235X0            -KQE----EDEYNEKNSLKFTLKIKCSKKEQYKNYSEADLENLTPETYLENAIVYSGDFKWVSQGE-QNKKFEE------ 189
hsap|ENSG00000171453   -RNQG---DEEGTEIDTLQFRLQVRCTRNPHA------AKDSSDPNELYVNHKVYTRHMTWIPLGN-QADLFPE------ 187
scer|YPR110C           -VDSNLPDDEKFTDENTIVLSLNVKCTRNPDA------PKGSTDPKELYNNAHVYARDLKFEPQGR-QSTTFAD------ 181

cvel|Cvel_24657        ------------------------------DPPRVANPDILLAVLFKGQEIDLHAIATKGKGKMHTKWSPVGTASYQMMP 234
bmic|BmR1_04g08170     ------------------------------NQPRPLFEDILLTKLAPGQQIELKAYLEKGIGRTHAKWSIVSSATYRFYP 212
htar|Htart_000383500   ------------------------------NPPKVVDTNILITKLATGQEIELICFLEKGIGKTHAKWSPVSTATYRMKP 332
pber|PBANKA_0905700    ------------------------------NPPKVVDDNILITKLGNGQEIELICFLEKGIGKTHAKWSPVCTAVYKMFP 230
pfal|PF3D7_1143300     ------------------------------NPPKVVDENILITKISSGQEIELICFLQKGIGKTHAKWSPVCTAVYKMYP 227
chot|ChTU502y2012_40   ------------------------------SPPKVVTDDILITKLRPGQSIEAILYCEKNIGRVHAKWSPVCTASYRLLP 237
tequ|BEWA_026680       ------------------------------DPPRPIHGDILLTKLAGGQELDLTAYLEKGIGKTHSKWSPVSTAVYRFKP 212
vbra|Vbra_22425        TPAAAAAASAASAAGATDEQAVELYDGEVYPPPAPVYPDILITKLGAGQEIELEAYCEKGVGKDHAKWSPVATAVYRLFP 266
gnip|GNI_141390        ------------------------------EPPRPVSEDYLITKLRPGQEIEAVCYLEKGIGKTHAKWSPVCPAVYRLHP 242
etht|ETH2_1306500      ------------------------------DPPAPVHPDILITKLRPGQEIELFGFLEKGLGKTHAKWSPVATAVYRLEP 227
sneu|SN3_02800130      ------------------------------CPPAPVHPDILITKLRPGQEIELKAYLEKGVGKTHAKWSPVCTAVYRLEP 277
tgon|TGME49_267390     ------------------------------NPPRAVHPDILITKLRPGQEIELKAYLEKGVGKTHAKWSPVCTAVYRLEP 239
tetr|Q235X0            ------------------------------NPIRMVHDNIIVAKLRENQEIICEVICQKGIGKTHAKWSPVCTAYYRLMP 239
hsap|ENSG00000171453   ------------------------------GTIRPVHDDILIAQLRPGQEIDLLMHCVKGIGKDHAKFSPVATASYRLLP 237
scer|YPR110C           ------------------------------CPVVPADPDILLAKLRPGQEISLKAHCILGIGGDHAKFSPVSTASYRLLP 231

cvel|Cvel_24657        DISFPRGSFFPEKMAEDLV-KCCPTGVFELGEEHSAEGGGRKKGKTKVKAEERQKTTATHSAAAGATESAEEEDIEDFGG 313
bmic|BmR1_04g08170     HIDISS--QLTRDESKELV-ATCPMNVFALDHKGN--------------------------------------------- 244
htar|Htart_000383500   LFIFNEPETLTKAEKYDLV-KLCPKNVFDIEDGDQ--------------------------------------------- 366
pber|PBANKA_0905700    TFDFDVHEKFTNYEKKDIL-SICPKNVFDIEESGN--------------------------------------------- 264
pfal|PF3D7_1143300     HFSFNTNEQLSYEEKKDLV-NICPQKVFDIEDSQQ--------------------------------------------- 261
chot|ChTU502y2012_40   EFGFPNG-PISGSDALKLRDTVCPMKVFDIEDVTG--------------------------------------------- 271
tequ|BEWA_026680       EITFSEEFPLSKSDKEELC-ELCPMDVFSMKKGD---------------------------------------------- 245
vbra|Vbra_22425        ELVFPKG-PLVDAEAQELK-DLCPMNCFDIEDTPT--------------------------------------------- 299
gnip|GNI_141390        KIIFPQG-VLRGPEAEDLV-NLCPMNVFDIEN--G--------------------------------------------- 273
etht|ETH2_1306500      EFVFTS--PIEGEEAKELK-ELCPMGVFDIEDSTG--------------------------------------------- 259
sneu|SN3_02800130      EIVFKK--PFRGEEAKELC-SLCPMGVFDVEDTSG--------------------------------------------- 309
tgon|TGME49_267390     EIVFKE--SIQGEDASDLC-SLCPMGVFDIEDTTG--------------------------------------------- 271
tetr|Q235X0            EININS--DITGAKAQELK-ERCPLKVYDVQNN----------------------------------------------- 269
hsap|ENSG00000171453   DITLLE--PVEGEAAEELS-RCFSPGVIEVQEVQG--------------------------------------------- 269
scer|YPR110C           QINILQ--PIKGESARRFQ-KCFPPGVIGIDEG----------------------------------------------- 261

cvel|Cvel_24657        LRLREVKVRDPYKCTSCKNCTE---EYPNHVKLKKVTDRFKFTVESVGSLPVDRIVKDGLRVLWQKNDDVLQDVIGLDKA 390
bmic|BmR1_04g08170     -----VIVEDPLKCTSCRACIE---RFK-QVTITKVTNHFIFSIETCGPISSWDAFVKSVNILKNKGDKLKCKLLE---- 311
htar|Htart_000383500   -----IIAKNILNCSSCRACIE---KYPTKVKFNKMKHHFIFSVESTGCFSAQDIFLKAVRILKKKCIRLKEQIRDKME- 437
pber|PBANKA_0905700    -----LVAKNPLDCSSCRACIE---KYPQKISFQKKKNHFIFTVESTGCFSSANIFKKALNILKEKVMNVKEMLEEQT-- 334
pfal|PF3D7_1143300     -----IFVKNPLSCSTCRVCIE---KYPKKISFQKEKNHFIFTIESTGCFSAADIFKKALFILRQKVINVKEALDEQLCP 333
chot|ChTU502y2012_40   ----NAVPTNPRNCTTCRACIE---KFPNKIKLLKDKFHFIFSIETTGCIPPVELVKTAIDILRKKGEKIKSSVKLFYEQ 344
tequ|BEWA_026680       -----ICIEDTLSCTSCRACVE---RFPKSVIVKKLNNHIIFSIESTGAVPSATLLLRAIEVLKNKSIRLMNRISELG-- 315
vbra|Vbra_22425        --GVESVVTNVRQCTTCRVCME---RFPGRVQVNKIKNHFIFNIESTGCIPADQLFERAVDVLRHKCAETFDMLKANCPD 374
gnip|GNI_141390        ----EAIVAKPTQCTTCRACIE---RFPTQVEVRKIKDHFIFQVEATGSIPAPDVFKRSLSVLIQKLQKIKNNIEKDIQA 346
etht|ETH2_1306500      ----RAYAKFPRNCTTCRACLE---RFENQLQLNKIPDQFIFSIESTGSVPAPELFEMAVEVLLEKAITFREIIRTKQLE 332
sneu|SN3_02800130      ----EAYARYPRNCTTCRACIE---RFPKAIELRKIKDHFIFSIESTGAVPAPVLFKMALEVLKEKAITFSHIIDCKLAA 382
tgon|TGME49_267390     ----EAYARYPRNCTTCRACIE---RFPKEIELRKIKDHFIFSIESTGSVPAPLLFKMAIEILKEKALTFRQIIDLKLVS 344
tetr|Q235X0            ----KAIVSNERACTMCRECIREDEEFNSKIELAKKKEHFIFTVESVGIYEPHEIVKEAIQILKQKASSFMLNL------ 339
hsap|ENSG00000171453   --KKVARVANPRLDTFSREIFR-NEKLKKVVRLARVRDHYIFSVESTGVLPPDVLVSEAIKVLMGKCRRFLDELDAVQMD 346
scer|YPR110C           --SDEAYVKDARKDTVSREVLR-YEEFADKVKLGRVRNHFIFNVESAGAMTPEEIFFKSVRILKNKAEY-LKNCPITQ-- 335
```

Figure S3.5. RPAC2

```
hsap|ENSG00000186184 ----------------------------MEEDQELERKISGLKTSMA--EGER----KT--ALEMVQAAGTDRHCVTFVL 44
scer|YNL113W         -------MTEDIEQKKTATEVTPQEPKHIQEEEEQDVDMTGDEEQEE--EPDR----EK--IKLLTQATSEDGTSASFQI 65
cvel|Cvel_13599      ---------------M----MDPSKVPPAEKAGEDQERMTDKKD-----------------ETAQEHHHGNKSRNASIFV 44
chot|ChTU502y2012_40 --------------------------------------MEDVSMKEETEHGNNSVNLVS----KNEMLSNLEGPNLTFQI 38
gnip|GNI_081780      ----------------------------------------------------MSINYLKEVDDPITKEKALKSSLITISI 28
tetr|Q22RW2          MSDSEENSFQEIEQED----DEAVNNPDIEEEEEDEDYMMEDKPKK-----------QSAEEKLVIDQNEKDRTCATYTF 65
vbra|Vbra_19110      ----------------------------------------------------MAQPRA---EMDIPDDLGDDQTCATFQF 25
etht|ETH2_1429800    --------------------------------------MAPVTAFASSSPASSGSPSSS--SSSSSSSSSSSSRSLTVCL 40
sneu|SN3_02000375    --------------------------------------MVHTKESIR-ATGSSSEPIAEALDE-KEANSQATSSQLTFTF 40
tgon|TGME49_261540   --------------------------------------MADDGAGGS-SCGASSSPSGKAWTAGVHEQEACKASQLTFTL 41
tequ|BEWA_008340     --------------------------------------------------------------------MSDSSPNITFSI 12
bmic|BMR1_02g02925   --------------------------------------------------------------------MKDLSPNLTFCI 12
htar|Htart_000034900 --------------------------------------------------------------MNNKQKNLRNLKQATFCF 18
pber|PBANKA_1027500  ------------------------------------------------------------MEEKSYFQNLKTITHATFCF 20
pfal|PF3D7_1415200   ------------------------------------------------------------MEEKKYYENLKNLTHATFCF 20

hsap|ENSG00000186184 HEEDHTLGNSLRYMIMKNPEVEFCGYTTTHPSESKI-NLRIQTRGT--------------------------------LP 91
scer|YNL113W         VEEDHTLGNALRYVIMKNPDVEFCGYSIPHPSENLL-NIRIQTYGE--------------------------------TT 112
cvel|Cvel_13599      RGERDTLGNALRNMLLRHPEVEYCGYTPSHPSGQTMFTLRLQTT-G--------------------------------RP 91
chot|ChTU502y2012_40 HEEDHTLGNALRHLAMLNPNTTFAGYAIPHPSISTM-NLRIETISNVEVDDKNYNLDLKAALSPSLDKKSSSKKQSSKVG 117
gnip|GNI_081780      SGEDHTLGNLLRSRLSQMPEVEFASYSIPHPSLSEM-NFRLQVNEG--------------------------------LV 75
tetr|Q22RW2          YDEDHTLGNTVRYMLVKRKDVEFCGYTIPHPSENKM-NLRLQTI-K--------------------------------KN 111
vbra|Vbra_19110      VNEDHTLGNVVRYMLMKNPDIEFAGYSVPHPTENIM-NLRVQTT-E--------------------------------RP 71
etht|ETH2_1429800    PGETHTLGAVLRTLLLQSETVEFAGYSVPHPSQCSM-NLRVQTT-D--------------------------------TP 86
sneu|SN3_02000375    TGEDHTLGNALRSILVRKPGVQFAGYSIPHPNQPEM-NIRLQTT-G--------------------------------EP 86
tgon|TGME49_261540   TGEDHTLGNTIRSILVRKPGVEFAGYSVPHPTQPEM-NIRLQTT-G--------------------------------ES 87
tequ|BEWA_008340     ENEDHTLGNVLRTLLVERDDVVFAGYSVPHPMQPEM-NIRIQTT-G--------------------------------QP 58
bmic|BMR1_02g02925   ENEDHTLGNVVRTILSKKKDVSFAGYTMPHPMQYQV-NIRVQTT-G--------------------------------KP 58
htar|Htart_000034900 ENEDHTLGNSLRCLILKKKGVEFAGYTVPHPAIPEV-NIRIQTD-G--------------------------------ES 64
pber|PBANKA_1027500  ENEDHTLGNCLRCILLQKEGIEFAGYTVPHPTQAEI-NLRIQTT-G--------------------------------KP 66
pfal|PF3D7_1415200   ENEDHTLGNCLRCILLQKEGVEFAGYTVPHPTQPEI-NVRIQTT-G--------------------------------KP 66

hsap|ENSG00000186184 AVEPFQRGLNELMNVCQHVLDKFEASIKDYKDQKASRNESTF------------------------------- 133
scer|YNL113W         AVDALQKGLKDLMDLCDVVESKFTEKIKSM------------------------------------------- 142
cvel|Cvel_13599      AVSILRESLVLLKEVFAHFDKTFVKEFAEMRDRMEREKSQDGGQ----KEKEKEEKDDSGDVIMS-------- 152
chot|ChTU502y2012_40 ATQILLKACDDLNLICNKIEDKFDKAVQVHLSSN--------------------------------------- 151
gnip|GNI_081780      ALDVFKQALDDLIYLSKGISKEFNEKYGQFDTTMADTTTAD-------------------------------- 116
tetr|Q22RW2          SNEVLKEGLDSLAGLCDVLNDKFDAAMKKFEKQQKKKSK---------------------------------- 150
vbra|Vbra_19110      ASSVLAESLTNLMAVCLHVKRTYRKALRIHSKYQQQQ-----------QQQEGE-GGGASAAAAAAAGGGSSS 132
etht|ETH2_1429800    ALEALQKAFEQLRGICQVLSSKYQLAMQHFKQQQQQQQQQQQQQQQQQQQQEGE-GEAAGEDIDADE------ 152
sneu|SN3_02000375    AMRLLRESLEDLANICDHLDFVYDEALAEFYEKRRAATKSATSA----------------------------- 130
tgon|TGME49_261540   AMQLLRESFDDLASICDHLDTLYDEALEDFMKKQGESSEMTS------------------------------- 129
tequ|BEWA_008340     AVEVFSQSLGDLVEICDTLTDAFKKAMDEAMLTD--------------------------------------- 92
bmic|BMR1_02g02925   AIQAMVESLDEMQQVCDQILGEFNRALNTG------------------------------------------- 88
htar|Htart_000034900 AFEIMEESLSDLASVCDIVTEKFKEALTNYK------------------------------------------ 95
pber|PBANKA_1027500  AINILKDSLDDLSNICTILLEKFKNALESA------------------------------------------- 96
pfal|PF3D7_1415200   AIDILKESLDDLGNMCDIMLNKFNEALKQS------------------------------------------- 96
```

Figure S3.6. RPABC1

```
tetr|Q24FV0           -------MSEDQRKSAQKWFKVYRTICEMARDRGYSEEAEQQLNLIKTKEQENMWIQNSMSNVQN--SLTINSFSHDANQ 71
scer|YBR154C          -------MDQENERNISRLWRAFRTVKEMVKDRGYFITQEEVELPLEDFKAK---YCDSMG--RPQRKMMSFQANPTE-- 66
hsap|ENSG00000099817  ---------MDDEEETYRLWKIRKTIMQLCHDRGYLVTQDELDQTLEEFKAQ---SGDKPSEGRPRRTDLTVLVAHND-- 66
vbra|Vbra_3035        ----------MTDDDVTRLWRCRRTIFQMLQDRDYMVGRNCLLESREDVSQA---LS----EV-RNRESLVIFVRHRS-- 60
cvel|Cvel_10624       MAAAGVGGSEQDQVLLGKLTRARRTVYQMLKDRGYKVPESCLEETRETVEQE---FR----QNEEKLEKMIILVSRPS-- 71
gnip|GNI_113430       ----------MSDDYVRRFWRCRKTCCEMLEDRGYIVSQHEKNEAFPDFMRR---FD----EANRQRGQMLLLGSLRT-- 61
htar|Htart_000326500  -----------MEDPCAKFYKCRKTCFEMLEDRGYLVTTNEKSITYTEFKEK---FE----ENERLRSRMPIVTCRKD-- 60
pber|PBANKA_1140600   -----------MEDPVTRFYKCRKTCCEMLEDRGYIITAREKLENFAAFKEL---FE----ENEKLRSRMTIITSHKN-- 60
pfal|PF3D7_1364800    -----------MEDPVTRFYKCRKTCCEMLEDRGYIMTPREKLENFSTFKEM---FE----DNDRQRSKMGISTTHKN-- 60
chot|ChTU502y2012_42  -----------MDSSDNRFFRARRTLCEMLEDRGYIVSSQDKEEDFSTFKER---FE----SHQRLRSRMLMVASHRQ-- 60
etht|ETH2_1131600     -----------MESSIRRLFRARRTCCEILTDRGYLLPAQEMAEGFAEFAQR---FN----ENEQSRSRMLLIASHKA-- 60
sneu|SN3_02100055     -----------MEDSTRRLFRARRTCCEMLQDRGYIVPAQEKNEPWQDFLQR---FQ----ENERNRSRMLLVAAHKA-- 60
tgon|TGME49_240590    -----------MEDSTRRLFRARKTCCDMLQDRGYIVSAQEKNESWNDFLQR---FK----ENECNRSRMLLIASHKV-- 60
bmic|BmR1_04g05035    -----------MEDAEVRLFRCRRTCCEMLEDRGYFIPIQEKLEILSEFKSR---YE----MYDKIRSKMLLVASMRN-- 60
tequ|BEWA_011640      -----------MDDEEGRLFRCRRTCCEMLEDRGYFIPSQEKLETFAEFKAR---YE----SYDRTRSKMLLVASQKT-- 60

tetr|Q24FV0           TDEHKFLGMEQHLYVFHSQTKLTDDILSSYDKTIQKYHESSIKNNPQLNVFINVVILVPEEKDIVTKKVQDIILISNKYF 151
scer|YBR154C          ESISKFP-DMGSLWVEFCDE--P----SVGVKTMKTFVIHIQE-----------------------KNFQTGIFVYQN-- 114
hsap|ENSG00000099817  ------D-PTDQMFVFFPEE--P----KVGIKTIKVYCQRMQE-----------------------ENITRALIVVQQ-- 108
vbra|Vbra_3035        ------D-PTETLVVYFPDEQGK----KVGVRPIANLVSKMEE-----------------------KNIPRAILVCRE-- 104
cvel|Cvel_10624       ------D-SEDKLLVFFAES-SK----KVGVLPIRKLTDQMTE-----------------------NNINKAILVVTH-- 114
gnip|GNI_113430       ------D-ENQKILVYFSDE-PK----KTGVRPIKELSDKMEE-----------------------RGISSAILVALN-- 104
htar|Htart_000326500  ------D-PNSKIIVFFADE-TK----KTGIKPLRELTERMDE-----------------------KAIQRAILVTQN-- 103
pber|PBANKA_1140600   ------D-ANNKIIVYFVDE-VK----KTGVKPLRELTEKMDE-----------------------KSIQRAILVTQN-- 103
pfal|PF3D7_1364800    ------D-SNNRIIVYFADE-VK----KTGVKPLRELTERMEE-----------------------KSIQRAILVTQN-- 103
chot|ChTU502y2012_42  ------D-QTKRIICYFADE-SK----KTGVKPIRDIVEKMDE-----------------------HAIQRAILISQN-- 103
etht|ETH2_1131600     ------D-PEAKLIVYFADE-TK----KTGVKPIRELTERMEE-----------------------RNIHRAILVTQN-- 103
sneu|SN3_02100055     ------D-PDNRVIVYFADE-TK----KTGVKPIRELTERMEE-----------------------RNIQRAILVTQN-- 103
tgon|TGME49_240590    ------D-PDNRVIVYFADE-TK----KTGVKPIRELTERMEE-----------------------RNIQRAILVTQN-- 103
bmic|BmR1_04g05035    ------D-PNDKIIVYFADE-TK----KTGVKPIRELTERMED-----------------------HGIHRAILVTQN-- 103
tequ|BEWA_011640      ------A-SENKVLVFFADE-TK----KTGVKPIRELTERMED-----------------------HDIHRAILVTQN-- 103

tetr|Q24FV0           KDRIELFEKRQVEMNFNSKKQTDIKSETDDKGDVIQIFYKYEIFFFDDLVINITKHQLVPKHVPLNAIEKKKLMDKYKIN 231
scer|YBR154C          --NITPSAMKLV----------------------P-SIPPATIETFNEAALVVNITHHELVPKHIRLSSDEKRELLKRYRLK 171
hsap|ENSG00000099817  --GMTPSAKQSL----------------------VDMAPKYILEQFLQQELLINITEHELVPEHVVMTKEEVTELLARYKLR 166
vbra|Vbra_3035        --KLTSFAKEAL----------------------SMSSSKQRIEVFMESELLVNITEHELVPRHVPLSEVDKKALLERYKVR 162
cvel|Cvel_10624       --PLTPFAKEAI----------------------NEMKPKHNIETFQMSELMVNITHHELVPKHIPLSKEDKERLLEKYKVK 172
gnip|GNI_113430       --SMTAFARNAI----------------------AELAPQRHIEYFMEAELLVNITRHELVPRHIPLSDEEKEELLAQYKIR 162
htar|Htart_000326500  --TLTPFAKDAI----------------------NESAPRHIIENFLDTELLVNITKHELVPKHIPLTVEEKRHLLQRYKIR 161
pber|PBANKA_1140600   --TLTPFAKDAI----------------------KEAAPRHIIENFLDTELLVNITKHELVPRHIPLTSDEKRNLLQRYKIK 161
pfal|PF3D7_1364800    --ILTPFARDAI----------------------KEAAPRHIIENFLDTELLVNITKHELVPRHIPLTSDEKKNLLQRYKIM 161
chot|ChTU502y2012_42  --VLTAHAKVAI----------------------LDAAPKHYIESFLENELLVNITKHELVPKHILLSDDEKLQLLDRYKIK 161
etht|ETH2_1131600     --VLTAFAKDAI----------------------AEAAPRHIIEYFMESELLVNITKHELVPKHVPLTPDEKQQLLQRYRVK 161
sneu|SN3_02100055     --VLTAFARDAI----------------------SEAAPRHIIENFMESELLVNITKHELVPKHVPLTAEEKQSLLNRYRVK 161
tgon|TGME49_240590    --VLTAFARDAI----------------------SEAAPRHIIENFMESELLVNITKHELVPKHVPLTNDEKQNLLNRYRVK 161
bmic|BmR1_04g05035    --VLTPFAKEAI----------------------SEAAPRNIIENFMETELLVNITKHELVPKHVPLTAEEKRNLLQRYKVK 161
tequ|BEWA_011640      --VLTSFAKDAI----------------------MEASPRNIIENFMETELLVNITRHELVPKHVPLTMEEKQNLLQRYKVK 161

tetr|Q24FV0           DAQLPKILSTDPITKYFGLRTKQVFKIERDSETAGKYITYRIVS- 275
scer|YBR154C          ESQLPRIQRADPVALYLGLKRGEVVKIIRKSETSGRYASYRICM- 215
hsap|ENSG00000099817  ENQLPRIQAGDPVARYFGIKRGQVVKIIRPSETAGRYITYRLVQ- 210
vbra|Vbra_3035        ADQLPRITRDDPVAKYYGLERGQVVKIIRPSETAGRYVTYRLCI- 206
cvel|Cvel_10624       EFQLPRMLHTDPIARYFNLEKGSVVKIIRPSETAGRYVTYRLISS 217
gnip|GNI_113430       DIMLPRILPTDPVARYFGLRRGQVVKIIRPSESAGRFVTYRLCT- 206
htar|Htart_000326500  ETKLPKIQSKDAVCRYLGLSKGQVVKIIRPSETAGKYVTYRLVI- 205
pber|PBANKA_1140600   ENKLPRIQDVDPVCRYFGLSKGQVVKIIRPSETAGRYVTYRLVV- 205
pfal|PF3D7_1364800    ENKLPRIQDVDPVCRYFGLSKGQVVKIIRPSETAGRYVTYRLVV- 205
chot|ChTU502y2012_42  ETQLPRIQYADPVARYFGLAKGQVVKIIRPSETAGRYVTYRLVV- 205
etht|ETH2_1131600     EAQLPRIQVADPVSRYFGLSRGQVVKIIRPSETAGRYVTYRLVV- 205
sneu|SN3_02100055     EVQLPRIQCADPVARYFGLNRGQVVKIIRSSETAGRYVTYRLVV- 205
tgon|TGME49_240590    EIQLPRIQSADPVARYFGLSRGQVVKIIRPSETAGRYVTYRLVV- 205
bmic|BmR1_04g05035    ESQIPRIQSADPVARYFGLAKGQVVKIIRPSETAGRYVTYRMCF- 205
tequ|BEWA_011640      ENQIPRIQSADPVARYFGLSKGQVVKIIRPSETAGRYVTFRLVV- 205
```

Figure S3.7. RPABC2

```
hsap|ENSG00000100142 ------------------MSDNEDNFDGDDFDDVEEDEGLD---------------DLEN-------AEEEGQENVEILP 40
tetr|I7M6R9          --------------MSDYGDNDYSDPGNFDEDNMGDDMDDGDQESEE-ASSRSGDEGDSADDSDHSGTESESQSEVDKDE 65
cvel|Cvel_15670      MADAE---------------EGFQNFDGEDFEGDGE------------MGDEEGM-GEDDMGGDGGGYDD---EAYDVIH 49
htar|Htart_000137000 --------MDG----------YNDGYGND-DDLLGDEFGQ----------------GIDSDDGNNYD------YDIDIIA 39
pber|PBANKA_0401900  --------MDI----------YNDNYGNDEEDYMGDEFGQ----------------GVDSDEGENYE------NDIDIIA 40
pfal|PF3D7_0303300   --------MDG----------FNDNYLNEDEDFMGDEFGQ----------------GIDSDDGENYE------NDIDIIT 40
bmic|BMR1_02g02970   -MDDEF----G----ETYMDEFGEAFGEDEEDIFDEDFDEVNDV------DLG-----------VKR------GDVDIIT 48
tequ|BEWA_008480     -----M----G----DIYMDEFGENFGIDEFAEE-EEYEE-YDD------DEQ-----------HRV------ADVDIIT 42
scer|YPR187W         --------MSDYEEAFNDGNENFEDFDVEHFSDE-ETYEEKPQFKDGETTDANG--KTIVTGGNGP----------EDFQ 59
vbra|Vbra_13295      --------MDAEAELD----DFLGEDEDQELEDA----EEDLG----NLMDEGGEGEEEQAGGEGGG------RDAEVHP 54
gnip|GNI_004920      --------------MADLGDDFDMGMEDDVFDDI---LQEE-------------D-GLELPEDTPDG------EETEIVP 43
chot|ChTU502y2012_40 --------MNDYDESGGNY-DEEEEYMEEELIE---------------EEIE------------GD-------E--NIEI 35
etht|ETH2_1123500    MADDENDQMFAE----PFGDDFGDELGEEDFEED---FEEARAA---AAA--------------AD-------PDLDVLV 49
sneu|SN3_01300865    MADDEADHMFAGEGAGGFGGDDFEDFGEEELPDE---FEEHAVV---AATAAGGG-GLSGAGGTGD-------QELDVIV 66
tgon|TGME49_270780   MADDEIDHMFAGEP-GGLGDDFGEDFGDDELIDD---FDVERAA---AAA--------------SH-------EPESDIL 52

hsap|ENSG00000100142 ------------SGERPQANQKRITTPYMTKYERARVLGTRALQIAMCAPVMVEL-EG-------------------ETD 88
tetr|I7M6R9          YPEEMVI--IKDQDRDQQASKTKVTPPFLTKYEKARIIGTRALQIAQNSPIYVETDDK-------------------DYD 124
cvel|Cvel_15670      ----------EDENAELKPSKERITSPFMTKYEKARIIGTRALQISMNAPVTVDVG-D-------------------LTD 99
htar|Htart_000137000 DNQTKKD-KSDNEQSDRNEENVRITSPYLTKYEKARIIGTRALQISLNAPLTIPIDTQNENI-HK---SE--YDNYVCND 112
pber|PBANKA_0401900  DNQIKKE-KYDYENCEGNEENIRITSPYLTKYEKARIIGTRALQISLNAPLTIPIETQNDKSNGK---SD--YDNYLNND 114
pfal|PF3D7_0303300   DHQIKKDNKSDYENSEANEDNIRITSPYLTKYEKARIIGTRALQISMNAPLTIPIETSNDMINSK---NE--YDNYLNND 115
bmic|BMR1_02g02970   DPNDPRV---HV------SSSNRITSPYLTKYEKARIIGTRALQISLNAPITIPFDFGEQDT------IDSASMVDAAID 113
tequ|BEWA_008480     DPAEFKA---QI------DNAHRITSPYLTKYEKARIIGTRALQISLNAPITIPIDSTDAGDDGMHSVGDFGDTQTAAID 113
scer|YPR187W         QHEQIRR---KTLKEKAIPKDQRATTPYMTKYERARILGTRALQISMNAPVFVDL-EG-------------------ETD 116
vbra|Vbra_13295      -----EG---QVPDTEAREVKDKMTSPFMTKFEKARVIGTRALQISMGAPITVDA-EG-------------------ETD 106
gnip|GNI_004920      AENALPS---HRPGTKA-ATGPRQTSPYMTKFEKARVIGTRALQISLGAPVTVPI-EG-------------------ESD 99
chot|ChTU502y2012_40 LTEDAVH---SRYDGKP-NEGPRITTPYMTKFEKARIIGTRALQISMNAPVAIPL-DG-------------------ETD 91
etht|ETH2_1123500    DPNDPRL---QRPEARP-NEGPRLTSPYLTKFEKARVVGTRALQLSLSAPLTVSA-EG-------------------ETD 105
sneu|SN3_01300865    DPNDPRA---QRPDARP-SEGPRVTSPFITKFEKARILGTRALQISLNAPVTIPLLEG-------------------ETD 123
tgon|TGME49_270780   DPSDPRA---ARPHARP-SEGPRITSPYITKFEKARVIGTRALQISMNAPITIPL-DG-------------------ETD 108

hsap|ENSG00000100142 PLLIAMKELNARSAS------EPPGEEPPWMDRGTRDEHLPSCPGCPAVASNTRCCPPAACLPGISLSRRRRLREE 158
tetr|I7M6R9          PIAIAEKEFVQKKIPFIIRRYLPNKEYEDWALDELEVLD------------------------------------- 163
cvel|Cvel_15670      PLAIAEKELHAGTLPFIIRRYLPNGAFEDWPLEDMEVPE------------------------------------- 138
htar|Htart_000137000 PLVIAEKELYNKSIPFILRRYLPDGSYEDWKLDELIID-------------------------------------- 150
pber|PBANKA_0401900  PLVIAERELYNKSIPFILRRYLPNGSYEDWRLDELIID-------------------------------------- 152
pfal|PF3D7_0303300   PLVIAEKELYNKSIPFILRRYLPNGSYEDWRLDELIID-------------------------------------- 153
bmic|BMR1_02g02970   PLVIAEKELYQKTIPFTIRRYLPNGSYEDWKIQELIID-------------------------------------- 151
tequ|BEWA_008480     PLIIAERELYQKTVPFIVRRYLPNGSYEDWRIEELIID-------------------------------------- 151
scer|YPR187W         PLRIAMKELAEKKIPLVIRRYLPDGSFEDWSVEELIVDL------------------------------------- 155
vbra|Vbra_13295      PLVIAEKELMSKSIPFVIRRWLPDGTYEDWTIKELHIDGI------------------------------------ 146
gnip|GNI_004920      PLVLAEKELIAKTMPFMIRRYLPNGEYEDWTIEELLVD-------------------------------------- 137
chot|ChTU502y2012_40 PLLIAEKELYTKKIPFVIRRYLPNGNYEDWKIDELILD-------------------------------------- 129
etht|ETH2_1123500    PLLLAEKELRQKTLPFRIRRYLPDGSFEDWRLDELLID-------------------------------------- 143
sneu|SN3_01300865    PLVIAEKELYQKTIPFTIRRYLPDGSYEDWKLQELIID-------------------------------------- 161
tgon|TGME49_270780   PLIIAEKELYQKTIPFTIRRYLPDGSYEDWKIQELIVD-------------------------------------- 146
```

Figure S3.8. RPABC3

```
hsap|ENSG00000163882 ----MAGILFEDIFDVKDIDPEGKKFDRVSRLHCESESFK-MDLILDVNIQIYPVDLGDKFRLVIASTLYEDGTLDDGE- 74
tetr|I7LTT4          --MSKSDILLEDRFKLEEIKKEE-KFSKVERVVLVSDN--NIKIEIDIHTDLYSMKKGNQYWVMITSSLGNFQEDKGIF- 74
scer|YOR224C         ----MSNTLFDDIFQVSEVDP-G-RYNKVCRIEAASTTQDQCKLTLDINVELFPVAAQDSLTVTIASSLNLEDTPANDSS 74
cvel|Cvel_9667       ---MASTSLFEDTFSTDAIND-E-VFQNVARLTCHSVAYE-TELVLDINSQLFPVSKSDQLIIKLASSIKDK-------- 66
gnip|GNI_101140      MAPSQSRILLEDRMTVKSMDS-S-KFEKVTRISGRSAAFD-AELTIDINTNVYPLKEGQVLTFAFA-------------- 63
vbra|Vbra_16380      --MALSPHLLDDAFKVKSVDN-S-KFEKVSRVKCEGMSLE-LEMTIDINTDIFPINENEVVKIALNDKLAPPDVEGGQY- 74
htar|LSRZ01002649    ---MLTTAIFEDRFVVDSVDN-S-KFEKVSRIKAKSTGYD-AELIIDICTELYNLEPRNTISLSIRDNPLYENGVKR--- 71
pber|PBANKA_1429500  ---MASNILFEDRFVISNVDN-S-KFEKVSRIKAKSTGYD-AELILDVHSELFKVEEKKAIYLALQDNFMGKNDDKT--- 71
pfal|PF3D7_1213700   ---MASNILFEDRFVISSVDN-S-KFEKVSRIKAKSTGYD-AELILDVHSELFKVEEKKAIYLALQDKLLNRNDEKG--- 71
chot|ChTU502y2012_30 --MSLPSCLFEDRFVINSVDS-G-KFDRIGRIRGRSVGFD-ADLILDINNELFPVREKESLYIGLSSQVSVNPNDIV--- 72
bmic|BMR1_02g00620   ------MVLFEDRFIVRGIDN-S-KYERVTRITAKSTGFD-AEIVLDVNSDLFPVRENTTLHILLTNNLS-TGDSGNIE- 69
tequ|BEWA_032210     --MATASCVFEDRFLVRSIDK-S-KFERVARISAKSTGFD-AELLLDVNSDLLPVNNKAMLHILITNSLLPSGSDVNLS- 74
etht|ETH2_0950600    ---MSIPCLFEDRFTVRNVDN-G-KFERVSRIRAKSSGLD-ADLILDVHNQLLPVAEKQGLHIGLTNSLSGPDRKGD--- 71
sneu|SN3_01000245    ---MSVACLFEDRFEVRSVDN-S-KFERVSRIKGKSSGFD-ADIQLDINSDLFPVREKQRLYIGLTNQLRQ-GSRKDDA- 72
tgon|TGME49_233720   ---MSIPCLFEDRFEVRSVDC-S-KFERVARLKGKSSGFD-ADLQLDVNSDLFPVKEKQRLYIGITNNLHSSGDARKDD- 73

hsap|ENSG00000163882 -----YNPTDDRPSRADQFEYVMYGKVYRIEGDETSTEAAT------RLLRLRAAEWQCSRITGWGLLFQLCVRVLWGPA 143
tetr|I7LTT4          -----VSEEFDRESIKDKFEYVMHGKIYNMNSDNKAKTVSIFASFGGLLMKVSGKDEDLKNFNVDSRIYILIRLS----- 144
scer|YOR224C         ATRSWRPPQAGDRSLADDYDYVMYGTAYKFEEVS-KDLIAVYYSFGGLLMRLEGNYRNLNNLKQE-NAYLLIRR------ 146
cvel|Cvel_9667       --------SGTDHNVLKKWDYCMYGKVFRIDEEN-QKKVTVYASFGGLMMSLKGQPRYLNSIQNNKRLYILMKKQGSDET 137
gnip|GNI_101140      --------DQGKESLMEDYEYVCLGRVFHEDDKS-AEKRAVYISFGGLLCQLIADKAIVSEFTMDKRLFFFLRRST---- 130
vbra|Vbra_16380      ----F---WDKGGDVMDMWDYIMYGKIFKYQEKG-DEKVNLYASFGGLLMSLSGDRIHLENLQLDTRIYFLLRRDH---- 142
htar|LSRZ01002649    ----A---TPDATTIFDSADYIMHGRVFKFEELS-SERR----------------------------------------- 102
pber|PBANKA_1429500  ----W---EQADNKSLNNIEYIMSGRIFKFEELS-SERRTVYASYGGLMMALTADKQFIGDLEIDMKIYLLAKNVDIEIV 143
pfal|PF3D7_1213700   ----W---EQNENVPLNNIEYIMSGRIFKFEELS-SERRTVYASFGGLMMALTTDKQFIGDLEIDMKIYLLTKNIDIERT 143
chot|ChTU502y2012_30 ----W--DNSGPQTLMDQYDYVMYGKIYRVEEKQ-SDRRSLYASFGGLLMSLTADKNLVGDFSLDMRLYCLVRRSEDII- 144
bmic|BMR1_02g00620   ----E---INDFGSLVGNYEYAMYGKIFKFEEIS-SERRCLYTSFGGLLMSLTADKQVILDLELDMRIYLLAQRVDYRN- 140
tequ|BEWA_032210     ----E---CNDIPTLLGDYEYAMYGKIFKFEEIS-SETRTIYASFGGLLMSLTADKQVVADLELDMKIYLLVRRITANR- 145
etht|ETH2_0950600    --------GAADSQVLSGYDYVMYGKVFKLEETS-SERRTLYLSFGGLLMSLAADKHVVGDLELDMRVYLLVKRSPEALA 142
sneu|SN3_01000245    ----A--AWAASLPFLQEYDYVMYGKIFRLEESS-SERRTLYASFGGLLLALTADKHVIGDLELDMRIYILIRRSDDVNP 145
tgon|TGME49_233720   ----S--AWTEPAAYLKEYDYVMYGKVFRTEECS-SERRTLYASFGGLLMALTADKHVVGDLELDMRIYILIRRSDEVHS 146

hsap|ENSG00000163882 HEAAGGCQQPAWIRGGLQSLSPDEEASLLNLA 175
tetr|I7LTT4          -------------------------------- 144
scer|YOR224C         -------------------------------- 146
cvel|Cvel_9667       MHF----------------------------- 140
gnip|GNI_101140      -------------------------------- 130
vbra|Vbra_16380      -------------------------------- 142
htar|LSRZ01002649    -------------------------------- 102
pber|PBANKA_1429500  -------------------------------- 143
pfal|PF3D7_1213700   RE------------------------------ 145
chot|ChTU502y2012_30 -------------------------------- 144
bmic|BMR1_02g00620   -------------------------------- 140
tequ|BEWA_032210     -------------------------------- 145
etht|ETH2_0950600    D------------------------------- 143
sneu|SN3_01000245    VLS----------------------------- 148
tgon|TGME49_233720   LLA----------------------------- 149
```

Figure S3.9. RPABC4

```
tetb|EI9_05168.1      ------------MNQQDSEKNINFSAQSVRYICGYCGKEVVLEETQRTRIKCSHCARNILYKKRRDDKKPFQ-----YEAI--- 64
hsap|ENSG00000147669 --------------MDTQKDVQPPKQQPMIYICGECHTENEIK--SRDPIRCRECGYRIMYKKRTKR-CILLTMLSKYELGGNE 67
htar|LSRZ01000001     --------------MYFRERHLNVPIDHVVYTCAECGVDTVIV--PNTTLRCRSCGSKIFFKKRCRR-AAMQ-----YEAR--- 59
pber|PBANKA_1355800   --------------MYIREHDEDISTDPVVYICGECGIDTVIP--PNASLRCKNCGSKIFFKKRSRR--VMQ-----YEAR--- 58
pfal|PF3D7_1342700    --------------MYIREQDEDISTEPVVYICGECGIDTVIP--PNASLRCKNCGSKIFFKKRSRR--VMQ-----YEAR--- 58
scer|YHR143W-A        --MSREGFQIPTNLDAAAAGTSQARTATLKYICAECSSKLSLS--RTDAVRCKDCGHRILLKARTKR--LVQ-----FEAR--- 70
gnip|KK211494         -MIRSAP----ATSASKSVDVAVLYSCAIGYICADCGATTTLQ--TTAIVRCRQCGSRILNKKRSRR--LTQ-----YSAR--- 67
cvel|HBKZ01045703     ------------------MTDVEPAQVAVVYLCGQCGNDVRLK--PQDAIRCRRCGCRILYKKRTKK--PMQ-----YEAR--- 54
vbra|HBGB01049052     --------------------MNTDTTPAVVYICGECGRDVELK--PTDAIRCRNCGCRILYKKRTRK--MMQ-----YEAR--- 52
chot|ChTU502y2012_40 --MNGAESKTTSILDNNGVNINKGMVEPVTYICGECGADVSLLS-HGAAVRCRTCGCRILYKKRTRK--MIQ-----YEAR--- 71
tequ|BEWA_017140      ----------------MSSSFNNDSVEPITYICGECGNDIALQ--PTAAVRCRNCGSRILYKKRSYR--VVQ-----YEAR--- 56
bmic|BmR1_04g08572    --------------MDVNVVDTDENLEPVTYICSECGKDVVLQ--PVAAVRCRNCGSRILYKNRSHK--VVQ-----YEAR--- 58
sneu|SN3_01500325     --MDGGGG---SQDAGGLDDVGGAQMEPVTYICGECGCDVLLQ--PNAAVRCRSCGSRILYKKRSRK--MMQ-----YEAR--- 67
etht|ETH2_0301400     ----------------MDIDDTNTQLEPVTYICGECGCDVSLQ--PTAAVRCRSCGSRILYKKRSRK--MMQ-----YEAR--- 56
tgon|TGME49_254140    MNVGGGGM---GRPQGSGLDDGNSQVEPVTYICGECGCDVMLQ--PNAAVRCRSCGSRILYKKRSRK--MMQ-----YEAR--- 69
```

Figure S3.10. RPABC5

```
gnip|GNI_054480        MIIPIRCFSCNKPIANKWEPYVALLNE-GV---SEGEALQRVGCERYCCKRMLLGHVDIIERLLMYNRTASIQHTPHTANRLAEPAPLLSNHSTSHLAEPAHLLPL 102
tetr|I7ME31            MIIPVRCFTCGKVVGHMWNDFLQMISESNPNKKDMKSALDELRLNRYCCRRMILTHCDLIDKLLNYNIYKGQS--------------------------------- 73
htar|Htart_000340200   MIIPIRCFTCGKLIGNLWNSYEKKVQE-GI---SKADALNELNLHRYCCRRMILTHADMMDKLLCYNIYERKAH-------------------------------- 70
pber|PBANKA_0805400    MIIPVRCFTCGKLIGNLWSLYEKKLDE-GI---SKCDALDELNLSRYCCRRMMLTHADMMDKLLCYNIYERKL--------------------------------- 69
pfal|PF3D7_0708100     MIIPVRCFTCGKLIGNLWSVYEKKLEE-GL---SKCDALNELNLYRYCCRRMILTHADMMDKLLCYNIYERKL--------------------------------- 69
chot|ChTU502y2012_40   MIIPVRCFTCGKVIGNLWQPWLVLLQN-NV---PEGEALDKLGLRRYCCRRMILTHADLIEKLMAYNIHERKTMMN------------------------------ 72
vbra|Vbra_13888        MIIPVRCFTCGKVIGHLWEKYVHLIAA-DK---TEGEALNELNLNRYCCRRMLLTHADLISKLLNYNMYEKRTDY------------------------------- 71
cvel|Cvel_9127         MIVPVRCFTCGKVIGHLWDEWLQLLEN-EV---PEGQALGRLGLKRYCCRRMILTHADLIDKLLAYNVYDKREGSQDEGV--E---EIIN---------------- 81
etht|ETH2_0740700      MIIPVRCFTCGRVIGHLWEQYVEKLQN-DV---DEGVALDDLGLKRYCCRRMILTHADLIDKLLAYNIYEKKLPDQ------------------------------ 72
tequ|BEWA_019450       MIIPVRCFTCGKVIGHLWEKWLEMLKE-NV---PEGQALDDLGLTRYCCRRMILTHVDLIEKLLVYNIYDKRNPEIL----------------------------- 73
bmic|BmR1_04g08755     MIVPIRCFTCGKVIGNLWNKWLDKLSR-DI---PEGQALDELGLSRYCCRRMILTHVDLIDKLLAYNIYEKRTINS------------------------------ 72
tgon|TGME49_217560     MIIPIRCFTCGKVIAHLWEKWIDMIDE-DR---PEGEALDILGLDRYCCRRMLLTHADLIDKLLAYNIYEKRIVES------------------------------ 72
srcn|SRCN_5808         MIIPIRCFTCGKVIAHLWEKWIDMLDE-DK---PEGEALDALGLERYCCRRMLLTHADLIDKLLAYNIYEKRIVEN------------------------------ 72
hsap|ENSG00000177700   MIIPVRCFTCGKIVGNKWEAYLGLLQA-EY---TEGDALDALGLKRYCCRRMLLAHVDLIEKLLNYAPLEK----------------------------------- 67
scer|YOR210W           MIVPVRCFSCGKVVGDKWESYLNLLQEDEL---DEGTALSRLGLKRYCCRRMILTHVDLIEKFLRYNPLEKRD--------------------------------- 70
```

Figure S3.11. RPA49

```
hsap|ENSG00000137054 MYQASAVSLLPRDIPSCHSPSPGFSHLPTSSSQLAPD-LLQFPLGQDPSFLAIPILTLPPSDSLVPPYIVWYIVWPSALI 79
scer|YNL248C         ------------------------MSVKRSVSEIEIESVQDQPSVAVGSFFKGFRAPS--DT------------------ 36
tetr|I7MCY4          ------------------------MSLKVEV-------IK-KKADQRGPFLANFEQNIPPQEDFEQSIKV---------- 38

hsap|ENSG00000137054 SFLGCTLTVQFSN--GKLQSPGNMRFTLYENKDSTNPRKRNQRILAAETDRLSYVGNNFGT-GALKCNTLCRHFVGILNK 156
scer|YNL248C         ------------------------TFDLYKKKKSEKDE---FVLHGEN-ERLEYEGY---TDSSSQA--SNQYVVGLFNP 83
tetr|I7MCY4          -------RVQEKNKKKTVLRMSNDILEIFGKGQNCSGQ---YNSNSNETIINENEGASESNNNANKC--SSAYLIGIKDD 106

hsap|ENSG00000137054 TSGQMEVYDAELFNMQPLFSDVSVESELALES----------QTKTYREKMDSCIEAFGTTKQKRALNTRRMNRVGNESL 226
scer|YNL248C         EKKSIQLYKAPV-----LVSKVVSKSSKNLRG-PKIK---SKSDTRPSALRNALGEAFGTKKAKKAIADLERNRIDSDKL 154
tetr|I7MCY4          KTNTIKLFQVEQ-----LFKLQQH-TLKELENSQNIDSNIAKTKTTHMEQKQMLVQEFGTVKSRKKVKQMQNNIVHEQNL 180

hsap|ENSG00000137054 NRAVAKAAETIIDTKGV------TALVSDAIHNDLQDDSLYLPPCYDDAAKPEDVYKFEDLLSPAEYEALQSPSEAFRNV 300
scer|YNL248C         TDSA-I---DIVD----SVRTASKDLPTRAQLDEITSNDRPTPLANIDATDVEQIYPIESIIPKKELQFIRVSS------ 220
tetr|I7MCY4          SQNLKV---SIQD-KGEELEADAMNHTIRERDQEIDSKREFLPEFDLETKNPTEIFSLNSIITPEDLTTLSFAAEYIKK- 255

hsap|ENSG00000137054 TSEEILKMI-------------EENSHCTFVIEALKSLPSDVESRDRQARCIWFLDTLIKFRAHRVVKRKS----ALGPG 363
scer|YNL248C         ----ILKEADKE----KKLELFPYQNNSKYVAKKLDSLTQ--PSQMTKLQLLYYLSLLLGVYENRRVNNKT-KLLERLNS 289
tetr|I7MCY4          --RDLIKSECKNMTHLVRNLLFDFQT--HFGGRNNHS--------LI-AKQLVYLDYLLRVANKVKLSGKTLDEVSKETN 322

hsap|ENSG00000137054 VPHIINTKLLKHFTCLT---YNN-GRLRNLISDSMKAKITAYVIILALHIHDFQIDLTVLQRDLKLSEKRMMEIAKAMRL 439
scer|YNL248C         PPEILVDGILSRFTVIKPGQFGRSKDRSYFIDPQNEDKILCYILAIIMHLDNFIVEITPLAHELNLKPSKVVSLFRVLGA 369
tetr|I7MCY4          IPANLLKNILKNFYQA----FQSGSGVIYQRSQALKDKLISYIIVLALMVFDYKFDAAPLASLLKIEPKRFLTYAKEAGC 398

hsap|ENSG00000137054 KISKRRVSVAA--G----SEEDHKLGTLSLPLPPAQTSDRLAKRRKIT 481
scer|YNL248C         IVKGATVAQAEAFGIPKSTAASYKIATMKVPFKLPEMTRRGRGPRR-- 415
tetr|I7MCY4          SVKIKENKDSSS------KNAHQIEIELKAPLKLIEPKKPTKKNK--- 437
```

Figure S3.12. RPA34

```
hsap|ENSG00000117877 --------MEE-PQAGGEDAARFSCPPNFTAKPPASESPRFSLEALTGPDTELWLIQAPADFAPECFNGRHVPLSGSQIV 71
scer|YJL148W         MSKLSKDYVSDSDSDDEVISNEFSIPDGFKKCKHLKNFPLNGDNKKKAKQQQVWLIKFPSNVDISKLKSLPVDFESSTTM 80
tetr|Q23DW4          MSL-------------------LEQLAGYEDV----EFSQISISEEDLNNKKIYLFQVPADFNIQKLDGIQLDLKSEEDQ 57

hsap|ENSG00000117877 KGKLAGKRHRYRVLSSCPQA-----------GEATLLAPSTEAGGGLTCASAPQGTLRILEGPQQSLSGSPLQPIPASPP 140
scer|YJL148W         T----IDKHDYKI--MDDTDIESSLTQDNL-SNMTLLVPSESKESLKIASTAKDNAPLQFDKV---FSVSETAKIPAIDY 150
tetr|Q23DW4          E----LAQKESESIVISKHDKE---TQKYMKKQILPLFPVKKSKELLI--------NKKFAGF---FKIYKKFTFPNISK 119

hsap|ENSG00000117877 PQ--IPPGLRPRFCAFGGNPPVTGPRSALAPNLLTSGKKKKEMQVTEAPVTQEAVNGHGALEVDMALGSPEMDVRKKKKK 218
scer|YJL148W         SKVRVPRKD---------VPKVEG----LKLEHFATGYDAEDFHVAE---------------EVKENKKEP-------KK 195
tetr|Q23DW4          KAIR----------------KTKP----ITMKKENQGDESD--------------------------------------- 140

hsap|ENSG00000117877 KNQQLKEPEAAGPVGTEPTVETLEPLGVLFPSTTKKRKKPKGKETFEPEDKTVKQEQINTEPLEDTVLSPTKKRKRQKGT 298
scer|YJL148W         RSHHDDEEE----------------------SSEKKKKKKEKREKREKKDKKDKK-----------------KKHRD--- 233
tetr|Q23DW4          -------------------------------------------------------------------------------- 140

hsap|ENSG00000117877 EGMEPEEGVTVESQPQVKVEPLEEAIPLPPTKKRKKEKGQMAMMEPGTEAMEPVEPEMKPLESPGGTMAPQQPEGAKPQA 378
scer|YJL148W         -------------------------------------------------------------------------------- 233
tetr|Q23DW4          -------------------------------------------------------------------------------- 140

hsap|ENSG00000117877 QAALAAPKKKTKKEKQQDATVEPETEVVGPELPDDLEPQAAPTSTKKKKKKKERGHTVTEPIQPLEPELPGEGQPEARAT 458
scer|YJL148W         -------------------------------------------------------------------------------- 233
tetr|Q23DW4          -------------------------------------------------------------------------------- 140

hsap|ENSG00000117877 PGSTKKRKKQSQESRMPETVPQEEMPGPPLNSESGEEAPTGRDKKRKQQQQQPV 512
scer|YJL148W         ------------------------------------------------------ 233
tetr|Q23DW4          ------------------------------------------------------ 140
```

Figure S3.13. RPA43 (trimmed)

```
hsap|ENSG00000105849 -------------------------------MAAGCSEA--------------------------------------PRP 11
pber|PBANKA_0305800  MAQ-NPWHITKLKELRTSKLEKVINKFQEE-------------------------------------------------- 29
pfal|PF3D7_0208700   MAQ-NPWFVKKSKTLRTSQLEKFINKFNEE-------------------------------------------------- 29
htar|LSRZ01002144    MAQ-NPWTIRKCDGVKFRKVEKWFKLFNEE-------------------------------------------------- 29
gnip|GNI_115650      MAQ-RPWEVLRETELTLQEARQFLIKSEGG-------------------------------------------------- 29
etht|ETH2_1062300    MAQ-IPWQILSRSPIGAPDALRQLRVCFRSIRTAAAAAEGSAAS------------------------------------ 43
bmic|BMR1_03g00145   MAN-VHWEVESSKSVGFTRARDFA--------IKSCNSF----------------------------------------- 30
chot|ChTU502y2012_41 MYQ-IPWVVKESCVVTNEVAYHLIGKCIRSIGKLGKSKFEENPSS----------------------------------- 44
srcn|SRCN_1403       MAQ-VPWQPVWQRPVTAEEAAALLQRCISRMEKSACSSFVFPSSFHRIACGTSSSNSNSTGPRGRSSSGHNAFRGAGGAG 79
tequ|BEWA_025350     MGQ-FSWTMLKSKPASLSKALKSGEKCIKEITRSLSSSA-----------------H----------------------- 39
tgon|TGME49_246630   MAQ-VPWEKLTQKPVSEATAGEILSRCLCQLRRCSLYSA-----------------SD----------------G----- 41
cvel|Cvel_28284      MDQEEPWEVISRGPADTPSAYADYLGK---ITSFLGSRCPE--------------------------------------- 38
vbra|Vbra_15090      MDDERPWEVVTCRPIKRSKVVPFILRSRRELAPHLDARLARTD------------------------------------- 43
scer|YOR340C         MSQ------VK-RANENRETARFIKKHKKQVT------------------------------------------------ 25
tetr|Q22DC8          -------------------------------------------------------------------------------- 0

hsap|ENSG00000105849 AAASDGSLVGQAGV------------------------------------------------------------------ 25
pber|PBANKA_0305800  ----NSHLMNIP-KFKHI---K---------------------------------------------------------- 43
pfal|PF3D7_0208700   ----YEHLMHMT-RFKYI---K---------------------------------------------------------- 43
htar|LSRZ01002144    ----NGHLMHIS-TFEQI---H---------------------------------------------------------- 43
gnip|GNI_115650      -------------------------------------------------------------------------------- 29
etht|ETH2_1062300    -----QSLL-QHDALGRLKTAHAALSRMLQA------------------------------------------------- 68
bmic|BMR1_03g00145   -------------------------------------------------------------------------------- 30
chot|ChTU502y2012_41 ----------MQETVREMEKL-------S--------------------------------------------------- 56
srcn|SRCN_1403       YAAEDGRLLQQSEVYRQLQVAAAALELQPRAQLLHLSQREEDPQQQDGGSGGLMSPKRRLRSAAASDEAEAGTEAVSAAA 159
tequ|BEWA_025350     -------------KVRVLSQLESFIKSLQ---EIE-------A------------------------------------- 59
tgon|TGME49_246630   -TGIDGAWIEQSEVVRQLRSVRAAVKRSQ---RLFSSRKT--P------------------------------------- 78
cvel|Cvel_28284      -------------------------------------------------------------------------------- 38
vbra|Vbra_15090      -------------------------------------------------------------------------------- 43
scer|YOR340C         -------------------------------------------------------------------------------- 25
tetr|Q22DC8          -------------------------------------------------------------------------------- 0

hsap|ENSG00000105849 ------------------------------------LPCLELPTYAAACALVNSRYSCLVAGPHQRHIALSPRYLNRKR- 68
pber|PBANKA_0305800  ---------------------------------------NALSTIQE-DSELIINKKSFNIAHICCVAQLQPTYINNVRD 83
pfal|PF3D7_0208700   ---------------------------------------RTLESIKE-NSDLIINKKTFSILRISCVAQLQPKYLNKIDD 83
htar|LSRZ01002144    ---------------------------------------KTLQNVYR-YRHFVSEKETFRIASIRCVASIRPQNLSNIQD 83
gnip|GNI_115650      --------------------------EVGQKLERSIYKCVNDSKLND-V-LSSGGQRLFKRYHCRGLAQVMPLHLQYVDV 81
etht|ETH2_1062300    ---------------------E-----------------TADAEQAA-AEVAGEANNCFEQIRIRGTAILSPCFWQVPRL 109
bmic|BMR1_03g00145   -------------------A-----KHVDQNNFKRVFDSINSLVVAL-ESPIRGCKSNFHILTVNGYAQVPISFSEDI-- 83
chot|ChTU502y2012_41 --------------------------TEQLQ----------NIRESF-INKGEYDNECFELLRVKGTVQLLPNSIGNIED 99
srcn|SRCN_1403       AVEEHERGVGTAGRGGDTVNAEEDDEEQQQQQTRTSAPKKNSSAVAE-EERKGLRRSVLHVVKARQAQQQIVHILTAIPF 238
tequ|BEWA_025350     -----SQGL-----------------------------------------GDLENLPCLSFVQASGVIQLHPSFLGNVSS 93
tgon|TGME49_246630   -----ASGLRDGGRD--------------------------RSDEAT-RHPDDLRRSVFHVIRAKGMIQLLPRHLGNVKL 126
cvel|Cvel_28284      -----------------------------------HDHLIALSKISSHFHVKSSKC--IQRMTVRNSLRLSSVYQPFLQT 81
vbra|Vbra_15090      -----------------------------------SSPVAQLHNMASEFHADGVDVDSFVVIEAKNTVQLEARFLNEPLT 88
scer|YOR340C         ------------------------------------NPI------DEK--NGTS--NCIVRVPIALYVSLAPMYLENPLQ 59
tetr|Q22DC8          --------------------------------------M------EVE--EGASSQAQIEKKKCEELIPLYPCDLR-NLK 33

hsap|ENSG00000105849 --TG------IREQLDAELLRYSESLLGVPIAYDNIKVVGELG------------------------------------- 103
pber|PBANKA_0305800  ---G------IAIYLSNFMLKINHDIEGFSVCFNSIKLKEKEPITL---------------------------------- 120
pfal|PF3D7_0208700   ---G------ISVYLSNFMLKANHDVEGFCLCFNKIKLKEKESRVM---------------------------------- 120
htar|LSRZ01002144    ---G------LRIYLSHFMLKANHDLEGYCLCFRRLKVIKKEPQIL---------------------------------- 120
gnip|GNI_115650      ---G------IKTFLRNFNMSFVPELGGVWIACRKARLVEPKGFTS---------------------------------- 118
etht|ETH2_1062300    ---------AVYRHVMGFLFKFVTELDGVWLAFTETRPATVSKSSN---------------------------------- 146
bmic|BMR1_03g00145   -NAG------IYNYLSTFLIKYIPDFNGIWISFRKVKKLDSLARL----------------------------------- 121
chot|ChTU502y2012_41 S---------IKKYLCCFLFHYIAEFQGVWVCFHKISTVN---------------------------------------- 130
srcn|SRCN_1403       ANTGAANLWKTFSRPHATLHLYLPEFHGVWLAFDKVKIIHPRTARAAGAARGAAAAQEEEKEETAEDAAPAGRTTKRGGG 318
tequ|BEWA_025350     ---G------IYSYLSNFLMHYDEDLKGVWVSCGNVKQLD---------------------------------------- 124
tgon|TGME49_246630   ---G------IQLYLLNFLLKYLPEFNGVWLAVDAIRVLQPKSRLG---------------------------------- 163
cvel|Cvel_28284      ---G-----V-HRQLVENITKFIPELGGIWLGHWADARLLNR-------------------------------------- 114
vbra|Vbra_15090      ---G-----I-QYFLSNLLHRYLPEFRGVWMAHHSARALESC-------------------------------------- 121
scer|YOR340C         ---G-----VMKQHLNPLVMKYNNKVGGVVLGYEGLKILDADPLSK---------------------------------- 97
tetr|Q22DC8          ---N-----SLKNFVQKKLFKWDQKHQGIVLQCKNIKKS----------------------------------------- 64

hsap|ENSG00000105849 -----------------------------------DIYDDQGHIHLNIEADFVIFCPEPGQKLMGIVNKVSSSHIGCLVH 148
pber|PBANKA_0305800  -------------------------------------NNDPTVMFLKISFKLLVIVLKENYKIKVKINNIEPSNMRMGIF 163
pfal|PF3D7_0208700   -------------------------------------NNDPSIMFVKISFKLLILVLKENYEIKAKINKIEPLKIHLDIF 163
htar|LSRZ01002144    -------------------------------------NGVSSCIFLAISFKLLYINLKKELILSINKNQIPKTKN----- 158
gnip|GNI_115650      ------------------------------------DAEKLGIVCFQLVVEVLVFCPQPGLNLTAQVTEIRHDGLSCSIF 162
etht|ETH2_1062300    ----------TSTSGIPTSKRSRSSSIGALGFLLPDIESNDGTIFVSVETTALVFRPKVGDLITCETQTVRPAVLRLLWL 216
bmic|BMR1_03g00145   -------------------------------------NHNAEILSFSISVRALVYIPQLGIKAYGQVSLVSMSRITVLCY 164
chot|ChTU502y2012_41 ----------------------------TYGYV--PDCEPNGSFLVNIEFKALVFKPKIGELLICRISHVSPSHVSGITY 180
srcn|SRCN_1403       SARSKQAEGTVVTAAAAAGSRPGAAAAGGLGYVVPDTCTGGGLLLFEVEVECLVFIPKRNAMLVGRVLTVRPTHLRLLWL 398
tequ|BEWA_025350     ----------------------------SLGYLTSG--DACGLVTFKVALKILVFIPKLDD-IIGKINRMRPTSISVLIY 173
tgon|TGME49_246630   ------------------------GVLSPLGYMIPDADTGGGVMLFEVELDCLVFKPKTNAMLIGRMQTVRPSHVRLLWL 219
cvel|Cvel_28284      ------------------------------GAMLRESVDEGGVSILEVSVEVLVLRPEKNDVLVCEVTNVSSDGITATAF 164
vbra|Vbra_15090      ------------------------------GYTTANCADNTGYTLVDVQLKCLVWQPVPEDLLLGRVHYVGSHYIAITVH 171
scer|YOR340C         ---------------------------EDTSEKLIKITPDTPFGFTWCHVNLYVWQPQVGDVLEGYIFIQSASHIGLLIH 150
tetr|Q22DC8          -------------------------------SEKASIHDESPVIFVPCSYRLKVLQIKQGDVFLGQVTQTFNTHISLQLF 113

hsap|ENSG00000105849 GCFNASIPKPEQLSAEQWQT------------------------------------------------------------ 168
pber|PBANKA_0305800  GLIEAVMVDENFKDFYY-EGKN-----------------------------NT--------------------------- 186
pfal|PF3D7_0208700   GIVEAIFSEDMFKDFHY-DSRN-----------------------------NR--------------------------- 186
htar|LSRZ01002144    -------------------------------------------------------------------------------- 158
gnip|GNI_115650      GCFNGHIALNRLLEGSKPIINE-----------------------------EDESLNIIE-------------------- 193
etht|ETH2_1062300    GHFTAIISREHLGDR-FRYLEE-----------------------------AK--------------------------- 239
bmic|BMR1_03g00145   GMFNTIVSDIKQK---CYINK----------------------------------------------------------- 182
chot|ChTU502y2012_41 GILNIVIPMKSFKGDEYEFQKA-----------------------------DSLAYETNT-------------------- 211
srcn|SRCN_1403       DVFSVVVGQQEIPPS-YFYSQT-----------------------------DR--------------------------- 421
tequ|BEWA_025350     GVFNATIKSEDVKDT-IQYDGT-----------------------------QY--------------------------- 196
tgon|TGME49_246630   GLFSAVIPRSRLSPS-YDIDKE-----------------------------QR--------------------------- 242
cvel|Cvel_28284      GIFPVFVPLALVH---------PAWIPTHKGDLLWSGKGPKMSTNS----PVKIQFVRSKV------------------- 212
vbra|Vbra_15090      GLFPAFVSRQRLPKYL-KYAQNRWWCVADEGQSLFTRWKNAVDRDIDLTKEDEEHFKRERVKGEIKRERDLVDDDPDM-- 248
scer|YOR340C         DAFNASIKKNNIPVDW-TFVHN------------------DVEEDADVINTDENNGNNNN-----EDNKDSNGGSNSLGK 206
tetr|Q22DC8          GLFNVLIYADDISDKI-LKLND------------------D--------------------------------------- 135

hsap|ENSG00000105849 -----------------MEINMGDELEFEVFRLDSDA--------AGVFCIRGKLNITSL---QFK--------R---SE 209
pber|PBANKA_0305800  ----------FVRNNM--TYSINDIISFTIRKITHADSGTNVKLLGFV-------------------------------- 222
pfal|PF3D7_0208700   ----------FRREGK--FFSLYDIVLFTIKKITYGDNGANVKVIGYF-------------------------------- 222
htar|LSRZ01002144    -------------------------------------------------------------------------------- 158
gnip|GNI_115650      --------IKNPVTGKAQTMKLGSKIKFVVYNIVCFAS-----ADQETYTVEGSMR------------PLKIRKR----- 243
etht|ETH2_1062300    --------VYVHRKDKTRRIAEKAFVRMHVIEVPK-------SQSGDRVAIRGSLLARGS---GPLVQSTEA-------E 294
bmic|BMR1_03g00145   ----------------------GDIVSFNIQKISPQNDFVTLFATKLKVCKSPSPQSDYN---QLWVRPWLH-------- 229
chot|ChTU502y2012_41 --------LVNKINKEK-NLKVGSIIFVRIKKYNFSDNG------DFISSISGQ-----------F--ESLID------- 256
srcn|SRCN_1403       --------VYVHRSGGP-SIRSGSPICFKLLQYLGNDGH------GDVLCLAGSLPRRAG---GIF--PSLQQLR---HS 478
tequ|BEWA_025350     --------K--TVTGER-IIDNDTIIPLKITNVTVLP-K------NRWVNLTAILNIKQ--------------------- 237
tgon|TGME49_246630   --------KLQHKKGGA-DIEEGDLVCFQLTSYTQGS-R------AELLSLEGSLESGYA---GLV--PDEKSGS---SE 298
cvel|Cvel_28284      -----------------------------------K-------YYANNFVFEGSLADEKKT--GVL--KSHTTS-KLLPL 245
vbra|Vbra_15090      ----DRYLVDAERSAAESSIGIDDYVQFRVVSLREG-------RDGGLMNLDGSLKDQNDT--GPV--DDEGVLRPLVPL 313
scer|YOR340C         FSFGNRSLGHWVDSNGEP---IDGKLRFTVRNVH---------TTGRVVSVDGTLISDADEEGNGY--NSSRSQAESLPI 272
tetr|Q22DC8          --------SEWVDAKENTVIEVSDDIQFEVKTVDLQ-------EFGDFMLI-GSLKN--TKKNGPY--SSIKDK---YKV 192
```

Figure S3.14. RRN3 (trimmed)

```
gnip|GNI_085590      ---P----KKTP-IPSGAGGPSRVAEVVFRTSKRDNNAEVLRLAEYVNELLVEKSRSVESHKKLESLVRQTVLDFNVLIA 80
tetr|I7M8B2          -------SC----YDDYLKGN-KVLMNKIVNEMSNRHKEKEHIKMWLRASTKKLIDLFKNKQDVNFLIEVILYQMPWFEA 127
hsap|ENSG00000085721 -------------------------------------------------------------------------------- 0
scer|YKL125W         -------------------------------------------------------------------------------- 0
chot|ChTU502y2012_37 ----------------------------------------------MIQVPEKT--------QKDSSIYGLIGEFDFFGA 26
cvel|Cvel_23963      ----------------------------------------------------------------------MIRQFDFLAA 10
etht|ETH2_0731400    AAAAEDRAASLGPPPNLTGGN----------------ASVLQLGSWLLQRSSKL-----PQQQQQQLLESFLLRFDFVAA 331
bmic|BmR1_04g07665   -------------SSIYCSTN----------------SEVINICTKLLHLKSDN------KTEYSRLIELAILQFKWNVA 50
sneu|SN3_03500250    EGACAEVSCTSPSPTLLLRNN----------------VDVLRLGKWLAAQPSPT------SHRFEALFERLLLHFDFVDA 436
tequ|BEWA_005240     ---------------------------------------MYRKLQSL--TSDLPI----CEDNLSCFADLLINQFVWKCV 35
tgon|TGME49_238880   QEKPAASSFFFPPPPSPFGGN----------------ASVLRLGAWLAERPGLD------DDRFTPLLESFLLHFDFLAA 518
vbra|Vbra_19783      VEK-----------VSPTRVN----------------VELLNEAAALLEKQGDKS----RENAGVAFIERFVKGFNWFMA 114

gnip|GNI_085590      SD-----------------------------------------------------------------------REFSQWI 89
tetr|I7M8B2          GNEYEIQLQEQEQKKI-------LQQQTHANQQQHGIGSSTSL--FSNASSSKRNIQQQHGQQLTIKKP-SPHFDLFS 197
hsap|ENSG00000085721 -----------------------MAAPLLHTRLPGDA----------AASSSAVK-KL-GASRTGISNMR--ALENDFFN 43
scer|YKL125W         ---------------M-------MAFENTSKRPPQDF----------VAPIDQKKRKVQFSDSTGLVTLQPEEIKDEVFS 48
chot|ChTU502y2012_37 SD--------------------------------------------------------------------------SDFG 32
cvel|Cvel_23963      ARDL---------------------------------------------------------------------PIFEDFI 21
etht|ETH2_0731400    AVADSSSSSSDGG--------------ILVEREVVDDDEGEPPAAA---AAGAAATAA-AAT------AAAVAGDAAAFT 387
bmic|BmR1_04g07665   -----------------------------------------------------------------------SSSEISSFS 59
sneu|SN3_03500250    AASAAAAASAAAG-AAASCGAGRGQQQLQLQQPVADEETQRAEQQNAVADAREHREKT-EGE------EDGLVDEGEEFC 508
tequ|BEWA_005240     -----------------------------------------------------------------------TPEEIEKCK 44
tgon|TGME49_238880   GKVSRRIQQDQQIQRDLRLGE-DEEGEVSFFRPVGRHTDEKSE------QTRTGKREE-DRE------RKGQTDQLEEFE 584
vbra|Vbra_19783      -----------------------------------------------------------------------SDGVARRFT 123

gnip|GNI_085590      RCLHGIASSRGDL--FG-VVYSSLVQQLKTFHDHDEYR----DYGYGK--------------DVVLSKDIRCKSI----- 143
tetr|I7M8B2          ELSKFIMTLLSFSNAFAPQIIKFLISNIVPMEVKE-ME------------------KNKKSEIELEVEGKVFEN------ 252
hsap|ENSG00000085721 SPP----------------RKTVRFGGTVTEVLLK-YK------------------KGETNDFELLKNQLLDPDI----K 84
scer|YKL125W         A---------------------AMYSRFVKSALDD-LD------------------KNDSTQIGIIANQVALPSKNPERI 88
chot|ChTU502y2012_37 ELVHTIVDQIGGENTNNEKLFDVLKKKLTILDNG--TL---------NLFA-PAKFSKPDIKDIVLPDESYFDCK----- 95
cvel|Cvel_23963      EIVANFACAGGG-DGLE-EVISILLKRIKSFGRDG-YE---------QL---------QKLNPPLQLEGKIYKSF----- 75
etht|ETH2_0731400    TLMLDLLALRAGE-GAA-AAAECLVNHLKEACRGE-RMGCSGDPAAGAAAAANSSSSSSSSSAWLRLGRFAFHNR----- 459
bmic|BmR1_04g07665   ALLIDIITNNTI--SLH-YVVKFLVKSAKPKIGE--------------------------F-SDIFILNTRFENM----- 104
sneu|SN3_03500250    TTLLDILSLRSSSQTVG-TALQHLVSQLVKKYR-H-PV-CG-AAGGGGV-------AERGM-LVLGEGRFTFLNK----- 570
tequ|BEWA_005240     NIVLDLVTIDG-N-AIN-YLLKRFISQFRTDNREY-L-------------------------KVD-ICSRTFESI----- 89
tgon|TGME49_238880   TTLLDILSLRGGN-GVD-TSVDILVGQLREKPHRH-KA-AG-EEGGGEP-------AGGTTGGIRIGEKFFFADK----- 647
vbra|Vbra_19783      SLLLDMLATNGD--ATS-MVLAQLVKKLRMRPSDE-EL-------------------DAKTP-TIVVGRFSFESR----- 174

gnip|GNI_085590      -DDVRQRCIKIIQGQ-----PLNKLIDRQSFD-FRFLVTVLAFHPRAADKL----RGVTDLKIIQNKHDAKCRVICLVYD 212
tetr|I7M8B2          QKKLLMYIRDIMNKYILNSINDVKELNKEDTNFLLG---VLKYHPNSKEKMQ-RYVKLII---G-QAQFDDKISKCLYIQ 324
hsap|ENSG00000085721 DDQIINWLLEFRSSI-MYL--------TKDFEQLISIILRLPWLNRSQTVVE-EYLAFLGNLVSAQTVFL---RPCLSMI 151
scer|YKL125W         NDKNLNILLDILSSN-INRIES-----SRGTFLIQSIINFEKWWELPPHTLS-KYIYFIKILCSSIPKWW---QDVSMIL 158
chot|ChTU502y2012_37 -SSLKKELKSICQNA----QIDSQIFHSKDLSILRF---VFSHHNFFNSQNETRYPQDYDFYVIKHPNTDFNGCRTFAIK 167
cvel|Cvel_23963      -DEIHNTVKAIVGDT-----PTNGILGRSDAALLNF---LVQHHPQATTKT----RDLLGFTVAPHPKGSLVHPRCFHAL 142
etht|ETH2_0731400    -RAVKQKLRAILQLG---GKEEGQDLQGEDFELVSA---VLRFHPRARIKT----RGMKGIYIGAHPNPRFHSTRTFFVR 528
bmic|BmR1_04g07665   -QQYSSYISNLLNKY-----PNKTSITGNEFHIVSE---LAKNHPSLRYTF----NKIIGIYSNTTNT----SLKSFIVA 167
sneu|SN3_03500250    -AALKKHLRQILQTY-----DENEALDGEDLELVKL---VFSFHPNFQRKLP-KEEAFGGIFVATHPNPQFHSTRCFFLR 640
tequ|BEWA_005240     -FLLKKHIRDIILTES----QDDINLQGENYDLLLE---LLKYHPRGIELH----KNVVSIFPSTHRNPKFNGLRCFFYR 156
tgon|TGME49_238880   -VALKSRLRQILQAC-----DDEEELRGDDFELVKG---VLEFHPKAKRKM----QNLSGLFVSTHPNPKFRATRCFFIR 714
vbra|Vbra_19783      -VALKQRIRTIMTDA-----ADGEDLKGEDFELLRE---VLRYHPRADYKQ----KDCVGIFVARHPNVKFDCR-AFFVR 240

gnip|GNI_085590      PKQ----------------------------------------------------------------------------- 215
tetr|I7M8B2          NKE----------------------------------------------------------------------------- 327
hsap|ENSG00000085721 A------------------------------------------------------------------------------- 152
scer|YKL125W         V------------------------------------------------------------------------------- 159
chot|ChTU502y2012_37 KKHGKSSKHDE--------------------------------------------------------------------- 178
cvel|Cvel_23963      QQTEKEREKREEEAK---------------------------------------RIREENPPA----------------- 166
etht|ETH2_0731400    REDPQLATASQPASQLATASQPASQPASQPAGQLAAASQPAGQLAGEEQQAAASQLATASQPA---------GQLATASQ 599
bmic|BmR1_04g07665   LD------------------------------------------------------------------------------ 169
sneu|SN3_03500250    RLILTSRSTAATAAATAAAA--------TTAGKRDAV-----ACAREESNR---DVSAPSPPASPSSSSLPFPPPPASSL 704
tequ|BEWA_005240     NKD----------------------------------------------------------------------------- 159
tgon|TGME49_238880   RTIHASPSSSSSSSSS----------------------------------------------------SSSASPPPGTVT 742
vbra|Vbra_19783      RSV----------------------------------------------------------------------------- 243

gnip|GNI_085590      ------------------------------------------------------REDGLTEDDISFSKCLPYIYDPNSDR 241
tetr|I7M8B2          ----------------------------------------------------------GQKIDISYVKSVFNLVDEQFQK 349
hsap|ENSG00000085721 ---------------------------------------------------------------SHFVPPRVIIKEG---D 166
scer|YKL125W         ---------------------------------------------------------------SCFI------------- 163
chot|ChTU502y2012_37 ------------------------------------------------------SDNEQQLIPISYVSSVENITTKWEKA 204
cvel|Cvel_23963      ------------------------EEGGKKEGDPEGDEDPYVL-----PPYEMDSHYPPWHEPFSYTRCLEGLRQRLCAE 217
etht|ETH2_0731400    PASQLATASEPDEQLATAA-QPASQPADQPAGQPASQPAGQLASQLASQLATASRAKLAKDEDFSYVRCVENILSADTKA 678
bmic|BmR1_04g07665   ---------------------------------------------------------DGTSIDLNYAQCLSNITLSSDIW 192
sneu|SN3_03500250    PAAALACA---------AAAAASS-------------P-PA-------AGVDWQEEVREEWEDFSYIRCVDNILSDECKA 754
tequ|BEWA_005240     ----------------------------------------------------------GFCEFFSYARCSDNVKTRGEIF 181
tgon|TGME49_238880   PEGVATADENAGERAAAALAGSDQKPGEKREEQEEREE-GQ-------TKAEEQTDVQEECEDFSYVRCVDNMLEDDSQA 814
vbra|Vbra_19783      ----------------------------------------------------------GMHEDFSYVRCVDGLPSADAYA 265

gnip|GNI_085590      IR--------------------------------------------DLLCDAIFRQFRLFFTSRGTYMID---VLQKSAP 274
tetr|I7M8B2          IGLKQGS----------------------------PN----QKKMMLQVVDLLAKIIKIY-PLYQNDLCR---ILQESYP 393
hsap|ENSG00000085721 VDVSDSD----------------------------DEDDNLPANFDTCHR-ALQIIARYV-PSTPWFLMP---ILVEKFP 213
scer|YKL125W         ----------------------------------------LPIKQTVCHHDMLKYFLRMI-PSSMGFIDT---YLAKFFP 199
chot|ChTU502y2012_37 GR---------------------------------------------RIINLMQAIVNIV-PSDIKLFIT---TMQDIYP 235
cvel|Cvel_23963      AAEKNPDTIKVRASKYGKGELYEVVPLPPDWLRIGAGSEGLAELARHKLHSLLKVLADKL-PLQVSKAVENGLQMAYNRP 296
etht|ETH2_0731400    AR---------------------------------------------AICFSLKQLADCF-PCCCCRIYE---ALKRAAP 709
bmic|BmR1_04g07665   RS---------------------------------------------KLCDIIVDIYRTF-PNSHDIILN---SLDENFP 223
sneu|SN3_03500250    DR---------------------------------------------LICATLQQVVEVF-PTCSRQLQE---LVRRYFP 785
tequ|BEWA_005240     RE---------------------------------------------HICNIIIEIYKLS-PVILSIITD---LLETYFP 212
tgon|TGME49_238880   DR---------------------------------------------LICHILKQLAEAF-PGCVAHLHE---ALRRNFP 845
vbra|Vbra_19783      YE---------------------------------------------RICDAIDRVCSFF-PQATQLLVK---VLGDNQP 296

gnip|GNI_085590      PPEFQQIGTSIVDARRTSTYILNYIQAVLGLMSRASLKPFSDNGNYVQLDTIDTYKLYRTLQTFIIRYLHNMETERMD-- 352
tetr|I7M8B2          H-RVQG-----------EQFHKIYLTNILQLAIKC-------------------PQLMPKILIIAVQKLIEIDSEIKLDH 442
hsap|ENSG00000085721 F-VRKS-----------ERTLECYVHNLLRISVYF-------------------PTLRHEILELIIEKLLKLDVNASRQG 262
scer|YKL125W         N-KNDT-----------RRKLVNYTSNLLKLRGYC-------------------SELGFQIWSLLIEKIISIDVELQNEL 248
chot|ChTU502y2012_37 IAFRNS-----------LDSYILYSKLLFHGIKLI-------------------PSTMSILLRFLINKLISLESEIHSKN 285
cvel|Cvel_23963      S-AFAP-----------LQDQESWVISLLDIASRS-------------------ARLFHPLLRIVLERIVELDLSIRHGD 345
etht|ETH2_0731400    A-AKQT-----------PDVHAAFAKCLLWLAHNI-------------------QSLRGPLWRLLLSRMAAIDSEIKQKD 758
bmic|BmR1_04g07665   H-HSFP-----------LESHVSYLKIFISLSHLL-------------------NDSRLKLYRIVISELIRMDSETQNLQ 272
sneu|SN3_03500250    P-LRGP-----------VETLAAYVQNVLSLALRI-------------------PQIRAVLWRLLFEKLIFLDVEVKLAD 834
tequ|BEWA_005240     H-HNIS-----------IEHHVSFTKAILYIARHI-------------------EPARPIFYRLIINKLTIIDAQIKLAN 261
tgon|TGME49_238880   P-LRFS-----------VDALVAYTKNLLWISQHV-------------------PQIRALLWKLLVSKMTSIDVEIKLAD 894
vbra|Vbra_19783      H-HRKN-----------VEEHQNYTRSMLWLCRRK-------------------PQMRAGILKLFIARLIDFDVEIKEDD 345

gnip|GNI_085590      ---DDIAEGFDEDQFNFWLKEQADELTRILQSKKHRY-------SAQQLEQAKQMVRN--RDLMYKKYNQVKTEEEVGPV 420
tetr|I7M8B2          DAQENEKNS--------------------------------------------------------------------QIM 454
hsap|ENSG00000085721 IED-----------------------AEETATQTCGGTDSTE--GLFNMDEDEETE---HETKAGPE----RLDQMVHPV 310
scer|YKL125W         DELDDDVDDDDLEEVDLEDDDDLDDDSGDDDDENCGNSNEELRSGAADGSQSDSEDMDIIEGMDGTEEYNVELTQGIKEL 328
chot|ChTU502y2012_37 PNN------YTKERFEKWRQEELQTVAI------------KIRRGEYADINSAKSYIQ-DLDGLKAQFSQRFTEEDIDRN 346
cvel|Cvel_23963      PQL------PSVKDVEKWREGKLMTLES------------DIRRGTLNAESAVSFLDS-DLGELLLRFLQSRTEEDIDKR 406
etht|ETH2_0731400    PNS------VDAEQLSAWKREQLAELSL------------QLQQGCVGLEAVQQLLQQ-QQQLLQELYCRVRSEEDIDAM 819
bmic|BmR1_04g07665   ISP------T------------------------------------------------------------------DKGV 280
sneu|SN3_03500250    PNS------IDLEQMQLWKKEQLTLLAL------------QLQQGGVSAAAVHSILGE--PQWFQMTYQKLRCEEDIDEM 894
tequ|BEWA_005240     PNS------VDVEKTESVISQKLKEYYN------------KLNKGDGNFQEINEKLRN--PNWFKDMYEKLRTEEDTDDM 321
tgon|TGME49_238880   PNS------IDLEQMQVWKREQLALLAR------------QLEKGAVAPSVVQSILGE--PQWFCLMYEKVRSEEDIDIM 954
vbra|Vbra_19783      PNS------IDIEEYELWKREQLNFLSV------------QVSVGAKDPAEAQKLLND--EERLRQLYRMRRGEEDIDKM 405

gnip|GNI_085590      KLKLDAAYCLLYRFFVKEFESKKTLILRRSTFDKHFSESAKVLQDVLGPTAYAAQAPQSPIVSPMLSPMMSPY---Q--A 495
tetr|I7M8B2          AEKLDWLMLIIFEFIDYQLKDKVSSDSCFGQKEE-----------------------------------------L---- 489
```

```
hsap|ENSG00000085721 AERLDILMSLVLSYMK---------DVCY--------------------------------------------------- 330
scer|YKL125W         STKLDSILTLVSTHVE---------E-QV--------------------------------------------------- 347
chot|ChTU502y2012_37 AQVIDNVMKGLFDFISEVYENGFQYT-----------------------------KIFLNSTETSYSTQTKNL------- 390
cvel|Cvel_23963      ALACDHLLHIVFQFLESLLSSTEMREGTEKLVPG---GGFDDLEEMEVPAAWKFAHNEIFDTGDKYSSSSSSGSSSD-SE 482
etht|ETH2_0731400    ARVLDSLMLQVFSFLKARLAAAARPRAAAAAAAA-AGSSSSA-AAAAAAAV----QRTQPSSNPDYSPCADDSPPLS--- 890
bmic|BmR1_04g07665   PLKLDVLMTLLLQDLDFILK------------------------------------------------------------ 300
sneu|SN3_03500250    ACRLDACMRVIFEFLEQSLPRLLASSSSRETRSRGEGGGGG----GGERRR----DSSRGSAERNASPSSSS---SKGRR 963
tequ|BEWA_005240     SQKLDVLMGIMFEELQVILKANIDKPASLTSRSMES-----------------------------SSNCDDSCSSESD-- 370
tgon|TGME49_238880   AAKLDGLMRVSFSFLQCFIPQLSGKPAPPGAYREEEDERES----VGSPSS-------RSSV-ADYSPCVDDEPKEEKKR 1022
vbra|Vbra_19783      AQGLDHLMSMGFDFLQAELLSLSHSPPVNPIKPD---------------------------------------------- 439

gnip|GNI_085590      -----------AGAGSIMDSGSAMGSG---------------S-----------------------------AIGSEPAS 520
tetr|I7M8B2          -------------------------------------------------------------------------------- 489
hsap|ENSG00000085721 -------------------------------------------------------------------------------- 330
scer|YKL125W         -------------------------------------------------------------------------------- 347
chot|ChTU502y2012_37 ---------------------------------------------------------------------------KVPNN 395
cvel|Cvel_23963      DGEEE------DSQGEEEDEGEGK-RG-------------KGG------------------------------------- 505
etht|ETH2_0731400    ----------------------------------------PAG------------------------------------- 893
bmic|BmR1_04g07665   -------------------------------------------------------------------------------- 300
sneu|SN3_03500250    NSSSDISLSSREAAAAEEEEGEAAARG--EETAAGRSAAAAAG-RGEAAHLQKRRSSSSSMRQGA-------REQRHPKE 1033
tequ|BEWA_005240     --FDDL-----------TDRNLSTPRSCNIKNEFVKSEVNTNS--------------SSSIRGAI--YKSSI--IKADSD 419
tgon|TGME49_238880   TGTSEK-----------DPRGDSERRSCSEERRDSPSPFSPQSILSPQPAVTSPQLASTSPRGGPSAFPASRHTTLAPRD 1091
vbra|Vbra_19783      ------------------PDTDG---------ATPPQPQQPQQQ--------QQQLQQQ--------------------- 463

gnip|GNI_085590      PQIDVRS----GGGLLM--------SEVSDIQ-----SITSSRKSR---AGPETLKVTNILLSHLLDDFEENCLGAYDSP 580
tetr|I7M8B2          -------------------------------KQNSQ-------------DKKDKSQMEDKFFKIFLNVFSEQILITHQSK 525
hsap|ENSG00000085721 -----------------------------------V-------------DGKVDNGKTKDLYRDLINIFDKLLLPTHASC 362
scer|YKL125W         -----------------------------------T-------------PESLESGEGVGVFNTLTTLFKTHVLPTYYTR 379
chot|ChTU502y2012_37 VSNILSSSDLE-------------SSAVEDIVVSGYGK-----K------EELLYKQFVDLESTILNIFETQILAIESCQ 451
cvel|Cvel_23963      ---------REGTGRKA-----D--------RENEEGGEDQGKKRRYDFRGIGQLISKREIVAEIFKFFEESVLATKGLR 563
etht|ETH2_0731400    -------------------------------------D--SPAAAA---ATAAAAAAGDPLIADLFEAFLRVVLPVPSCR 931
bmic|BmR1_04g07665   ----------------------------------------------------DPDFDSNAFVSEIFQILKHNIHTLSTSQ 328
sneu|SN3_03500250    PQQDLQQGAKEGDETQQQEEDEDREEKIEEAYE--AGKEEEGQERR---EDEEEESTADAFVQELLALFAEVVLPAHKSK 1108
tequ|BEWA_005240     VK-----------------------HVKNEIFENGADIRSCTSSSS---SSSVKYSVSDIIVIELIDTFEDIILPTYNCK 473
tgon|TGME49_238880   PRRLLSPALSPA------------PAWPPDAARGPPGLRDRSRETR---RDKKPLTEADVLVYELMDVFVEVVLPVHKCK 1156
vbra|Vbra_19783      -----------A------------SSI-------------SSKRGR---GLSVAHCEGDVLMDELLMIFEQIVLPTHRCR 504

gnip|GNI_085590      VLPLLFFGIVNLDFEVHGNMFLNRILTAFRNQTT-NELSKEKIATYMIGYACRCNRISVQ-----AIQDVAVHLANDLLF 654
tetr|I7M8B2          YVQFIIFYLCSLRNSFQNAFLNHLVKNITEKNPLIYKSTQINSIYYLGSYIARAKYIE-NETIRKIFEYLITHLNRYIQK 604
hsap|ENSG00000085721 HVQFFMFYLCSFKLGFAEAFLEHLWKKLQDPSNP--AIIRQAAGNYIGSFLARAKFIP-LITVKSCLDLLVNWLHIYLNN 439
scer|YKL125W         SIQYIMFHVSQQQLELMDSFLVTLIDISFAVNEA--AEKKIKSLQYLGSYIARAKKLS-RTQIIFVASYLTSWLNRYVIE 456
chot|ChTU502y2012_37 FVNYIPIYLVCHCDSWCEKFLQIIFKKLFNPHET--LIIRESSVDYIVFFVTNYQIVCNFKFYTPCIKYLMQFLHDFVAH 529
cvel|Cvel_23963      YVQFIYYYICSFHATYAEAFSLRLIKCMEREGVS--VETQRHAACYLSSFASRAGFLP-YDFVRLAIRRLLMAMERQLNK 640
etht|ETH2_0731400    YTQFAVFFAASFDPQAQQLLLQQLFLLLANRQRS--IRLRRAACCYIASYLCRARNLP-PSVAAAALQQLLLLLHRG--F 1006
bmic|BmR1_04g07665   SVQFIFIYISSLKPYYTEILLQNLFSILYDEAQL--LDCRIAATGYIASLICRQQLLC-DTFIIFTFEYLLKFLGKWSKR 405
sneu|SN3_03500250    YVQFIFFYLSSLRMRWAGRFLQVLFQILFDKRQH--VLLRRIACVYIASFVCRARHLP-QRFVCLSLQHLLQLLHGG--F 1183
tequ|BEWA_005240     YVQFLYFFIFSFNAQWFKTFLERLFTIVFDNNQH--VVKRRTAASYISSLICRSKYVN-PKFVCGTVNYFFALLSLFDHL 550
tgon|TGME49_238880   YVQFLVFYLANLRVEWAGRLMSLLFRMLFDRQQH--MLLRRISAVYISSFLCRALLVP-PRFVRLALHHFLQLLHRG--F 1231
vbra|Vbra_19783      HTQFLLFYATSLKPAWAERFLQLLFDRLYDSNVY--VATRRKCAFYIASYVCRAEFLP-LEFSRQSLVYLLGFLHQH--C 579

gnip|GNI_085590      LARKASVSGSPT---------------------------------SGGPTIAGLNAAVTCFSGLLEIFCARWNQLKDDLE 701
tetr|I7M8B2          FDERQKEENYNE----------------------------------GLHKIH--LNLYHLLQAMLYILCYRFKAVLESK- 647
hsap|ENSG00000085721 QDSGTK----AF----------------------------------CDVALH--GPFYSACQAVFYTFVFRHKQLLSGNL 479
scer|YKL125W         REEEVD----QR----------------------------------GGMERF--KHFYAAFQALCYIFCFRHNIFRDTD- 495
chot|ChTU502y2012_37 WSIEKSDQNLSSQEIFHSNK-----------RSGFKRSFGGNSQKHSHLTLR--NLFGLYC-HVVFSLCKFVSVIINTIL 595
cvel|Cvel_23963      WKKESENEDTSAGGAKSKRKGKGKGRAR-----TFVPIPGDTS----DPRL---LLFYTYAECAAYILIYRGEWLARDTA 708
etht|ETH2_0731400    LRTGASHLQQQQ-Q----------QRM--ALLQGSFSGEFDSP----DSPL---ALLRTVVECLCYGLCFWLDELLLQCK 1066
bmic|BmR1_04g07665   A--------------------------------------KDNK----HIPS---NLIKSVFEACIRIIYFHADPVLTNIR 440
sneu|SN3_03500250    LRTPRKLPASAA-SAAAQAKQQQHKRLSQLWET--QRHTLCNP----GSPL---ALFFTVCQCCCYILCYHFDALAWSSR 1253
tequ|BEWA_005240     LLKSYDSSSSVK-SD--SIAGSSVGDLS-SRRKNKYGDYGVKS----SSKL---SLFYSIFQNILYIYCYHADILAVSPS 619
tgon|TGME49_238880   LMTGTPNAPR-Q-QQ------RLLG----LLRAQHQTV--YNP----ESPL---ALFYTVCQSCCYILCYHLDALAKSAP 1290
vbra|Vbra_19783      NKLAITNTPHNT-AE------RAAG----LLRAGSASAYPINP----EGNR---TLFYAVCQAVCYIVCYHGAAFMADTR 641

gnip|GNI_085590      TRV--------------------------LL----------------EIFLIMAYANSVFENRFV--------------- 724
tetr|I7M8B2          ---------------------------ENLLQKFYEIMDK-----ACSNFNALFFISPHIQNVFS--------------- 680
hsap|ENSG00000085721 KEG-----------------------LQYLQSLNFERI-------VMSQLNPLKICLPSVVNFFA--------------- 514
scer|YKL125W         -GN-----------------------WECELDKFFQRM-------VISKFNPLKFCNENVMLMFA--------------- 529
chot|ChTU502y2012_37 SENVQ-----------DSS----FEDLCFLIDSLLNMNRGFIPFILCGDLSPIDNIDPSTLTIFLKSVIILIHKVISIQK 660
cvel|Cvel_23963      RKG-----------------------LDEFKDTSYGLNQLFSPT--LNKYLPMRYMKMTIRKHLA--------------- 748
etht|ETH2_0731400    --------------------------LGFLLLPPFDLAAVL------LLLQPLQQLKRGIVRQFV--------------- 1099
bmic|BmR1_04g07665   KYEIL---------------------RLFSSIIYGSLGTLLN-----CNIDLLEKAKLSIMRINGPNKML---------- 484
sneu|SN3_03500250    NSSSSTPPSSSSISSSGSGGKRNSWPGAFLLERETSLGAVLT-----CPLQPLMHIKRSIAREFI--------------- 1313
tequ|BEWA_005240     --C-----------------------LSFVQNSEKGLIAYLD-----SSLSPLMYCKESIITEAI--------------- 654
tgon|TGME49_238880   --S-----------------------LAFLKERNTSVAALLT-----SPLRPLMHIKRGIAREFV--------------- 1325
vbra|Vbra_19783      NGG-----------------------LDSILHGEQSLIAILQ-----SPYRPLANIRKGIATELR--------------- 678

gnip|GNI_085590      ---QMTRKIVCSTFVTKM-------KRFDVSYHKELSAFLSG-LSDH-----------------------GET------- 763
tetr|I7M8B2          ---DVCVSYGEDQL-------------V-----------E--LFEK---------------------------------- 697
hsap|ENSG00000085721 ---AITNKYQLVFC-------------Y-----------T--IIERN--NR---------------QMLPVIRST----- 543
scer|YKL125W         ---RIAQQESVAYC-------------F-----------S--IIENN--NN---------------ERLRGIIGK----- 558
chot|ChTU502y2012_37 EKLSISKYYGAIKLISELLNSNSSKEIV----KVESKLFLDKLWLWH----------------------SGKYVNKF--- 711
cvel|Cvel_23963      ---RLLKKVGGFET-------------LVPSFEKEDLQDS-QRLTKKARDDLAVRQTALRVVSEEAEKDRGSSAVSSASA 811
etht|ETH2_0731400    ---AACRRVGLFGS-------------L-------EAYFA-AVLLHH-RKQQ---Q-----QQRQQQQLRGTAAKAAL-- 1144
bmic|BmR1_04g07665   ---RLLKYLGDRTEK------GSNLQLLHLPFDSCNLVYT-KYYLML-NSDPLAN------T------LSGKY------- 535
sneu|SN3_03500250    ---AACRRVGCFPD-------------L-------EPLFQ-DVLRKR-DSNSSSRP-----QQQQQQREAGGATD----- 1358
tequ|BEWA_005240     ---NATKNFPNLTK-------------L-------HDCLM-NFKKLA--------------------------------- 677
tgon|TGME49_238880   ---AACRRVGSLDS-------------S-------SDFEN-ALKEVQ-LRDP---------------------------- 1352
vbra|Vbra_19783      ---NLFHRLPGYDY-------------L-------RPL-----FE----------------------------------- 695

gnip|GNI_085590      ----VIAPEA----------G------------------------------------KKK--KG-----------ETM-- 778
tetr|I7M8B2          --------------TS-------------------------------------------------------QTS-- 702
hsap|ENSG00000085721 ------AGGDSVQ------------------------------------------------------------------- 550
scer|YKL125W         ------ADSDKKENSA-------------------------------------------------------QAN-- 571
chot|ChTU502y2012_37 ----KTSNSN----L--------IEDQDISFHILELLKVIED-----EIEIH----SEEEP-EPPNQEIDI----PEKKY 761
cvel|Cvel_23963      QSEGDGTPSSRLGNLSE--EG----GEEGKDGMLESLGEEEEKEEEAGADGEEAEEGEGEGGAGEEEPEGEQKKKEAMKF 885
etht|ETH2_0731400    ----SAAPAAAAAAPAAAATAAASEREEAADDVLSPLSPTEQ--QQQQQQLQQQQQQQDEE--PPYSPM------SPNPF 1210
bmic|BmR1_04g07665   -----ISDFHIYQDLVV---G-NIEYDWESRSSDVPKKRFSK----YGSSISKDVD-----GLGDFQLL------RA--- 588
sneu|SN3_03500250    ----KTAPSRALSSSGE---G-SSSEDSSSSESS----SSER----NGVAIGTAVEEEEEEGRGKRESE------EGNLF 1416
tequ|BEWA_005240     -------------------------------------------------------------------------------- 677
tgon|TGME49_238880   ------PPSSCYRSSA----G-ENEEE--------------E----EGREEREAERGEDEKERGKRIS----------QV 1393
vbra|Vbra_19783      ------------QDAA----G-G----------------------------------GGDH-GG---------------- 707

gnip|GNI_085590      ----------------SVFEMETTW-PVRPFARVDLFKFGSYVHRPEIWF-----DCWAKDDVESAASQPLPLGAEIAQD 836
tetr|I7M8B2          --------------QATESISAALQQRAQLL--DNYFPFDPYCLQYSDKFICEIYQNWQDTNSLSSQSF-----VEQ-QL 760
hsap|ENSG00000085721 -------------------------ICTNPL--DTFFPFDPCVLKRSKKFIDPIYQVWEDMSAEELQEF-----KKP-MK 597
scer|YKL125W         --------------TTSSSWSLATRQQFIDL--QSYFPYDPLFLKNYKILMKEYYIEWSEASGEYESDG-----SDD--- 627
chot|ChTU502y2012_37 GEM-----------ESSILPEQSLW---EYV--WGDVPVSYEELEQELYVTKNKMYKEDLDRQFDYSRE-----ELNGQD 820
cvel|Cvel_23963      GTALVTFAGQLAEAEKDRVGLDGKGDPWDPL--KNQFPLAAELLMVSSSYIEPTYRDWHLVLRGPGKDE-----MIF--- 955
etht|ETH2_0731400    SPSCVSAAV----PSSSSSAAAAAAATADPL--DAFFPFDPYRLRHSQRFITNKYREWKHVEAAAMEQQ-----QQQ--- 1276
bmic|BmR1_04g07665   ---------------------GLTTSIFKPQ--S-KSQLDYLLLSDAYKYSKTLYN------------------------ 620
sneu|SN3_03500250    SSS-----------NSAERVNDVFW-LFDPL--DAFFPFDPFLLRHARRFIIHKYREWKDVVAYVMTLQ-----KKV--- 1474
tequ|BEWA_005240     --I-----------SSLEY-EDINWLYYSPI--NAFFPFDPYLLHHSSYYIRDKYRHKLVNESSDLPRD-----SVK--- 733
tgon|TGME49_238880   DAV-----------SSNDLLSDVLW-LFDPL--DAFFPFDRYLLRHSGRFIANKYRDWSAVVAFVMQRE-----EEK--- 1451
vbra|Vbra_19783      ----------------GELEAMTDD-SFDPV--DAMFPFGPYRLRHSVRFVQDKYRDYAAPALADIKAE-----GTT--- 760
```

Figure S3.15. TAF12 (trimmed)

```
bmic|BMR1_02g03651   -------------------------------------------------------------------------------- 0
hsap|ENSG00000120656 ----------------------------------MNQFGPSALINLSNFSSIKPEPASTP------PQGSMANSTAVVKI 40
scer|YDR145W         NVNTSQTEQSKAKVTNVNATASMLNNISSSKSAIFKQTEPAIPI-SENISTKTPAPVAYRSNRPTITGGSAMNA-SALNT 398
tgon|TGME49_244160   ------------------------------------------------------------------------MSESACIVDC 10
chot|Chro.70325      -------------------------------------------------------------------------------- 0

bmic|BMR1_02g03651   ---------------------------MGELSNLITTQNVHN-LLNEISNGYQVEDPVIQSLSIVMEDYLQDVLLNAYQM 52
hsap|ENSG00000120656 PGTPG----AGGRL-----SPENNQVLTKKK----LQDLVREV-----DPNEQLDEDVEEMLLQIADDFIESVVTAACQL 102
scer|YDR145W         PATTK-----LPPY-----EMDTQRVMSKRK----LRELVKTVGIDEGDGETVIDGDVEELLLDLADDFVTNVTAFSCRL 464
tgon|TGME49_244160   PEVGPLVPAHTPSFAPASLSANRQEVSVEEVFSEIHAELVAE-ALKDLDPDAELDSSAATLVGDLLDAFVVDTGIQAARI 89
chot|Chro.70325      ---------MQDEFVDH--------KLETELFSILSPELLNE-IMIEEFPHIRLGPSVSQVLTTLTNGFIQDVVKNSYII 62

bmic|BMR1_02g03651   CRHRYSKVITPQDVWYYLNITDSSPIEPLAGLGATMGL--GAVISL-----IAGSDFKD--------------------- 104
hsap|ENSG00000120656 ARHRKSSTLEVKDVQLHLERQWNMWIPGFG-----------------------SEEIRPYKKACTTEAHK---------- 149
scer|YDR145W         AKHRKSDNLEARDIQLHLERNWNIRIPGYS-----------------------ADEIRSTRKWNPSQNYN---------- 511
tgon|TGME49_244160   CRFRRGKTLTREDLDFCLSRNFAVPSTSDLPVSSVVSSTVSSVVSSSVSSSVSSSVSRDTGGWRPLAVASPLSSFAWGRQ 169
chot|Chro.70325      SKHKNHSEVQADDILVYLRMK----------------------------------------------------------- 83

bmic|BMR1_02g03651   ---KADKTKS---------------MVNYLQLPRSNYSLSHVLNRGSGGRNNRKIASAAAEQRRILSSYFSDPETNKTDE 166
hsap|ENSG00000120656 -----------------------QRMALIRKTTKK--------------------------------------------- 161
scer|YDR145W         -----------------------QKLQSITSDKVAA-AK-------NNGNNV--ASLNTKK------------------- 539
tgon|TGME49_244160   RRQEGDPSESLKALVSRQQETHTRLFHQILQEQKEG-ISGGL-SRGEGGRKIRIRTLVSQKGSAQKGVSWAPG-----DR 242
chot|Chro.70325      -----------------YPDTKLEEFCETISQRKEG-RSGAGTLESEEGNAVSNSGLLSIKLFNTE--RFKSN------S 137

bmic|BMR1_02g03651   IAIRKIRTRNHTHLHTSTGSETDSISVIS-------------------FVSDSQ-------------------------- 201
hsap|ENSG00000120656 -------------------------------------------------------------------------------- 161
scer|YDR145W         -------------------------------------------------------------------------------- 539
tgon|TGME49_244160   EEKKRVRGGDC----TEAGEDSDGRSSASPQGGEGETAETGVSAKKSCVASDGRPDAAAASVSTDLRPSGEEQTEARGGQ 318
chot|Chro.70325      KIMNNLRNSSI--------------------------------------------------------------------- 148
```

Figure S3.16. TBP (trimmed)

```
etht|ETH2_1220200    GGGHELLSVGGGGAAA-----------AAAAAAVAAGAAQQQQQPVQVQNVIASGRF----------------------- 171
sneu|SN3_00300415    ASAGNVAAVAA--AAAPLS-------RAAGRTAVQPLPELPAPLLPELQNVVASAQAAWVAGPAEKGTAAEAAGRQTLHC 329
gnip|GNI_108050      ------LAGGPMI-----------SCGNAT----AEALSVEQYQVPAVRVVNC-----------V--------------- 65
cvel|Cvel_17608      -----------------MDRTERLHDQLALSSSSADQSDLPRPRSPTVSNLICSVQT----------------------- 40
tgon|TGME49_258680   LSAESLLALAPTHATQIEQREERISVLSKEEIRASMPPLFSPPTVPEVQNVVASARVTWSSAALD--------------- 319
tetr|Q22V65          -AQSNSIDQN-----------K--NKNNILSTIETMDKSISEDLYPKLQNIVSTVNL----------------------- 62
hsap|ENSG00000112592 -FHSQTLTTAPLPGTTPLY-PS--PMTPMTPITPATPASESSGIVPQLQNIVSTVNL----------------------- 174
scer|YER148W         -FQSE----------------E--DIKR-AAPESEKDTSATSGIVPTLQNIVATVTL----------------------- 76
htar|Htart_000366600 --------------SDKKC-NS--DISTTAELS-RAETISVDGVILTIHNIICSANL----------------------- 191
pber|PBANKA_1105800  --------------NDDNK-FS--NI---------KNKLIHKDIPLKIHNIISSANL----------------------- 118
pfal|PF3D7_0506200   --------------NEKEK-SD--DL---------KNKLVHKNISLNIHNIISSANL----------------------- 160
vbra|Vbra_6491       -SPESSLALA-VPSGASAA-AA--DAIDSSESALVPSAEPPPEMKPRVQNLIASVQL----------------------- 77
tequ|BEWA_045490     -LENEALLLD-IASASRNS-KS--FIEKFAKYSHILKTSSTETSTPVVQNIVASVHL----------------------- 136
bmic|BMR1_03g01240   -DKSQEI------TTFKVD-KP--ILSKIS----SLTTIHSGYRSPTVQNIVASVYI----------------------- 109
chot|ChTU502y2012_42 -VNEK---------IIEAS-NS--IIENSNEI--VDLTIRNESITPEIQNIIASVHL----------------------- 81

etht|ETH2_1220200    ----VLE------GPRGSLDLRRIAISCRFAEYNPRKVNACIVRLRRPRCTAMIFRS----------------------- 218
sneu|SN3_00300415    SSRWARGGEGGGTSENMRLDLRQLAISCRCVEYNPRKINACIVRLRRPKCTALIFRTGGKGLLLGFLLLMLLLCCCCCCS 409
gnip|GNI_108050      -VAF---------YTGCQLDLRLVTVSTRNVEYNPRKLNAAVVRFVDPRLTASVFPN----------------------- 112
cvel|Cvel_17608      ---------------GCYLDLKRVYFALRNTEYNPKRTNAVIFRLQDPSVTAIIYEK----------------------- 82
tgon|TGME49_258680   -ANW---------KQEVKLDLRHLAISCRFAEYNPRKINACIVRLRNPKCTVLVFRS----------------------- 366
tetr|Q22V65          ---------------STKLDLKQIALRARNAEYNPKRFAAVIMRLRDPKTTALIFAS----------------------- 104
hsap|ENSG00000112592 ---------------GCKLDLKTIALRARNAEYNPKRFAAVIMRIREPRTTALIFSS----------------------- 216
scer|YER148W         ---------------GCRLDLKTVALHARNAEYNPKRFAAVIMRIREPKTTALIFAS----------------------- 118
htar|Htart_000366600 ---------------GTQIDLRLVAISIRNAEYNPKKANTLIVRLNKPCSTALIFQT----------------------- 233
pber|PBANKA_1105800  ---------------DAEINLRLVAVSIKSAEYNPSKINTLIIRINNPKCTALIFKS----------------------- 160
pfal|PF3D7_0506200   ---------------CIDINLRLVAVSIRNAEYNPSKINTLIIRLNKPQCTALIFKN----------------------- 202
vbra|Vbra_6491       ---------------GRELDLKSIALSTRNSEFNPKKINAVVMRIREPRCTALVFRT----------------------- 119
tequ|BEWA_045490     ---------------GRELDLREIAISTRNAEYNPKKFNALVLRMHNPKCTGLIFRT----------------------- 178
bmic|BMR1_03g01240   ---------------GIDIDLRQVAISTRNAEYNPKKVNALIFRLLKPKCTSLMFQT----------------------- 151
chot|ChTU502y2012_42 ---------------QCELDLRMIAISARNAEYNPKKVNAVVMRLRDPKCTGLLFRS----------------------- 123

etht|ETH2_1220200    -----GRVMLTGAQSEAAALRAARLLVKMLQAIFCPKPPVAAAAAAAAEPQQLLQLLQLPPPETQEAPGDPAAAAGPLAL 293
sneu|SN3_00300415    AAAAAGRVMLTGARSEEDVGRAARLLAKVLTYAYTNQCDALFRKQRRAA-----------------AVRDTAAADGDVVV 472
gnip|GNI_108050      -----GRVTLAGRASLPAIKKTAKTLAKLLQK------------------------------------------------ 139
cvel|Cvel_17608      -----GRVLITSAQSEAEARLAGKKIIRLLQL------------------------------------------------ 109
tgon|TGME49_258680   -----GRLIITGARSDTEAERAARLTARMLTFAYCGGSTKAPSLENQ--------------------------------- 408
tetr|Q22V65          -----GKMVCTGAKTEEDSNRAARKYAKIIQK------------------------------------------------ 131
hsap|ENSG00000112592 -----GKMVCTGAKSEEQSRLAARKYARVVQK------------------------------------------------ 243
scer|YER148W         -----GKMVVTGAKSEDDSKLASRKYARIIQK------------------------------------------------ 145
htar|Htart_000366600 -----GRIMLTGAQTINDAIYGCRKVGKIIKK------------------------------------------------ 260
pber|PBANKA_1105800  -----GRIMLTGTKSKTDSIKGCKKIGKIIKI------------------------------------------------ 187
pfal|PF3D7_0506200   -----GRIMLTGTRTKKDSIMGCKKIAKIIKI------------------------------------------------ 229
vbra|Vbra_6491       -----GRMMISGSKTEKDAKLGGKRMARIIQK------------------------------------------------ 146
tequ|BEWA_045490     -----GRIIITGSRTTEAARLGAKRMAKIIRRV----------------------------------------------- 206
bmic|BMR1_03g01240   -----GRIMITGAKTIEDAKIGAKRIVKLLLKV----------------------------------------------- 179
chot|ChTU502y2012_42 -----GRLMITGARLENDAKLGGKKMAKICQK------------------------------------------------ 150

etht|ETH2_1220200    LSESSESSTATAAAAAAAGAASAPAAAAAAAGAAAAA-----------------------AAAPEQQQLAVR-------- 342
sneu|SN3_00300415    SSAEA------GGNAVRGGGSDAPAAAAAAGGEDAAASASLTWDEAGTTSAAAAAGAASARAAPAASREERGRGASGRAA 546
gnip|GNI_108050      -------------------------------------------------------------------------------- 139
cvel|Cvel_17608      -------------------------------------------------------------------------------- 109
tgon|TGME49_258680   -----------------------PALALPA------------CSENGTSDISE--------------------------- 426
tetr|Q22V65          -------------------------------------------------------------------------------- 131
hsap|ENSG00000112592 -------------------------------------------------------------------------------- 243
scer|YER148W         -------------------------------------------------------------------------------- 145
htar|Htart_000366600 -------------------------------------------------------------------------------- 260
pber|PBANKA_1105800  -------------------------------------------------------------------------------- 187
pfal|PF3D7_0506200   -------------------------------------------------------------------------------- 229
vbra|Vbra_6491       -------------------------------------------------------------------------------- 146
tequ|BEWA_045490     -------------------------------------------------------------------------------- 206
bmic|BMR1_03g01240   -------------------------------------------------------------------------------- 179
chot|ChTU502y2012_42 -------------------------------------------------------------------------------- 150

etht|ETH2_1220200    ---EGPPS-------PEKALGAP--------------AAAQGAPQS--STENCSSKQICSSKQSSSSSSSKQNCSSIRLA 396
sneu|SN3_00300415    GRGEGDVGRIETAAEKEEELEGMADGSSNSRSRPEIEAEQQDLPLARRKRRNDSDQLLQETEENKKTKEQQQHQVLCLQ 626
gnip|GNI_108050      -----------------------------------------------------------------------SGHPEVTFN 148
cvel|Cvel_17608      -----------------------------------------------------------------------SVHPEANFC 118
tgon|TGME49_258680   RCGESTVCP----------------A------------------------GPNSESLVLLEA------EKNKRTKKLCLR 460
tetr|Q22V65          -----------------------------------------------------------------------IGFP-VQFK 139
hsap|ENSG00000112592 -----------------------------------------------------------------------LGFP-AKFL 251
scer|YER148W         -----------------------------------------------------------------------IGFA-AKFT 153
htar|Htart_000366600 -----------------------------------------------------------------------VTKKPVILK 269
pber|PBANKA_1105800  -----------------------------------------------------------------------VTNQNIRLK 196
pfal|PF3D7_0506200   -----------------------------------------------------------------------VTKDKVKFC 238
vbra|Vbra_6491       -----------------------------------------------------------------------VGFPDVVFA 155
tequ|BEWA_045490     -----------------------------------------------------------------------LGGDDFAFR 215
bmic|BMR1_03g01240   -----------------------------------------------------------------------LKSENIRFK 188
chot|ChTU502y2012_42 -----------------------------------------------------------------------SGFPKVKFT 159

etht|ETH2_1220200    DFAVENIVATADCGLPVRLEG------------------LVYRYSPTP------------SLKAVLLIFVSGKVVITGCR 446
sneu|SN3_00300415    NLKIENVVVSADCGVPVRLEGLAFDHKEFCSYEPELFPGLVYRYNPAE------------GVKAVLLVFVSGKLVITGCR 694
gnip|GNI_108050      NFKICTLVCTANCGFPVRLENIAQDHGPFCSYEPEIFCGLVYRFNPRP------------ELKATALIFVSGRMVISGCT 216
cvel|Cvel_17608      NFKVESLMANGDVCHPIRLEELAKANPAYASYEPEVFSGLVWQSI---------------KPYARVLIWVTGSVSINGQV 183
tgon|TGME49_258680   DFKIENVVASADCGVPVRLEGLAFEHKEFSSYEPELFSGLVYRYNPTA------------SLKAVLLVFVSGKVVITGCK 528
tetr|Q22V65          DFKIQNIVGSTDVKFPINLDHLEQDHKKFVQYEPEIFPGLIYREF---------------NTKIVLLIFVSGKIVLTGAK 204
hsap|ENSG00000112592 DFKIQNMVGSCDVKFPIRLEGLVLTHQQFSSYEPELFPGLIYRMI---------------KPRIVLLIFVSGKVVLTGAK 316
scer|YER148W         DFKIQNIVGSCDVKFPIRLEGLAFSHGTFSSYEPELFPGLIYRMV---------------KPKIVLLIFVSGKIVLTGAK 218
htar|Htart_000366600 NFKIENMIAKADCNMPVRLESLAHDHKEYCNYEPELFAGLIFRHNPTG------------QQKSVILIFVSGKMVISGCK 337
pber|PBANKA_1105800  NFKIENIIASANCNIPVRLEMLAHDHKEYCNYEPELFAGLVYRYKPAS------------NLKSVILIFVSGKIIITGCK 264
pfal|PF3D7_0506200   NFKIENIIASANCNIPIRLEVLAHDHKEYCNYEPELFAGLVYRYKPTS------------NLKSVILIFVSGKIIITGCK 306
vbra|Vbra_6491       NFKVENVIASADVGFPVRLEGLAFDHRERCSYEPELFSGCVFRFRDVLKVKDERVEGGEKACSCTALVFVSGKVSLSGAR 235
tequ|BEWA_045490     NFKIENIIATFNCNVPIRLEVLSSEHEDLCNYEPEFFAGLVFRFSLTD------------TSEAVLLIFVSGNVIITGCK 283
bmic|BMR1_03g01240   NFKIENIIATADCCLPVRLESLARDYREFCSYEPELFAGLVYRCPPTR------------NTKAVLLVFVSGKIIITGCK 256
chot|ChTU502y2012_42 NFKMENIIATADCKFPIRLEGLAYDHRDFCNYEPELFPGLVYRYHPDN-----------SPTKAVLLLFVSGKVIVTGCK 228

etht|ETH2_1220200    AEAEVEEVFNSFFPVLLKYHT--------------------- 467
sneu|SN3_00300415    SFADAVSVFHATYPLLIRYHC--------------------- 715
gnip|GNI_108050      SPRDANEVFHSLYAVLYRYRA--------------------- 237
cvel|Cvel_17608      SQAEVQEVFRKIVELVRPFRS--------------------- 204
tgon|TGME49_258680   SLSEVNHVFESLYPVLVRYHQ--------------------- 549
tetr|Q22V65          TRENINKAFQKIYWVLYNYQKKDYRGANLHNQNLNIKPSIKN 246
hsap|ENSG00000112592 VRAEIYEAFENIYPILKGFRKTT------------------- 339
scer|YER148W         QREEIYQAFEAIYPVLSEFRKM-------------------- 240
htar|Htart_000366600 SVQKLNTVFQDVYNILKAYSI--------------------- 358
pber|PBANKA_1105800  SLQKLNTVFQDIYNVLVQYKS--------------------- 285
pfal|PF3D7_0506200   SVNKLYTVFQDIYNVLIQYKN--------------------- 327
vbra|Vbra_6491       SEEELHMAFRHLYPILLNYNKL-------------------- 257
tequ|BEWA_045490     SMEEVHHVFNTMHPILLRYQE--------------------- 304
bmic|BMR1_03g01240   SMREVHRVFTYMYPILRQYQV--------------------- 277
chot|ChTU502y2012_42 NYQEICNVFDNIYPVLCQYRK--------------------- 249
```

Figure S3.17. UBF (trimmed)

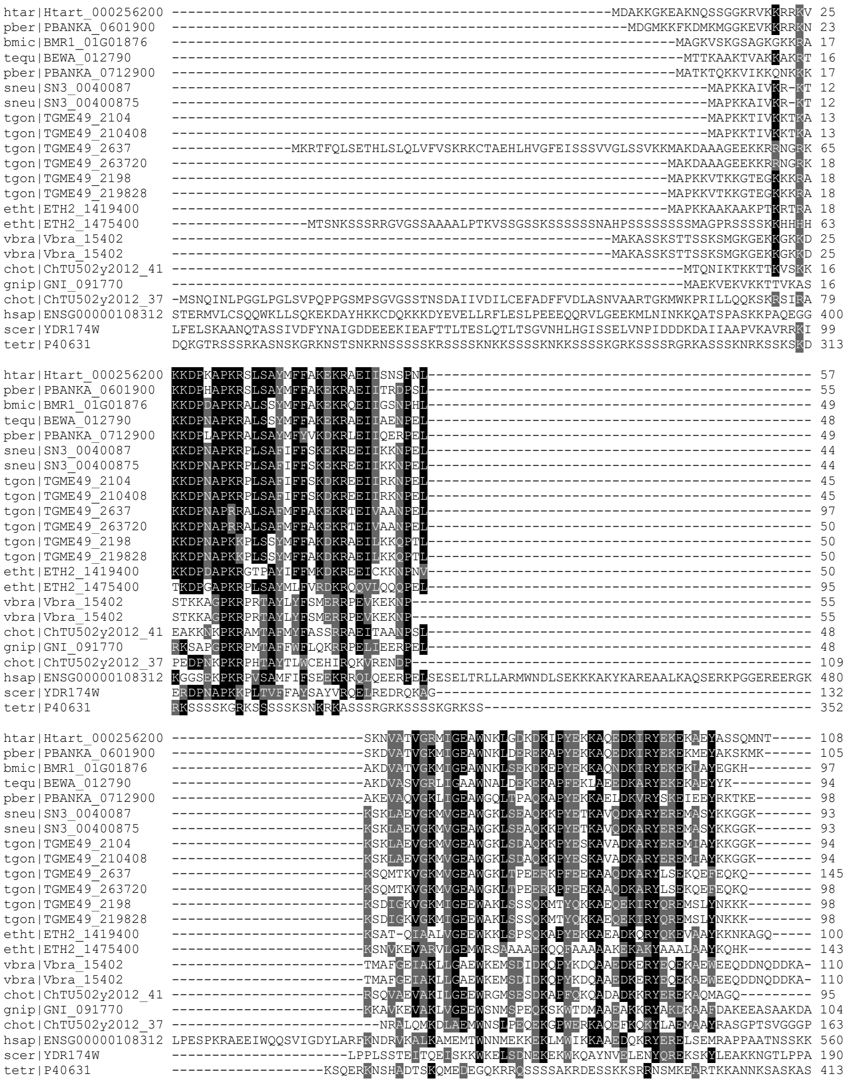

Figure S3.18. C34 / RPC6

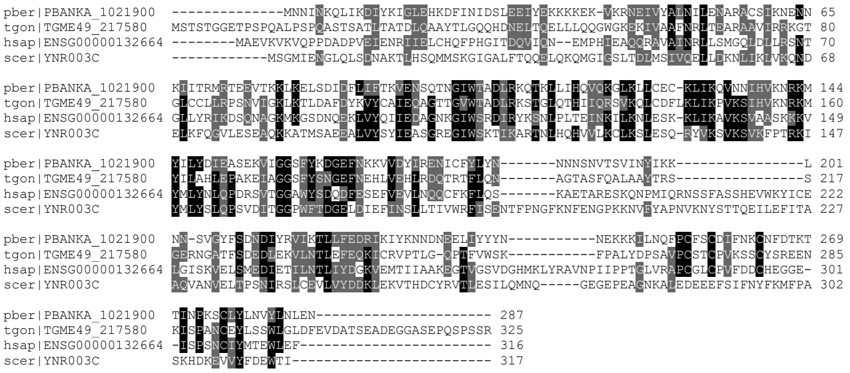

Table S1. Components of RNA polymerase I transcription machinery.

P. falciparum 3D7 piggyBac insertion mutagenesis - mutagenesis index score; interval: [0.119;1]; {essential; dispensable}
P. falciparum 3D7 piggyBac insertion mutagenesis - mutant fitness score; interval: [-4.094; 2.769]
T. gondii genome-wide CRISPR screen – phenotype score; interval: [-6.89; 2.96]. **WARNING**: The TGME49 genes do not have data for this experiment. Instead, we are showing data for this same gene(s) from the reference *Toxoplasma gondii* GT1 strain. This may or may NOT accurately represent the gene shown.

				piggyBac			PlasmoGEM		CRISPR Screen
				Mutagenesis Index	Mutant Fitness				Phenotype
	Yeast	Human	P. falciparum	Score	Score	P. berghei	Gene deletion	T. gondii	Score
Core Pol I	RPA1		PF3D7_0509400	0.126	-2.929	PBANKA_1109000	refractory	TGME49_244880	-4.35
	RPA2		PF3D7_1134700	0.12	-2.941	PBANKA_0913800	refractory	TGME49_297530	-4.85
	RPA12		PF3D7_0407300	0.119	-3.016	PBANKA_1004900	refractory	TGME49_250060	-3.75
	RPAC1		PF3D7_1143300	0.129	-2.889	PBANKA_0905700	refractory	TGME49_267390	-5.2
	RPAC2		PF3D7_1415200	0.132	0	PBANKA_1027500	refractory	TGME49_261540	-3.54
	RPABC1		PF3D7_1364800	0.224	-2.651	PBANKA_1140600	refractory	TGME49_240590	-5.78
	RPABC2		PF3D7_0303300	0.147	-3.103	PBANKA_0401900	refractory	TGME49_270780	-4.91
	RPABC3		PF3D7_1213700	0.133	-3.188	PBANKA_1429500	refractory	TGME49_233720	-3.75
	RPABC4		PF3D7_1342700	nda	nda	PBANKA_1355800	refractory	TGME49_254140	-4.69
	RPABC5		PF3D7_0708100	0.126	0	PBANKA_0805400	refractory	TGME49_217560	-3.16
Heterodimer	RPA49		absent	—	—	absent	—	absent	—
	RPA34		absent	—	—	absent	—	absent	—
Stalk	RPA43		PF3D7_0208700	0.872	-1.746	PBANKA_0305800	attenuated	TGME49_246630	-5.32
	RPA14	absent	absent	—	—	absent	—	absent	—
Associated Factor	RRN3		absent	—	—	absent	—	TGME49_238880	-3.78
Promoter recognition complex human SL1		TAF1A	absent	—	—	absent	—	absent	—
		TAF1B	absent	—	—	absent	—	absent	—
		TAF1C	absent	—	—	absent	—	absent	—
		TAF1D	absent	—	—	absent	—	absent	—
TATA-binding protein	TBP		PF3D7_0506200	0.175	-2.784	PBANKA_1105800	refractory	TGME49_258680	-3.73
Yeast core factor	RRN11		absent	—	—	absent	—	absent	—
	RRN7		absent	—	—	absent	—	absent	—
	RRN6		absent	—	—	absent	—	absent	—
Yeast UAF	UAF30		PF3D7_0518200	0.353	0	PBANKA_1233000	nda	TGME49_273930	0.36
	RRN5		absent	—	—	absent	—	absent	—
	RRN9		absent	—	—	absent	—	absent	—
	RRN10		absent	—	—	absent	—	absent	—
High mobility box group proteins (HMGB)		UBF	PF3D7_1202900	—	—	PBANKA_0601900	—	TGME49_210408	-0.28
	HMGB1		PF3D7_1202900	0.339	-2.546	PBANKA_0601900	nda	TGME49_210408	-0.28
	HMGB2		PF3D7_0817900	0.368	-3.27	PBANKA_0712900	attenuated	TGME49_210408	-0.28
	HMGB3		PF3D7_1205800	1	-2.862	PBANKA_0604500	disrupted	TGME49_203950	-5.25
	HMGB4		PF3D7_1359200	1	0.402	PBANKA_1135500	disrupted	TGME49_217500	0.89
FACT complex	Spt16		PF3D7_0517400	0.123	-2.94	PBANKA_123220	refractory	TGME49_221670	-4.55
	Pob3	SSRP1	PF3D7_1441400	0.12	-3.034	PBANKA_130530	refractory	TGME49_261460	-4.17
