# Supplementary material for: The ribosomal RNA transcription landscapes of Plasmodium falciparum and related apicomplexan parasites
Source: Nucleic Acids Res. 2025 Jul 8;53(13):gkaf641. doi: 10.1093/nar/gkaf641 (PMC12235521; doi:10.1093/nar/gkaf641)
Supplement: gkaf641_Supplemental_Files [file gkaf641_supplemental_files.zip › Supp_methods_rev2.pdf]

OrthoMCL search.

We obtained representative RNA polymerase I core components, accessory factors, RRN3, and promoter-binding proteins by text-searching for the human and yeast proteins at OrthoMCL (Release 6.20, 21 Feb 2024). Each orthologous gene group was then searched for the relevant homolog proteins of the queried alveolates organisms within that ortholog group. If absent from the human or yeast ortholog groups, Basic Local Alignment Search Tool P (BlastP) was used to identify the protein in question.

Multiple Protein sequence alignments.

Clustal Omega multiple protein alignments and protein identity matrices were performed at https://www.ebi.ac.uk/jdispatcher/msa/clustalo [1]. Sequence alignments were colored (boxshade) at https://www.bioinformatics.org/smS1/color_align_cons.html using the default settings for shading, setting the percentage at 60% or 100%.

ClustalW alignments of with proteins from different ortholog groups (for example RPA12, RPB9, and RPC11) were performed at https://www.genome.jp/tools-bin/clustalw.

Modelling.

Sequence alignments of all Pol I subunits from *S. cerevisiae*, *H. sapiens*, *T. thermophila*, *T. gondii* and *P. falciparum* were performed with Clustal Omega (https://www.ebi.ac.uk/jdispatcher/msa/clustalo) [2] to identify substantial variations of parasite Pol I subunits compared to its yeast counterpart.

AlphaFold 3 (AlphaFold Server, https://alphafoldserver.com/) [3] was used for structural predictions. However, complete polymerases exceeded the size limitations. Therefore, partial structure predictions comprising combinations of parasite Pol I subunits were determined (see **Table SM1**). All predictions contained subunits RPA1 and RPA2 to be able to assemble a complete prediction for each organism for consistency. To build a complete model of *P. falciparum* Pol I, subunit RPA1 and RPA2 were assembled from all three or two different predictions whereas the other subunits were taken from one prediction, respectively. To evaluate the confidence of the predictions, the pTM (predicted template modeling) and ipTM (interface predicted template modeling) scores from AlphaFold of the whole predictions and the single subunits (see **Table SM1**) were considered. Furthermore, pLDDT (predicted local distance difference test) scores, PAE ("predicted aligned error" sometimes also referred to as "expected position error", or "expected distance error") plots of the three separate predictions (**Figure SM1**) and the five top-scoring predictions of each job were comparatively evaluated to define contributions to the composite model. Additionally, parts of the polymerase which were inconsistently predicted in the five top-scoring results of each sub-complex were considered to be of low confidence. The pLDDT score is a per-atom confidence estimate, easily visualized within the predictions. pTM and ipTM are derivates from the template modeling score and measure the accuracy of the entire structure [4, 5]. The pTM scores the overall fold of the complex whereas the ipTM scores the accuracy of the predicted relative positions of the individual subunits. The PAE plot shows the estimated error of the relative position between two specific residues. All predictions were compared to the monomeric yeast

structure (PDBs 5M3M and 5M64) and human elongation complex (PDB 7OB9) in detail. To determine whether substantial insertions in parasite Pol I subunits might share features known from other transcription system of factors, predictions of *P. falciparum* subunits insertions were isolated and subjected to 3D similarity searches using via the Dali server (http://ekhidna2.biocenter.helsinki.fi/dali/) [6] and PDBeFold (https://www.ebi.ac.uk/msd-srv/ssm/cgi-bin/ssmserver) [7]. Independently, sequence-based domain homology prediction searches of substantial insertions were performed using HHpred (https://toolkit.tuebingen.mpg.de/tools/hhpred) [8, 9]. Partial structures were manually combined using Coot [10] and ChimeraX [11] to obtain a composite 3D model of parasite Pol I.

Table SM1. Scores from AlphaFold for each model - overall and the used chains

pTM > 0.5 = overall predicted fold for the complex might be similar to the true structure
ipTM > 0.8 = confident high-quality predictions
ipTM 0.8-0.6 = grey zone
ipTM < 0.6 = suggest likely a failed prediction
color scheme by pIDDT values (>90 = very high; 90-70 = confident; 70-50 = low; <50 = very low)

Prediction	Overall / chain		pTM	ipTM
Plasmodium falciparum				
predication #1	overall		0.72	0.85
includes RPA1, RPA2, RPAC1, RPAC2, RPABC4, RPABC5				
predication #2	overall		0.68	0.77
includes RPA1, RPA2, RPA12, RPABC1				
predication #3	overall		0.69	0.81
includes RPA1, RPA2, RPABC2, RPA43, RPABC3				
composite	RPA1	from #1, #2, and #3 (#1: aa 911-1227; #2: aa 116-530, aa 1231-1557, aa 1598-2346 and aa 2503-2895; #3: aa 1-112, aa 534-907 and aa 2899-2914)	0.58 / 0.58 / 0.57	0.89 / 0.73 / 0.82
	RPA2	from #1 and #3 (#1: aa 1-887, aa 905-1286 and aa 1302-1384; #3: aa 1388-1517)	0.75 / 0.74	0.89 / 0.77
	RPABC1	from #2	0.75	0.70
	RPABC2	from #3 (aa 1-18 and 45-153)	0.52	0.64
	RPABC3	from #3	0.68	0.63
	RPABC4	from #1	0.79	0.85
	RPABC5	from #1	0.66	0.71
	RPAC1	from #1	0.44	0.67
	RPAC2	from #1	0.62	0.74
	RPA43	from #3	0.64	0.61
	RPA12	from #2 (aa 71-320)	0.19	0.41
Toxoplasma gondii				
predication #1	overall		0.75	0.87
includes RPA1, RPA2, RPAC1, RPAC2, RPABC4, RPABC5, RPABC3				
predication #2	overall		0.76	0.85
includes RPA1, RPA2, RPA12, RPABC1, RPABC3				
predication #3	overall		0.73	0.76
includes RPA1, RPA2, RPABC1, RPABC2, RPA43				
Tetrahymena thermophila				
predication #1	overall		0.84	0.84
includes RPA1, RPA2, RPAC1, RPAC2, RPABC1, RPABC2, RPABC3 (isoform 1), RPABC5, RPA12, RPA49, RPA34				
predication #2	overall		0.84	0.84
includes RPA1, RPA2, RPAC1, RPAC2, RPABC1, RPABC2, RPABC3 (isoform 2), RPABC5, RPA12, RPA49, RPA34				
predication #3	overall		0.85	0.84
includes RPA1, RPA2, RPAC1, RPAC2, RPABC1, RPABC2, RPABC3 (isoform 1), RPABC5, RPA43, RPA12				
predication #4	overall		0.85	0.84
includes RPA1, RPA2, RPAC1, RPAC2, RPABC1, RPABC2, RPABC3 (isoform 2), RPABC5, RPA43, RPA12				

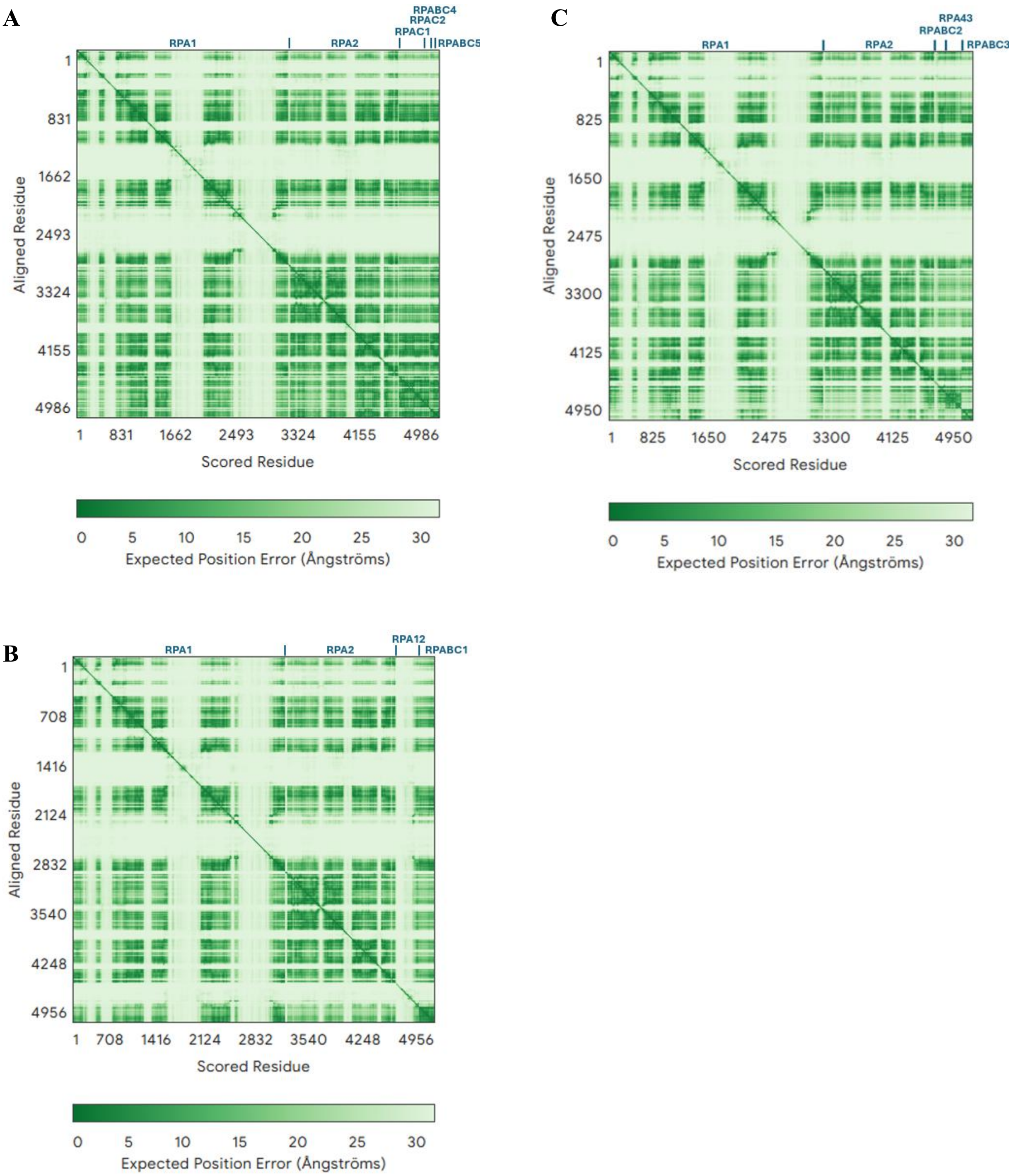

Figure SM1. Expected distance error plots of the three separate predictions. A. *P. falciparum* prediction #1, includes RPA1, RPA2, RPAC1, RPAC2, RPABC4, RPABC5. B. *P. falciparum* prediction #2, includes RPA1, RPA2, RPA12, RPABC1. C. *P. falciparum* prediction #3, includes RPA1, RPA2, RPABC2, RPA43, RPABC3.

References:

1. Madeira, F., et al., *Search and sequence analysis tools services from EMBL-EBI in 2022.* Nucleic Acids Res, 2022. **50**(W1): p. W276-W279.
2. Madeira, F., et al., *The EMBL-EBI Job Dispatcher sequence analysis tools framework in 2024.* Nucleic Acids Res, 2024. **52**(W1): p. W521-W525.
3. Abramson, J., et al., *Accurate structure prediction of biomolecular interactions with AlphaFold 3.* Nature, 2024. **630**(8016): p. 493-500.
4. Zhang, Y. and J. Skolnick, *Scoring function for automated assessment of protein structure template quality.* Proteins, 2004. **57**(4): p. 702-10.
5. Xu, J. and Y. Zhang, *How significant is a protein structure similarity with TM-score = 0.5?* Bioinformatics, 2010. **26**(7): p. 889-895.
6. Holm, L., et al., *DALI shines a light on remote homologs: One hundred discoveries.* Protein Sci, 2023. **32**(1): p. e4519.
7. Krissinel, E. and K. Henrick, *Secondary-structure matching (SSM), a new tool for fast protein structure alignment in three dimensions.* Acta Crystallogr D Biol Crystallogr, 2004. **60**(Pt 12 Pt 1): p. 2256-68.
8. Zimmermann, L., et al., *A Completely Reimplemented MPI Bioinformatics Toolkit with a New HHpred Server at its Core.* J Mol Biol, 2018. **430**(15): p. 2237-2243.
9. Gabler, F., et al., *Protein Sequence Analysis Using the MPI Bioinformatics Toolkit.* Curr Protoc Bioinformatics, 2020. **72**(1): p. e108.
10. Emsley, P., et al., *Features and development of Coot.* Acta Crystallogr D Biol Crystallogr, 2010. **66**(Pt 4): p. 486-501.
11. Meng, E.C., et al., *UCSF ChimeraX: Tools for structure building and analysis.* Protein Sci, 2023. **32**(11): p. e4792.
